# Controversial causal association between IGF family members and osteoporosis: a Mendelian randomization study between UK and FinnGen biobanks

**DOI:** 10.3389/fendo.2023.1332803

**Published:** 2024-01-08

**Authors:** Jie Tang, Chanjuan Zhao, Sha Lin, Xing Li, Binlu Zhu, Yifei Li

**Affiliations:** Key Laboratory of Birth Defects and Related Diseases of Women and Children of Ministry of Education (MOE), Department of Pediatrics, West China Second University Hospital, Sichuan University, Chengdu, Sichuan, China

**Keywords:** osteoporosis, IGF-1, IGF family, developmental disorders, Mendelian randomization

## Abstract

**Objectives:**

Osteoporosis, a prevalent skeletal disorder characterized by reduced bone strength, is closely linked to the IGF system, crucial for skeletal metabolism. However, the precise nature of this relationship remains elusive. In this study, we employed Mendelian randomization (MR) to unravel the associations between genetically predicted serum IGF system member levels and osteoporosis.

**Methods:**

A two-sample MR approach was employed to investigate these causal associations based on two individual datasets. Predictions of 14 serum levels of IGF system members were made using 11,036,163 relevant Single Nucleotide Polymorphisms (SNPs) within a cohort of 4,301 individuals of European descent. Genetic association estimates for osteoporosis were derived from two publicly available GWAS consortia: the Finnish consortium from the FinnGen biobank, comprising 212,778 individuals of Finnish descent (3,203 cases and 209,575 controls), and the UK consortium from the UK Biobank, including 337,159 individuals of European descent (5,266 cases and 331,893 controls).

**Results:**

According to the UK dataset, IGF-1 levels were associated with a reduced risk of osteoporosis, as indicated by the weighted median method (Odds Ratio [OR] = 0.998, 95% CI = 0.997–1.000, P = 0.032). Additionally, higher levels of IGFBP-3 were linked to a decreased risk of osteoporosis using the Inverse-Variance Weighted (IVW) method (OR = 0.999, 95% CI = 0.998–1.000, P = 0.019), and CTGF levels exhibited a negative association with osteoporosis, as determined by the weighted median method (OR = 0.998, 95% CI = 0.996–0.999, P = 0.004). In the FinnGen dataset, IGF-1 and IGFBP-3 were not identified to be associated with osteoporosis. While, IGF-LR1 levels displayed a negative association with osteoporosis, according to the MR-Egger method (OR = 0.886, 95% CI = 0.795–0.987, P = 0.036), while CYR61 was linked to an increased risk of osteoporosis based on both the weighted median and IVW methods (OR = 1.154, 95% CI = 1.009–1.319, P = 0.037, and OR = 1.115, 95% CI = 1.022–1.215, P = 0.014, respectively).

**Conclusion:**

This study provides compelling evidence that certain IGF family members play a role in the pathogenesis of osteoporosis between different datasets, indicating population specific causal effects between IGF family and osteoporosis. Although the results from both datasets demonstrated that IGF family involved in the pathogenesis of osteoporosis, but the responding key molecules might be various among different population. Subsequent research is warranted to evaluate the potential of these biomarkers as targets for osteoporosis prevention and treatment in specific population.

## Introduction

Osteoporosis poses a significant public health challenge, impacting nearly 200 million individuals and resulting in 8.9 million fractures worldwide annually (1). The clinical definition of osteoporosis is primarily based on bone mineral density (BMD), a surrogate marker of bone strength that is substantially influenced by the peak bone mass attained during childhood and adolescence (2, 3). Osteoporosis results from an imbalance between bone formation and bone resorption. This imbalance is regulated by osteoblasts and osteoclasts. Disruptions in the molecular signals controlling the activity of these cells can lead to excessive bone loss. Although genetic factors influence an individual’s susceptibility to osteoporosis, but the adverse environmental exposure contributes as a dominant role in the pathogenesis of osteoporosis. As a result, early life is a critical phase for establishing lifelong skeletal health, influenced by a multitude of factors (3–5). It is confirmed that maternal factors during pregnancy, such as maternal nutrition, smoking, and alcohol consumption, have been linked to bone health in offspring (6). Thus, the programmed metabolic disorders would be existed throughout lifelong. In the past two decades, the conception of fetal or early life originated diseases had been accepted by investigators. Mechanically, the environmental exposure might result in metabolic and genetic modification dysfunction, leading to long-term impacts, especially in bone and cardiovascular diseases. Importantly, the early life hormonal imbalances can impact bone health (7). Studies have shown that poor nutrition and inadequate growth during childhood can lead to lower peak bone mass, which is a major determinant of bone health later in life (8). Inadequate intake of calcium, vitamin D, and protein during childhood and adolescence can impair bone development. Conditions such as early puberty or delayed puberty may affect peak bone mass. Additionally, endocrine disorders in childhood, such as diabetes or thyroid disorders, can influence bone development (9, 10). Moreover, longitudinal epidemiological studies have provided insights into the long-term effects of early life exposures on bone health. These studies track individuals over time and assess how early life factors influence the risk of osteoporosis in adulthood (11).

The hormone insulin-like growth factor (IGF) family comprises two ligands (IGF-1 and IGF-2), two receptors (IGF-1R and IGF-2R), seven high-affinity binding proteins (IGFBPs 1-7), a substantial group of IGFBP proteases, and a novel category of proteins known as low-affinity IGFBP-related proteins (IGFBP-rPs) (12). This family is widely recognized for its pivotal roles in growth and development, regulating processes such as proliferation, differentiation, metabolism, and cell survival across various tissues, including bone development and hemostasis (13). Among them, CCN1 (also named IGFBP-10), CCN2 (IGFBP-8) and CCN3 (IGFBP-9) which were identified as low-affinity IGFBPs, along with those high-affinity IGFBPs 1-6, together constitute an IGFBP superfamily whose products function in IGF-dependent or IGF-independent modes to regulate skeletal metabolism (13). Fundamental research suggests that the IGF regulatory system plays crucial roles in bone acquisition and maintenance (14, 15). Nevertheless, observational studies and randomized controlled trials examining the association between the IGF regulatory system and osteoporosis (or fracture risk) have yielded conflicting results (16–24). Growth hormone and IGF family members had been identified to be served as important regulators in bone remodeling and metabolism, while the fundamental role had been well established in bone mass maintenance. Previous studies introduced that the impairment of IGF family members would increase the risk of fractures (15, 25, 26). Besides, several single nucleotide polymorphisms had been identified to be involved in osteoporosis patients (15, 25, 26). Moreover, some FDA approved medication had been issued for particular treatment, some integrative therapeutic management would be evaluated since the causal effect between IGF family and osteoporosis was addressed.

If a particular plasma biomarker is directly involved in an underlying pathological process, then inherited variation changing plasma concentrations of this biomarker should affect risk of disease in the direction and magnitude predicted by the plasma concentrations (27). Mendelian randomization (MR) is a valuable analytical approach for establishing causal links between exposures and specific outcomes, which is just such an analytical method to reach a causal inference between a genetically predicted exposure and an outcome, which uses genetic variants that are strongly and solely associated with exposure as instrumental variables (IVs) hence avoiding confounding factors and reverse causality. In this study, we employed a two-sample MR analysis to explore a potential causal relationship between genetically predicted serum IGF system components and osteoporosis.

## Methods

### Study design

This study was design to assess the causal effects of IGF family members in the risk of osteoporosis (OP). The related traits of IGF family members had been identified, and fourteen IGF family members traits included: IGF-1, IGF-1 sR, IGF-2R, IGFBP-1, IGFBP-2, IGFBP-3, IGFBP-4, IGFBP-5, IGFBP-6, IGFBP-7, IGF-LR1, CTGF, WISP-1 and CYR61. First, the effects of fourteen IGF family members and their serum concentration were evaluated to identify the potential Single nucleotide polymorphisms (SNPs) as one sample MR analysis. Then two-sample MR analysis had been completed among OP traits to measure the causal effects of IGF family members in OP pathogenesis in the large sample size trait (finn-b-M13_OSTEOPOROSIS and ukb-a-87). Then, further confirmation had been performed among two OP traits to validate the results.

### Outcome data sources

Data for the genetic associations on osteoporosis were obtained from the publicly available GWAS summary datasets, the FinnGen biobank (Risteys FinnGen R6 - M13_OSTEOPOROSIS) and the Neale lab secondary analysis of UK Biobank phenotypes (https://pheweb.org/UKB-Neale/pheno/20002_1309), which had no sample overlap with each other. GWAS data of osteoporosis from the FinnGen biobank consisted of 212,778 Finnish-descent individuals (3,203 cases and 209,575 controls). Osteoporosis here was determined from hospital episode statistics, including osteoporosis (further divided into postmenopausal, postoophorectomy, drug-induced, idiopathic osteoporosis and so on) with pathological fracture, osteoporosis without pathological fracture and osteoporosis in other diseases (e.g., multiple myelomatosis, endocrine disorders), all of which were classified by the International Statistical Classification of Diseases and Related Health Problems (ICD) 10 codes. Another osteoporosis GWAS obtained from the UK Biobank involved 337,159 European-descent individuals (5,266 cases and 331,893 controls) for self-reported osteoporosis without cancer illness. This study only utilized publicly available summarized results from published genome-wide association studies. No individual-level data were involved.

### Genetic instrument selection

Single nucleotide polymorphisms (SNPs) associated with IGF system members were obtained and selected from the summary statistics of the genome-wide association study (GWAS) of 14 IGF system members in 4,301 participants of European descent from two cohort studies, the KORA study (28) and the INTERVAL study (29). Genetic associations were adjusted for relevant covariates. We extracted SNPs that strongly predicted exposures at the genome-wide significance threshold (P < 1.00E-5) as instrument variants, then clumped based on 1,000 Genomes Project linkage disequilibrium (LD) structure to omit the superposition effect of correlated SNPs (R2 < 0.01 with any other associated SNP within 5Mb).

### Statistical analysis

We hypothesized that there exist causal association (whether inverse or positive) of genetically predicted serum IGF system members with osteoporosis. To obtain a reliable foundation for the MR analysis, the following assumptions were satisfied: the genetic variants used as instrumental variables were associated with the exposure; the genetic variants were not associated with any confounders; and the genetic variants were associated with osteoporosis through the exposure (serum IGF system members) only.

In this study, we used TwoSampleMR packages (version 0.5.6) in R (version 4.0.4) to estimate the effect of each IGF system member on osteoporosis by applying MR Egger regression (30), inverse variance weighted (IVW) (31) and weighted median methods (32). And the codes used for R were available in **Supplementary file 1**. Odds ratios (OR) and 95% confidence intervals (CIs) for osteoporosis were estimated, and a P < 0.05 was considered as statistically significant. To avoid weak IVs, average SNP-specific F-statistics were calculated, and IVs with F-statistics > 10 were considered as strong IVs for MR analysis. All the results of F-statistics and P values for included SNPs had been listed in **Supplementary Table 1**. Regarding directional pleiotropy analyses, we conducted MR-Egger regression methods to evaluate the possible pleiotropic effect by the intercept in MR-Egger regression model (P for intercept < 0.05) (30). Heterogeneity was tested for by applying Cochran’s Q test on the IVW and MR-Egger estimates. We also performed the leave-one-out analysis with the IVW method to evaluate whether the overall estimate was driven by single SNP.

## Results

In accordance with our study design strategy, we investigated the potential causal effects of serum IGF family members’ concentrations on the risk of osteoporosis. We included fourteen molecules in the initial one-sample MR analysis to identify SNPs that might influence their serum concentrations. These molecules were IGF-1 (prot-c-2952_75_2), IGF-1 sR (prot-c-4232_19_2), IGF-2R (prot-c-3676_15_3), IGFBP-1 (prot-c-2771_35_2), IGFBP-2 (prot-c-2570_72_5), IGFBP-3 (prot-c-2571_12_3), IGFBP-4 (prot-c-2950_57_2), IGFBP-5 (prot-c-2685_21_2), IGFBP-6 (prot-c-2686_67_2), IGFBP-7 (prot-c-3320_49_2), IGF-LR1 (prot-a-1455), CTGF (prot-c-2975_19_2), WISP-1 (prot-c-3057_55_1), and CYR61 (prot-a-758).

Out of these fourteen serum concentration traits related to IGF family members, which had previously been substantiated in published studies, thirteen of them displayed more than one genome-wide significant SNP site. Further details, including the outcomes of the clumping process for LD-independent SNPs related to the exposure, are provided in the supplementary figures. Notably, all calculated F-statistics exceeded a value of ten, indicating that the results were less susceptible to the bias associated with weak instruments. The essential information regarding the enrolled traits has been summarized in **Table 1**.

**Table 1:** All GWAS datasets selected in this article.

Trait	GWAS id	Sample size	Number of SNPs
**Non-cancer illness code self-reported: osteoporosis**	ukb-a-87	337,159	10,894,596
**Osteoporosis**	finn-b-M13_OSTEOPOROSIS	212,778	16,380,452
**IGF-1**	prot-c-2952_75_2	1,000	501,428
**IGF-1 sR**	prot-c-4232_19_2	1,000	501,428
**IGF-2R**	prot-c-3676_15_3	1,000	501,428
**IGF-LR1**	prot-a-1455	3,301	10,534,735
**IGFBP-1**	prot-c-2771_35_2	1,000	501,428
**IGFBP-2**	prot-c-2570_72_5	1,000	501,428
**IGFBP-3**	prot-c-2571_12_3	1,000	501,428
**IGFBP-4**	prot-c-2950_57_2	1,000	501,428
**IGFBP-5**	prot-c-2685_21_2	1,000	501,428
**IGFBP-6**	prot-c-2686_67_2	1,000	501,428
**IGFBP-7**	prot-c-3320_49_2	1,000	501,428
**CTGF**	prot-c-2975_19_2	1,000	501,428
**WISP-1**	prot-c-3057_55_1	1,000	501,428
**CYR61**	prot-a-758	3,301	10,534,735

**Figure 1:**
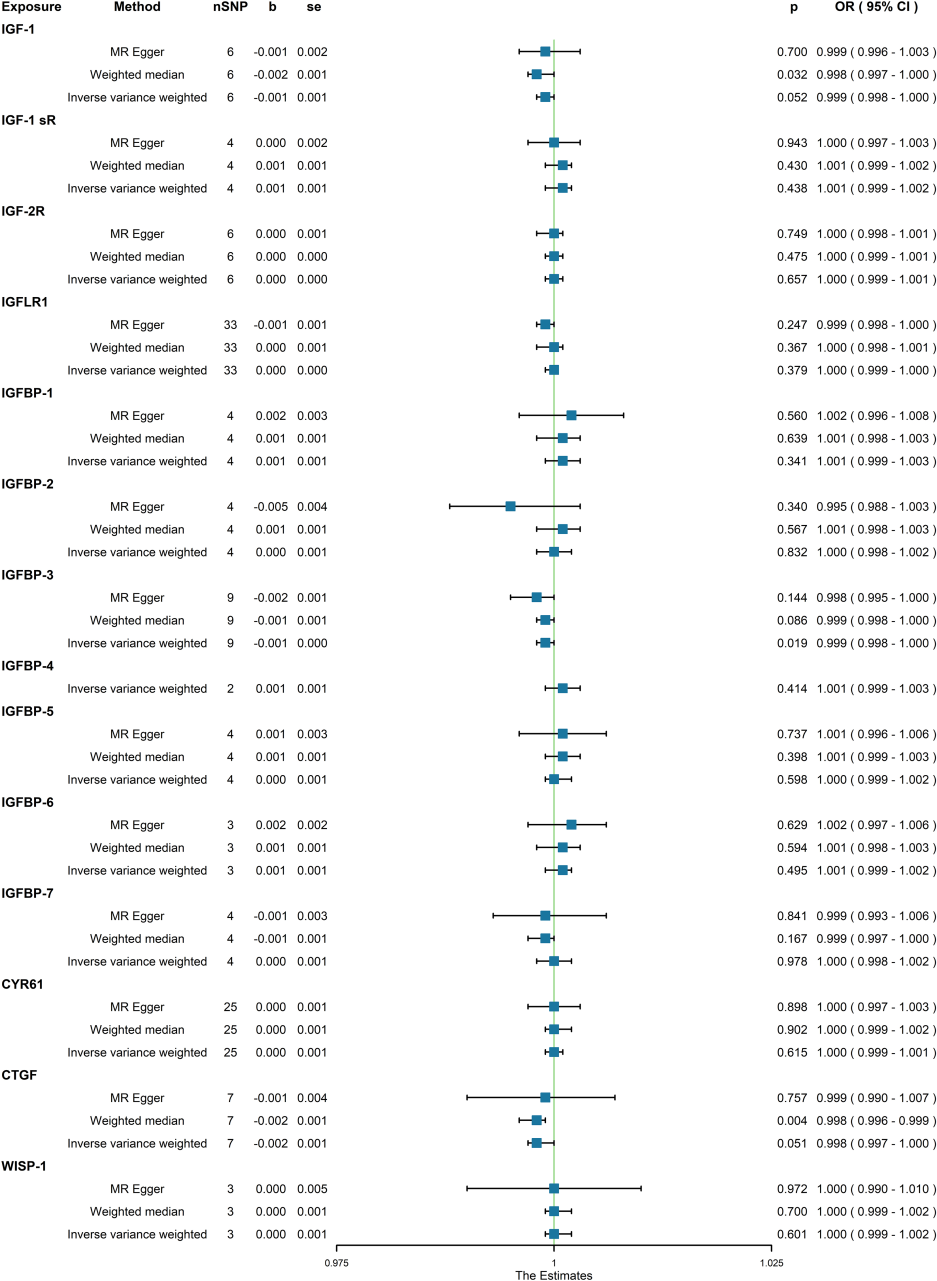
Association between genetical causes of IGF family members and osteoporosis from the UK trait.

**Figure 2:**
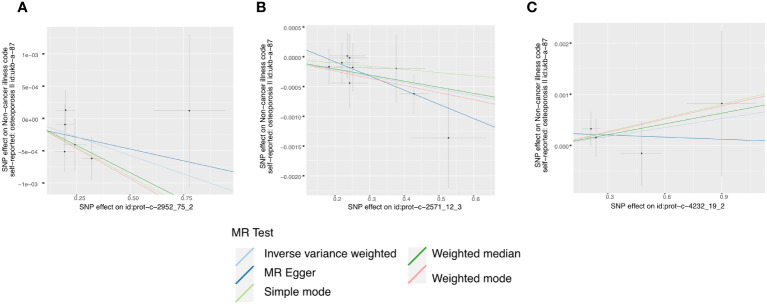
Scatter plots for MR analyses of the causal effect of IGFs on osteoporosis based on UK trait. **(A)**, IGF-1. **(B)**, IGFBP-3. **(C)**, CTGF. Analyses were conducted using the conventional IVW, MBE, WMM, MR-Egger, and MR.RAPS methods. The slope of each line corresponding to the estimated MR effect per method.

In the initial one-step MR analysis, we employed both the MR-Egger and IVW methods. Subsequently, we identified multiple SNPs that reached genome-wide significance (P<1×10-5) among the fourteen IGF family molecules, which were employed to assess their causal effects on osteoporosis. Upon pooling the data, three IGF family molecules were found to be associated with osteoporosis. IGF-1 level is associated with a reduced risk of osteoporosis, as indicated by the weighted median method (odds ratio [OR] = 0.998, 95% CI = 0.997-1.000, P = 0.032) (**Figure 1**). Furthermore, higher levels of IGFBP-3 were also associated with a decreased risk of osteoporosis using the IVW method (OR = 0.999, 95% CI = 0.998-1.000, P = 0.019) (**Figure 1**). Additionally, the CTGF level exhibited a negative association with osteoporosis, according to the weighted median method (OR = 0.998, 95% CI = 0.996-0.999, P = 0.004) (**Figure 1**). We conducted a series of sensitivity analyses to validate the causal associations between each trait and osteoporosis. Heterogeneity was not detected for IGF-1 (MR Egger, Cochran P value = 0.449; IVW, Cochran P value = 0.585) and IGFBP-3 (MR Egger, Cochran P value = 0.978; IVW, Cochran P value = 0.961). However, there appeared to be moderate heterogeneity in the analysis of CTGF (MR Egger, Cochran P value = 0.027; IVW, Cochran P value = 0.049). Notably, we did not find any evidence of potential horizontal pleiotropy for IGF-1, CTGF, and IGFBP-3 (intercepts = -0.000, P = 0.814; intercept = -3.242E-5, P = 0.977; intercept = -0.000, P = 0.377, respectively) when employing the MR-Egger method. Leave-one-out analyses for these two traits suggested that the estimated causal effects were not significantly influenced by any single instrumental variable. Scatter plots depicting the MR analyses of the causal effects of IGFs on osteoporosis with statistical significance are presented in **Figure 2** (A for IGF-1, B for IGFBP-3, and C for CTGF, respectively). All the involved funnel plots, scatter plots and “leave-one out analysis” plots in assessing the association between IGFs family and osteoporosis in UK trait were shown in **Supplementary file 2**.

**Figure 3:**
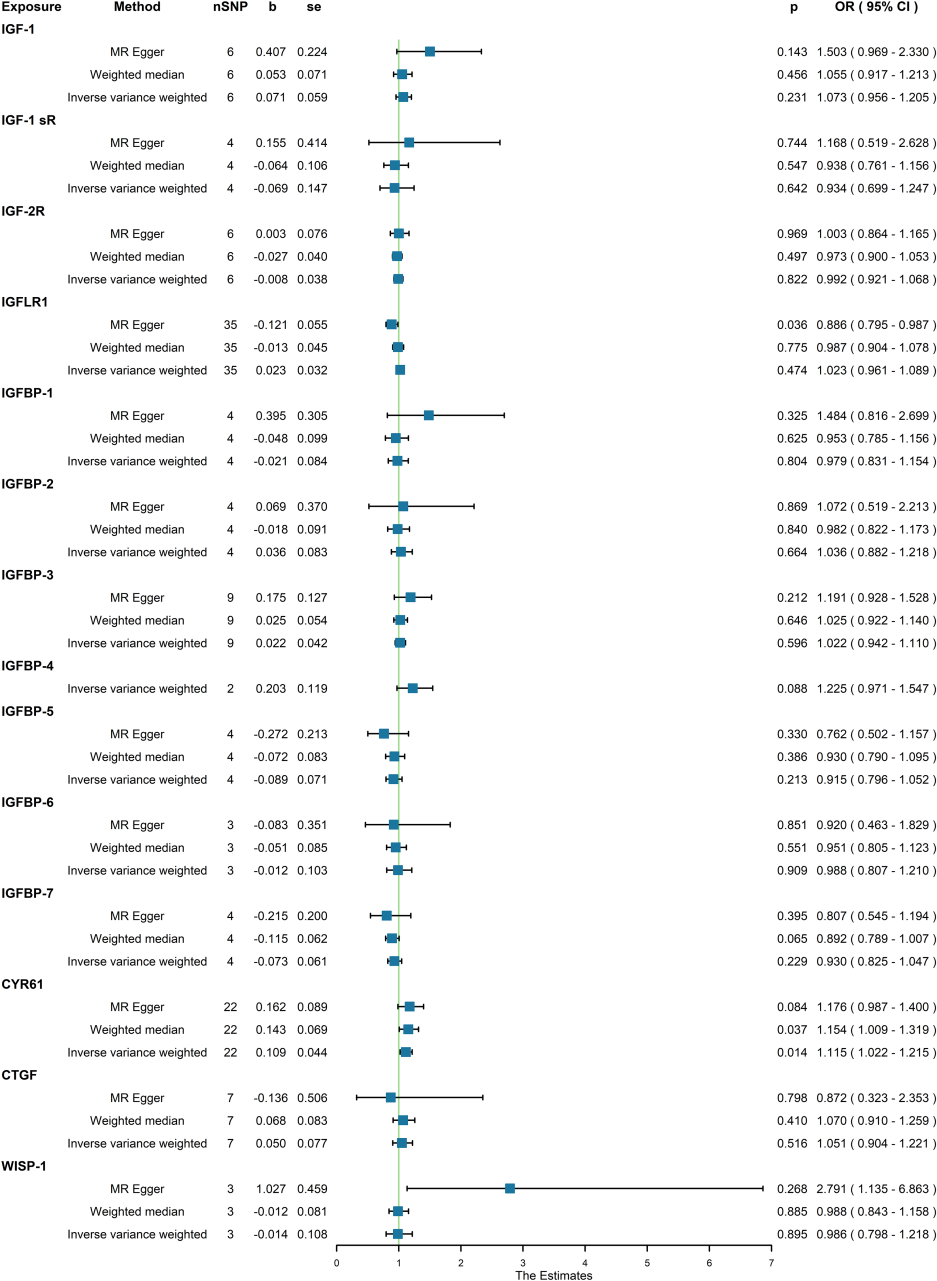
Association between genetical causes of IGF family members and osteoporosis from the FinnGen trait.

**Figure 4:**
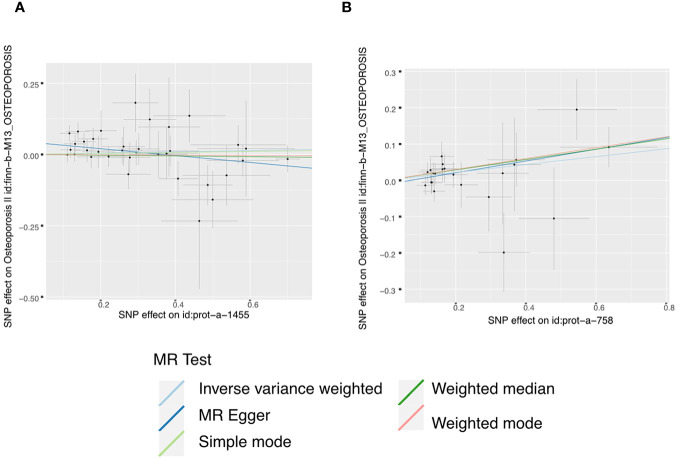
Scatter plots for MR analyses of the causal effect of IGFs on osteoporosis based on FinnGen trait. **(A)**, IGF-LR1. **(B)**, CYR61. Analyses were conducted using the conventional IVW, MBE, WMM, MR-Egger, and MR.RAPS methods. The slope of each line corresponding to the estimated MR effect per method.

Moreover, in our analysis using data from the FinnGen consortium, we also explored the associations of other IGF family members with osteoporosis. The IGF-LR1 level was negatively associated with osteoporosis according to the MR-Egger method (OR = 0.886, 95% CI = 0.795-0.987, P = 0.036) (**Figure 3**). On the other hand, CYR61 was linked to an increased risk of osteoporosis based on both the weighted median and IVW methods (OR = 1.154, 95% CI = 1.009-1.319, P = 0.037, and OR = 1.115, 95% CI = 1.022-1.215, P = 0.014, respectively) (**Figure 3**). We observed no heterogeneity of effects for IGF-LR1 (MR Egger, Cochran P value = 0.702; IVW, Cochran P value = 0.297) and CYR61 (MR Egger, Cochran P value = 0.709; IVW, Cochran P value = 0.735) when utilizing Cochran’s Q test. Additionally, no clear evidence of horizontal pleiotropy was found for CYR61 (intercept = –0.012, P = 0.499). However, we did detect possible pleiotropy for IGF-LR1 (intercept = 0.044, P = 0.004). The results remained robust as indicated by the leave-one-out analysis. Scatter plots illustrating the MR analyses of the causal effects of IGFs on osteoporosis with statistical significance are presented in **Figure 4** (A for IGF-LR1, and B for CYR61, respectively). MR Egger regression tests suggested no significant horizontal pleiotropy in this part. All the involved funnel plots, scatter plots and “leave-one out analysis” plots in assessing the association between IGFs family and osteoporosis in FinnGen trait were shown in **Supplementary file 3**.

## Discussion

This MR study conducted a comprehensive assessment of the causal relationship between genetically predicted IGF system members and osteoporosis, utilizing extensive GWAS summary statistics. Our findings indicate that genetically proxied higher serum levels of IGF-1, IGFBP-3, IGF-LR1, and CTGF, as well as lower levels of CYR61, are associated with a decreased risk of osteoporosis. However, it’s worth noting that these results did not consistently withstand all sensitivity analyses.

IGF-1, which reaches its peak during puberty, a critical period for acquiring peak bone mass (33), has been extensively studied in relation to bone strength and, consequently, osteoporosis. In our analysis using data from the UK trait, IGF-1 level is associated with a reduced risk of osteoporosis, as indicated by the weighted median method (OR=0.998, 95% CI = 0.997-1.000, P = 0.032). While analyzing data from the FinnGen consortium, the OR value of IGF-1 failed to reach a significant association between IGF-1 and osteoporosis with all p value above 0.05. Although the result from UK trait demonstrated the IGF-1 level is associated with reduced risk, but the 95%CI is near 1.00. Thus, we considered there is only some mild difference between the two traits, which indicates the causal effects of IGF-1 in reducing the prevalence of osteoporosis held population or ethic specificity. Also, we consider the differences in interpreting the results of IGF-1and osteoporosis might be related with the sample sizes and population internal differences of the two datasets, which may include some potential confounding factors, including dietary, living environment, and other genetic background. Observational studies have consistently shown that lower-to-normal circulating levels of IGF-1 correlate with an increased risk of fractures in older individuals. Notably, research involving a sizable cohort of 425 women and 257 men (aged 72–94 years) from the Framingham Heart Study provided compelling evidence that IGF-1 levels are associated with higher BMD in older women, even after adjusting for various confounding variables, including weight, height, protein intake, smoking, mobility, weight changes, and BMI (17). Similarly, a separate cohort of 2,902 older men also identified low serum IGF-1 levels as a potential risk factor for fractures in older individuals (18). This association mirrors the situation observed in men with idiopathic osteoporosis (IOM), who develop osteoporosis at a young age, further highlighting the potential pathogenic role of IGF-1 in the development of low bone mass (34). Previous whole-genome scan studies in human populations have also suggested a link between the IGF-1 gene and BMD (35, 36). Furthermore, a recent MR study aligns with the notion that IGF-1 plays a role in fracture prevention (37). In our own findings, we observed that a higher circulating level of IGF-1 is associated with a decreased risk of osteoporosis (OR = 0.998, 95% CI = 0.997–1.000, P = 0.032), in line with previous research indicating that elevated IGF-1 levels may serve as a protective factor against osteoporosis.

IGFBP-3 represents another member of the IGF system that exhibited a negative association with the risk of osteoporosis (OR = 0.999, 95% CI = 0.998–1.000, P = 0.019) in UK biobank. While the results from FinnGen dataset did not find a clear association between IGFBP-3 and osteoporosis. Notably, IGFBP-3 is a prominent component of the circulating IGF complex (38). Much like other IGF binding proteins, it plays a role in modulating skeletal growth and metabolism by regulating the access of IGFs to their receptors (14). *In vitro* studies have shown that IGFBP-3 can either inhibit or stimulate IGF activity, the latter by enhancing the delivery of IGF-1 to cell surface receptors (38). Consistent with our findings, existing evidence suggests that IGFBP-3 may have a positive impact on bone formation. It has been demonstrated to participate in the storage of IGFs within the skeletal matrix by binding to the type I collagen molecule (39). Additionally, individuals with growth hormone deficiency who exhibit low serum levels of IGF-1 and IGFBP-3 tend to have reduced BMD and a significantly higher risk of osteoporotic fractures (40). Furthermore, concurrent administration of IGF-1 and IGFBP-3 has been shown to stimulate bone growth (41). Circulating IGF-LR1 was also identified as having a negative association with the risk of osteoporosis (OR = 0.886, 95% CI = 0.795–0.987, P = 0.036) in FinnGen consortium. IGF-LR1 is a gene that encodes a protein and is situated on chromosome 19. It exhibits widespread expression in organs such as lymph nodes, spleen, and kidney (42). While our literature review indicated limited observational studies on the relationship between IGF-LR1 and osteoporosis, it suggests the need for further investigation in this area. And the molecular function of IGF-LR1 presented population specificity as controversial results identified between UK and FinnGen biobanks.

Similarly, CTGF/CCN2 displayed an inverse association with osteoporosis (OR = 0.998, 95% CI = 0.996–0.999, P = 0.004) in UK biobank, which failed to demonstrate consistent result in FinnGen dataset. CTGF, which is expressed and secreted by osteoblasts during their proliferation, differentiation, bone formation, and fracture healing processes, has been shown to play a regulatory role in osteogenesis within osteoblasts (43, 44). For instance, Kawaki and colleagues reported that the knockout of CTGF in osteoblasts led to delayed osteoblast maturation and mineralization in cultured osteoblasts (45). These findings, in conjunction with our results, suggest that CTGF may serve as a protective factor against osteoporosis. Conversely, cysteine-rich protein 61 (CYR61/CCN1) was the sole member that we identified as being associated with an increased risk of osteoporosis (OR = 1.115, 95% CI = 1.022–1.215, P = 0.014). However, it’s noteworthy that most basic research (46–48) has reported CYR61 to have a bone-stimulating effect, which appears to contrast with our findings. Therefore, future studies of higher quality investigating the relationship between CYR61 and osteoporosis may be warranted to reconcile these seemingly contradictory results.

This analysis possesses several notable strengths, primarily stemming from the MR design, which effectively mitigated bias associated with residual confounding, thereby bolstering the causal inference. Furthermore, the study benefits from the extensive availability of GWAS data on IGF system members and osteoporosis, rendering it a well-powered investigation for elucidating the observational relationship between IGF system members and osteoporosis. However, it is crucial to acknowledge certain limitations within these analyses. Firstly, a higher significance threshold of P < 1.00E-5 was employed for the selection of SNPs from the GWAS datasets on IGF family members. This decision was necessitated by the limited number of members with at least one genome-wide significant SNP when using P < 5.00E-8 as the threshold. Additionally, 13 of these members exhibited more than two genome-wide significant SNPs when P < 1.00E-5. Secondly, the precise functionality of the ultimately selected SNPs remains unclear. This lack of clarity implies that these genetic variants have not been definitively established as biologically linked to the exposure (IGF family members), introducing a potential source of bias in causal estimates. Also, the GWAS summary datasets for osteoporosis were derived from two published studies that did not account for specific factors such as age, sex, and BMI. The presence of population stratification and imbalances in age and gender between the two GWASs may introduce additional sources of bias. Moreover, it’s important to note that the MR results offer insight into the direction of how concentrations of IGF system components may influence the risk of osteoporosis, but they do not provide a specific magnitude. This limitation yields a relatively broad causal inference.

## Conclusion

In conclusion, our MR study provides support for causal relationships between five IGF system members and the development of osteoporosis. Specifically, our findings indicate that lower levels of IGF-1, IGFBP-3, IGF-LR1, and CTGF, along with higher levels of CYR61, genetically contribute to an increased risk of osteoporosis. However, controversial results had been identified between different datasets indicating population specific causal effects between IGF family and osteoporosis. Although the results from both datasets demonstrated that IGF family involved in the pathogenesis of osteoporosis, but the responding key molecules might be various among different population. Further research is warranted to elucidate the underlying mechanisms governing these associations. Essentially, our results suggest that IGF system members, particularly IGF-1, may serve as potential predictive markers during early growth stages and represent promising therapeutic candidates for addressing osteoporosis in validated regional individuals.

## Data availability statement

The original contributions presented in the study are included in the article/**Supplementary Material**. Further inquiries can be directed to the corresponding authors.

## Author contributions

YL: Conceptualization, Data curation, Funding acquisition, Investigation, Methodology, Project administration, Supervision, Validation, Writing – review & editing. JT: Conceptualization, Formal Analysis, Investigation, Methodology, Software, Validation, Writing – original draft. CZ: Data curation, Formal Analysis, Investigation, Methodology, Writing – original draft. SL: Investigation, Methodology, Software, Writing – original draft. XL: Formal Analysis, Investigation, Methodology, Writing – original draft. BZ: Conceptualization, Investigation, Project administration, Supervision, Validation, Writing – review & editing.

## Supplementary material

Supplementary Table 1 The results of F-statistics and P values for included SNPs.

Supplementary file 1 The codes used for R software in the research.

Supplementary file 2 Funnel plots and Leave-one-out sensitive analysis of IGFs on osteoporosis based on UK trait.

Supplementary file 3 Funnel plots and Leave-one-out sensitive analysis of IGFs on osteoporosis based on FinnGen trait.
