# Supplementary material for: Controversial causal association between IGF family members and osteoporosis: a Mendelian randomization study between UK and FinnGen biobanks
Source: Front Endocrinol (Lausanne). 2024 Jan 8;14:1332803. doi: 10.3389/fendo.2023.1332803 (PMC10801076; doi:10.3389/fendo.2023.1332803)
Supplement: Supplementary file 1 — The codes used for R software in the research. [file DataSheet_1.pdf]

```
library(TwoSampleMR)
library(dplyr)
explist <- read.table('exp.txt',stringsAsFactors = F,
                    header = F,quote = '',sep = ',')
outLIST <- read.csv('out.txt',sep = ',',header = F)
explist <- explist$V1
outLIST <- outLIST$V1

for (i in outLIST) {
  dir.create(path = i)
  setwd(i)
  pdf(file = 'IGFs_on_OP.pdf',width = 10,height = 10)
  for (n in explist) {
    exp <-extract_instruments(outcomes=n,
                           clump=TRUE, r2=0.01, p1=1e-5,
                           kb=5000,access_token = NULL)
    out <- extract_outcome_data(snps = exp$SNP,
                             outcomes=i,
                             proxies = FALSE,
                             maf_threshold = 0.01,
                             access_token = NULL)
    mydata <- harmonise_data(exposure_dat=exp,outcome_dat=out, action= 2)
    res <- mr(mydata)
    write.csv(res,file = paste('OP_',n,'_res.txt'))
    ordata <- generate_odds_ratios(res)
    write.csv(ordata,file = paste('OP_',n,'.csv'))
    het <- mr_heterogeneity(mydata)
    write.csv(het,file = paste('OP_',n,'_het.txt'))
    mr(mydata,method_list=c('mr_ivw_mre'))
    pleio <- mr_pleiotropy_test(mydata)
    write.csv(pleio,file = paste('OP_',n,'_pleio.txt'))
    single <- mr_leaveoneout(mydata)
    write.csv(single,file = paste('OP_',n,'_single.txt'))
    p1 <- mr_leaveoneout_plot(single)
    print(p1)
    p2 <- mr_scatter_plot(res,mydata)
    print(p2)
    res_single <- mr_singlesnp(mydata)
    p3 <-mr_forest_plot(res_single)
    print(p3)
    p4 <- mr_funnel_plot(res_single)
    print(p4)
  }
  dev.off()
}

#For F value caculation
```

```
for (n in c(1:length(explist))) {
  exp <-extract_instruments(outcomes=explist[n],
                            clump=TRUE, r2=0.01, p1=1e-5,
                            kb=5000,access_token = NULL)
  exp <-  mutate(exp,R=get_r_from_bsen(exp$beta.exposure,
                                       exp$se.exposure,
                                       exp$samplesize.exposure))
  exp<-  mutate(exp,
                F=(samplesize.exposure-2)*((R*R)/(1-R*R)))
  write.csv(exp,file = paste(explist[n],'exp.csv'))
  }
```
