# Supplementary material for: Controversial causal association between IGF family members and osteoporosis: a Mendelian randomization study between UK and FinnGen biobanks
Source: Front Endocrinol (Lausanne). 2024 Jan 8;14:1332803. doi: 10.3389/fendo.2023.1332803 (PMC10801076; doi:10.3389/fendo.2023.1332803)
Supplement: Supplementary file 2 — Funnel plots and Leave-one-out sensitive analysis of IGFs on osteoporosis based on UK trait. [file DataSheet_2.pdf]

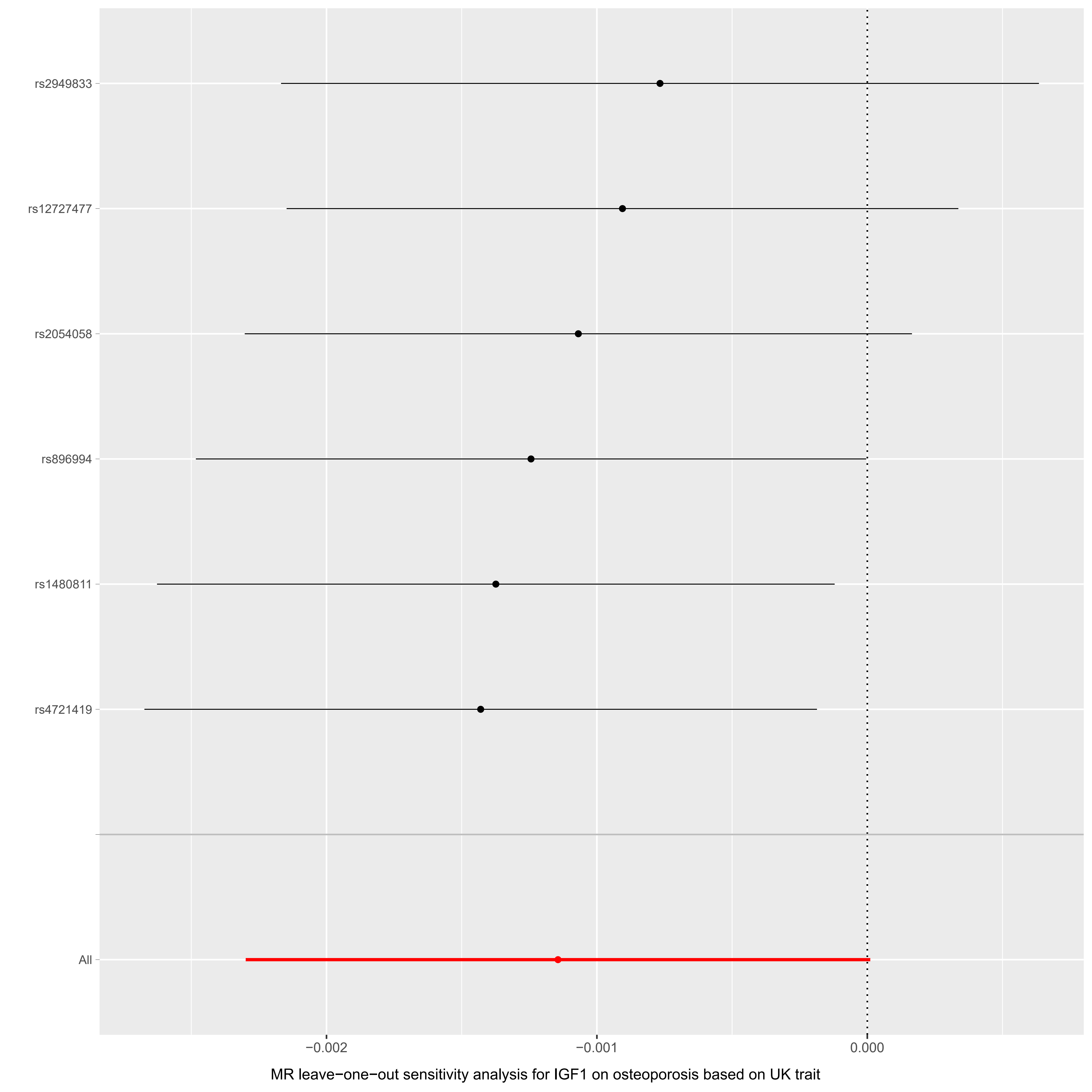
rs2949833
rs12727477
rs2054058
rs896994
rs1480811
rs4721419
All
−0.002
−0.001
0.000
MR leave−one−out sensitivity analysis for IGF1 on osteoporosis based on UK trait

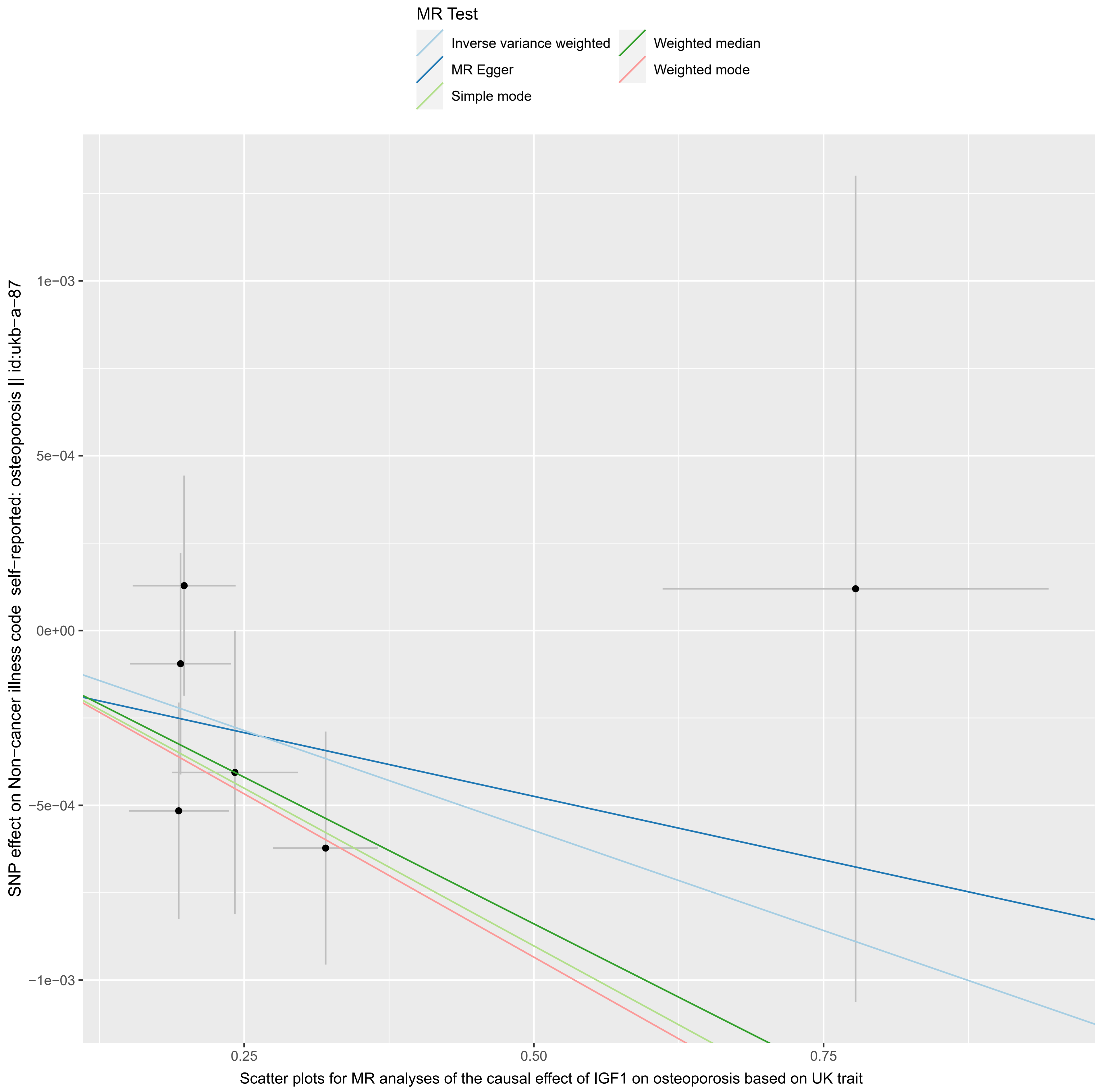

MR Test
Inverse variance weighted
MR Egger
Simple mode
Weighted median
Weighted mode
SNP effect on Non−cancer illness code self−reported: osteoporosis || id:ukb−a−87
1e−03
5e−04
0e+00
−5e−04
−1e−03
0.25
0.50
0.75
Scatter plots for MR analyses of the causal effect of IGF1 on osteoporosis based on UK trait

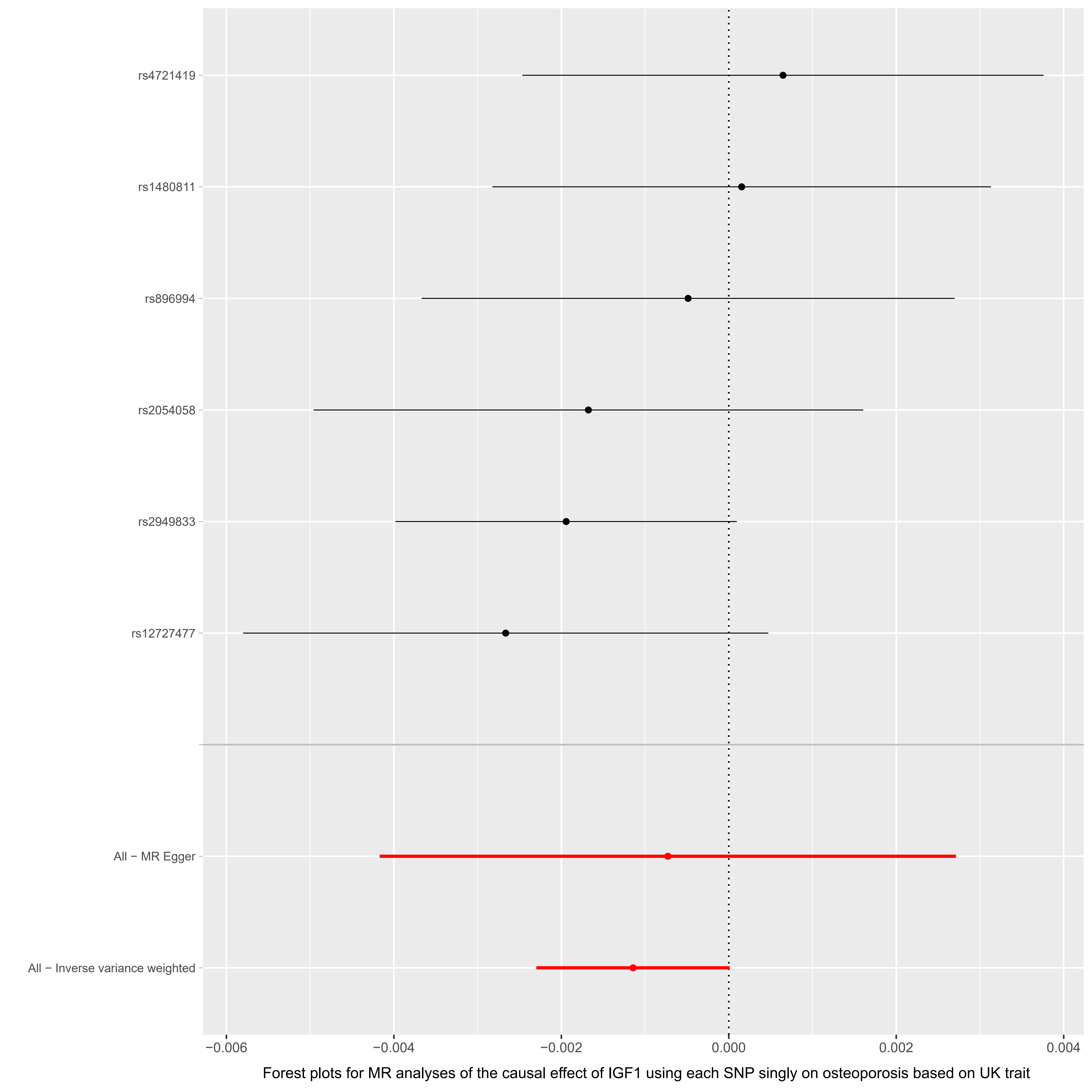
rs4721419
rs1480811
rs896994
rs2054058
rs2949833
rs12727477
All − MR Egger
All − Inverse variance weighted
−0.006
−0.004
−0.002
0.000
0.002
0.004
Forest plots for MR analyses of the causal effect of IGF1 using each SNP singly on osteoporosis based on UK trait

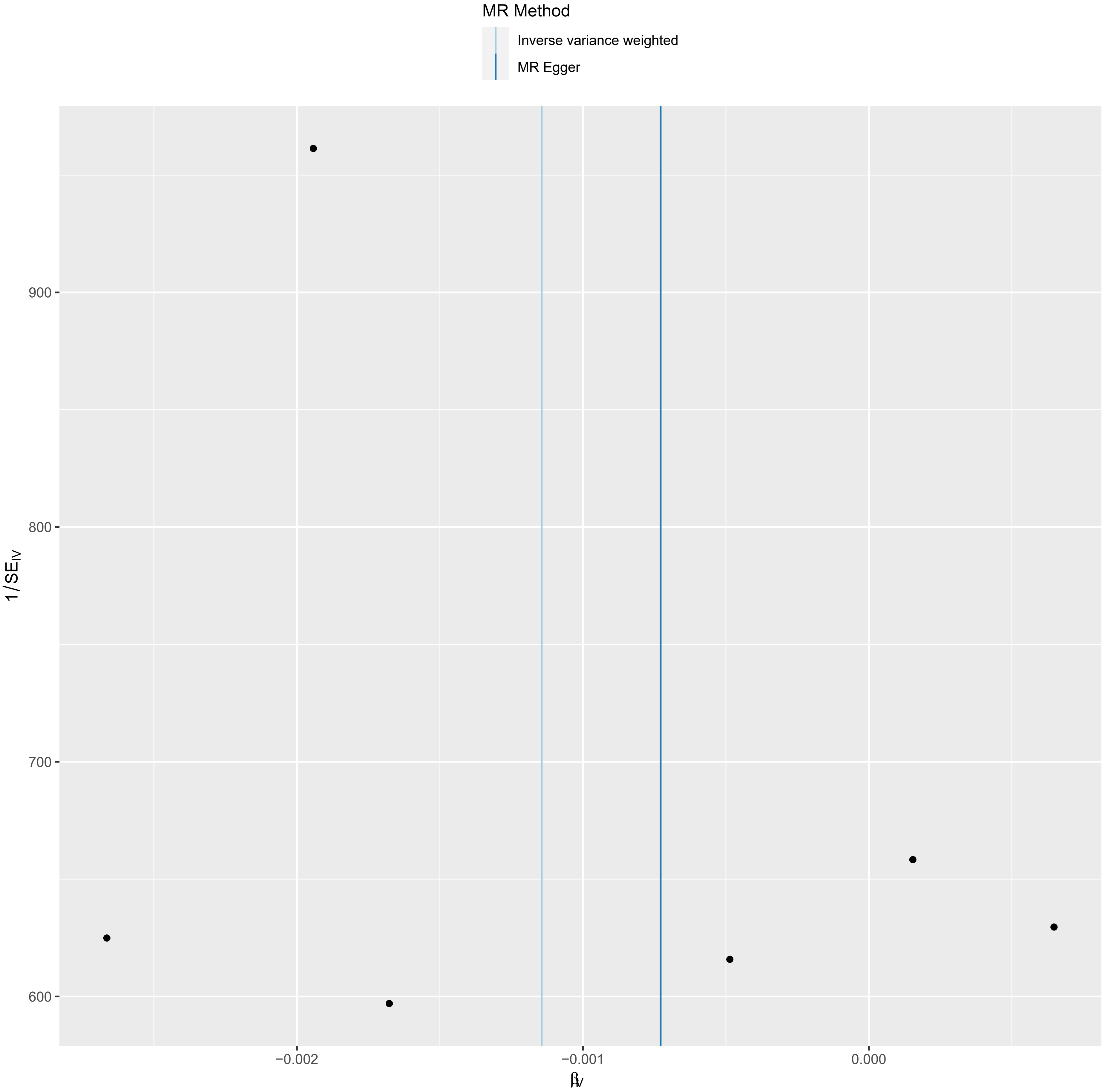

Funnal plots to assess heterogeneity for IGF1 using all SNPs with the MR Egger and IVW methods

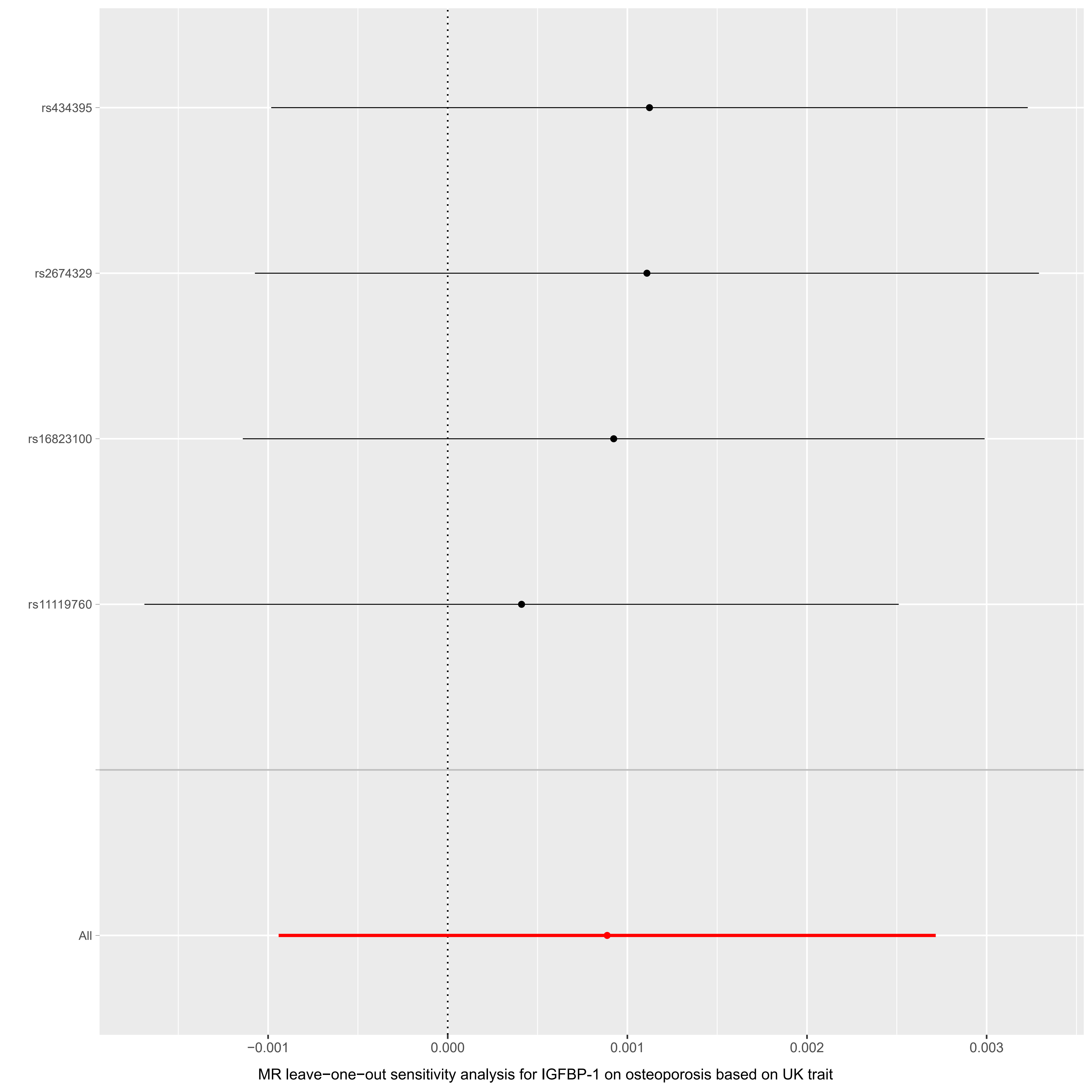

rs434395
rs2674329
rs16823100
rs11119760
All
−0.001
0.000
0.001
0.002
0.003
MR leave−one−out sensitivity analysis for IGFBP-1 on osteoporosis based on UK trait

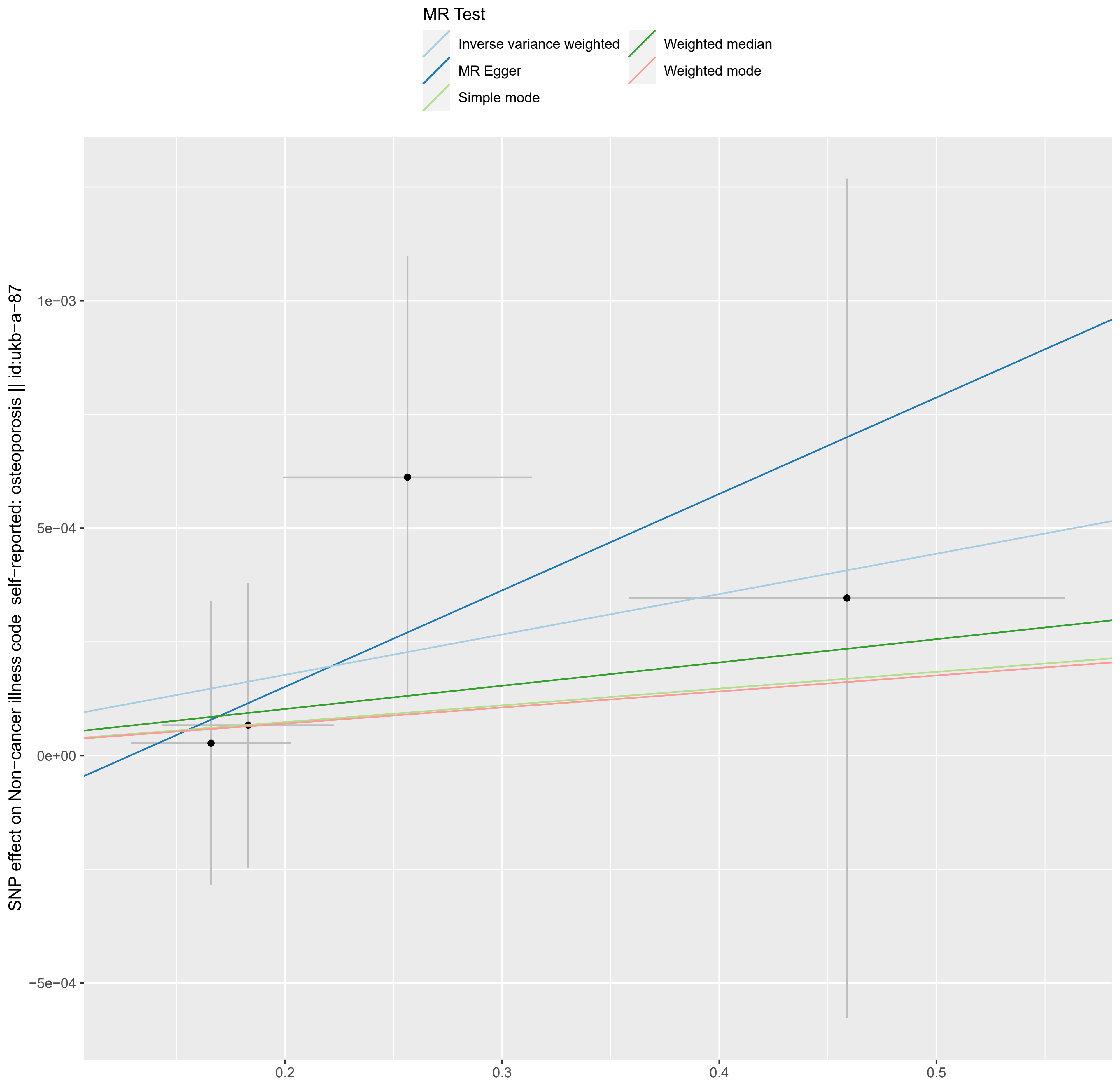

Scatter plots for MR analyses of the causal effect of IGFBP-1 on osteoporosis based on UK trait

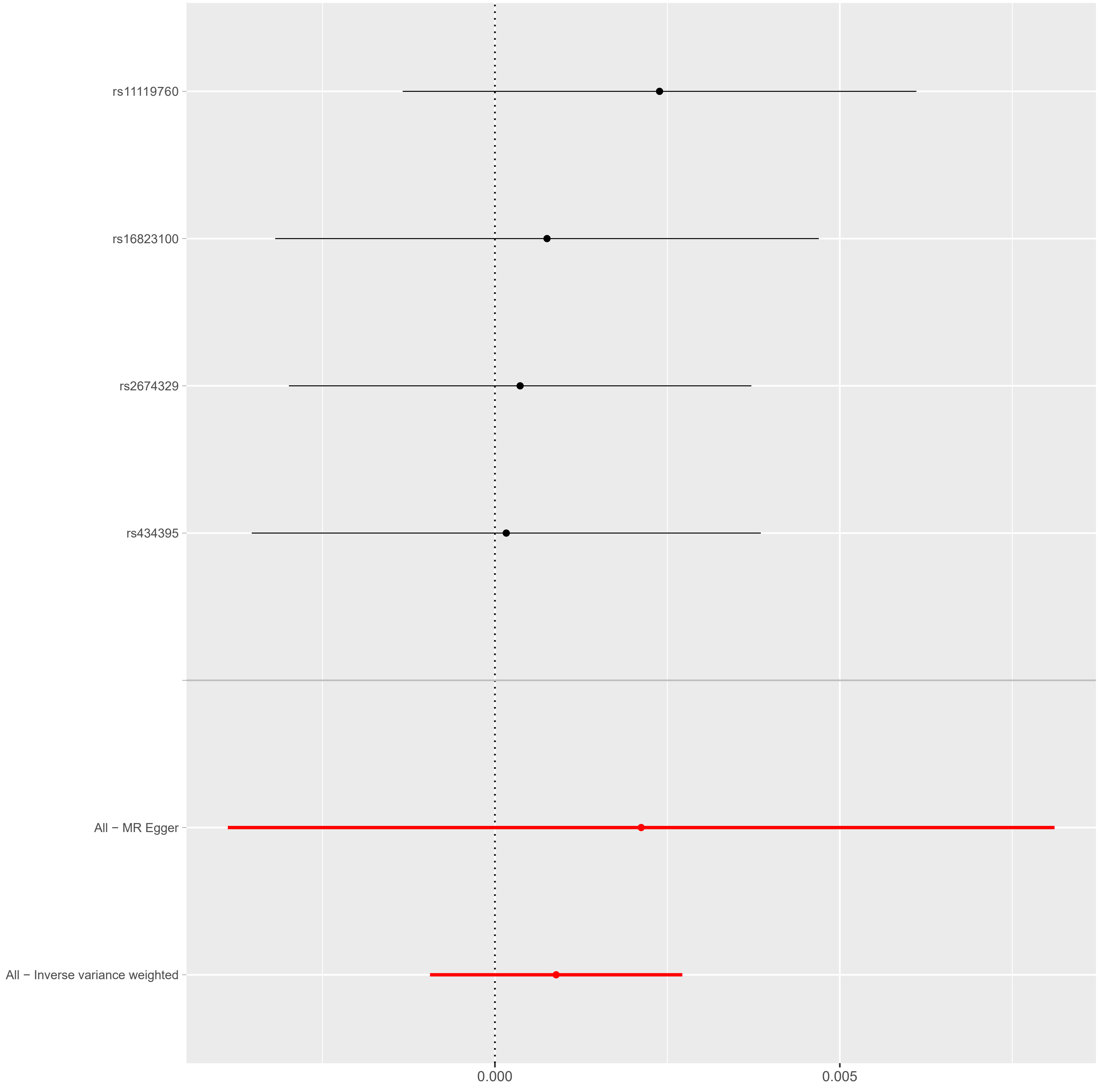

Forest plots for MR analyses of the causal effect of IGFBP-1 using each SNP singly on osteoporosis based on UK trait

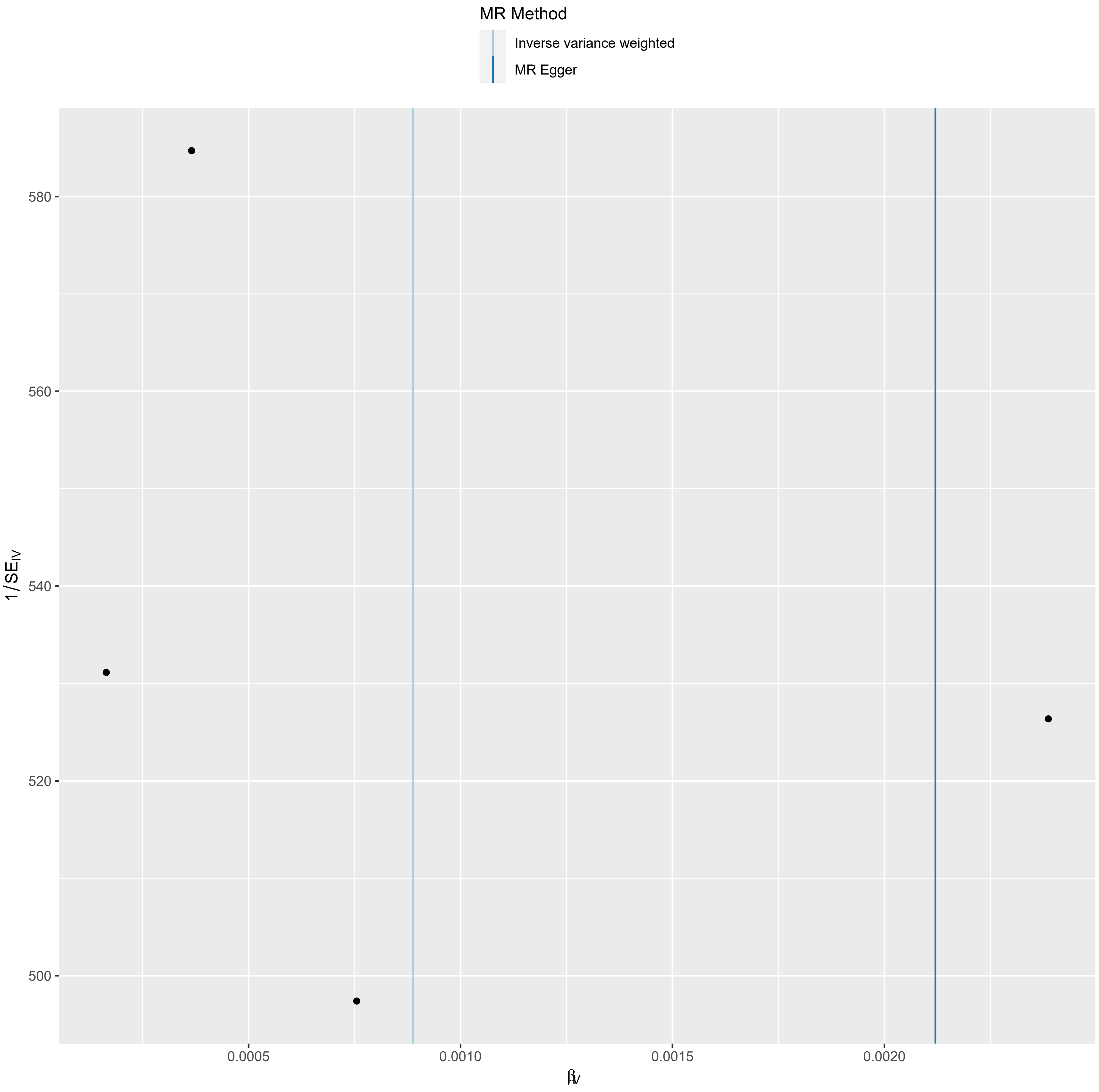

Funnal plots to assess heterogeneity for IGFBP-1 using all SNPs with the MR Egger and IVW methods

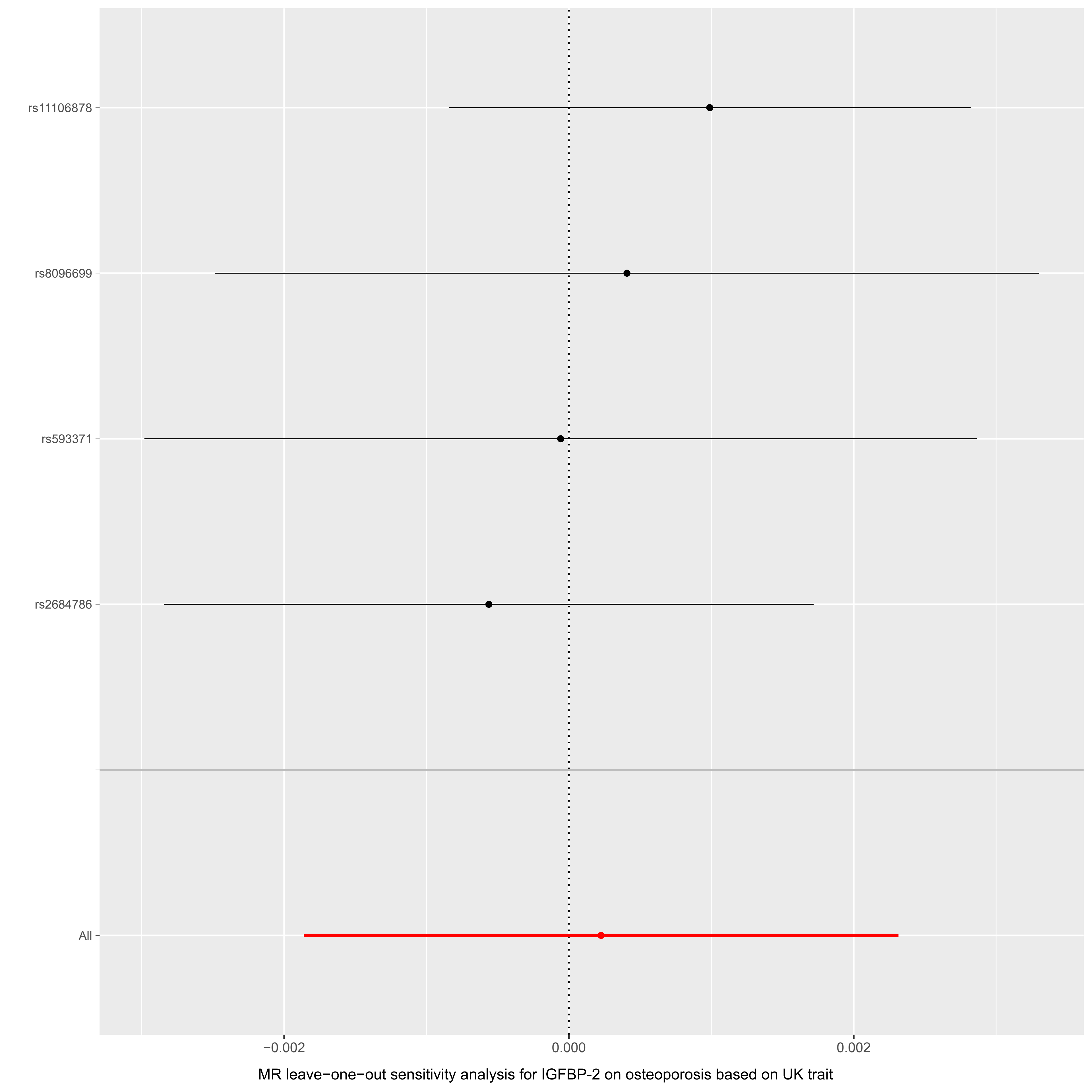

rs11106878
rs8096699
rs593371
rs2684786
All
−0.002
0.000
0.002
MR leave−one−out sensitivity analysis for IGFBP-2 on osteoporosis based on UK trait

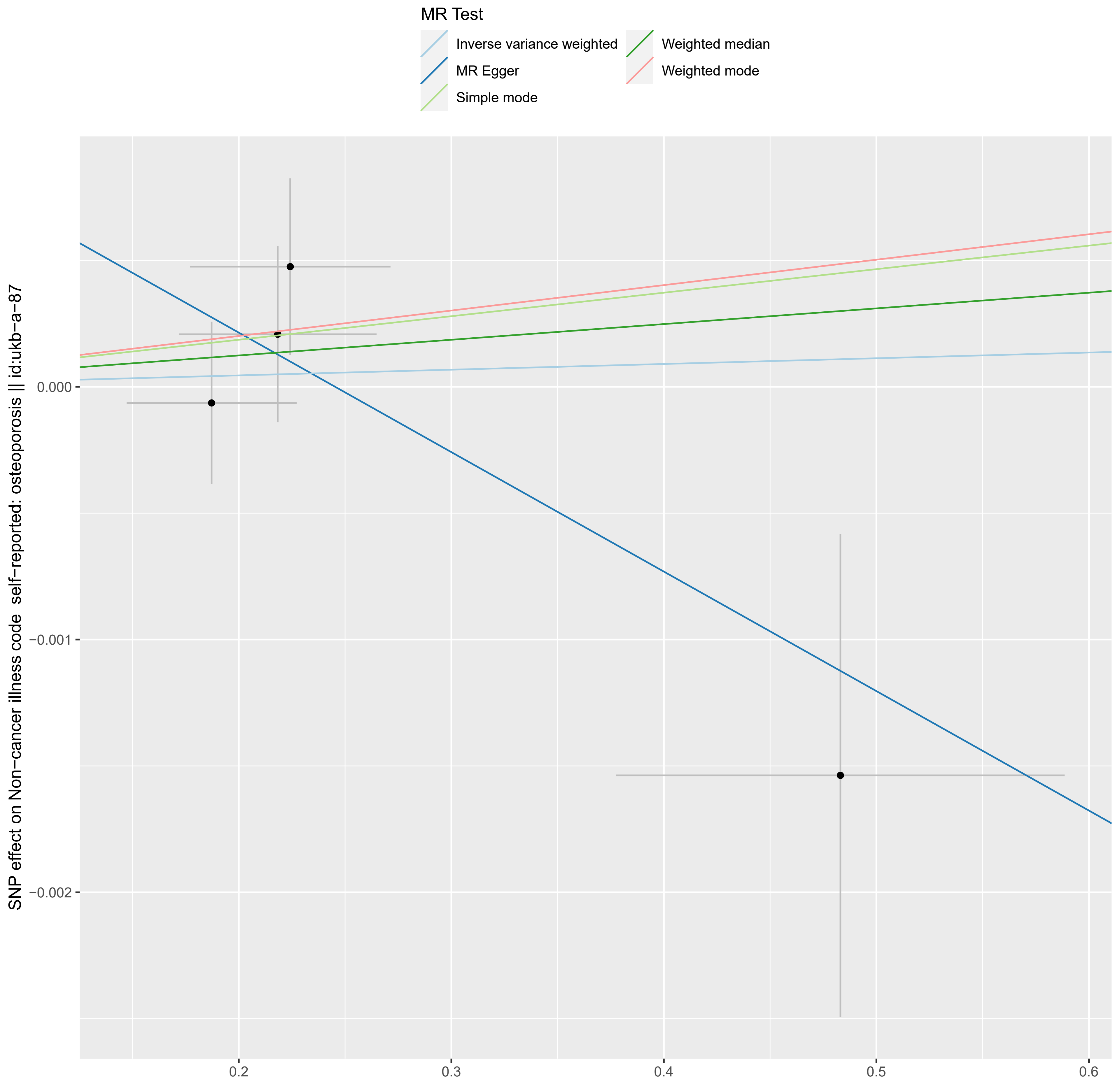

Scatter plots for MR analyses of the causal effect of IGFBP-2 on osteoporosis based on UK trait

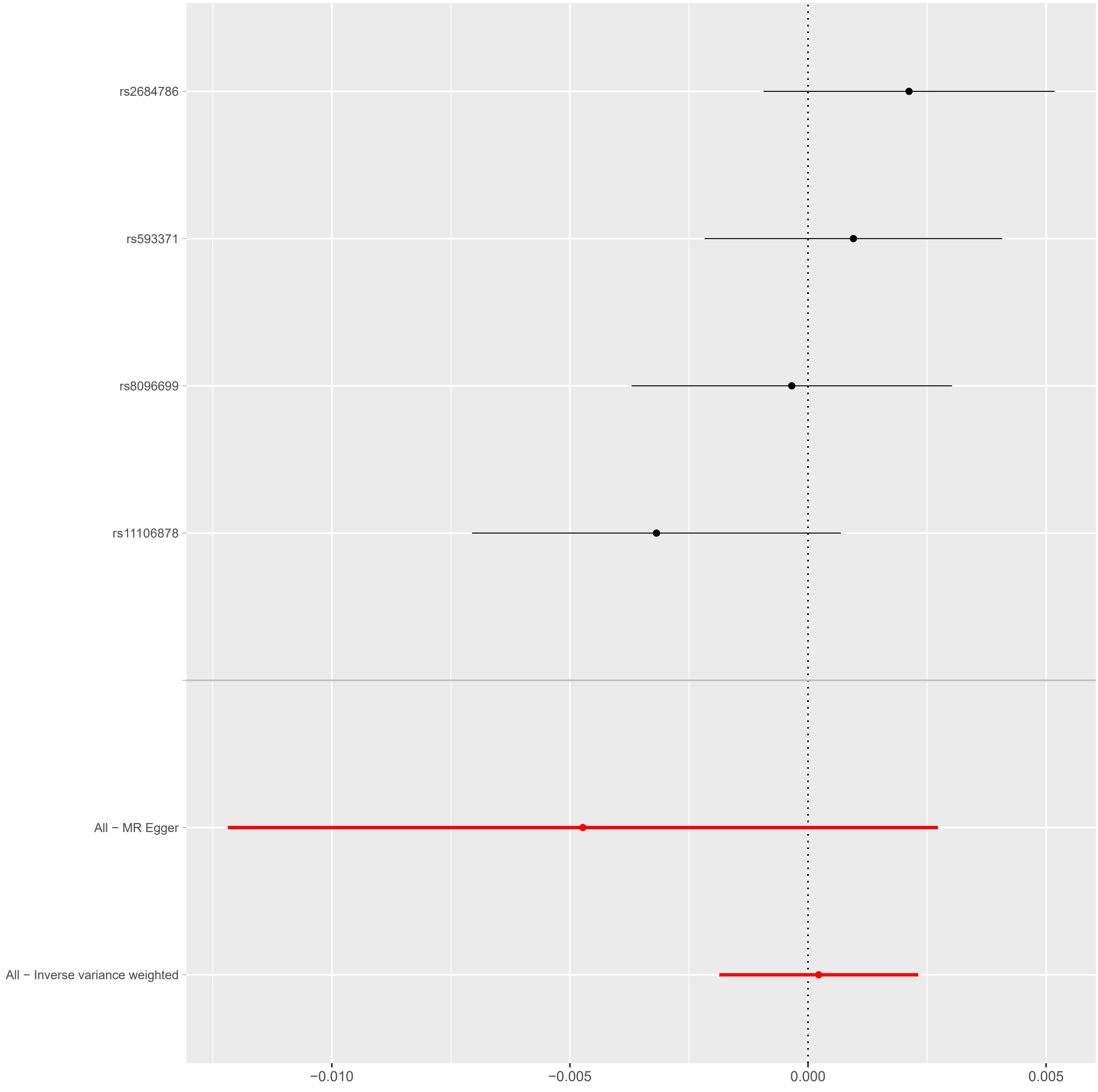

Forest plots for MR analyses of the causal effect of IGFBP-2 using each SNP singly on osteoporosis based on UK trait

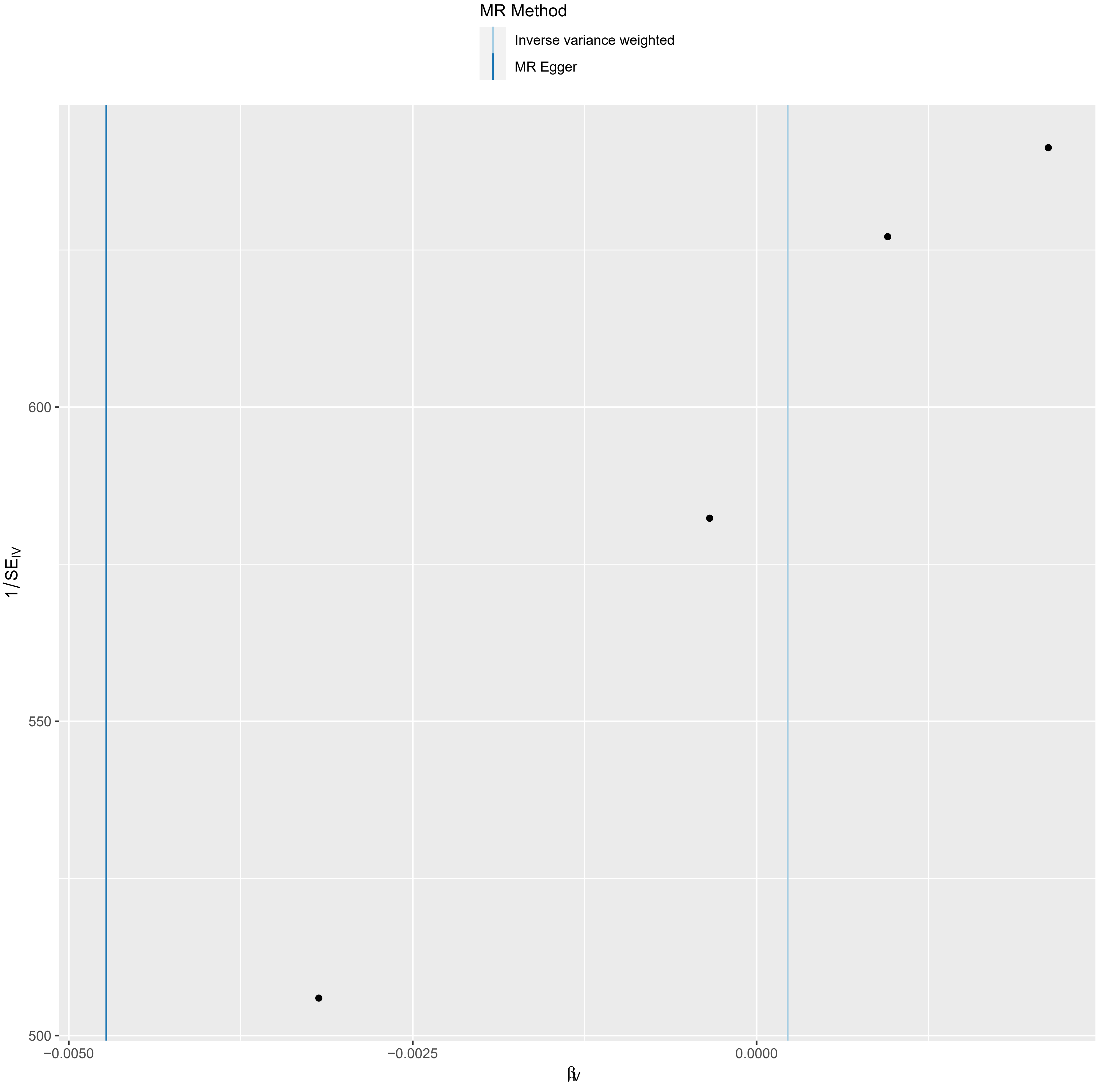

Funnal plots to assess heterogeneity for IGFBP-2 using all SNPs with the MR Egger and IVW methods

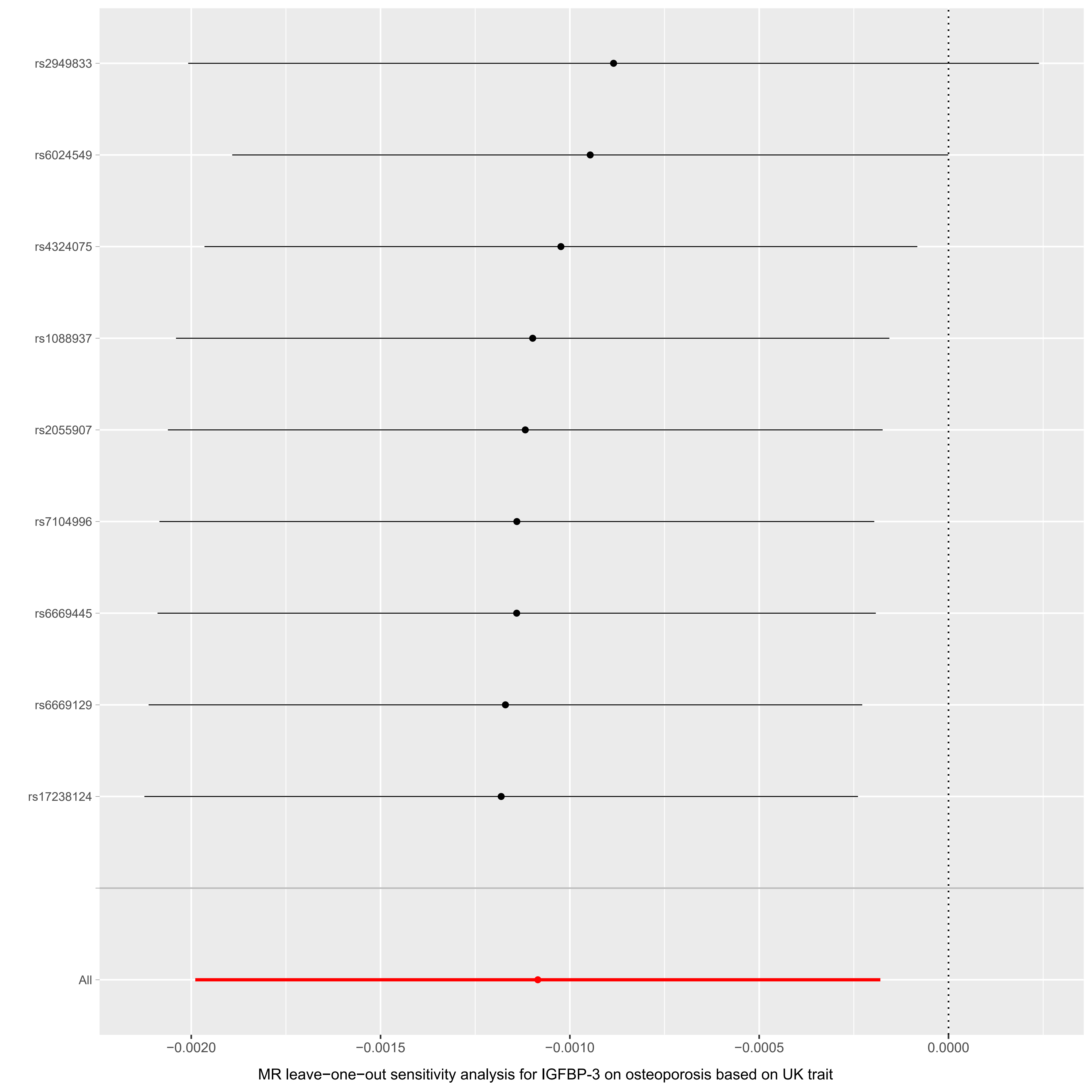
rs2949833
rs6024549
rs4324075
rs1088937
rs2055907
rs7104996
rs6669445
rs6669129
rs17238124
All
−0.0020
−0.0015
−0.0010
−0.0005
0.0000
MR leave−one−out sensitivity analysis for IGFBP-3 on osteoporosis based on UK trait

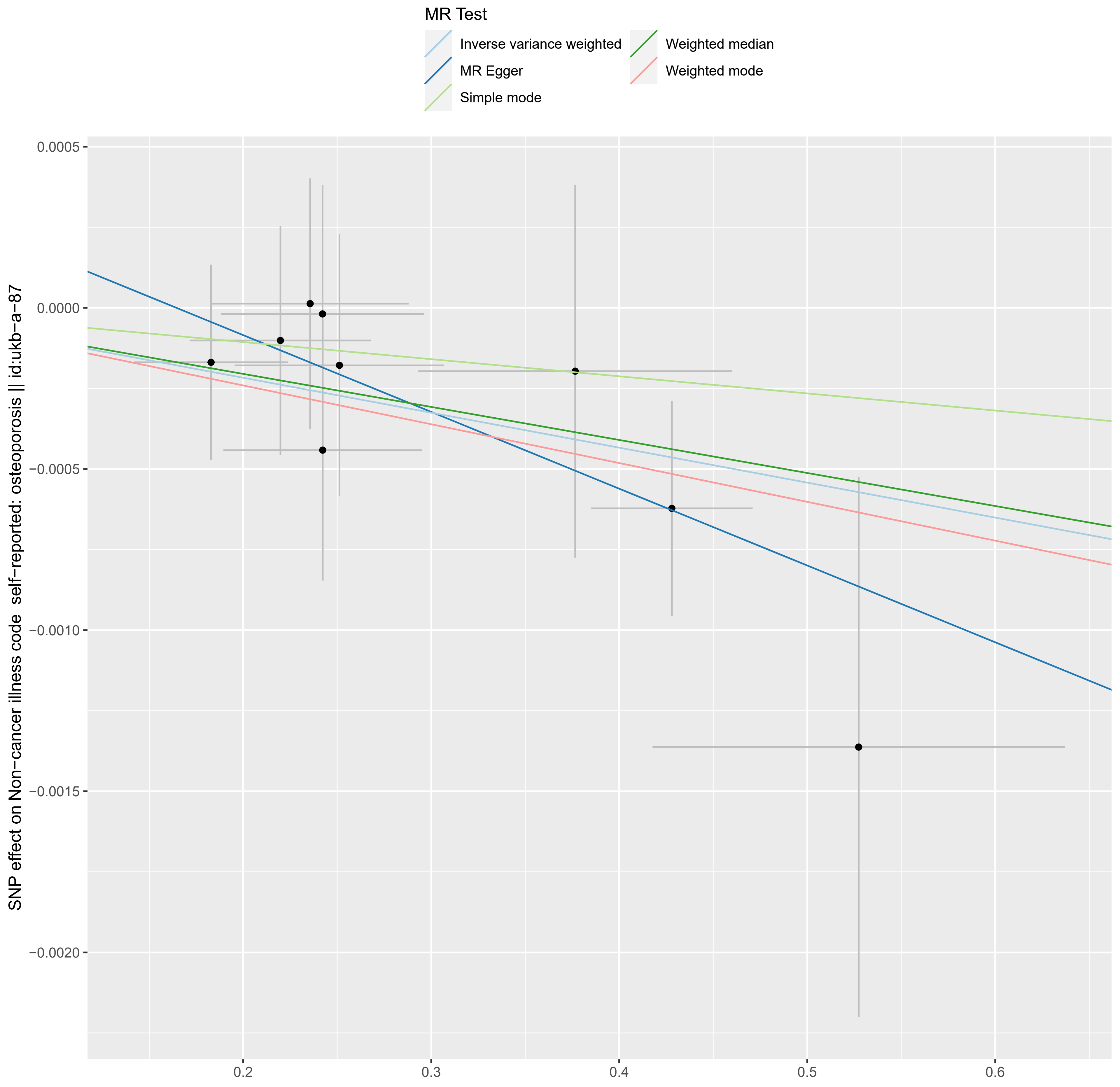

Scatter plots for MR analyses of the causal effect of IGFBP-3 on osteoporosis based on UK trait

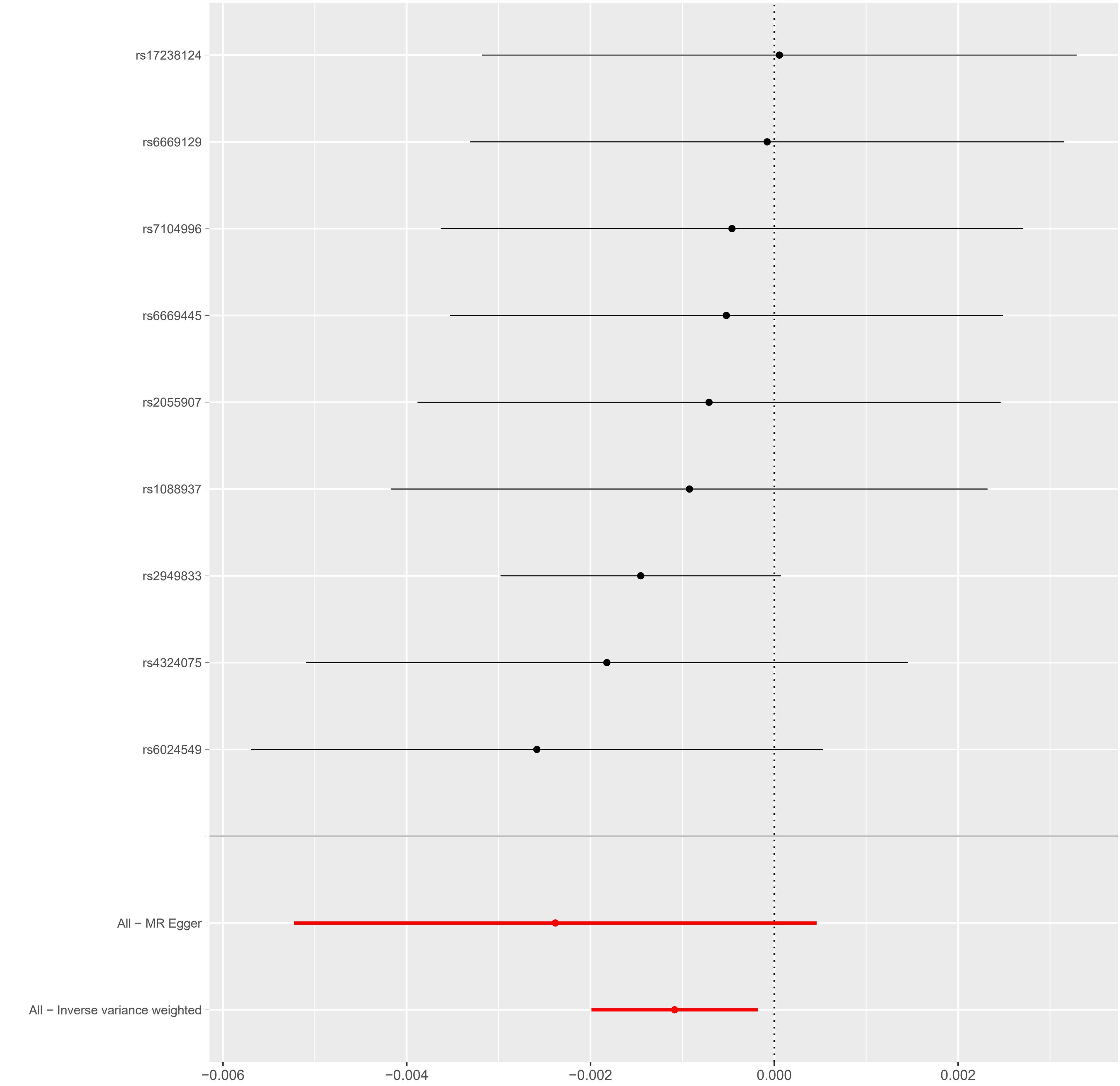

Forest plots for MR analyses of the causal effect of IGFBP-3 using each SNP singly on osteoporosis based on UK trait

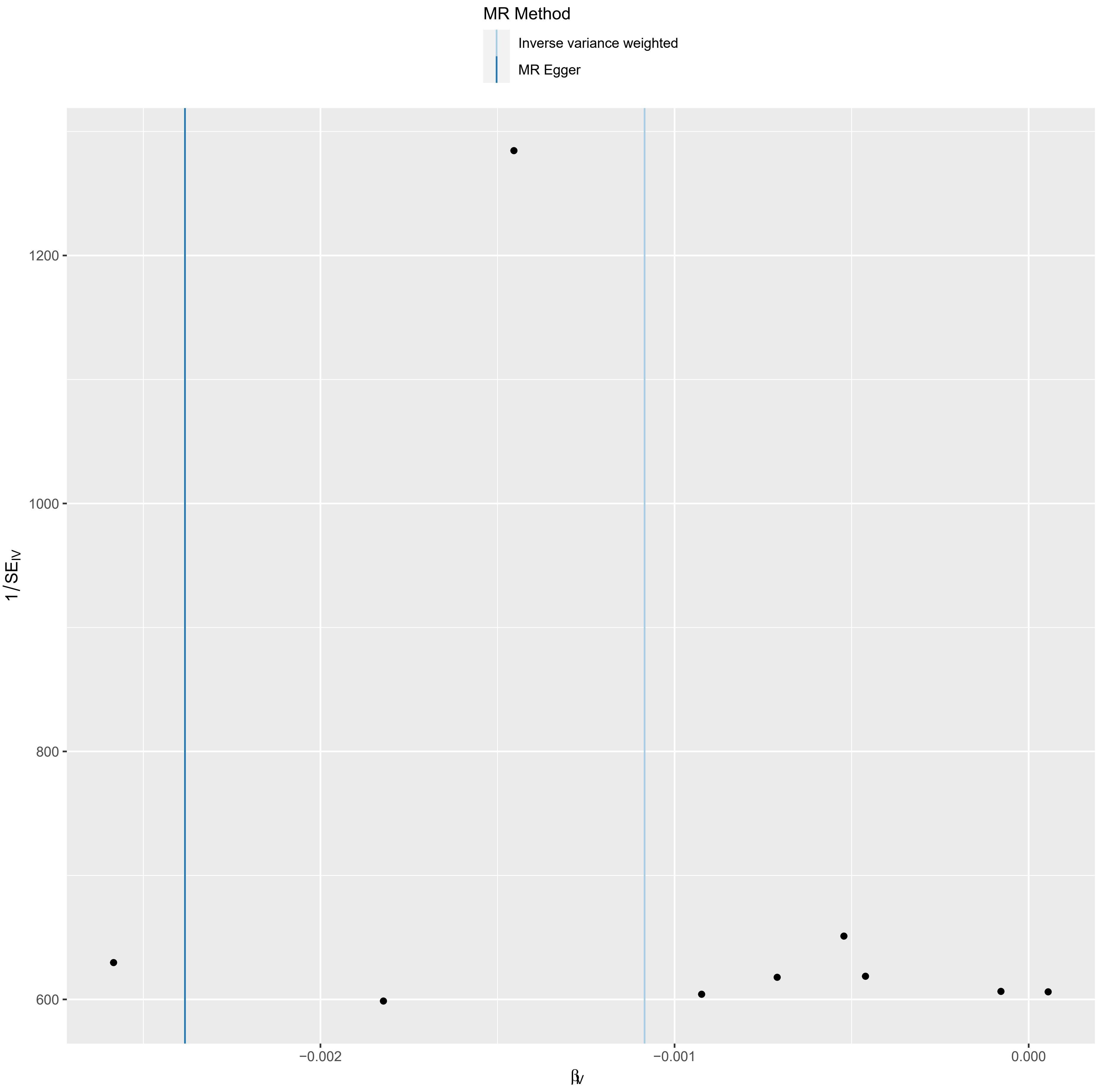

Funnal plots to assess heterogeneity for IGFBP-3 using all SNPs with the MR Egger and IVW methods

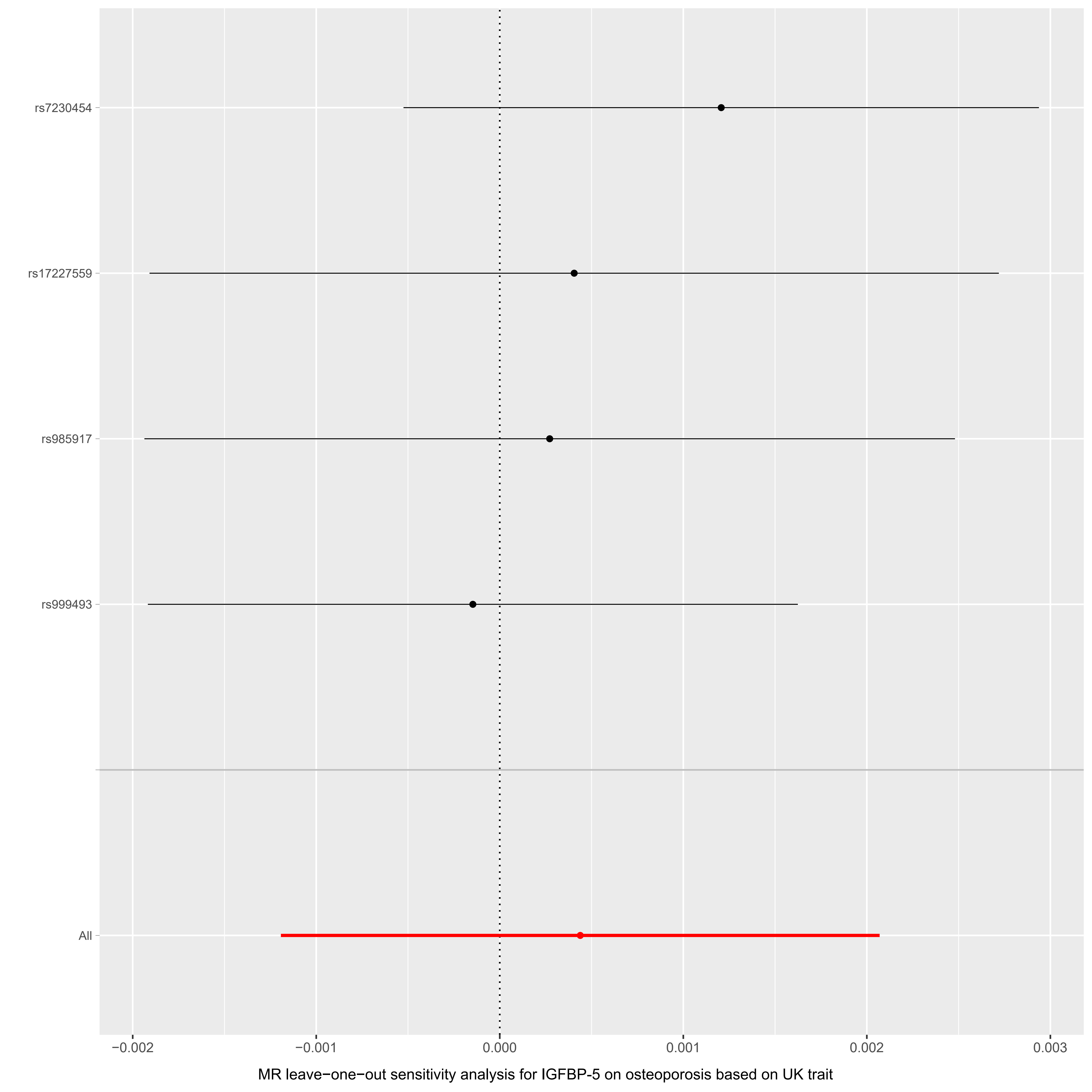

rs7230454
rs17227559
rs985917
rs999493
All
−0.002
−0.001
0.000
0.001
0.002
0.003
MR leave−one−out sensitivity analysis for IGFBP-5 on osteoporosis based on UK trait

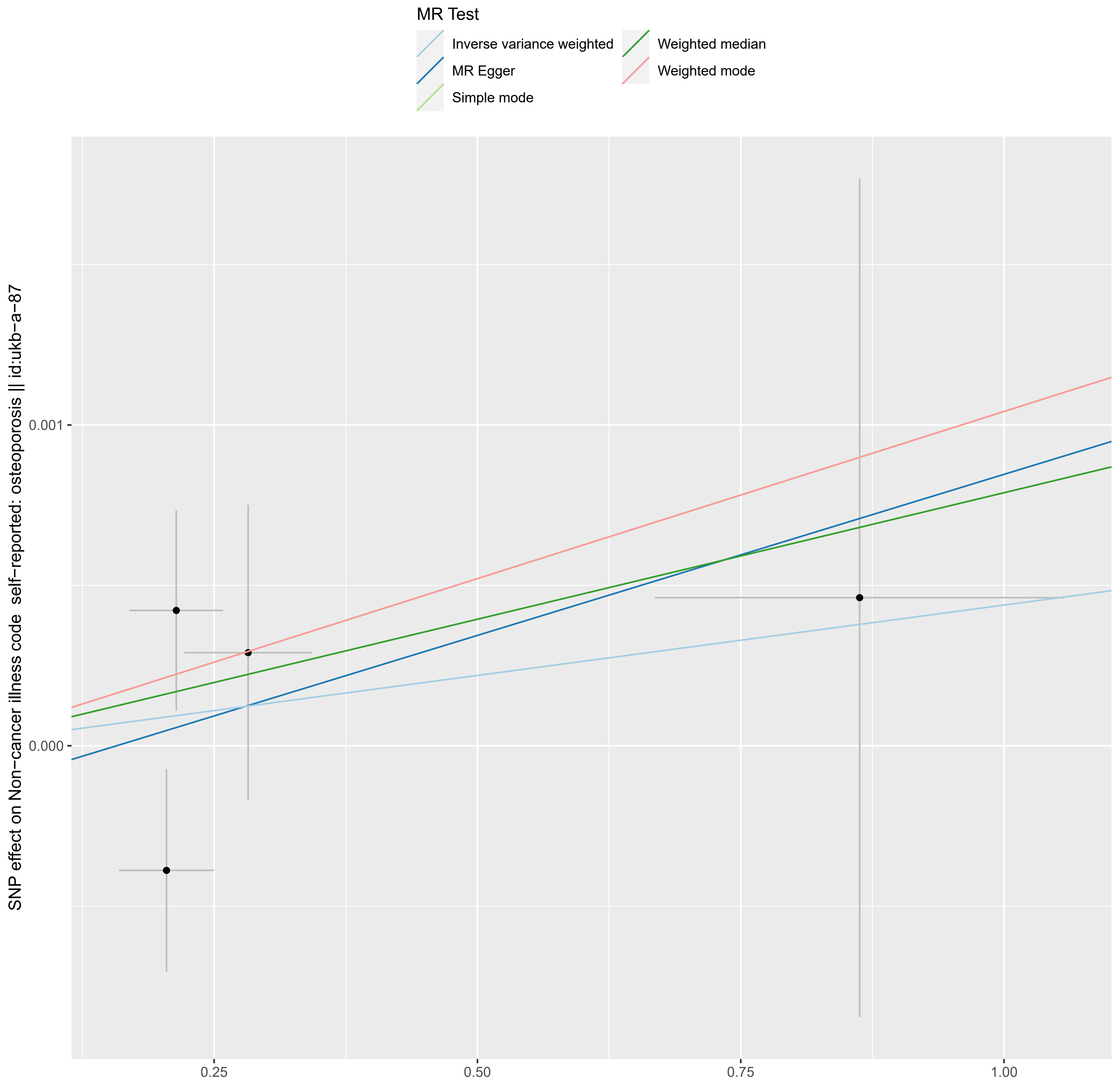

Scatter plots for MR analyses of the causal effect of IGFBP-5 on osteoporosis based on UK trait

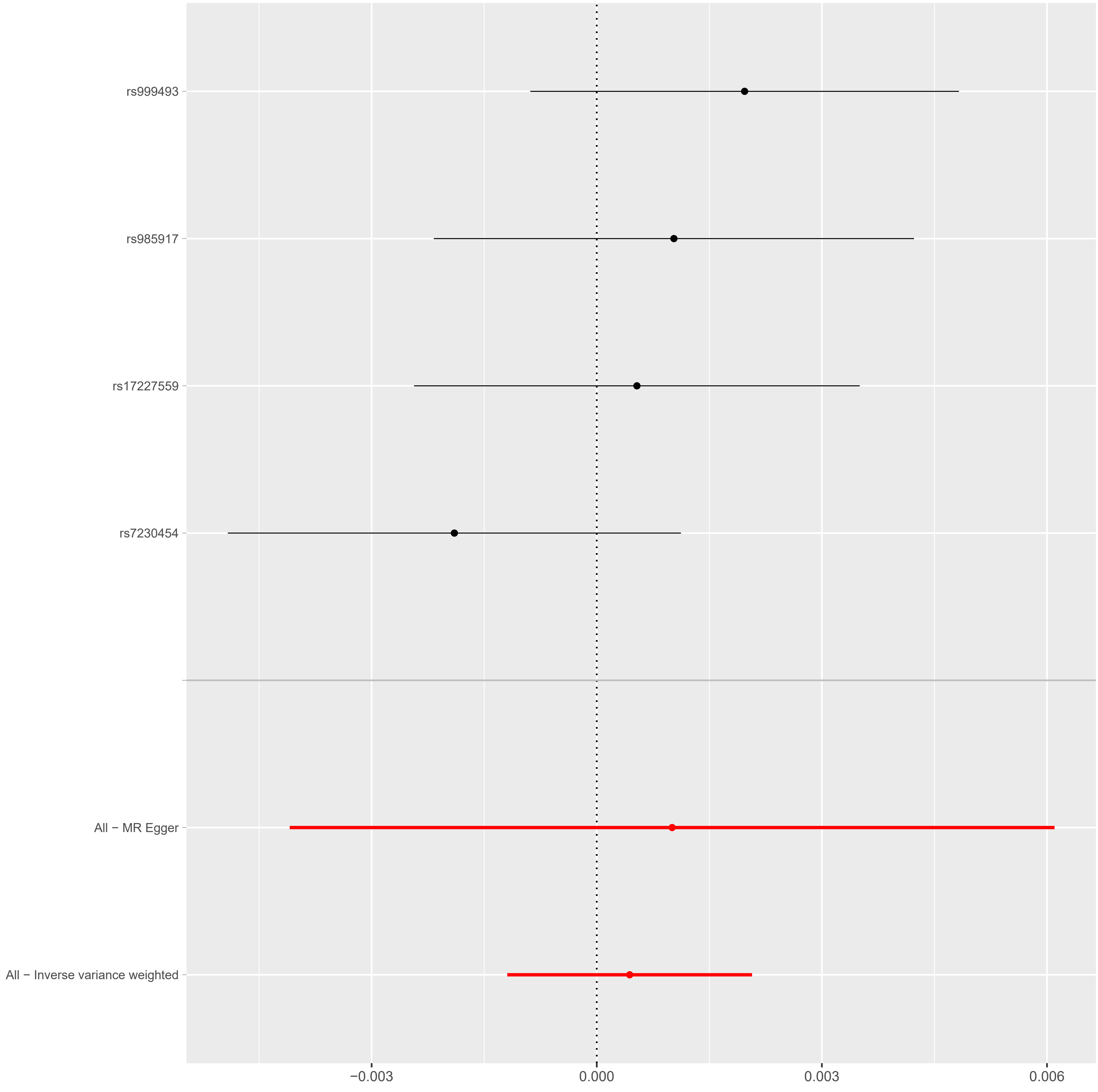

Forest plots for MR analyses of the causal effect of IGFBP-5 using each SNP singly on osteoporosis based on UK trait

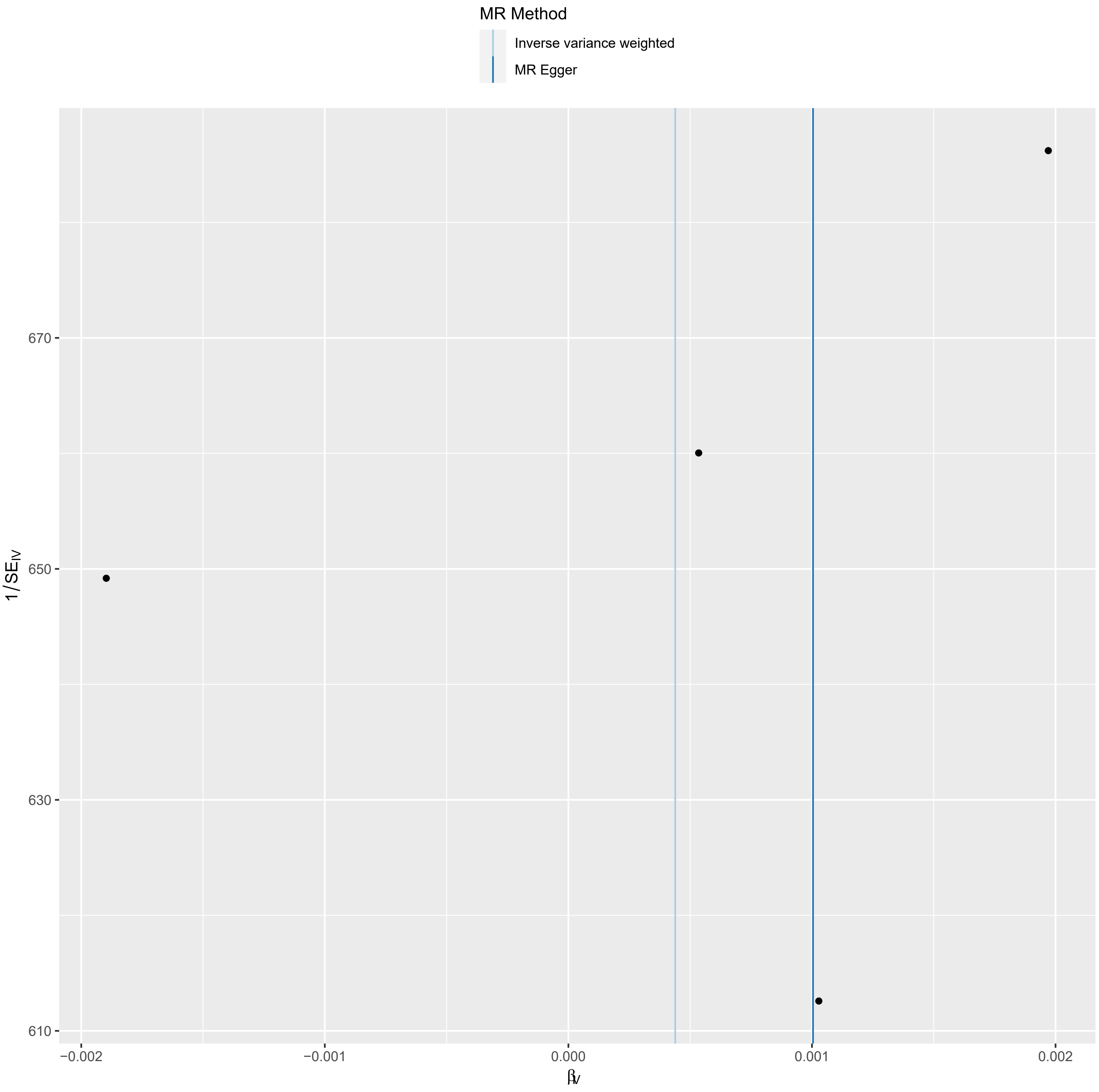

Funnal plots to assess heterogeneity for IGFBP-5 using all SNPs with the MR Egger and IVW methods

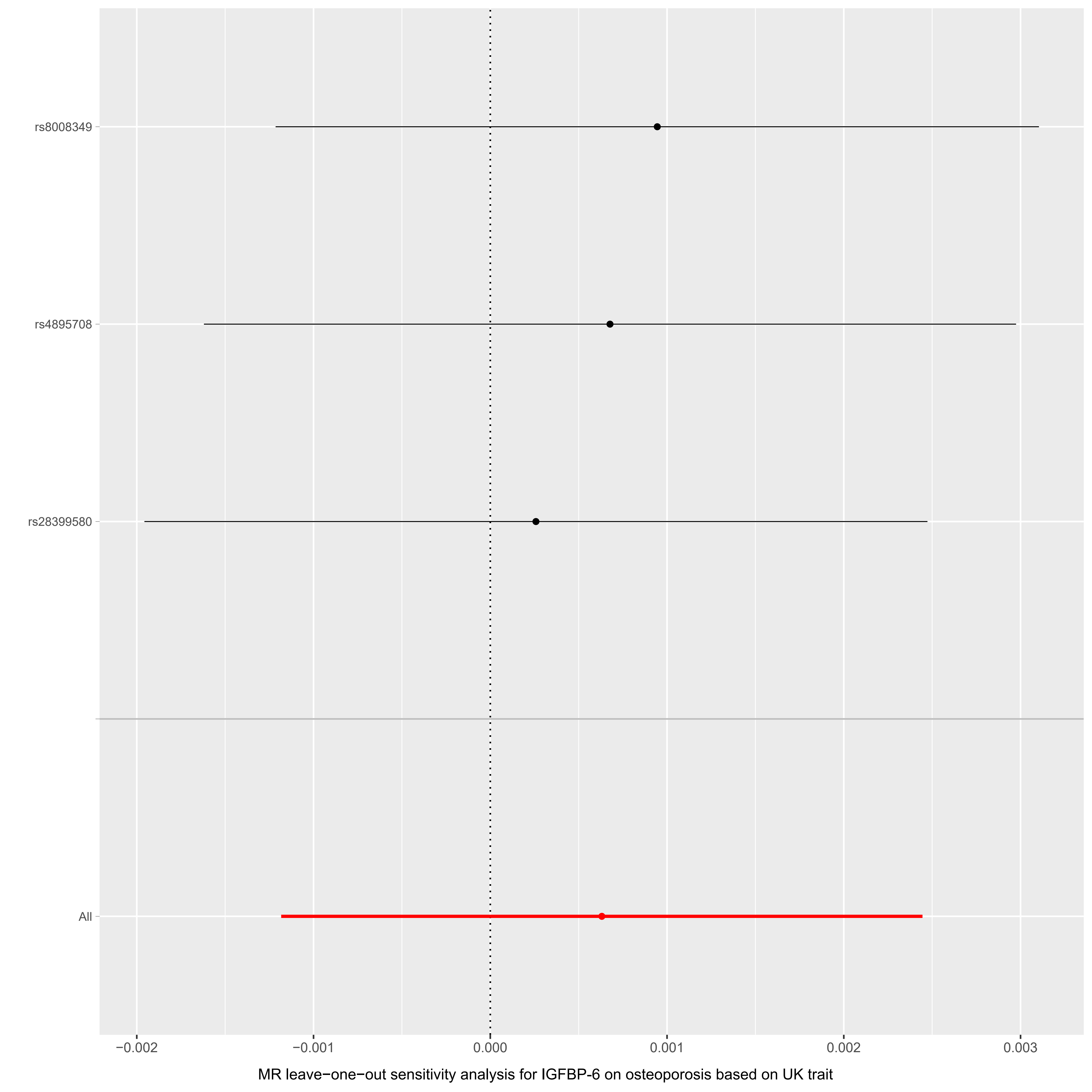
rs8008349
rs4895708
rs28399580
All
−0.002
−0.001
0.000
0.001
0.002
0.003
MR leave−one−out sensitivity analysis for IGFBP-6 on osteoporosis based on UK trait

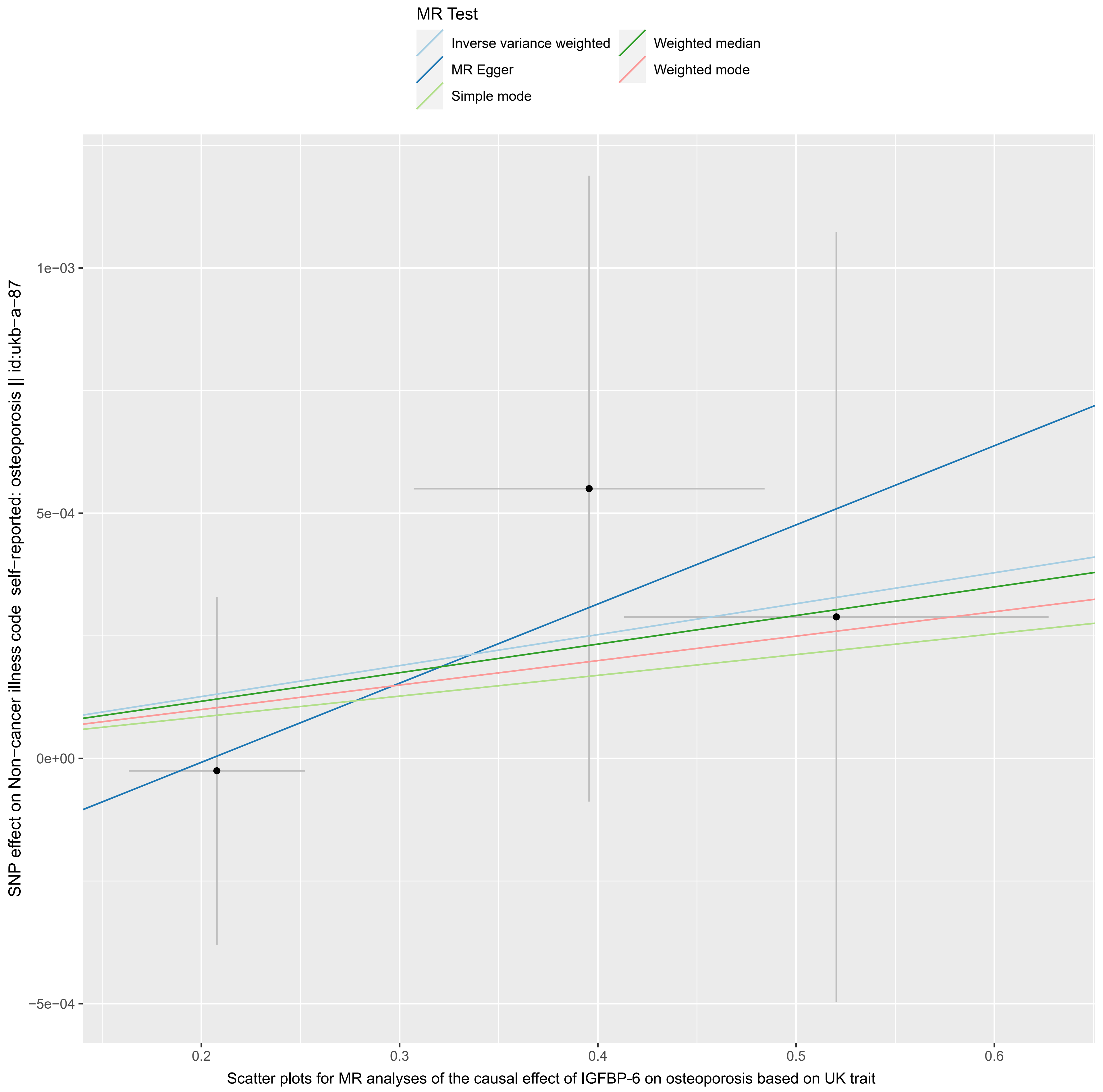
MR Test
Inverse variance weighted
MR Egger
Simple mode
Weighted median
Weighted mode
1e−03
5e−04
0e+00
−5e−04
0.2
0.3
0.4
0.5
0.6
SNP effect on Non−cancer illness code self−reported: osteoporosis || id:ukb−a−87
Scatter plots for MR analyses of the causal effect of IGFBP-6 on osteoporosis based on UK trait

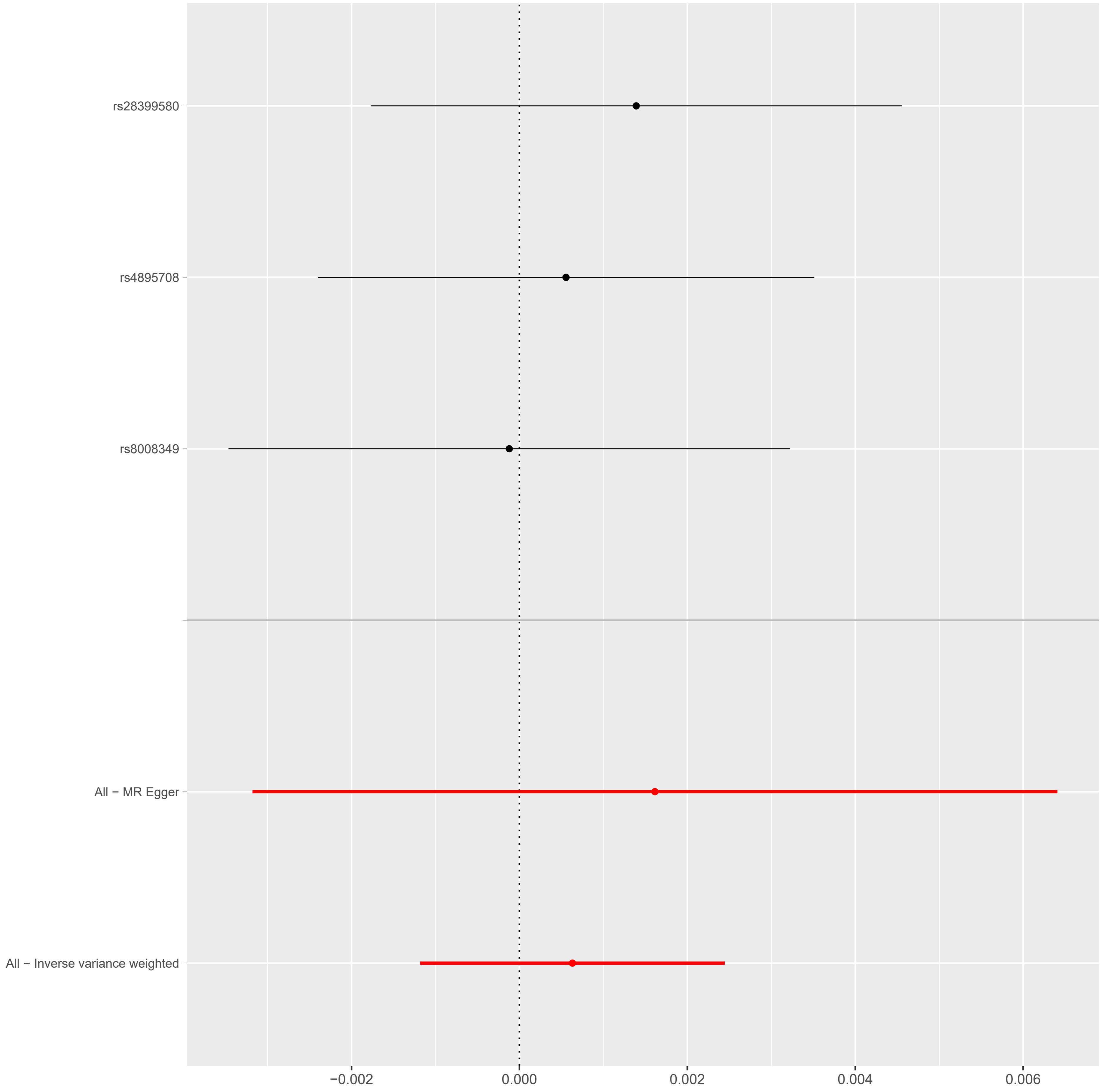

Forest plots for MR analyses of the causal effect of IGFBP-6 using each SNP singly on osteoporosis based on UK trait

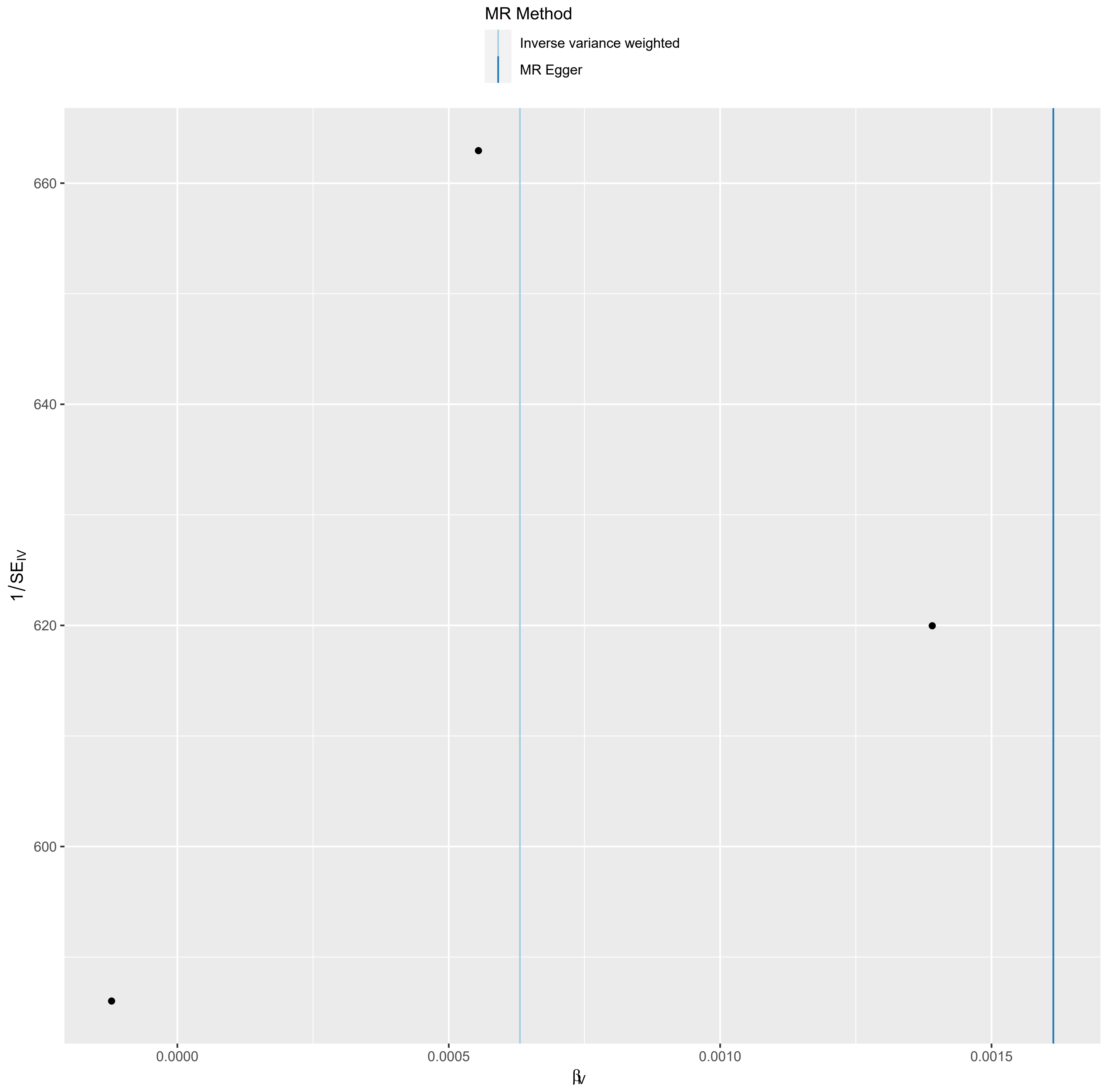

Funnal plots to assess heterogeneity for IGFBP-6 using all SNPs with the MR Egger and IVW methods

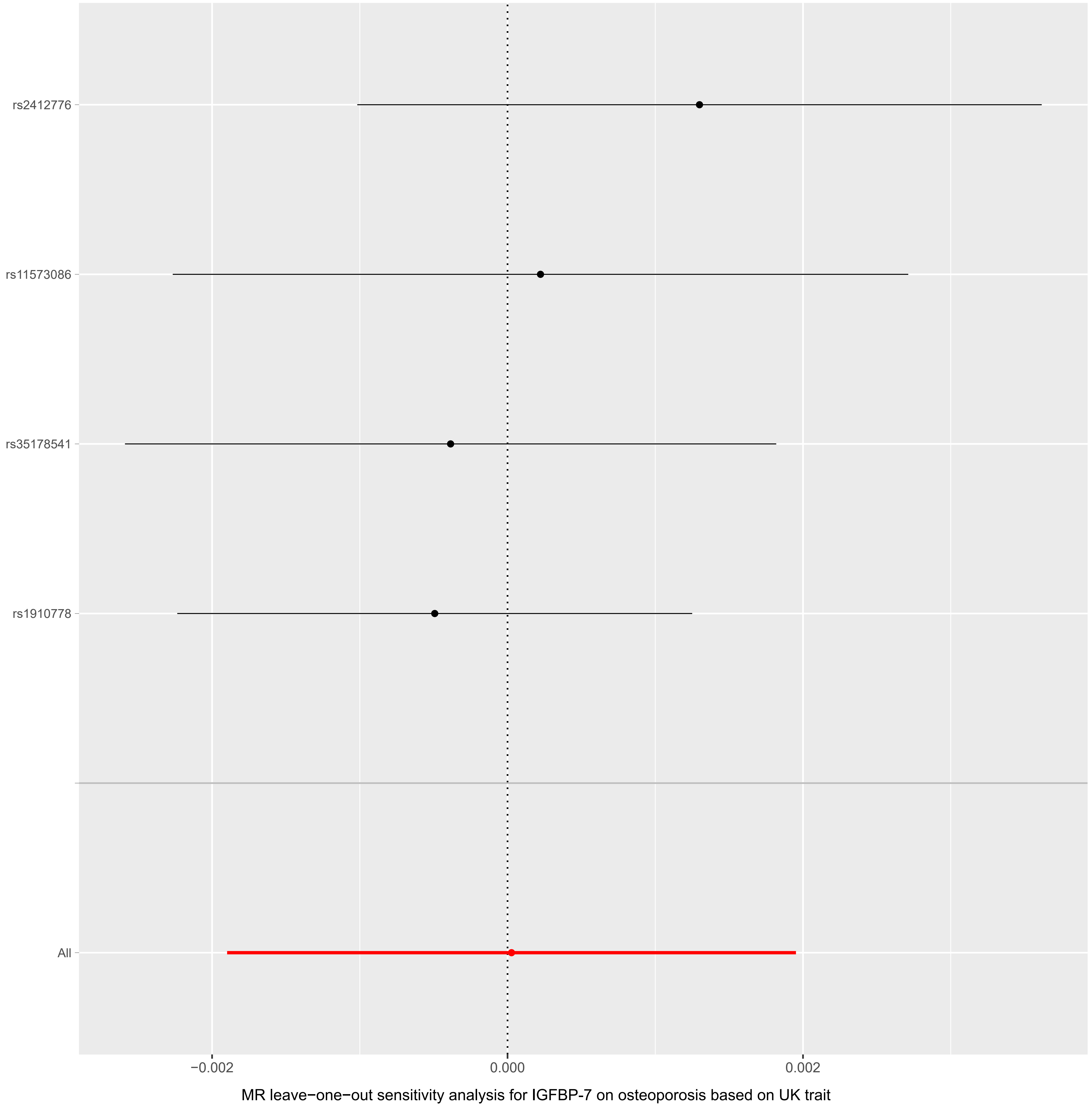
rs2412776
rs11573086
rs35178541
rs1910778
All
−0.002
0.000
0.002
MR leave−one−out sensitivity analysis for IGFBP-7 on osteoporosis based on UK trait

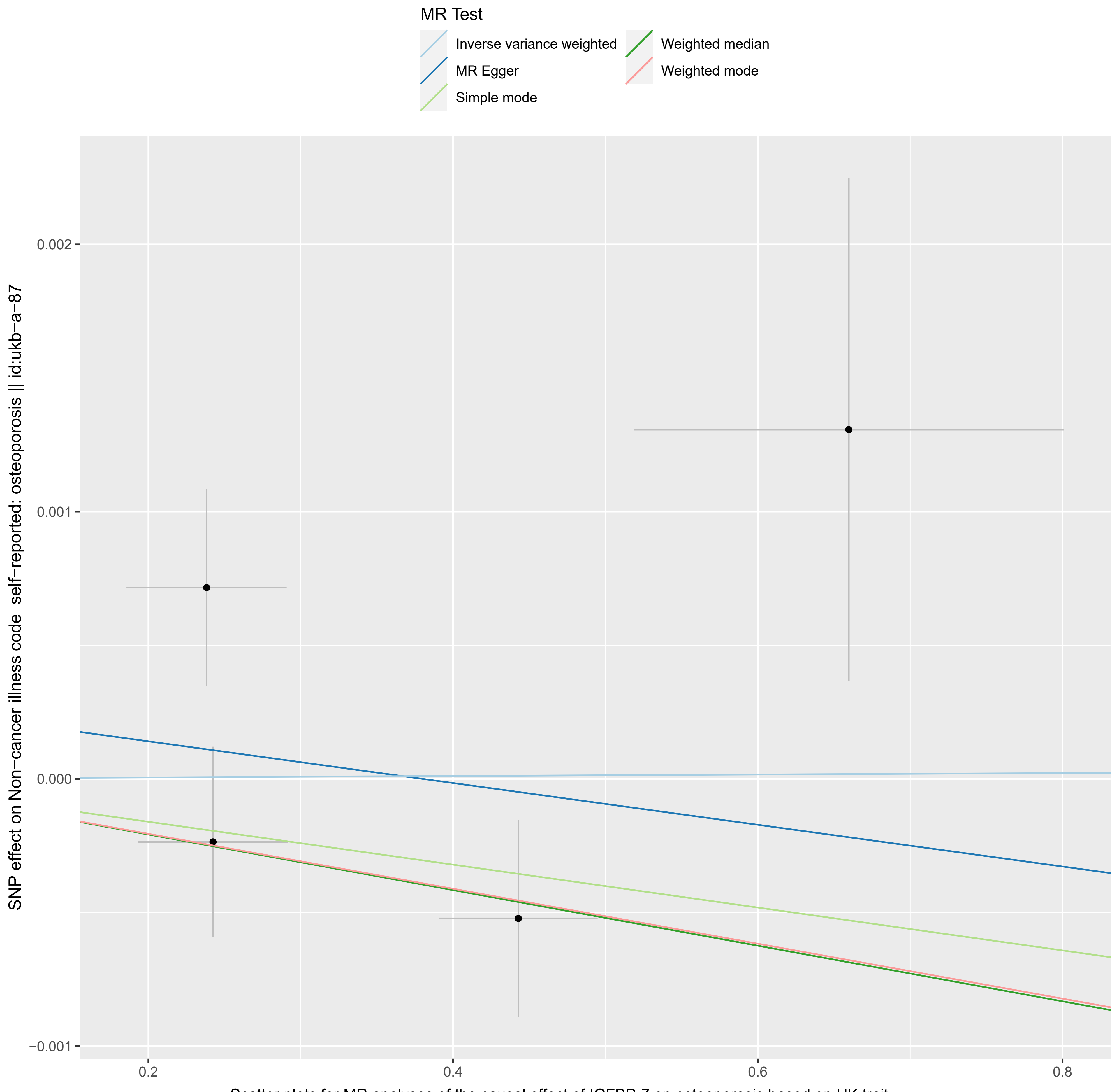

Scatter plots for MR analyses of the causal effect of IGFBP-7 on osteoporosis based on UK trait

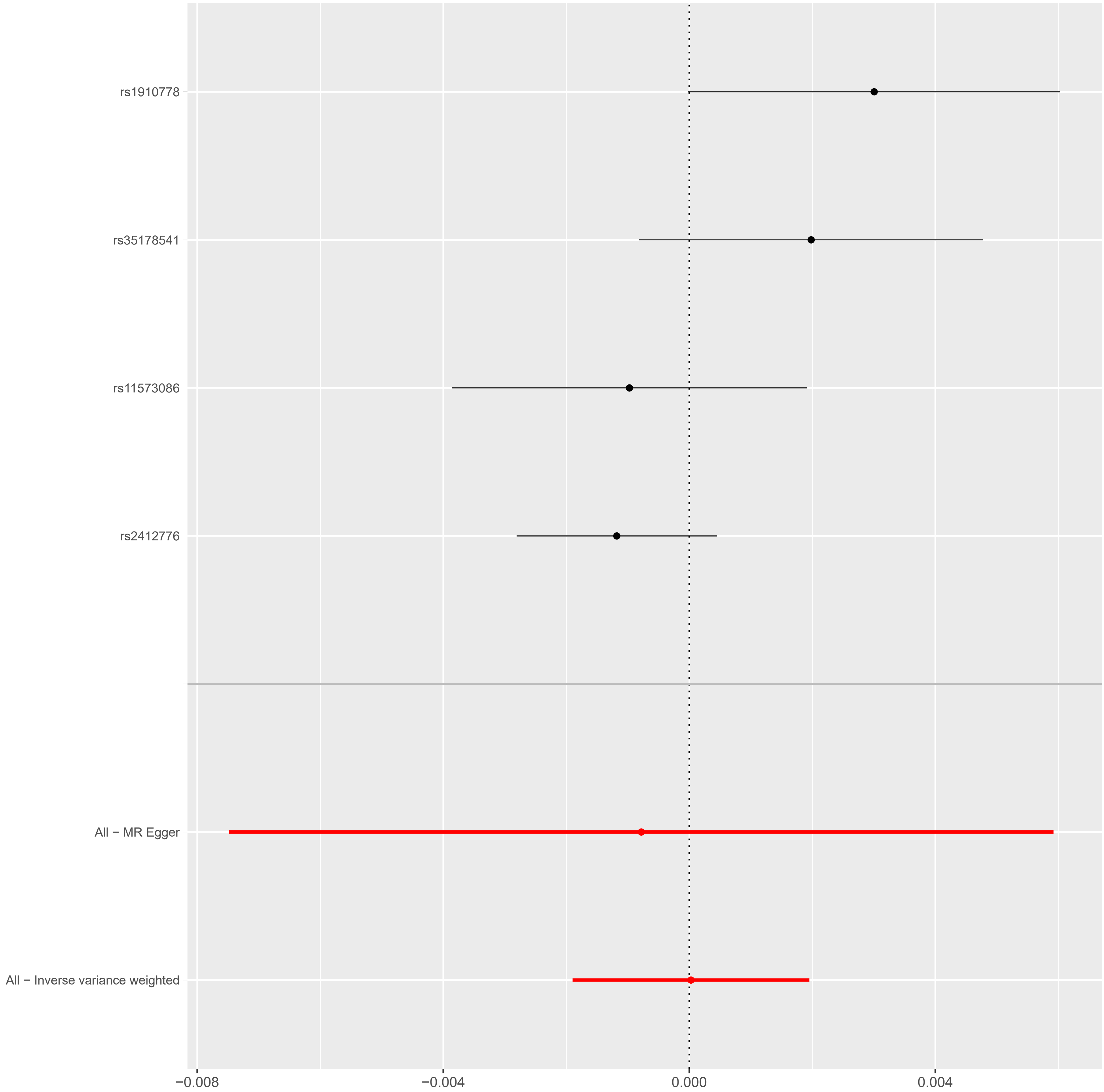

Forest plots for MR analyses of the causal effect of IGFBP-7 using each SNP singly on osteoporosis based on UK trait

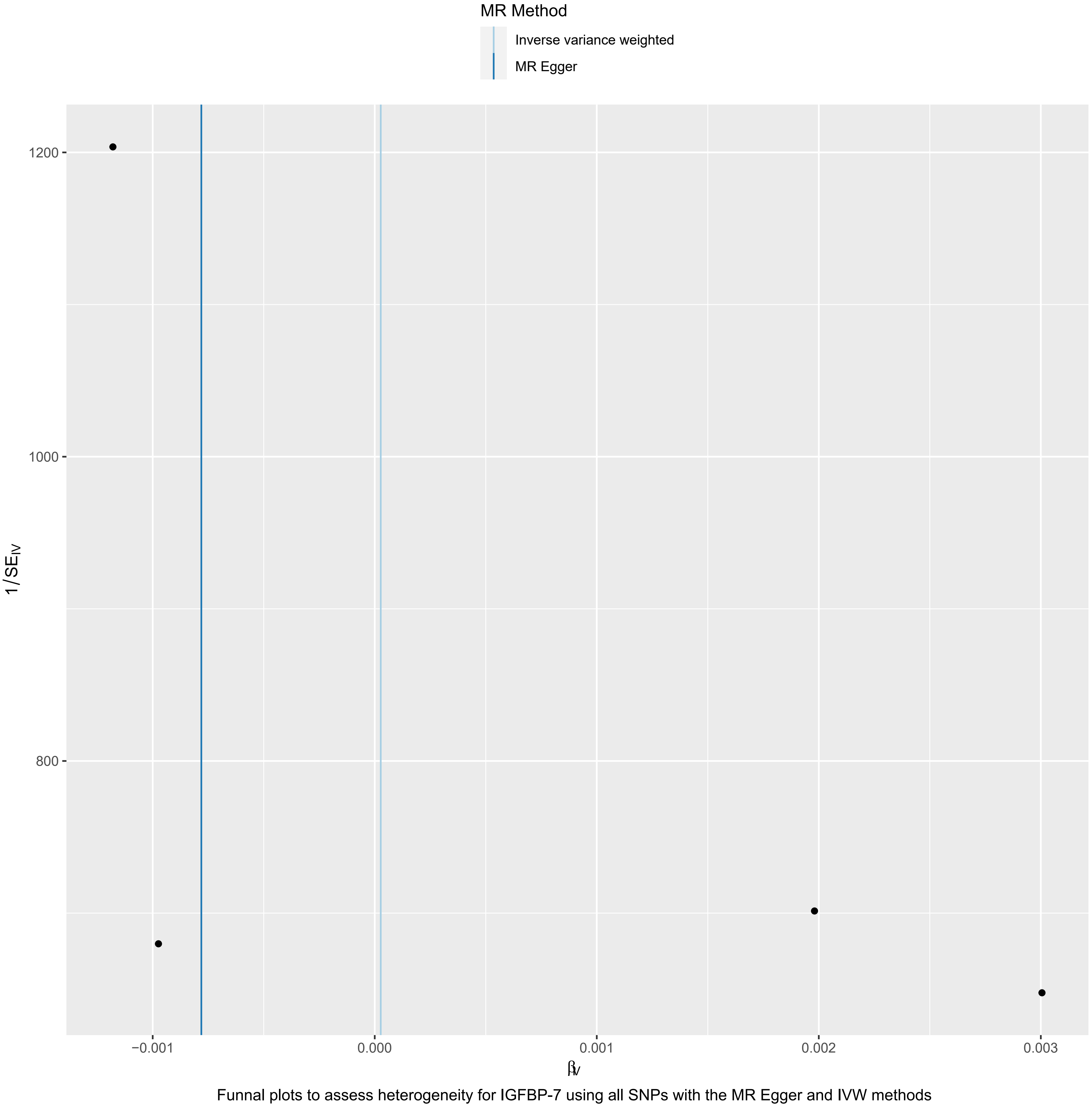

Funnal plots to assess heterogeneity for IGFBP-7 using all SNPs with the MR Egger and IVW methods

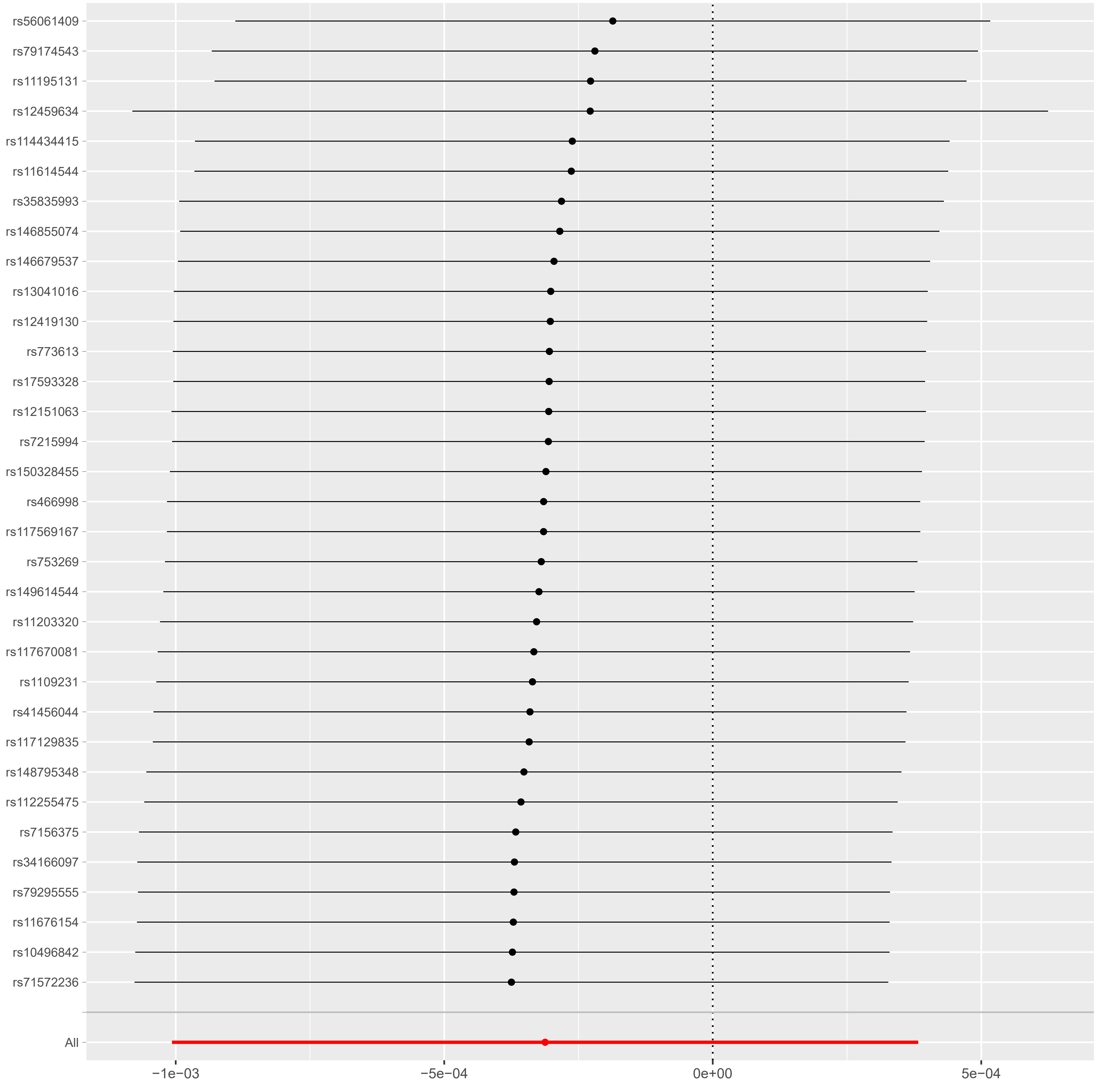

MR leave−one−out sensitivity analysis for IGF-LR1 on osteoporosis based on UK trait

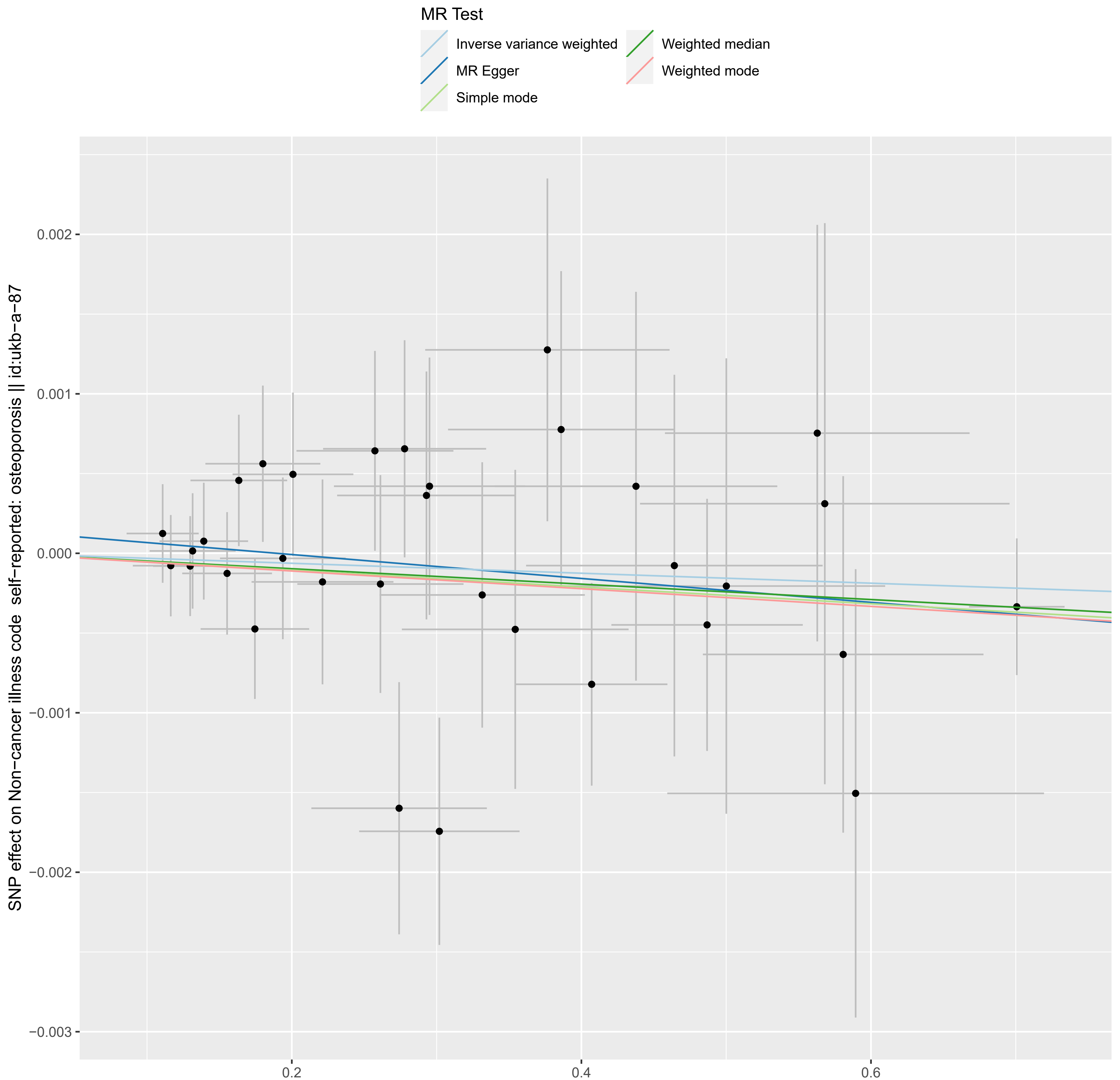

Scatter plots for MR analyses of the causal effect of IGF-LR1 on osteoporosis based on UK trait

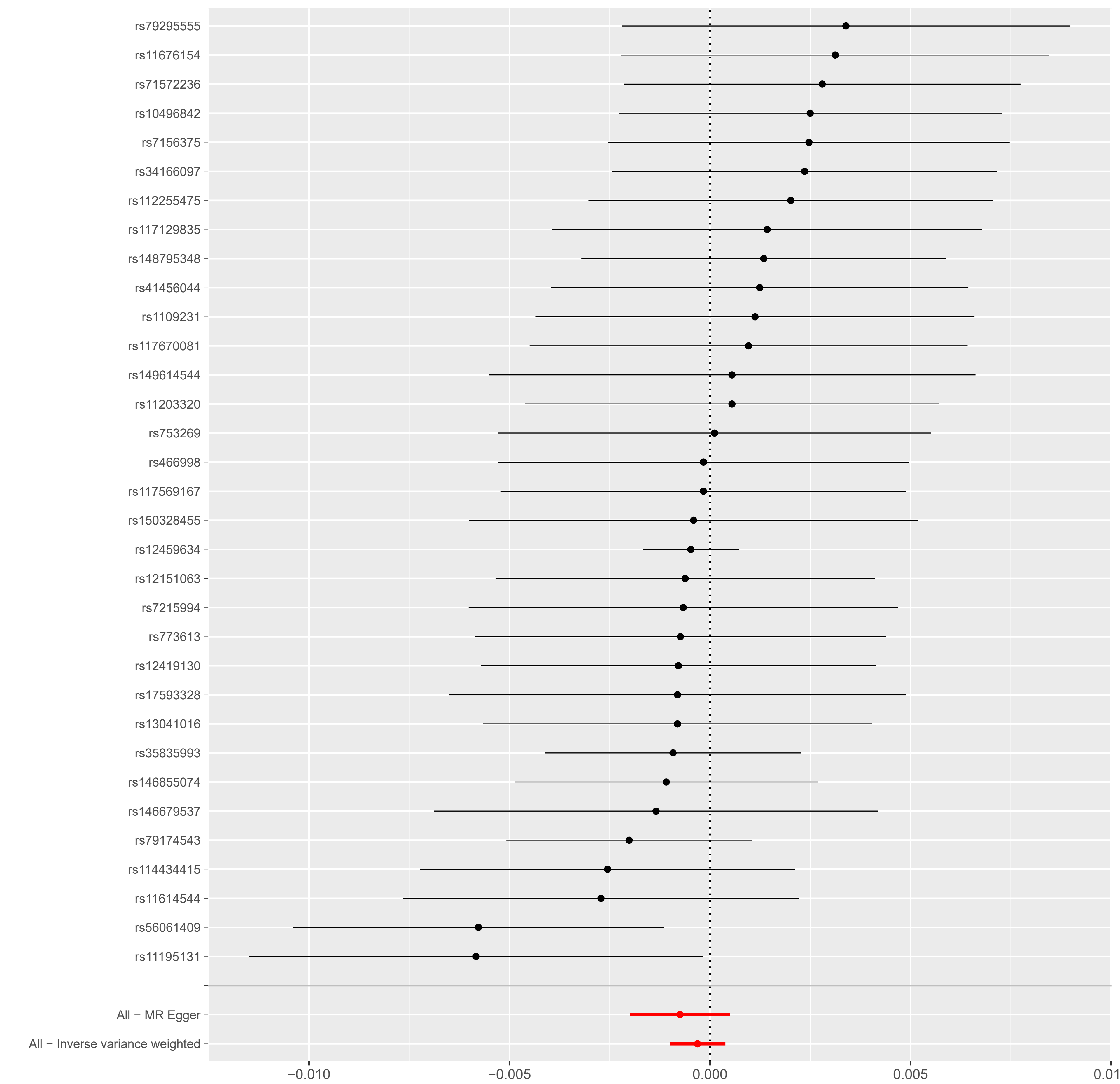

Forest plots for MR analyses of the causal effect of IGF-LR1 using each SNP singly on osteoporosis based on UK trait

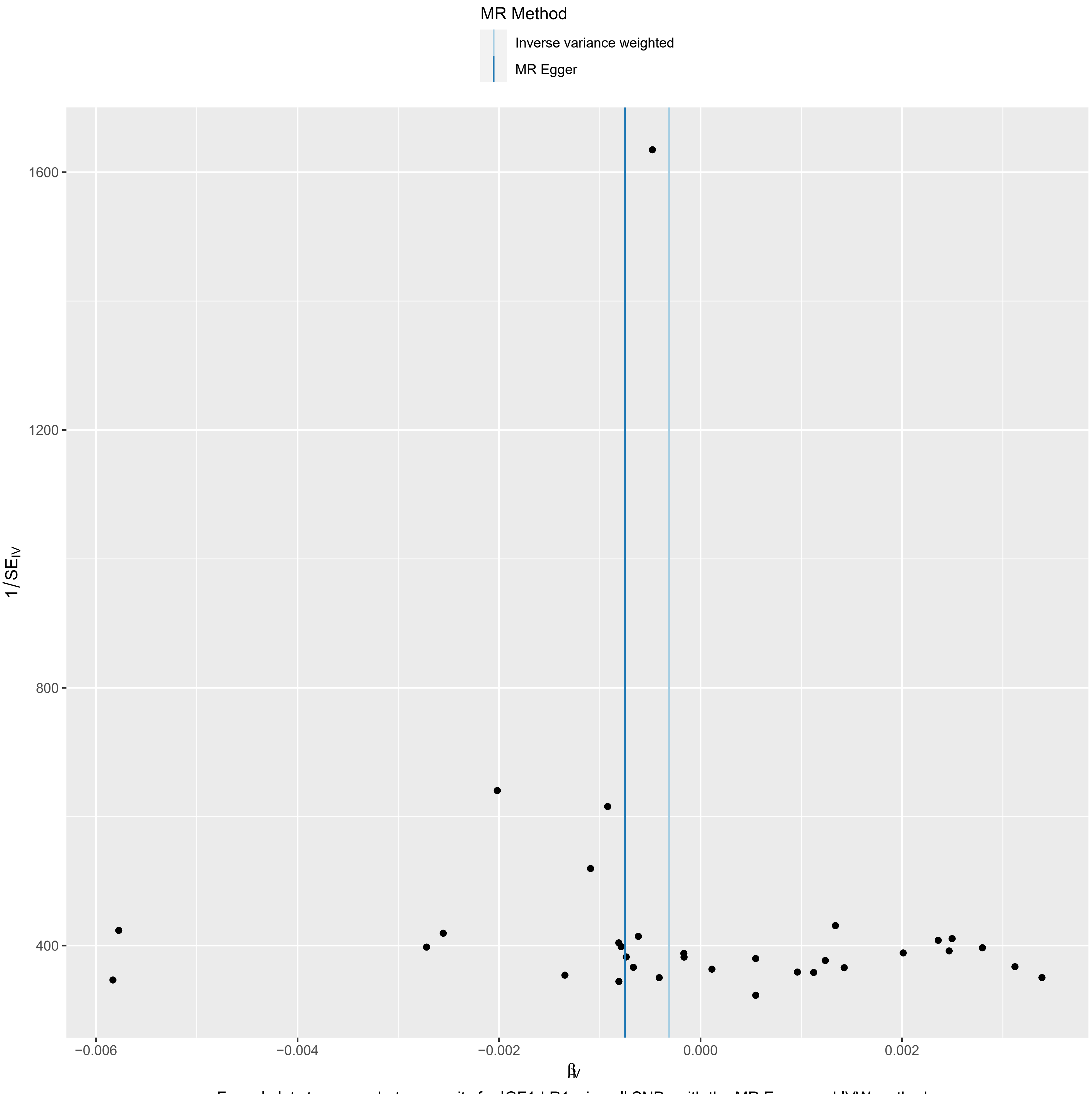

Funnal plots to assess heterogeneity for IGF1-LR1using all SNPs with the MR Egger and IVW methods

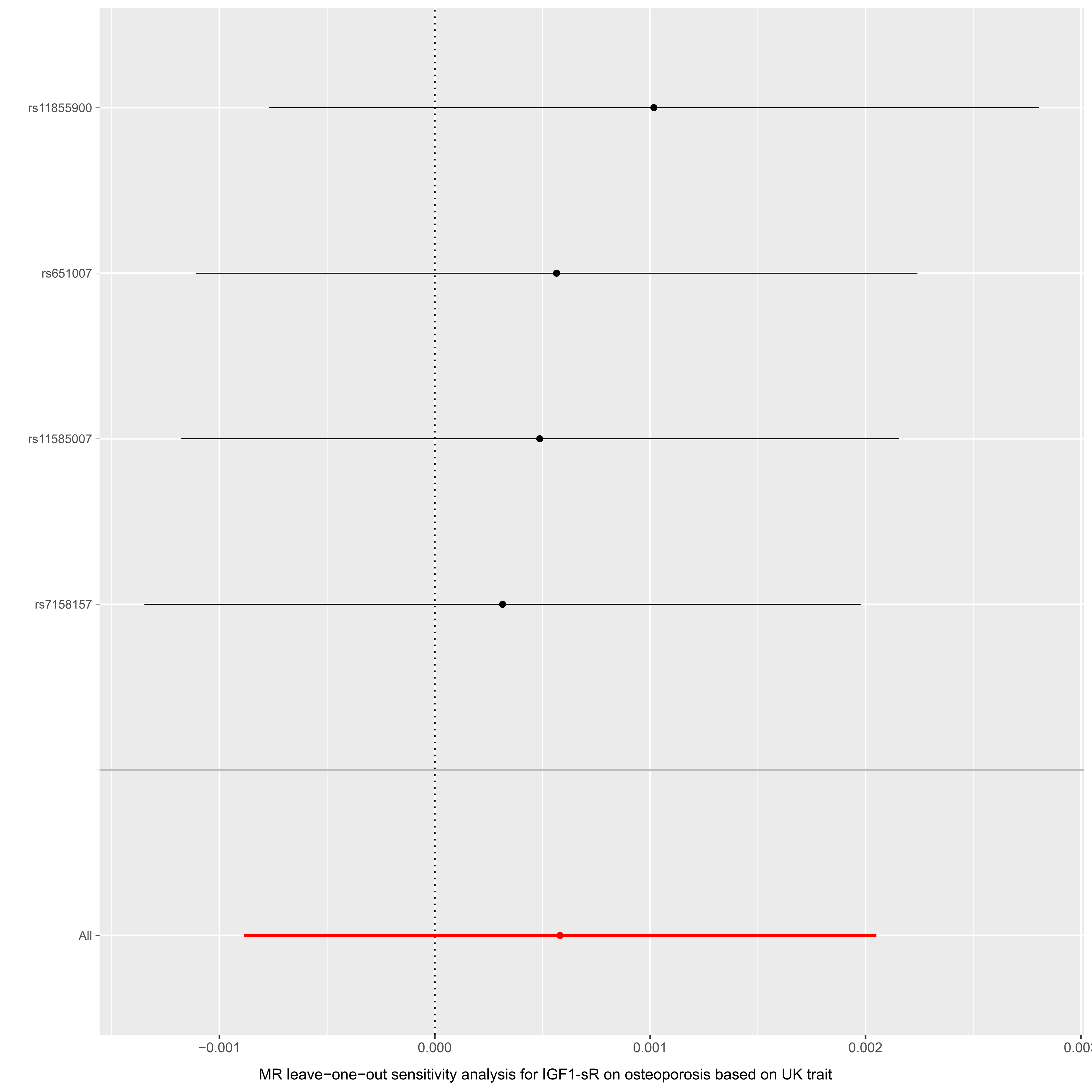
rs11855900
rs651007
rs11585007
rs7158157
All
−0.001
0.000
0.001
0.002
MR leave−one−out sensitivity analysis for IGF1-sR on osteoporosis based on UK trait

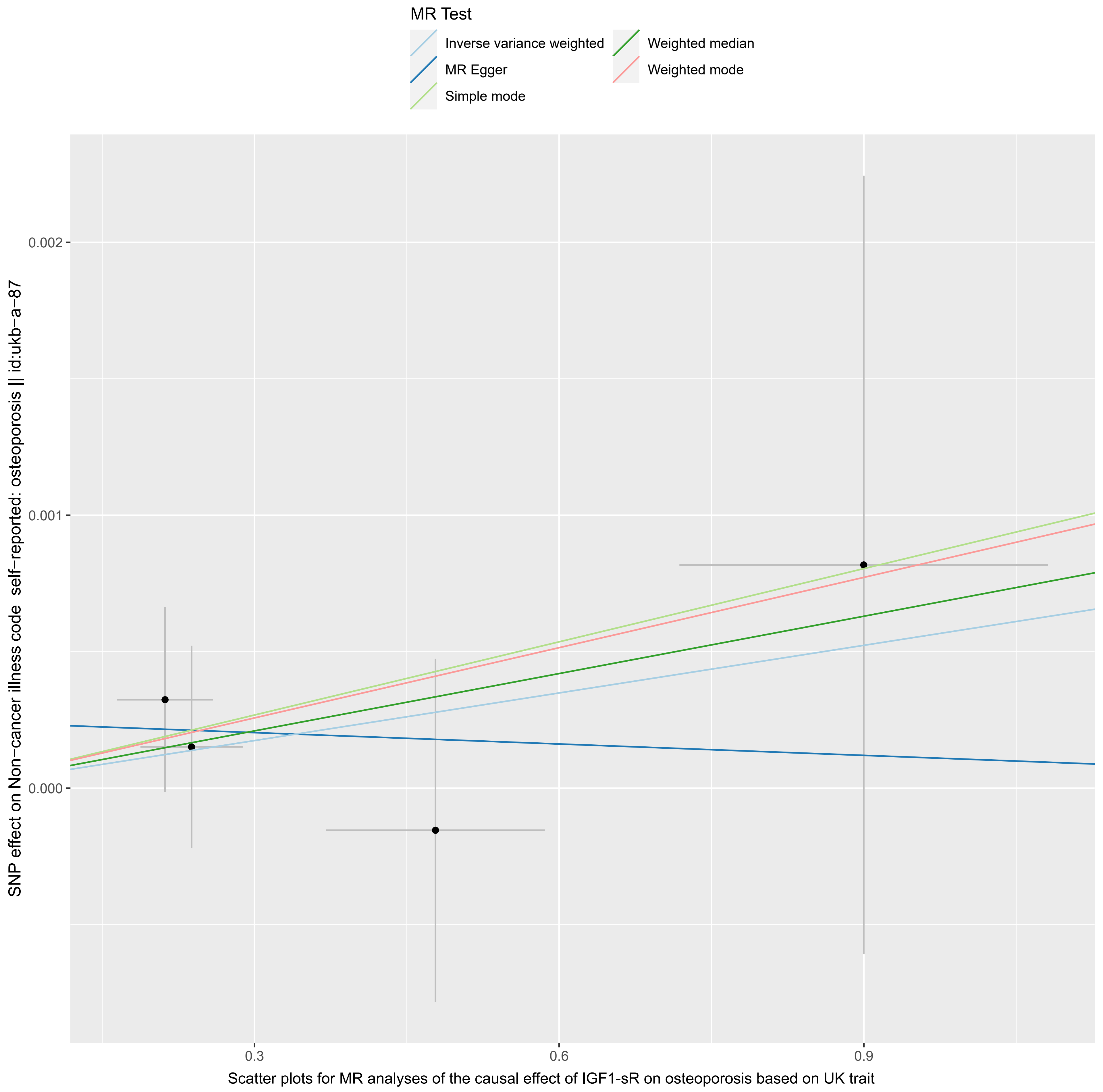

MR Test
Inverse variance weighted
MR Egger
Simple mode
Weighted median
Weighted mode
SNP effect on Non−cancer illness code self−reported: osteoporosis || id:ukb−a−87
0.002
0.001
0.000
0.3
0.6
0.9
Scatter plots for MR analyses of the causal effect of IGF1-sR on osteoporosis based on UK trait

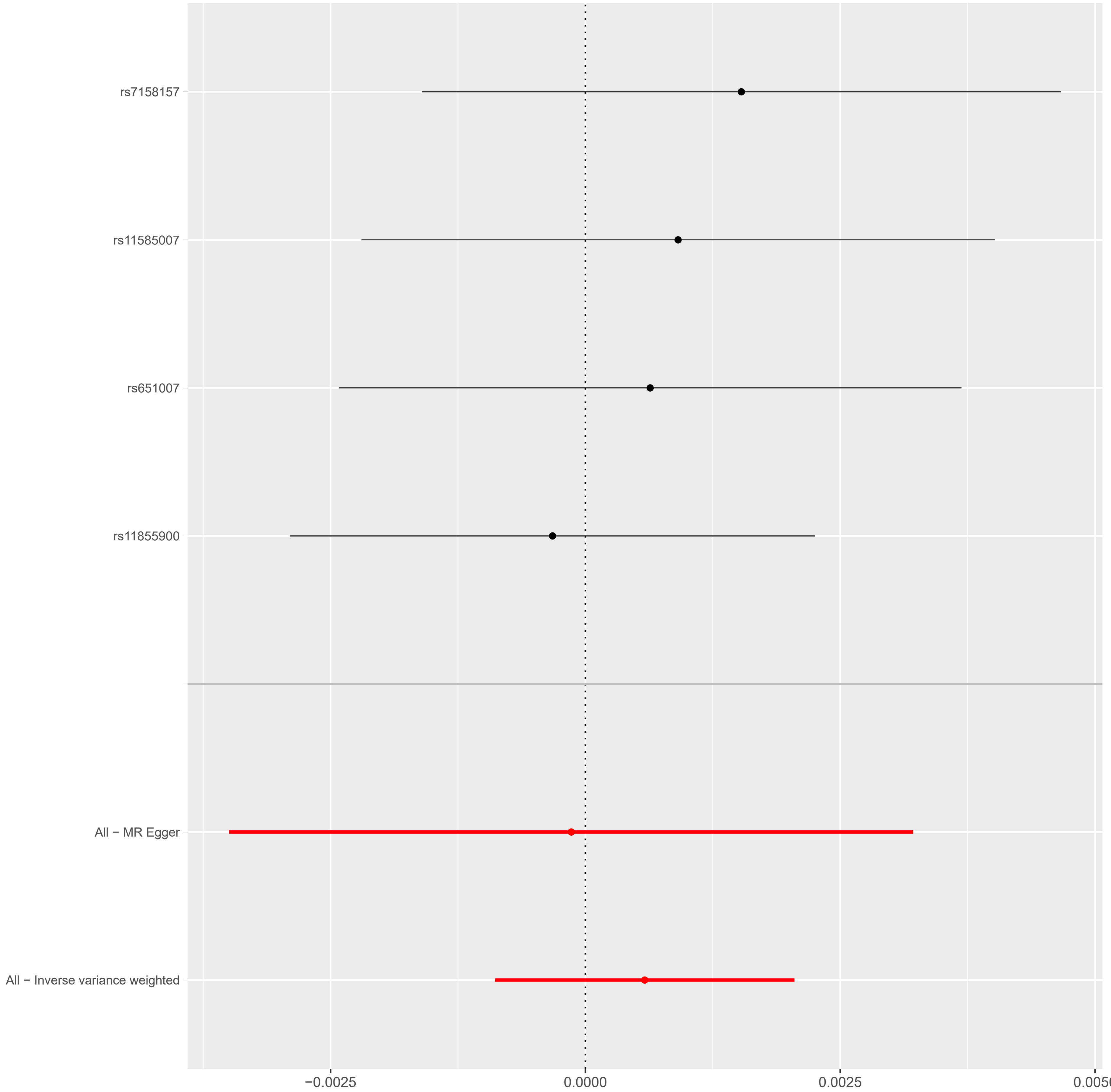

Forest plots for MR analyses of the causal effect of IGF1-sR using each SNP singly on osteoporosis based on UK trait

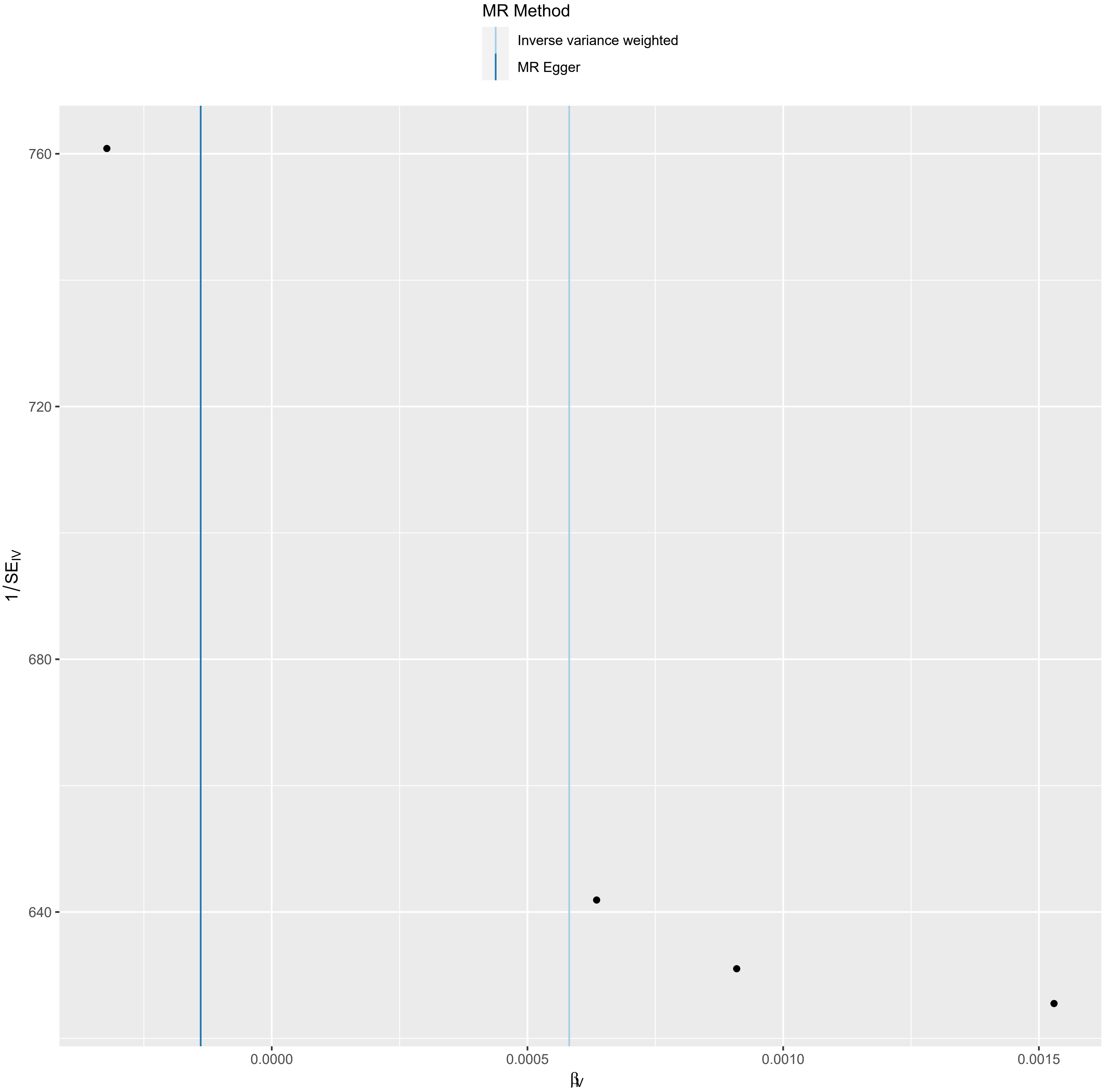

Funnal plots to assess heterogeneity for IGF1-sR using all SNPs with the MR Egger and IVW methods

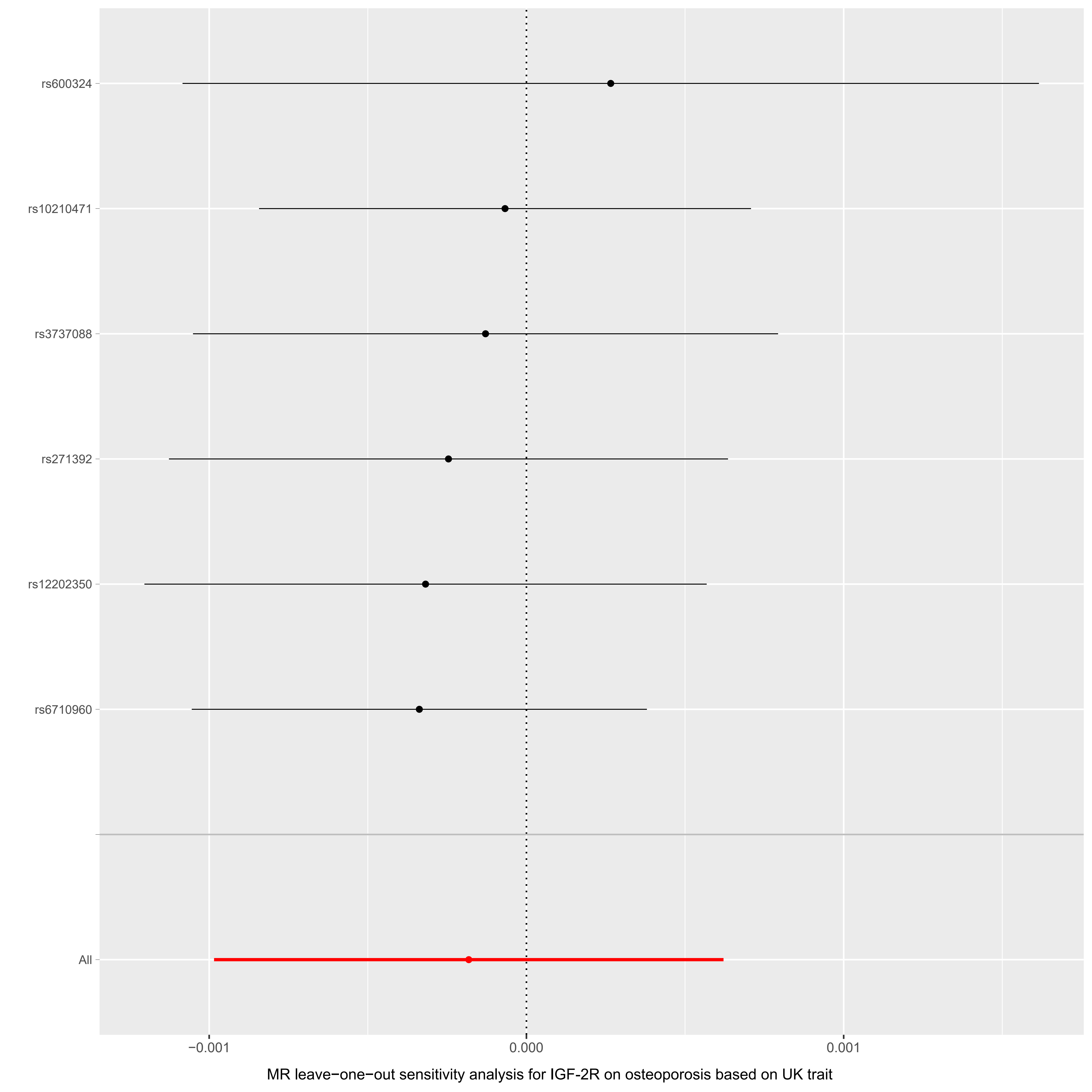

rs600324
rs10210471
rs3737088
rs271392
rs12202350
rs6710960
All
−0.001
0.000
0.001
MR leave−one−out sensitivity analysis for IGF-2R on osteoporosis based on UK trait

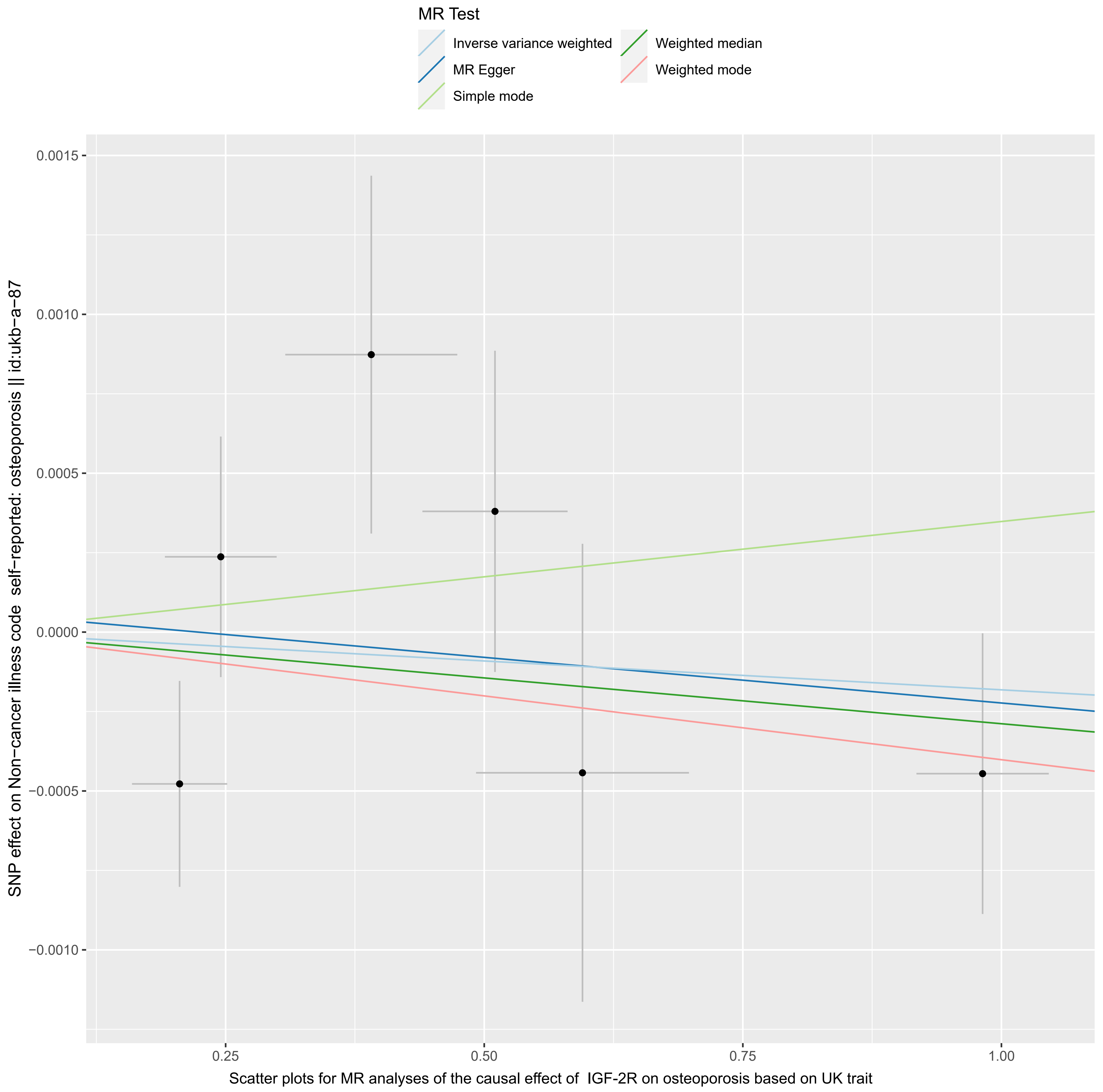

MR Test
Inverse variance weighted
MR Egger
Simple mode
Weighted median
Weighted mode
SNP effect on Non−cancer illness code self−reported: osteoporosis || id:ukb−a−87
0.0015
0.0010
0.0005
0.0000
−0.0005
−0.0010
0.25
0.50
0.75
1.00
Scatter plots for MR analyses of the causal effect of IGF-2R on osteoporosis based on UK trait

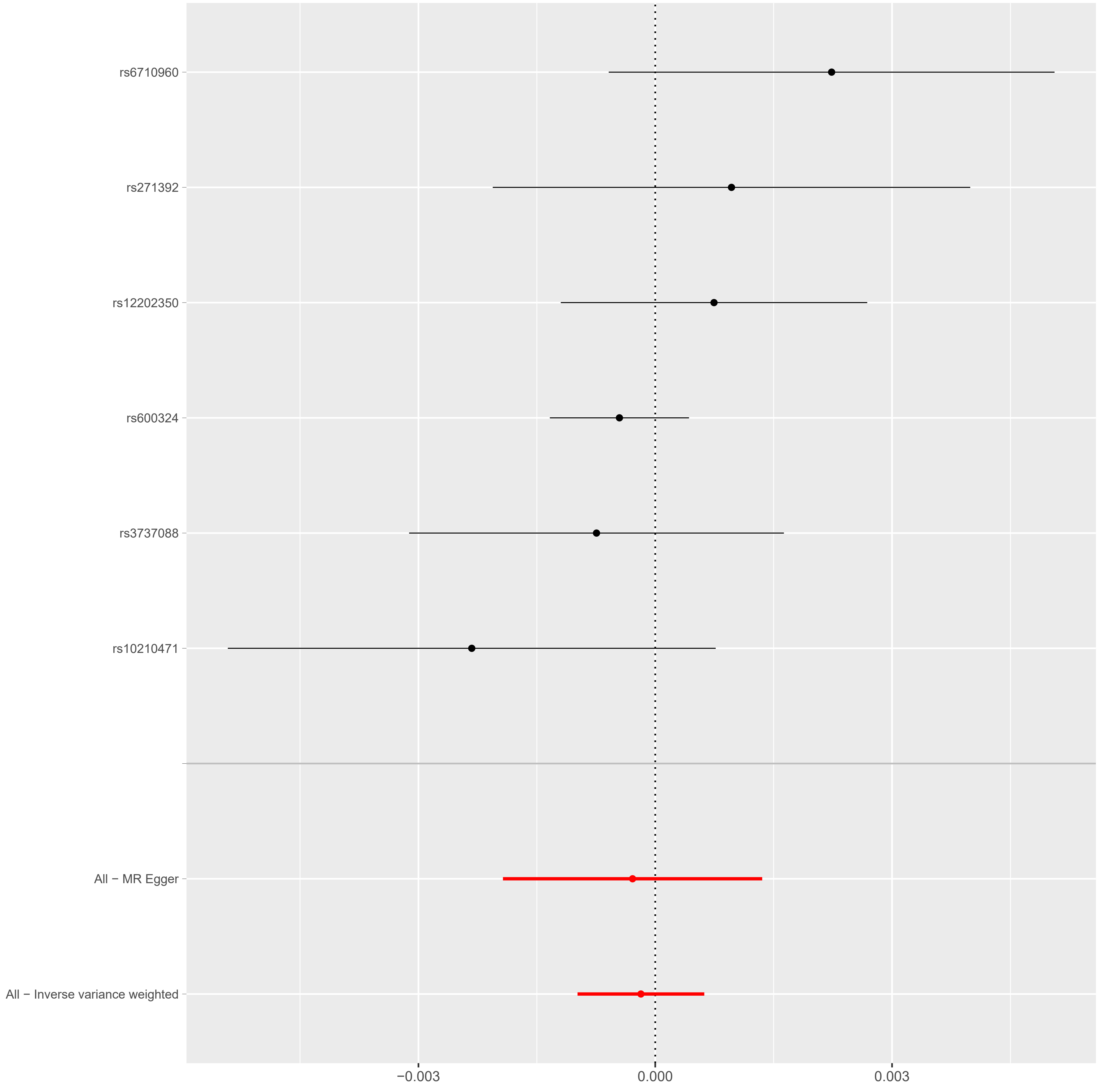

Forest plots for MR analyses of the causal effect of IGF-2R using each SNP singly on osteoporosis based on UK trait

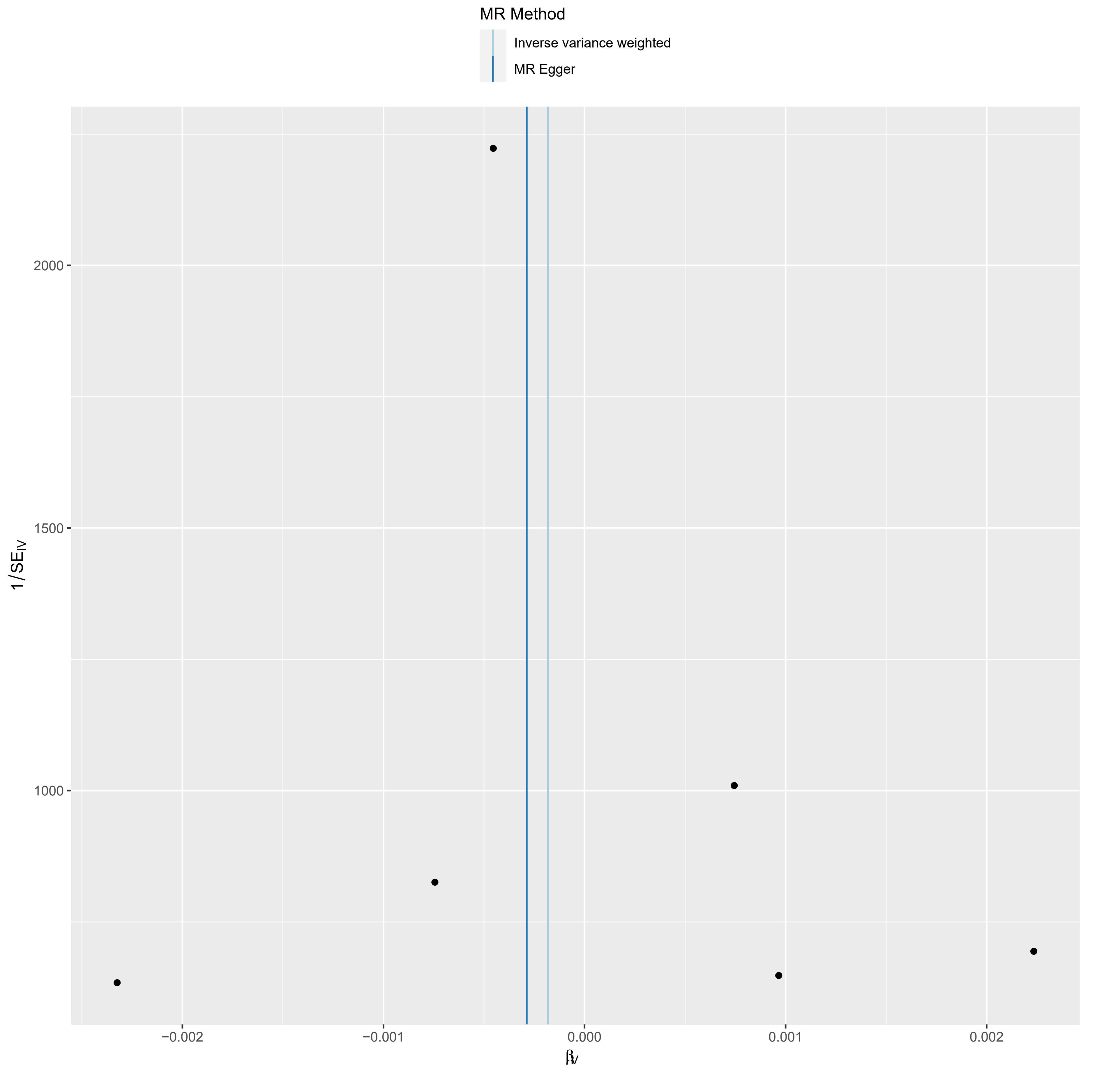

Funnal plots to assess heterogeneity for IGF-2R using all SNPs with the MR Egger and IVW methods

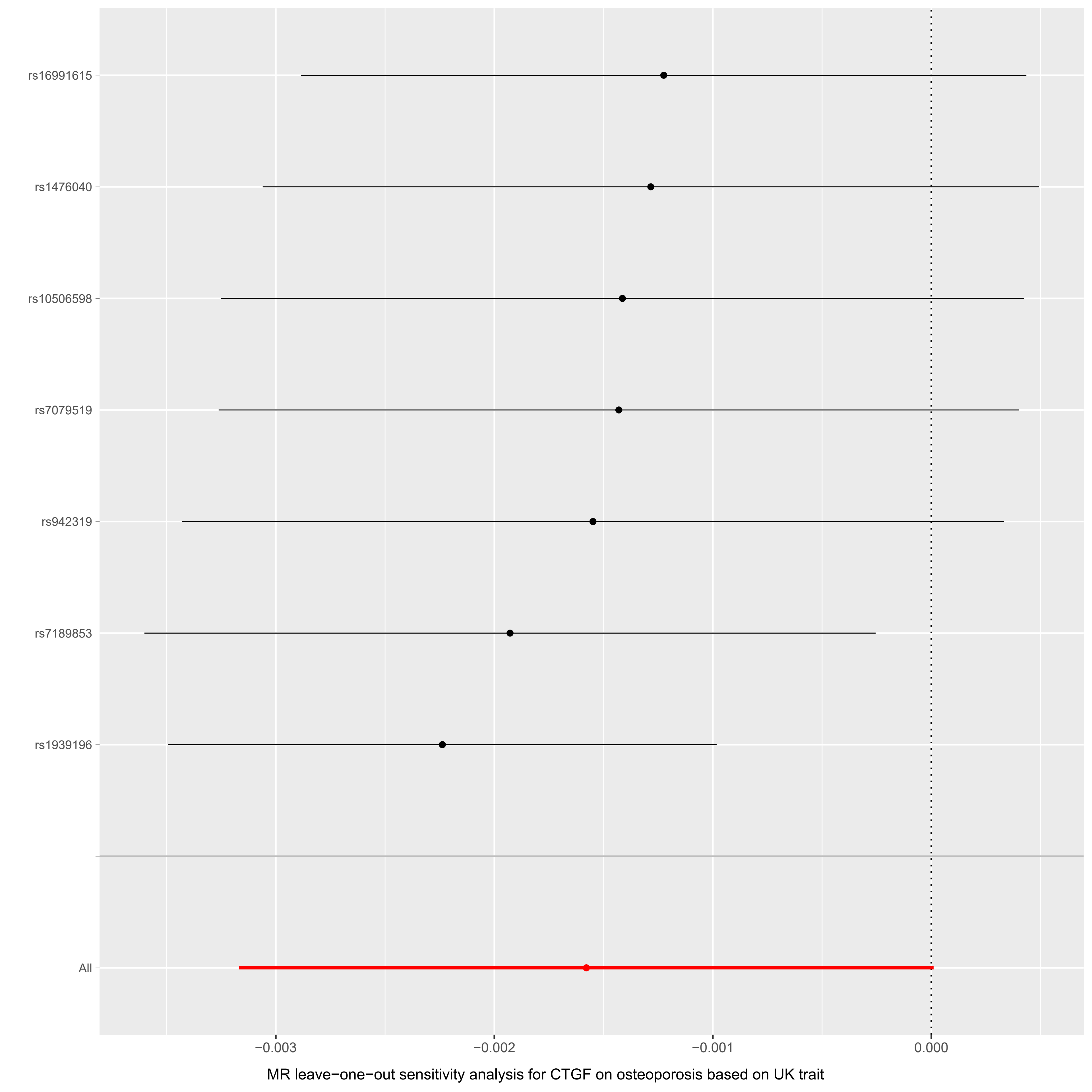

rs16991615
rs1476040
rs10506598
rs7079519
rs942319
rs7189853
rs1939196
All
−0.003
−0.002
−0.001
0.000
MR leave−one−out sensitivity analysis for CTGF on osteoporosis based on UK trait

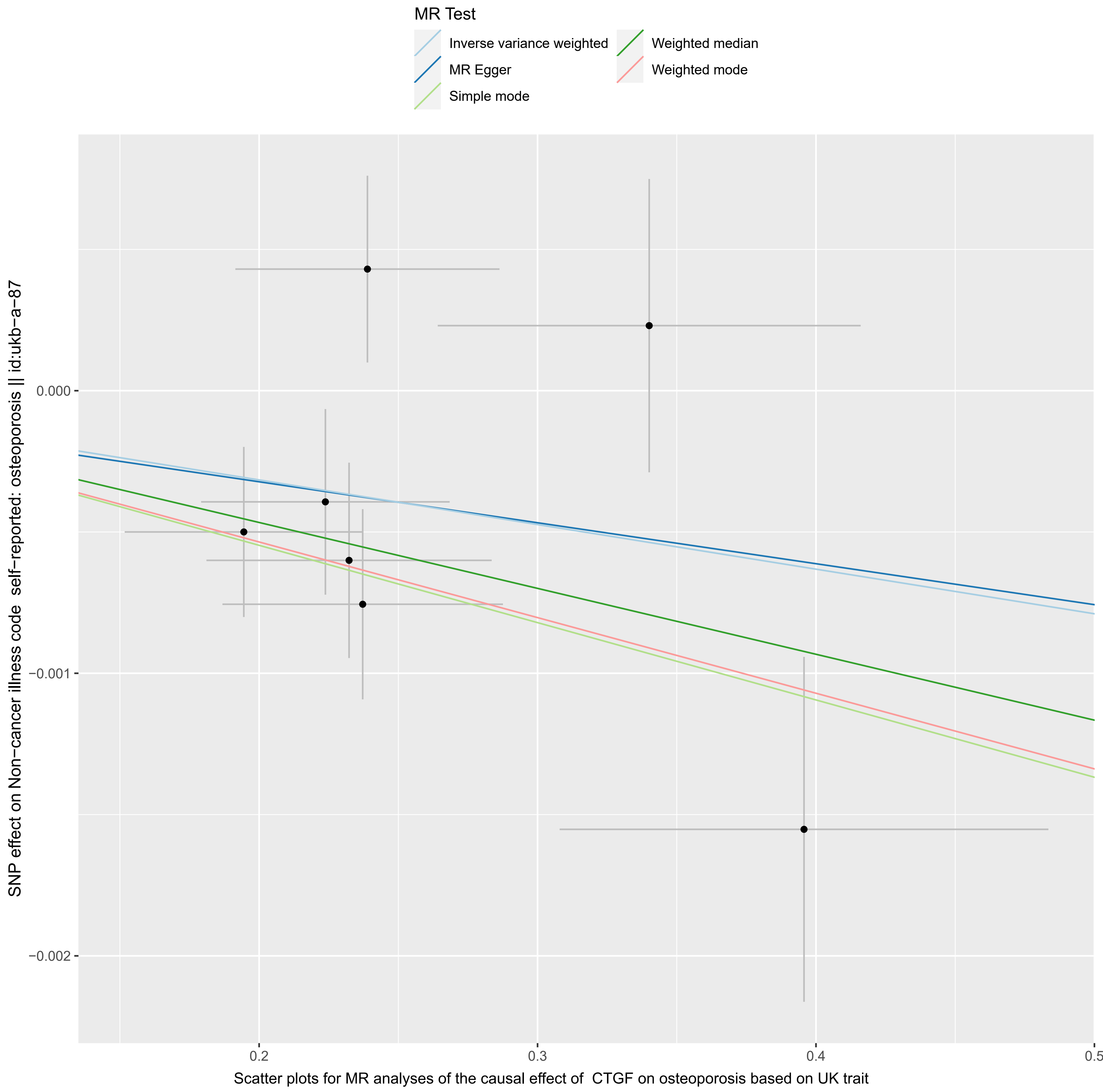

MR Test
Inverse variance weighted
MR Egger
Simple mode
Weighted median
Weighted mode
SNP effect on Non-cancer illness code self-reported: osteoporosis || id:ukb-a-87
0.000
-0.001
-0.002
0.2
0.3
0.4
0.5
Scatter plots for MR analyses of the causal effect of CTGF on osteoporosis based on UK trait

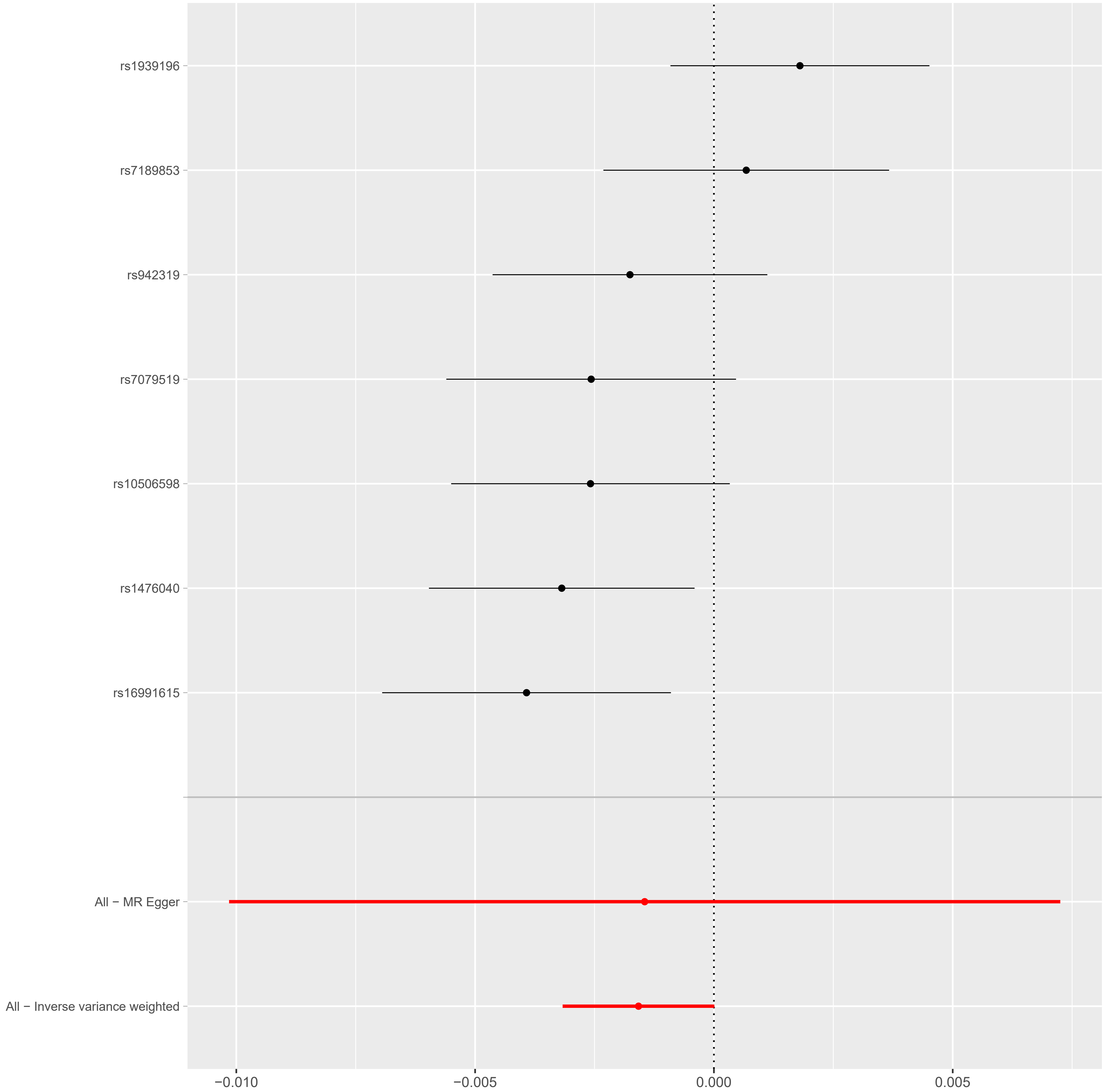

Forest plots for MR analyses of the causal effect of CTGF using each SNP singly on osteoporosis based on UK trait

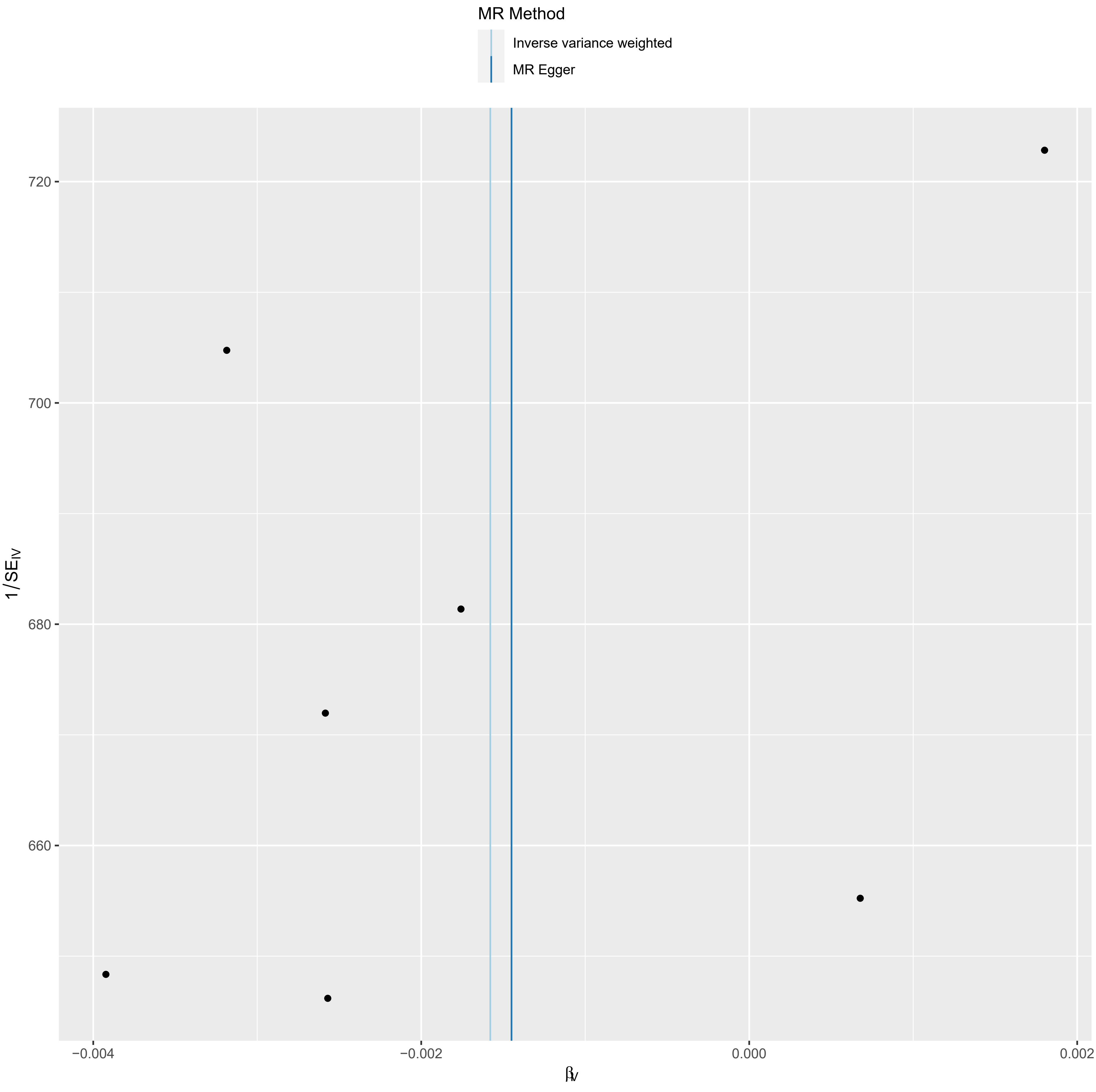

Funnal plots to assess heterogeneity for CTGF using all SNPs with the MR Egger and IVW methods

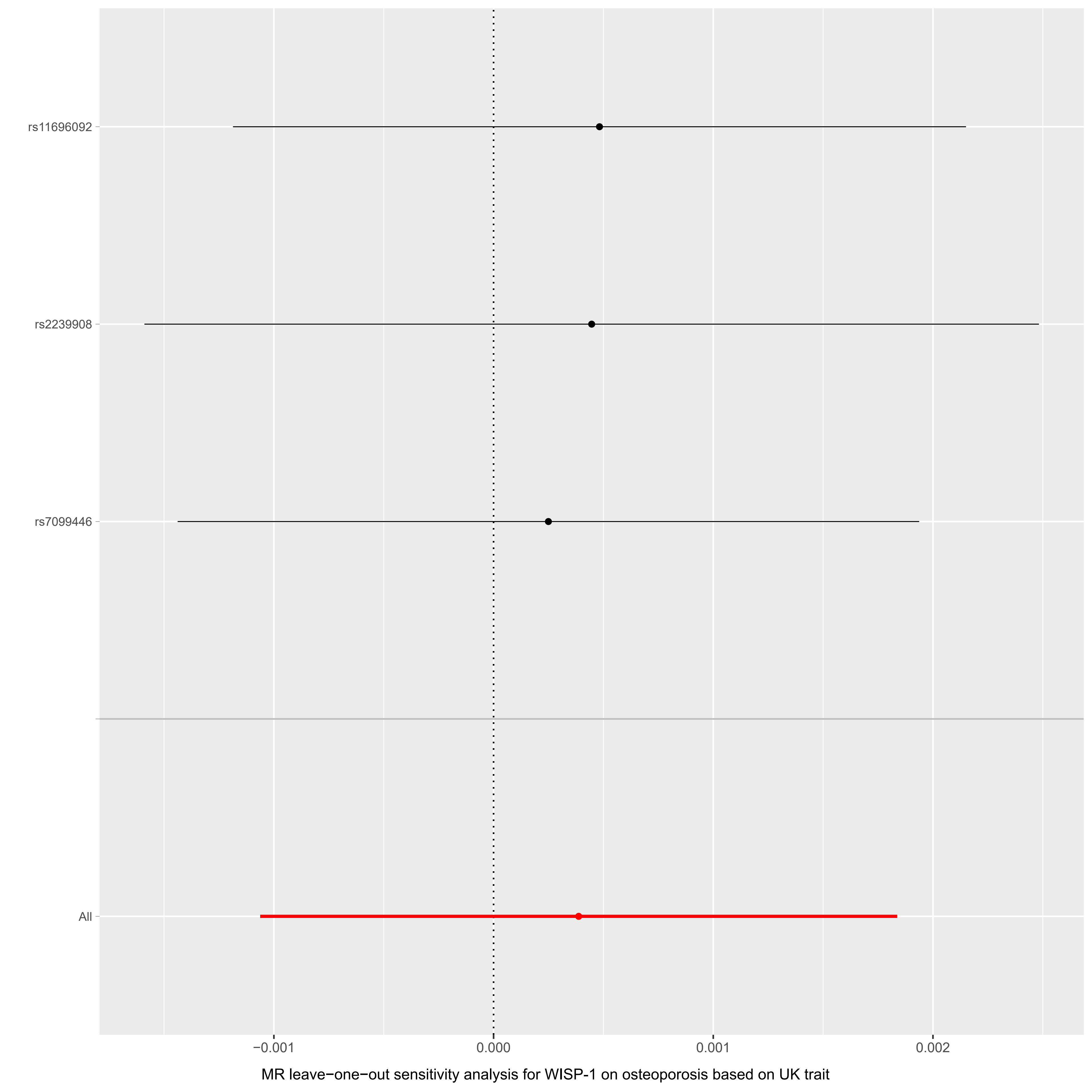
rs11696092
rs2239908
rs7099446
All
−0.001
0.000
0.001
0.002
MR leave−one−out sensitivity analysis for WISP-1 on osteoporosis based on UK trait

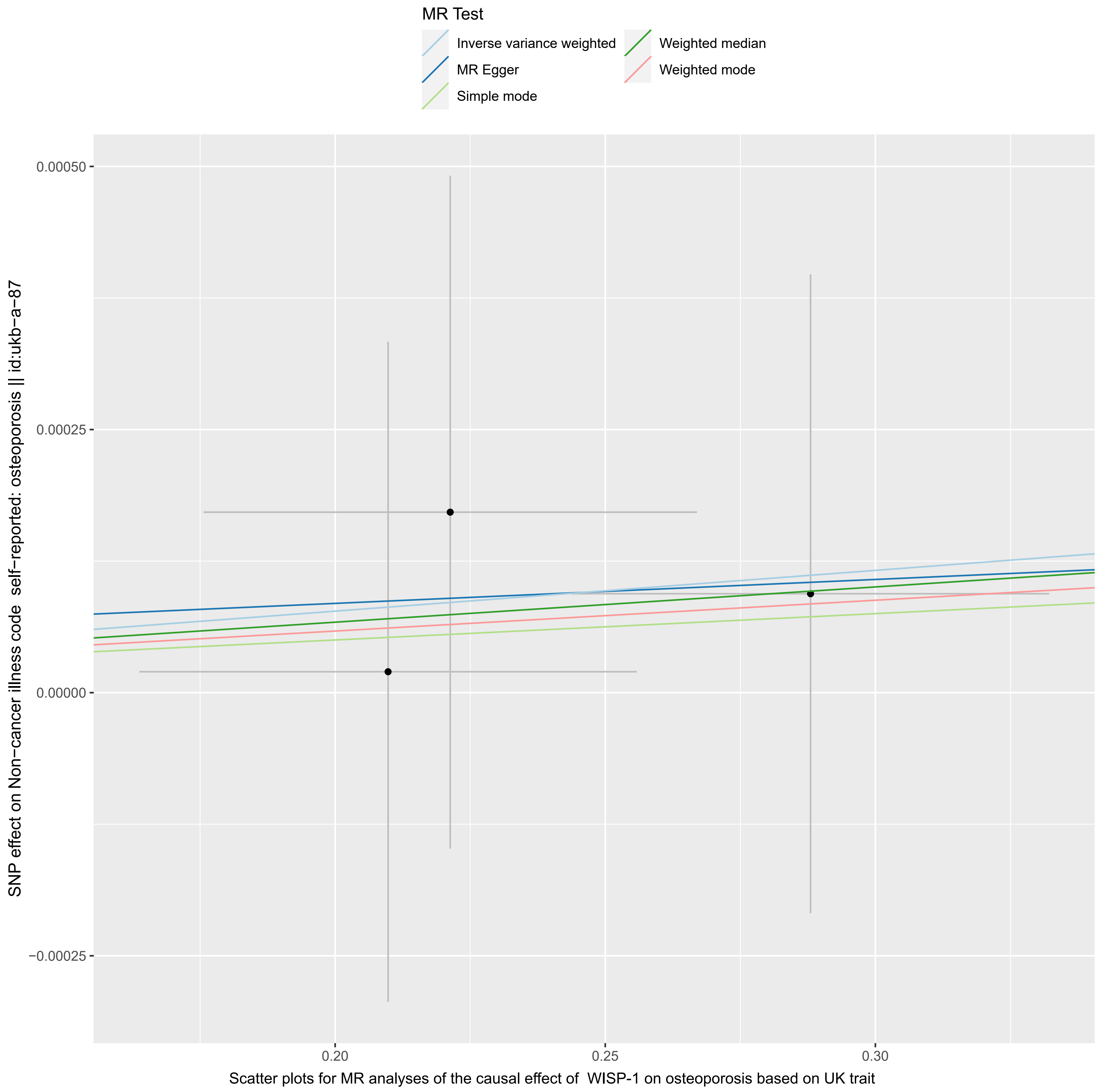

MR Test
Inverse variance weighted
MR Egger
Simple mode
Weighted median
Weighted mode
0.00050
0.00025
0.00000
−0.00025
0.20
0.25
0.30
SNP effect on Non−cancer illness code self−reported: osteoporosis || id:ukb−a−87
Scatter plots for MR analyses of the causal effect of WISP-1 on osteoporosis based on UK trait

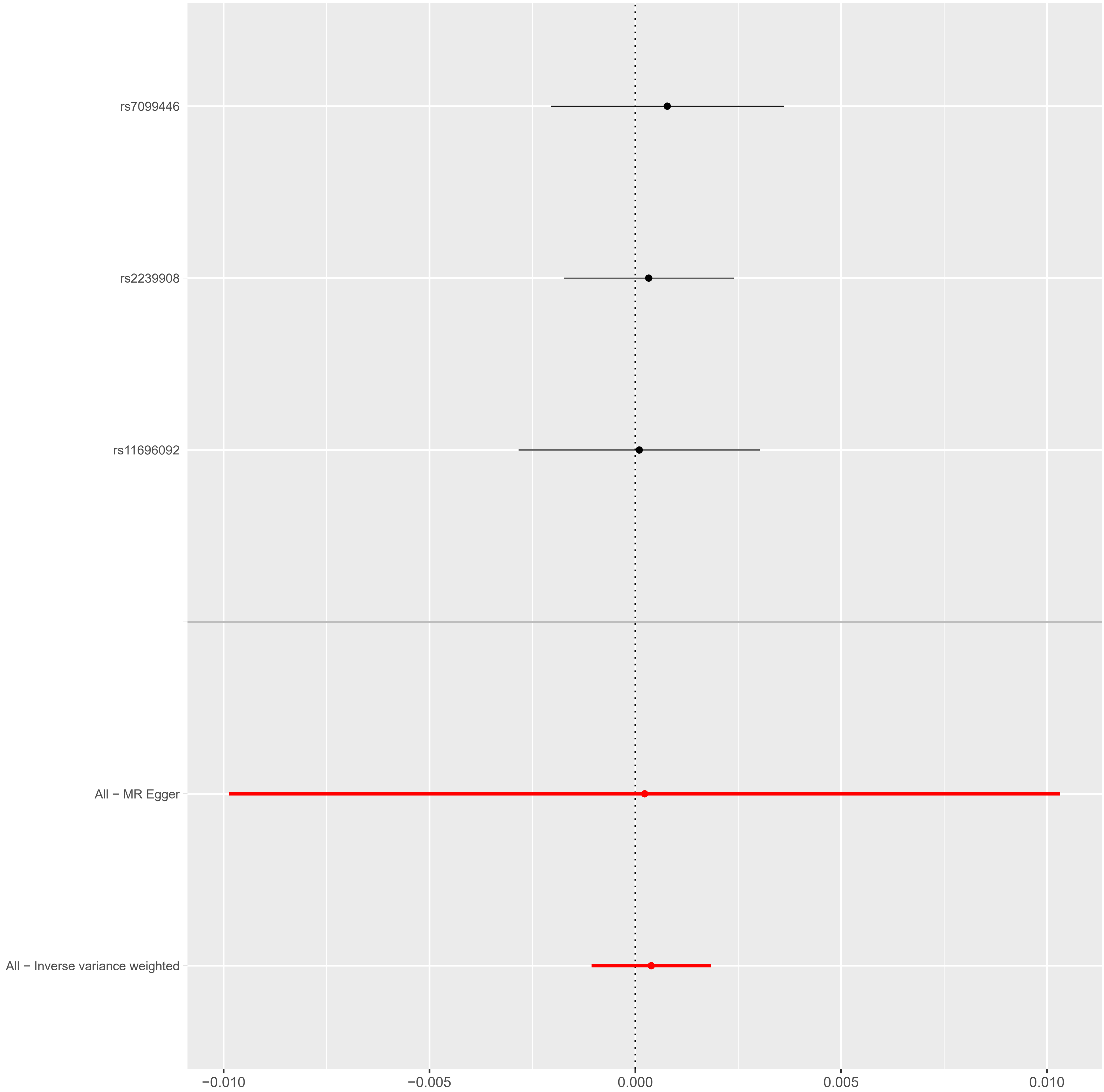

Forest plots for MR analyses of the causal effect of WISP-1 using each SNP singly on osteoporosis based on UK trait

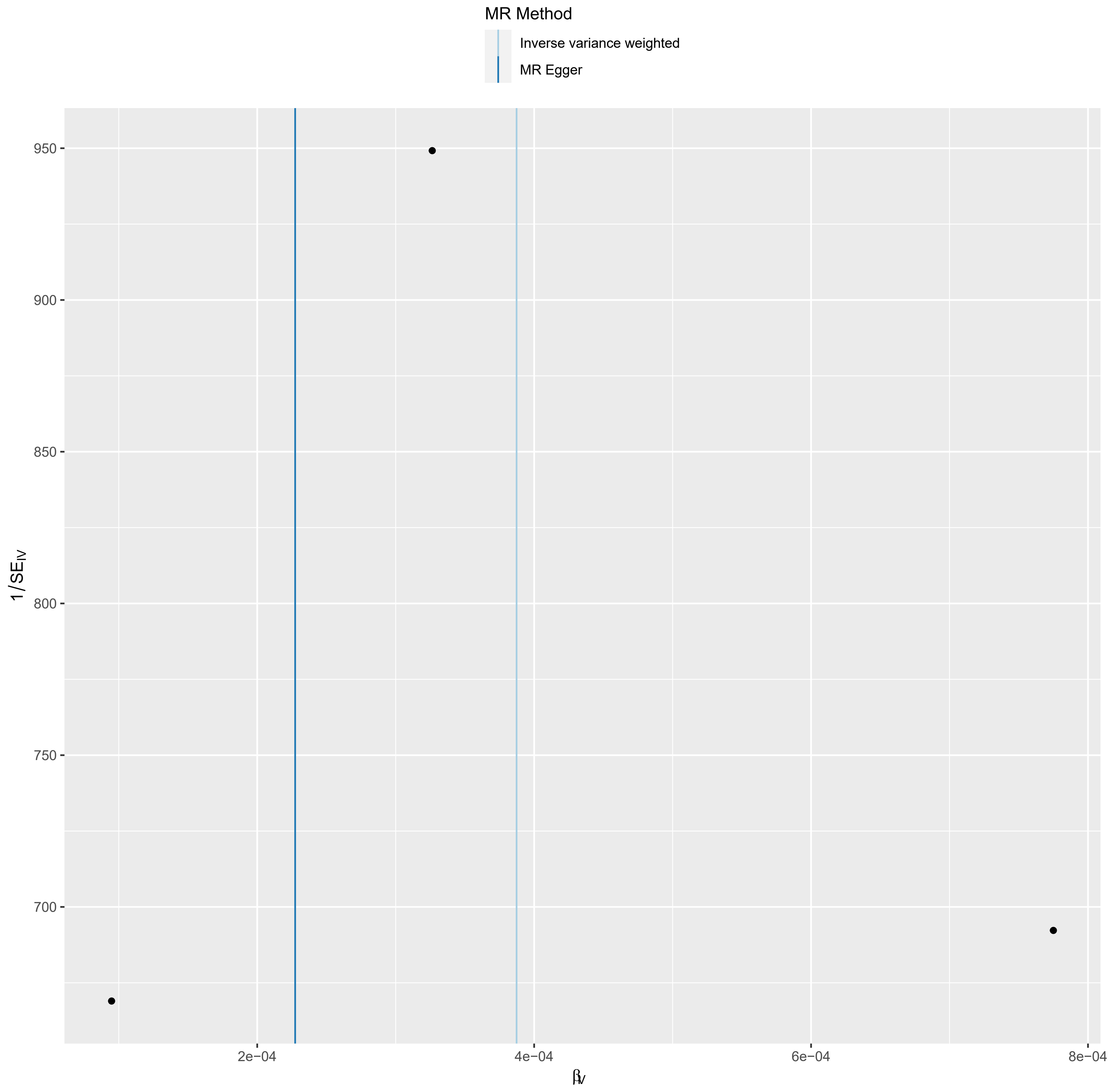

Funnal plots to assess heterogeneity for WISP-1 using all SNPs with the MR Egger and IVW methods

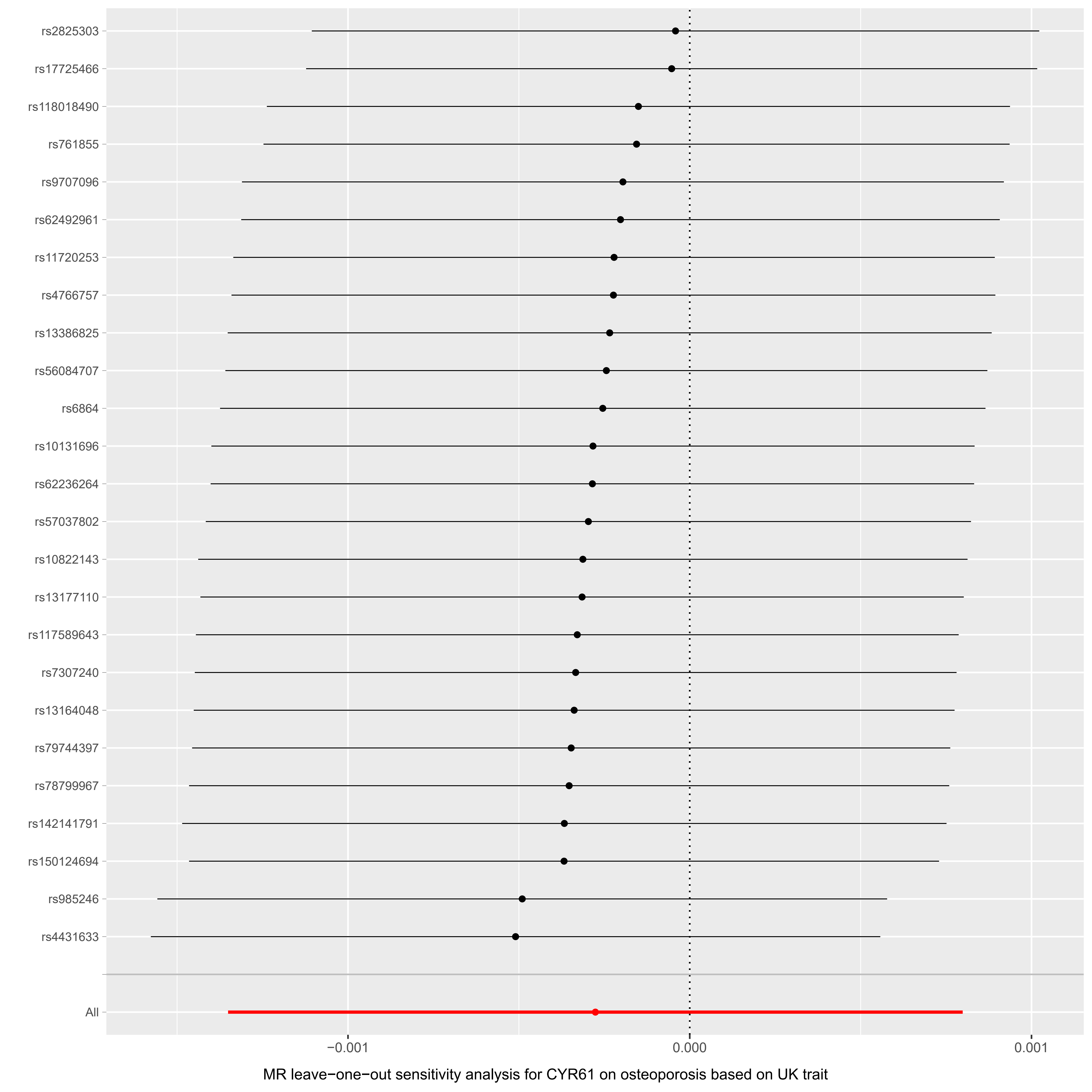
rs2825303
rs17725466
rs118018490
rs761855
rs9707096
rs62492961
rs11720253
rs4766757
rs13386825
rs56084707
rs6864
rs10131696
rs62236264
rs57037802
rs10822143
rs13177110
rs117589643
rs7307240
rs13164048
rs79744397
rs78799967
rs142141791
rs150124694
rs985246
rs4431633
All
−0.001
0.000
0.001
MR leave−one−out sensitivity analysis for CYR61 on osteoporosis based on UK trait

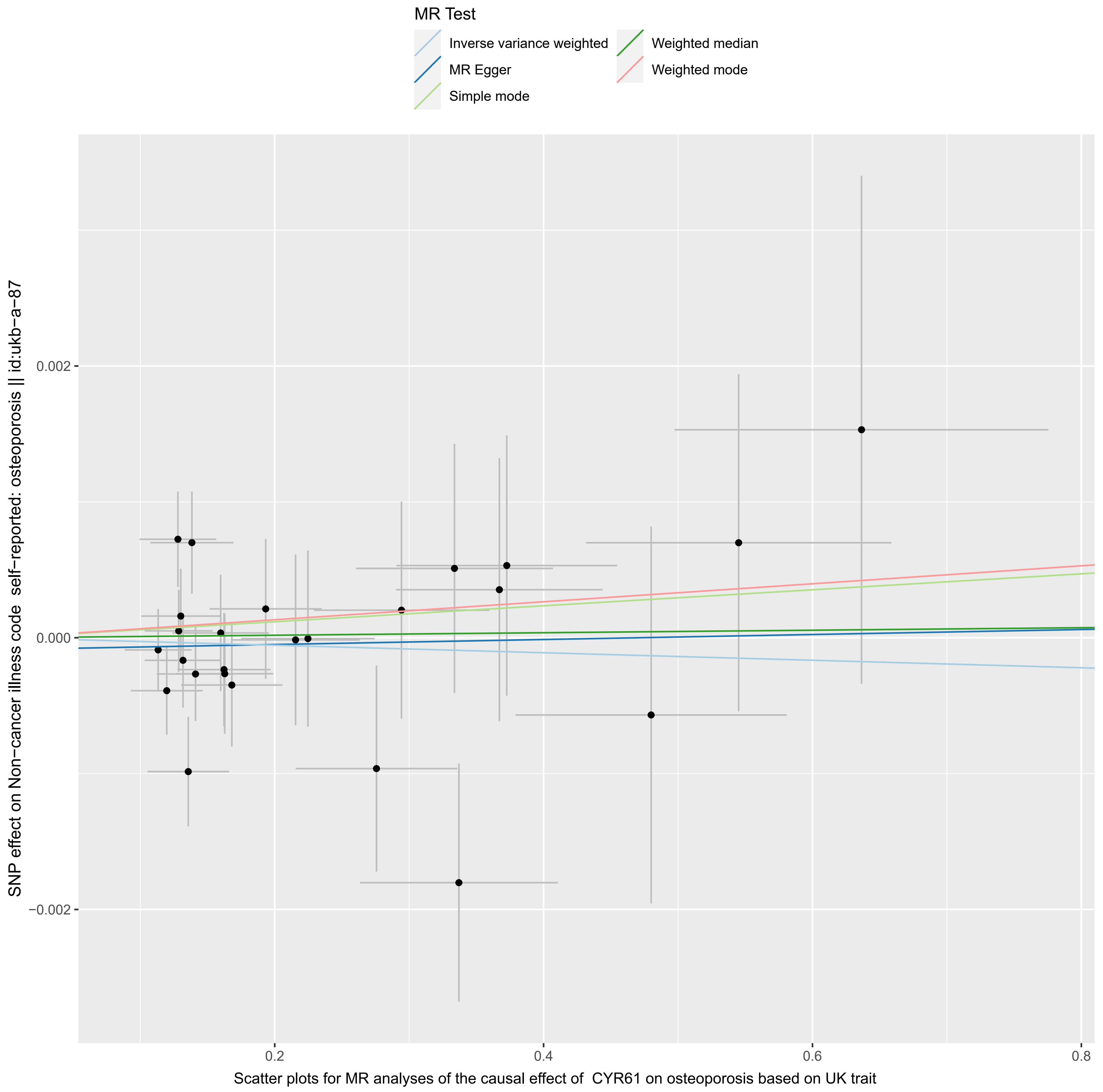
MR Test
Inverse variance weighted
MR Egger
Simple mode
Weighted median
Weighted mode
SNP effect on Non−cancer illness code self−reported: osteoporosis || id:ukb−a−87
0.002
0.000
−0.002
0.2
0.4
0.6
0.8
Scatter plots for MR analyses of the causal effect of CYR61 on osteoporosis based on UK trait

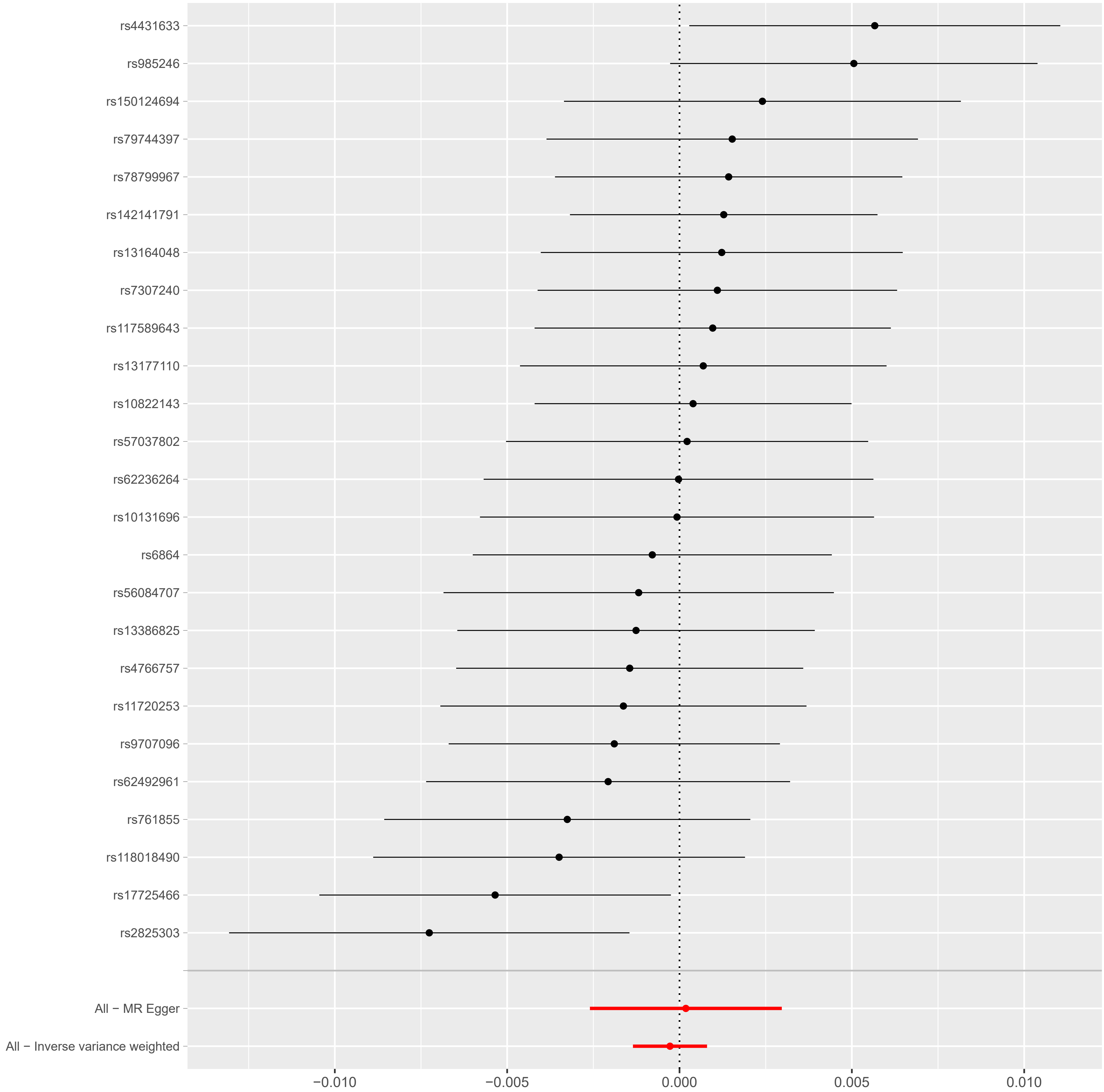

Forest plots for MR analyses of the causal effect of CYR61 using each SNP singly on osteoporosis based on UK trait

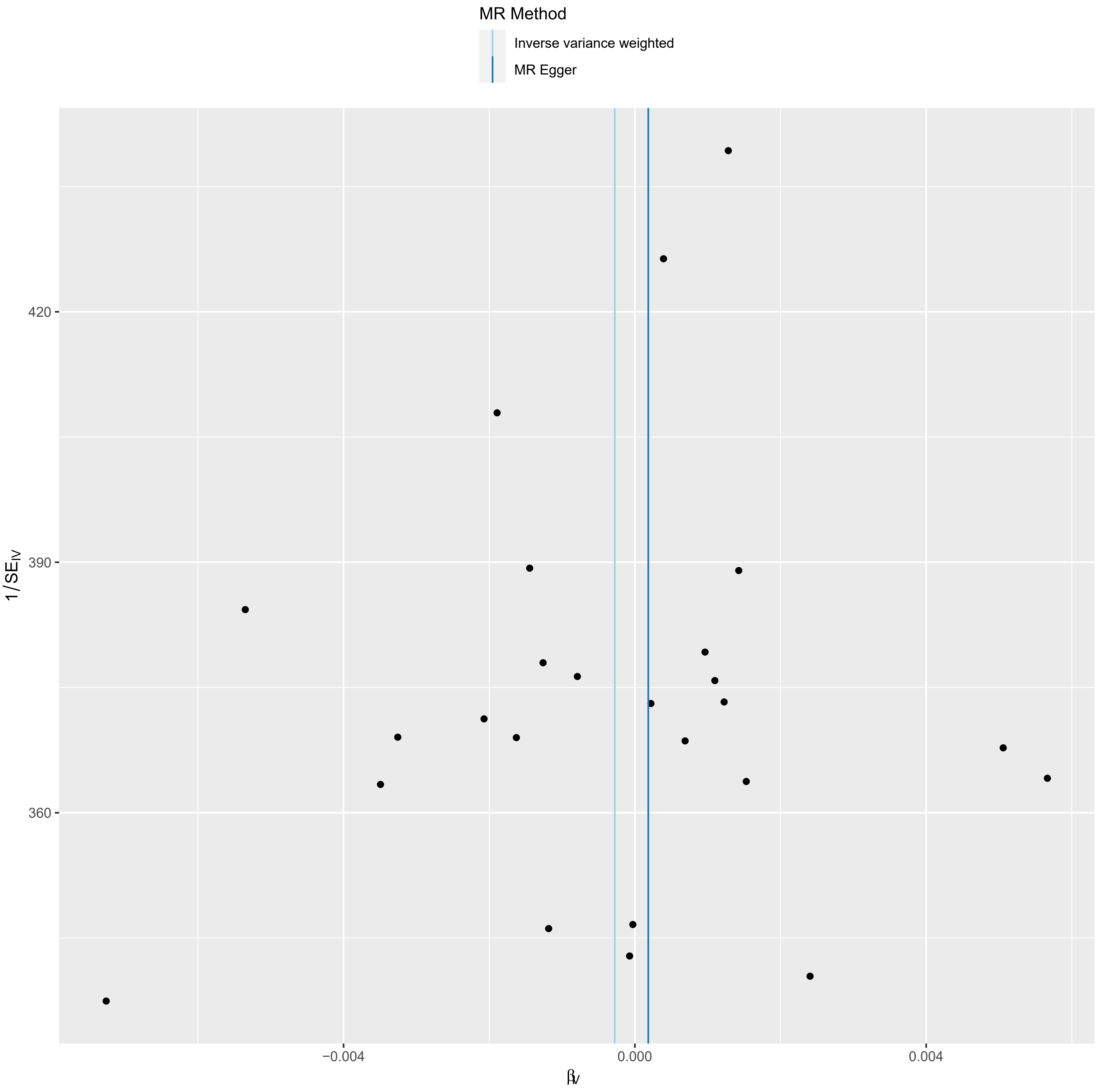

Funnal plots to assess heterogeneity for CYR61 using all SNPs with the MR Egger and IVW methods
