# Supplementary material for: Controversial causal association between IGF family members and osteoporosis: a Mendelian randomization study between UK and FinnGen biobanks
Source: Front Endocrinol (Lausanne). 2024 Jan 8;14:1332803. doi: 10.3389/fendo.2023.1332803 (PMC10801076; doi:10.3389/fendo.2023.1332803)
Supplement: Supplementary file 3 — Funnel plots and Leave-one-out sensitive analysis of IGFs on osteoporosis based on FinnGen trait. [file DataSheet_3.pdf]

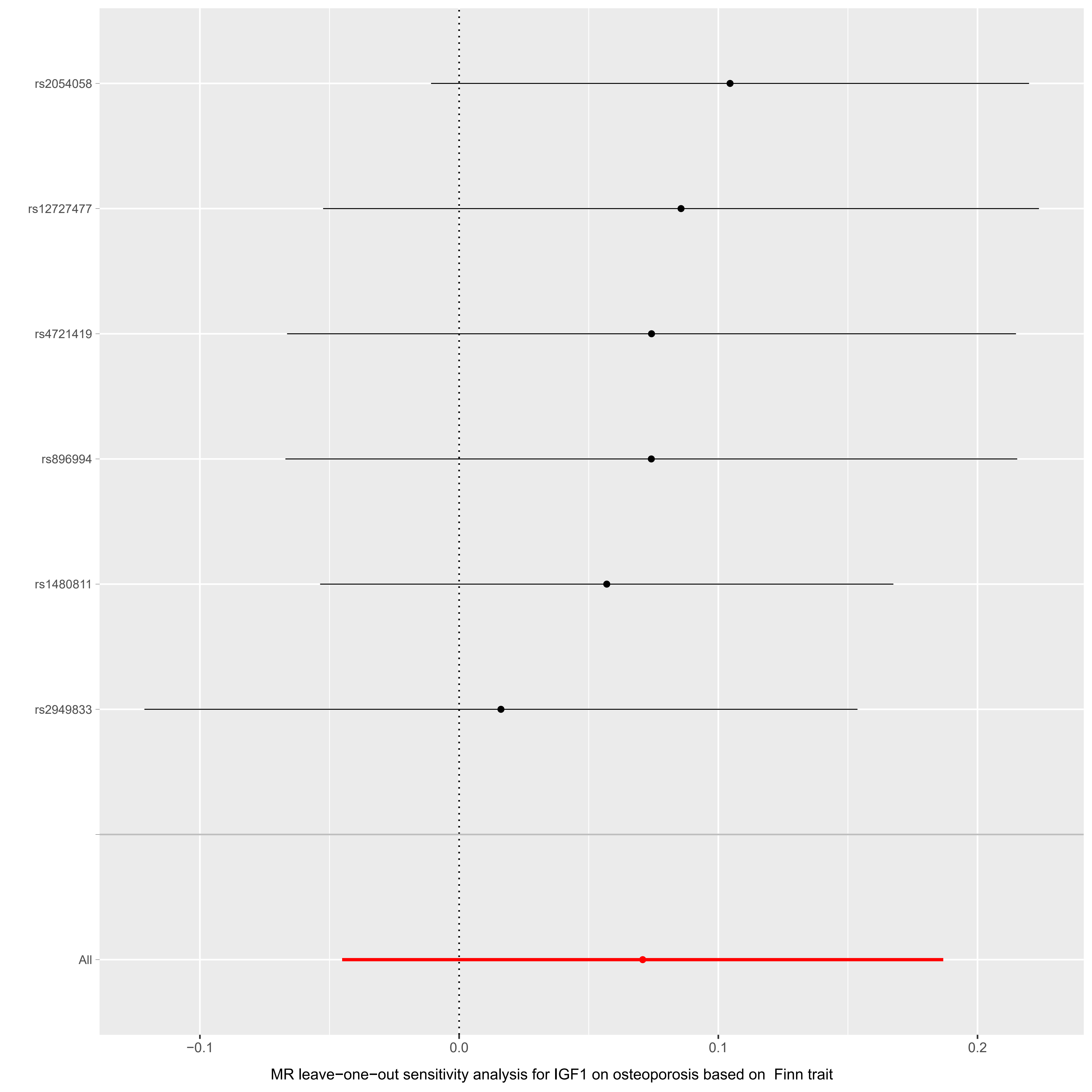

rs2054058
rs12727477
rs4721419
rs896994
rs1480811
rs2949833
All
−0.1
0.0
0.1
0.2
MR leave−one−out sensitivity analysis for IGF1 on osteoporosis based on Finn trait

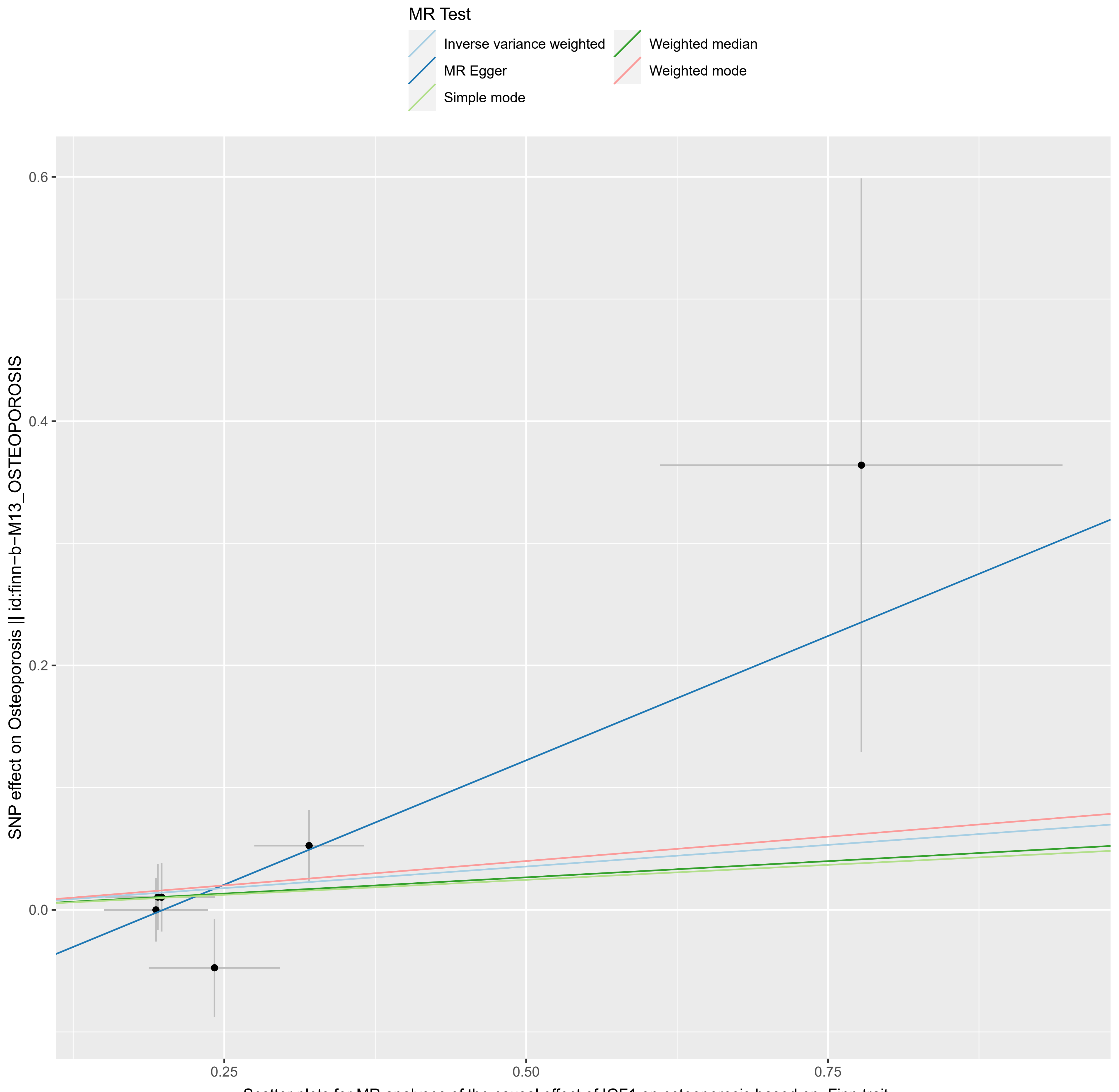

Scatter plots for MR analyses of the causal effect of IGF1 on osteoporosis based on Finn trait

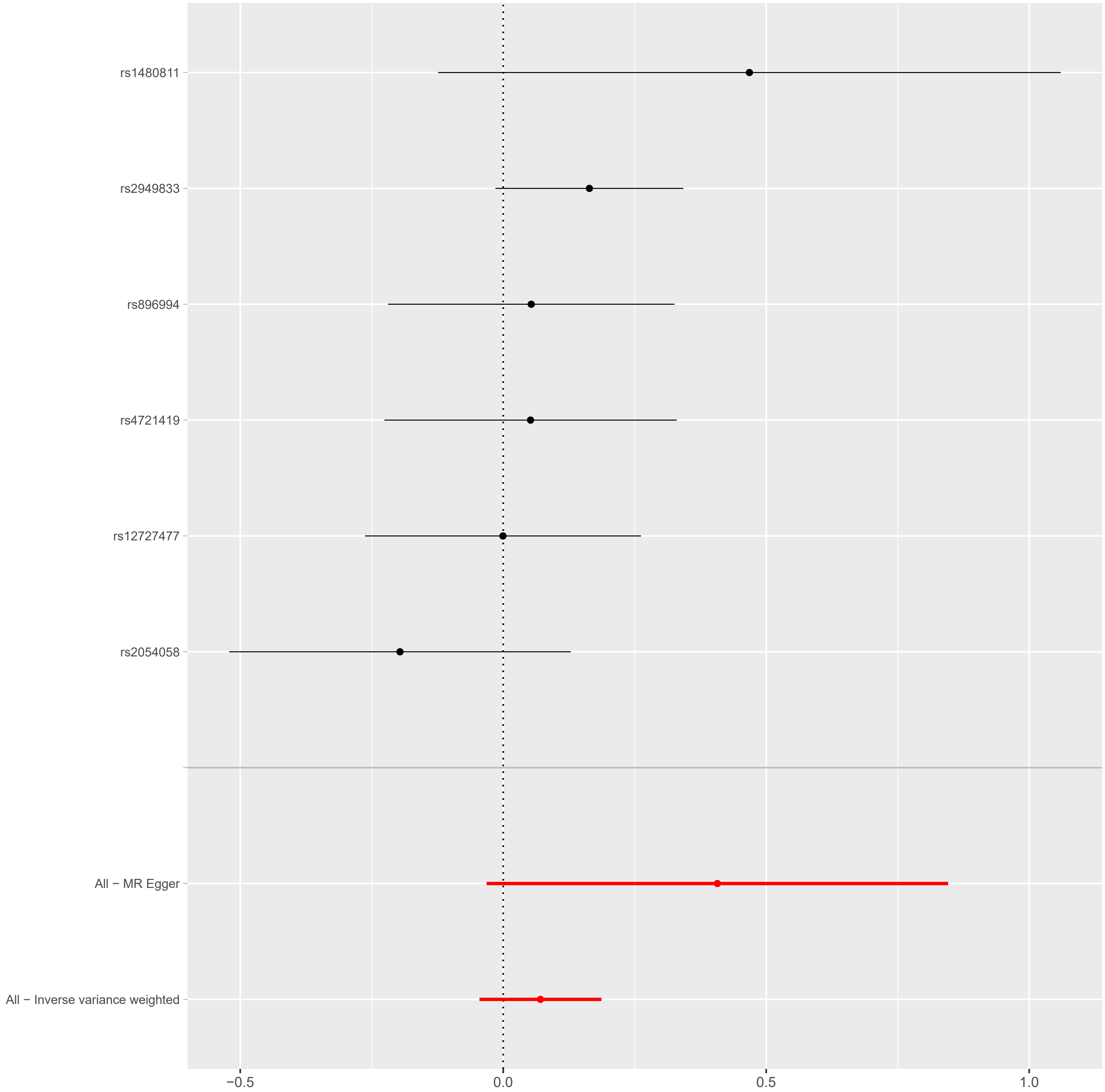

Forest plots for MR analyses of the causal effect of IGF1 using each SNP singly on osteoporosis based on Finn trait

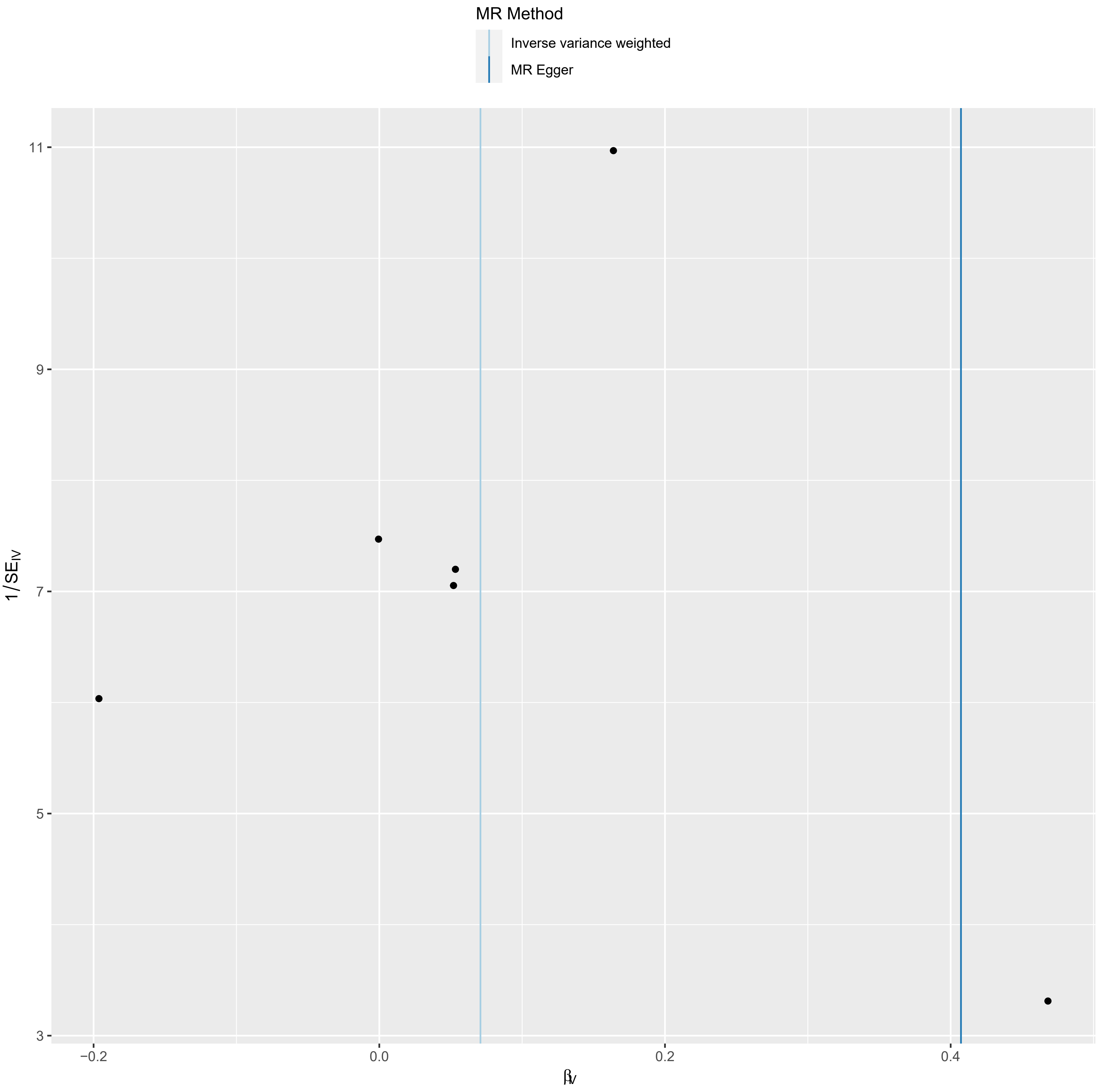

Funnal plots to assess heterogeneity for IGF1 using all SNPs with the MR Egger and IVW methods

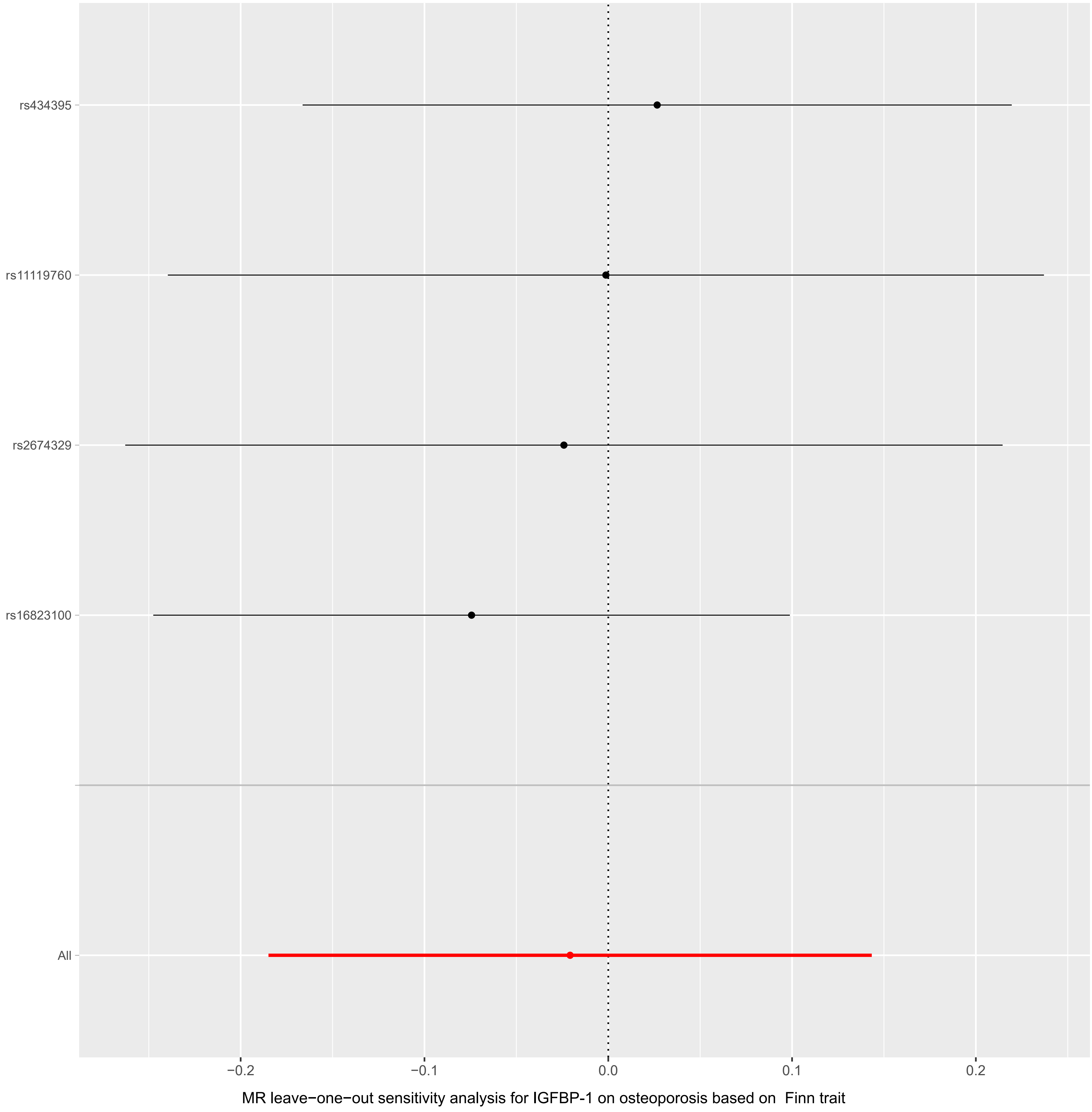

rs434395
rs11119760
rs2674329
rs16823100
All
−0.2
−0.1
0.0
0.1
0.2
MR leave−one−out sensitivity analysis for IGFBP-1 on osteoporosis based on Finn trait

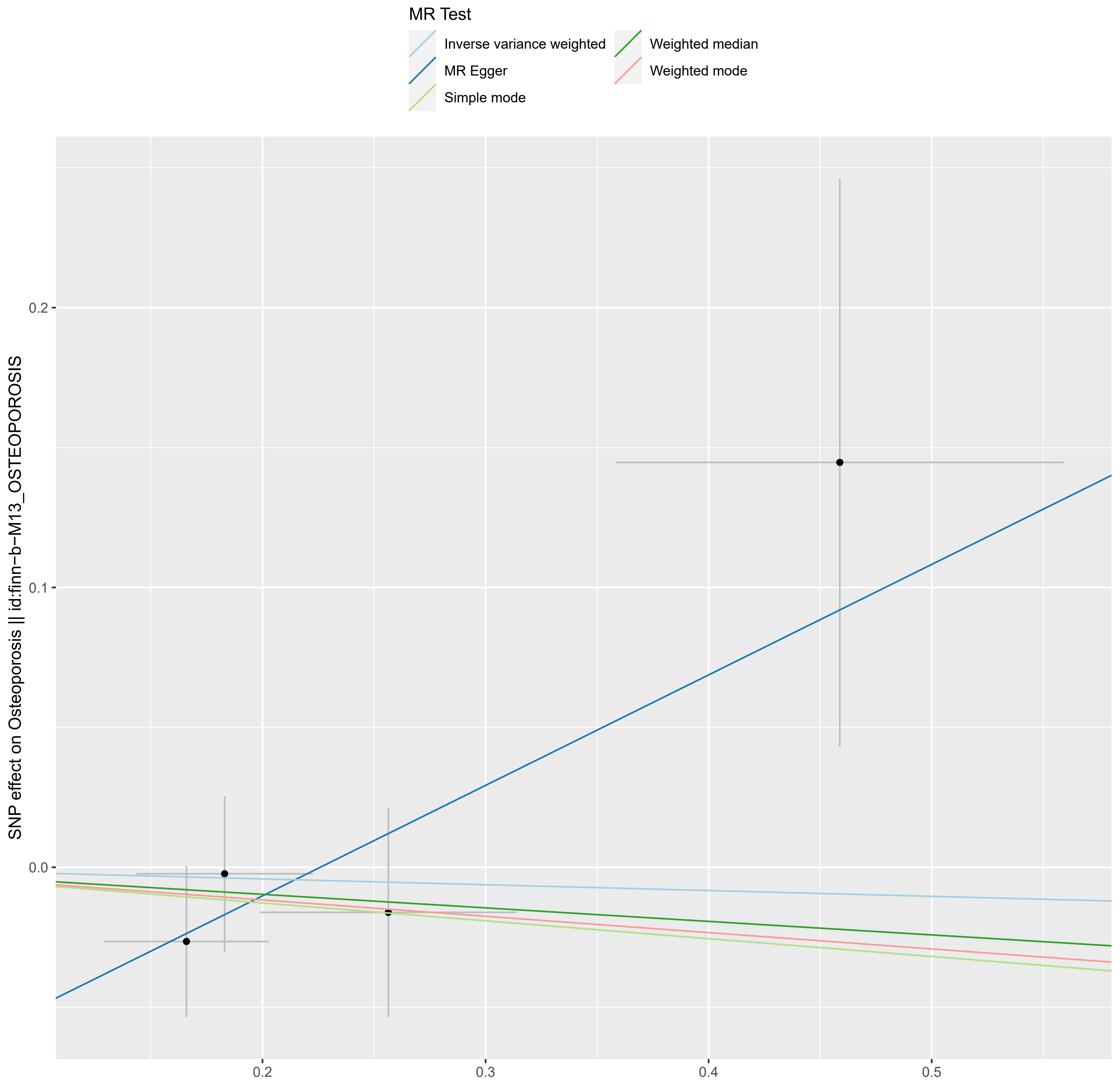

Scatter plots for MR analyses of the causal effect of IGFBP-1 on osteoporosis based on Finn trait

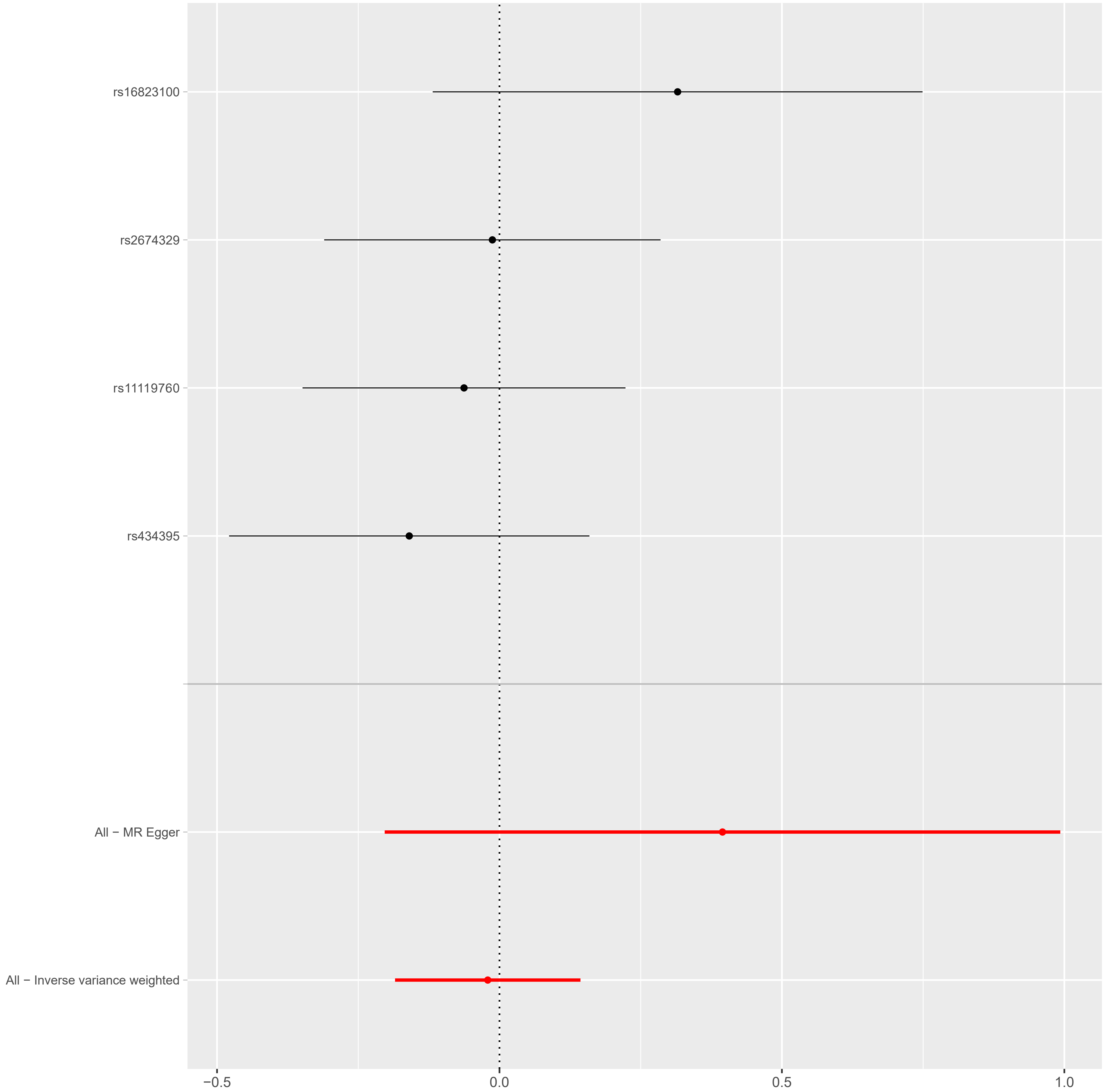

Forest plots for MR analyses of the causal effect of IGFBP-1 using each SNP singly on osteoporosis based on Finn trait

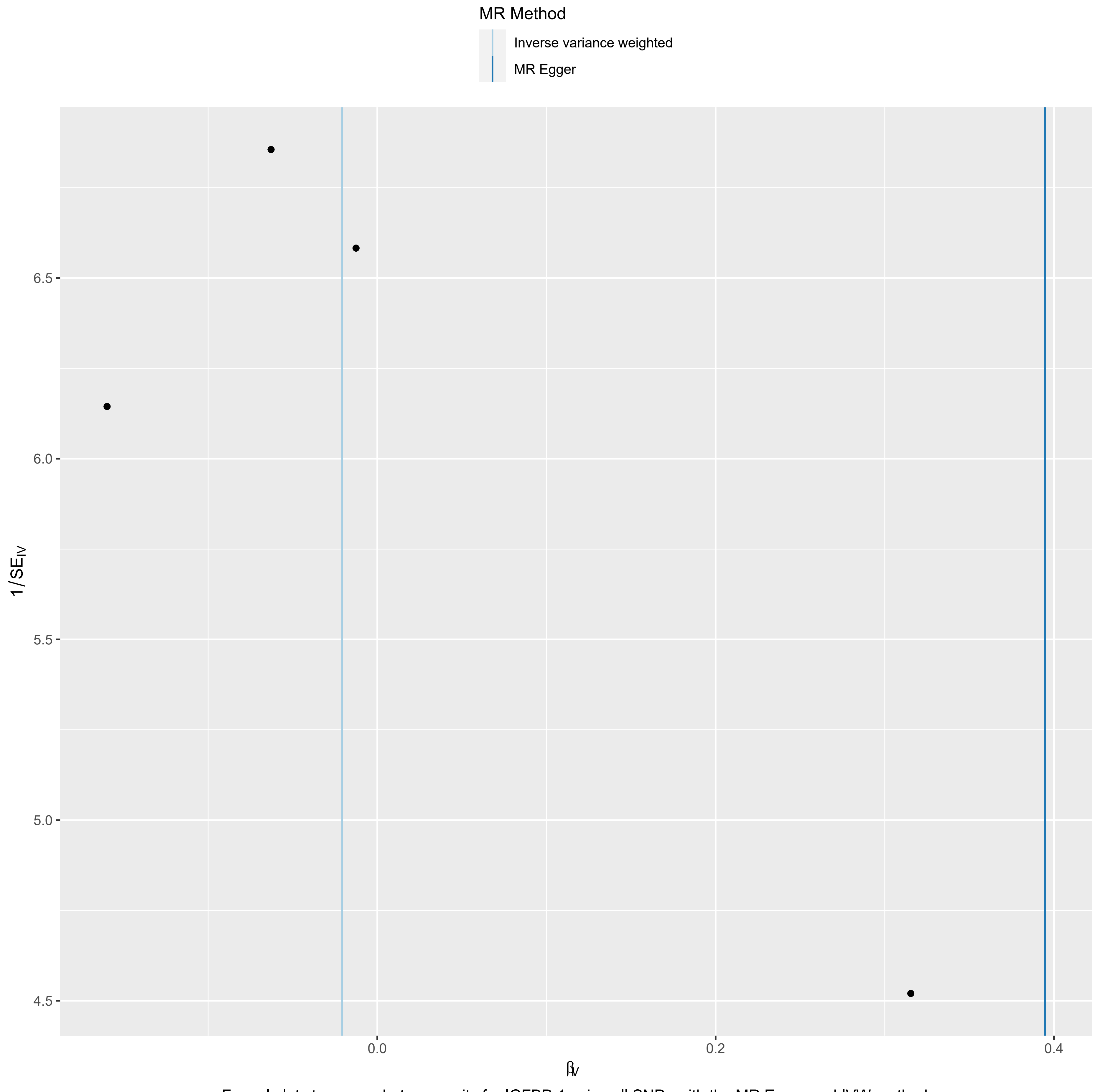

Funnal plots to assess heterogeneity for IGFBP-1 using all SNPs with the MR Egger and IVW methods

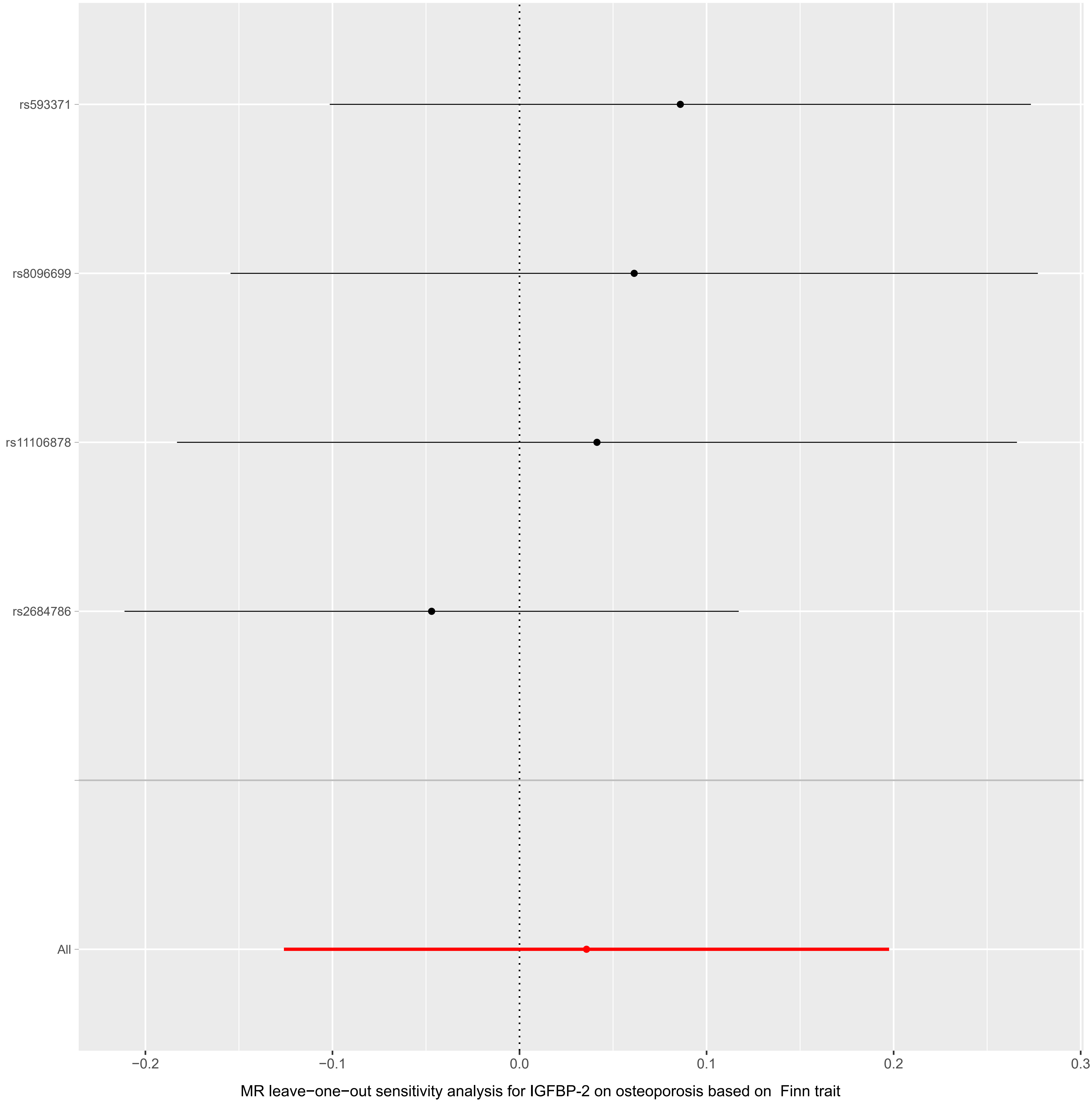
rs593371
rs8096699
rs11106878
rs2684786
All
−0.2
−0.1
0.0
0.1
0.2
0.3
MR leave−one−out sensitivity analysis for IGFBP-2 on osteoporosis based on Finn trait

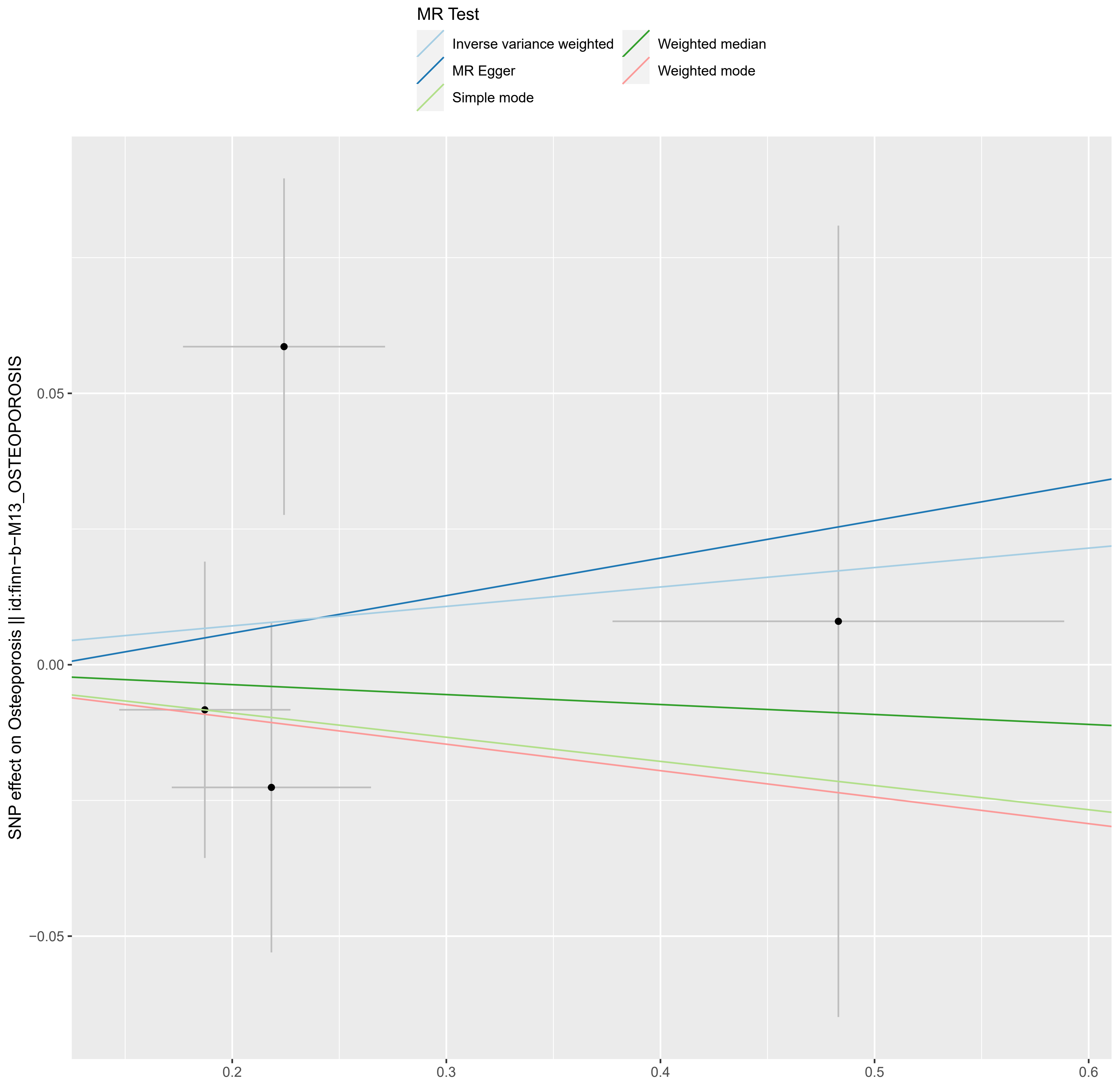

Scatter plots for MR analyses of the causal effect of IGFBP-2 on osteoporosis based on Finn trait

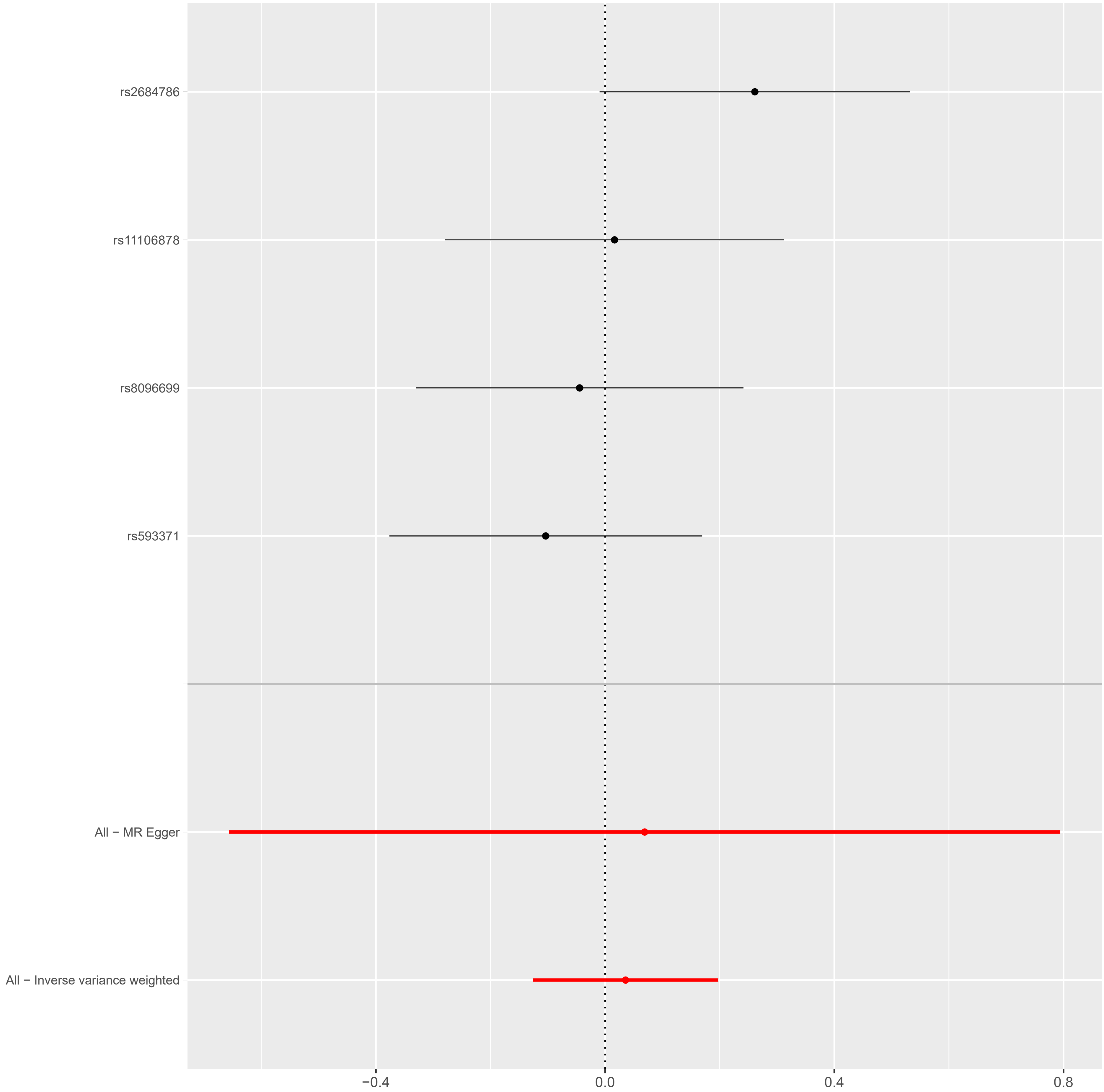

Forest plots for MR analyses of the causal effect of IGFBP-2 using each SNP singly on osteoporosis based on Finn trait

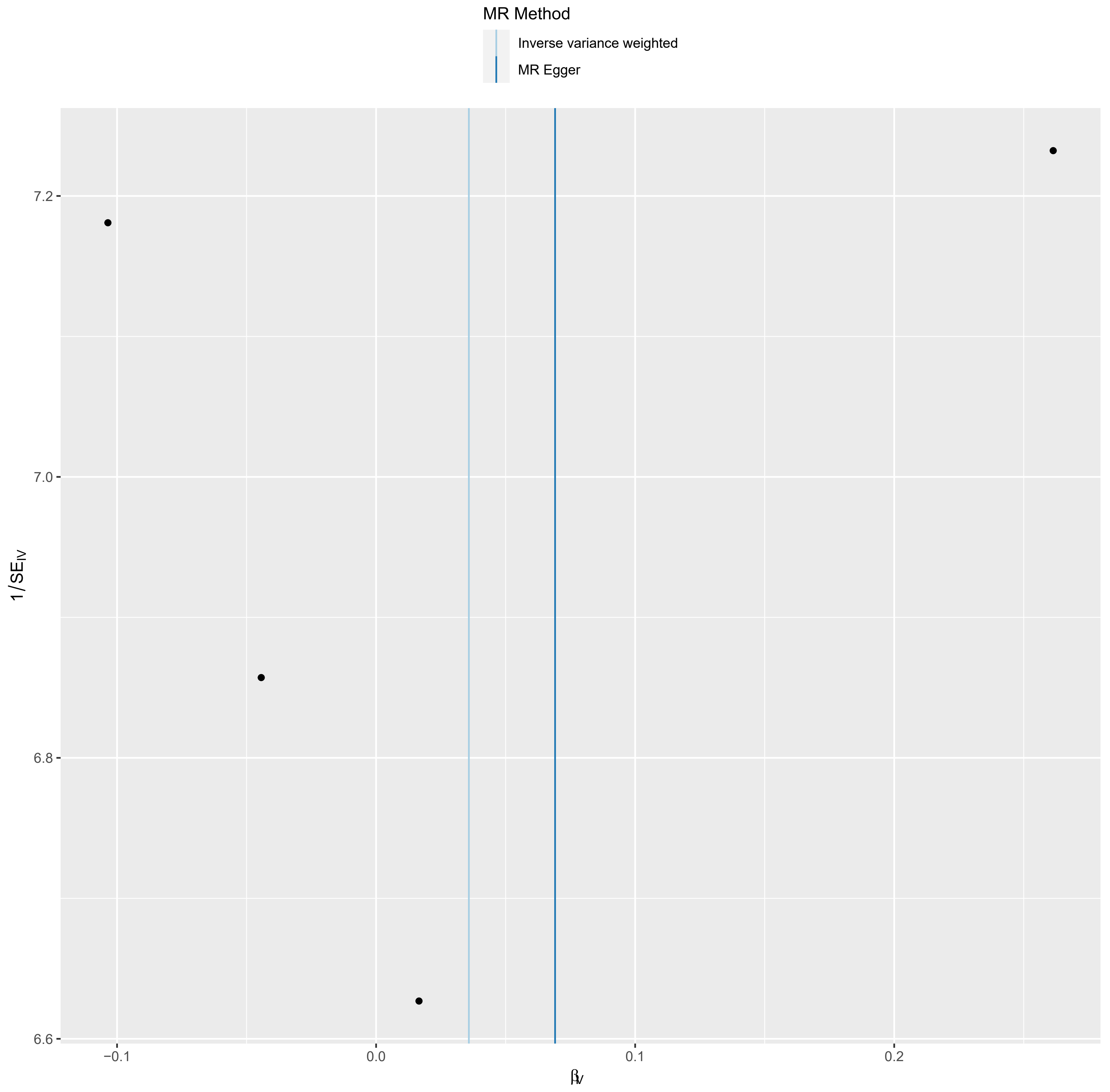

Funnal plots to assess heterogeneity for IGFBP-2 using all SNPs with the MR Egger and IVW methods

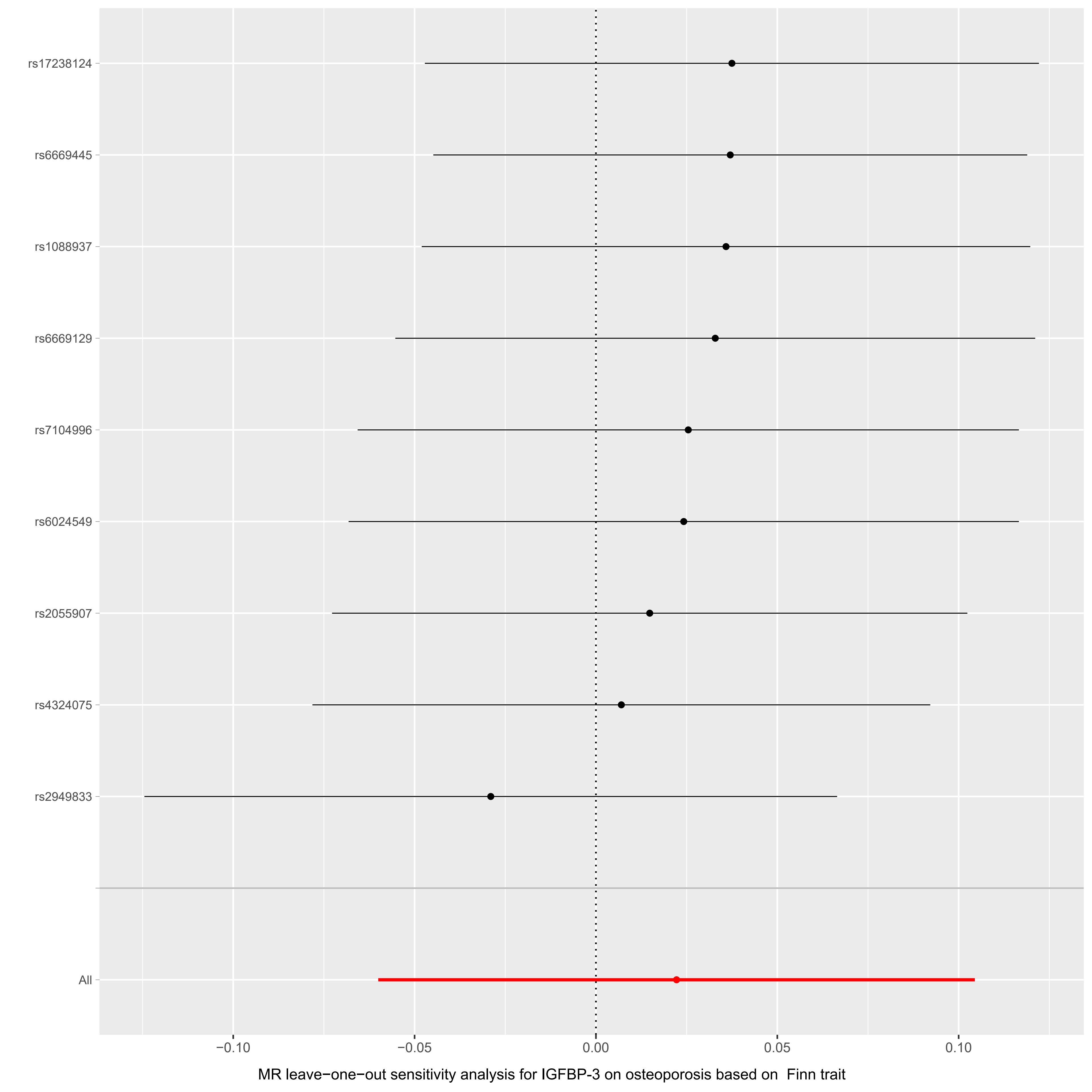
rs17238124
rs6669445
rs1088937
rs6669129
rs7104996
rs6024549
rs2055907
rs4324075
rs2949833
All
−0.10
−0.05
0.00
0.05
0.10
MR leave−one−out sensitivity analysis for IGFBP-3 on osteoporosis based on Finn trait

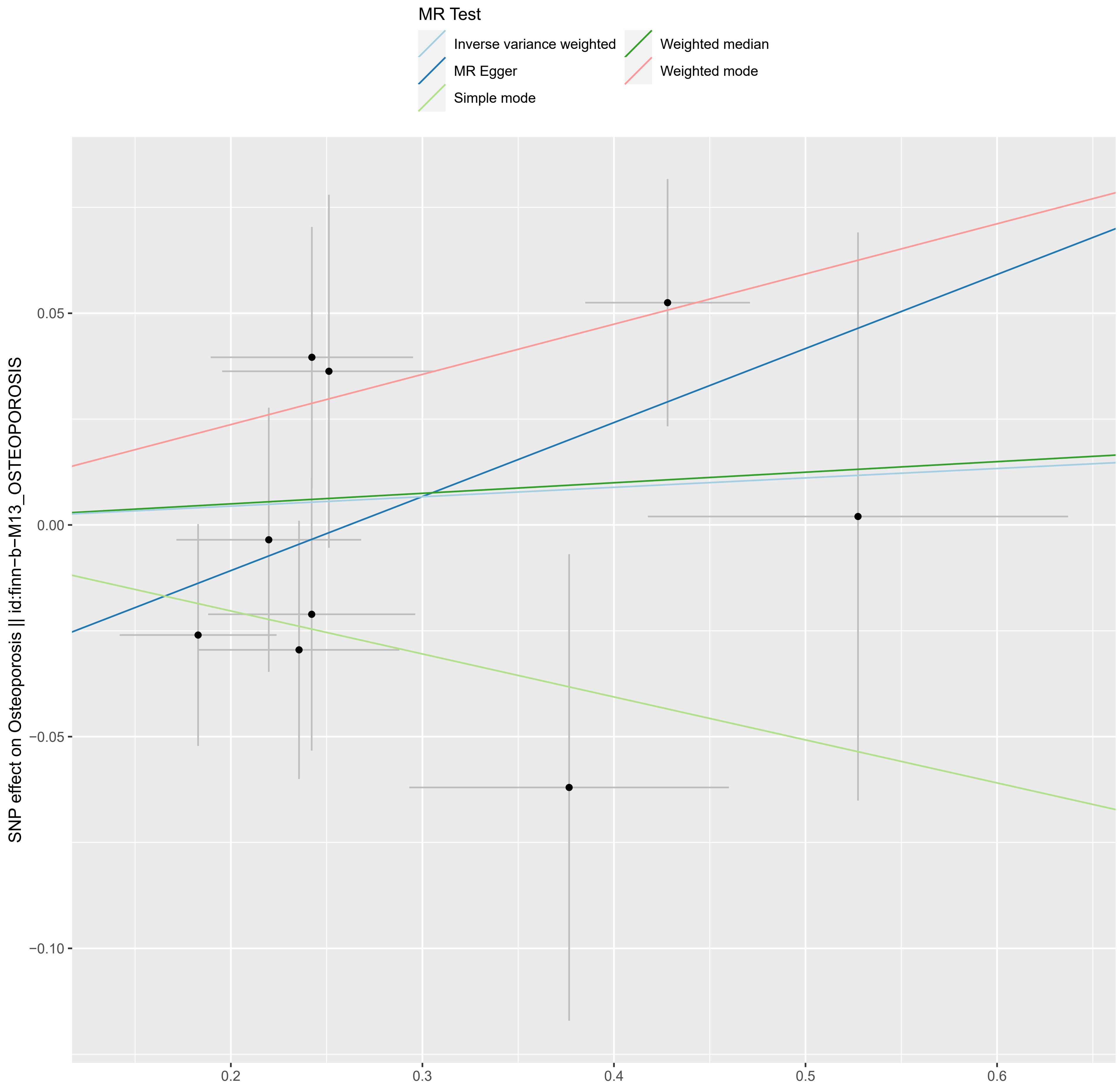

Scatter plots for MR analyses of the causal effect of IGFBP-3 on osteoporosis based on Finn trait

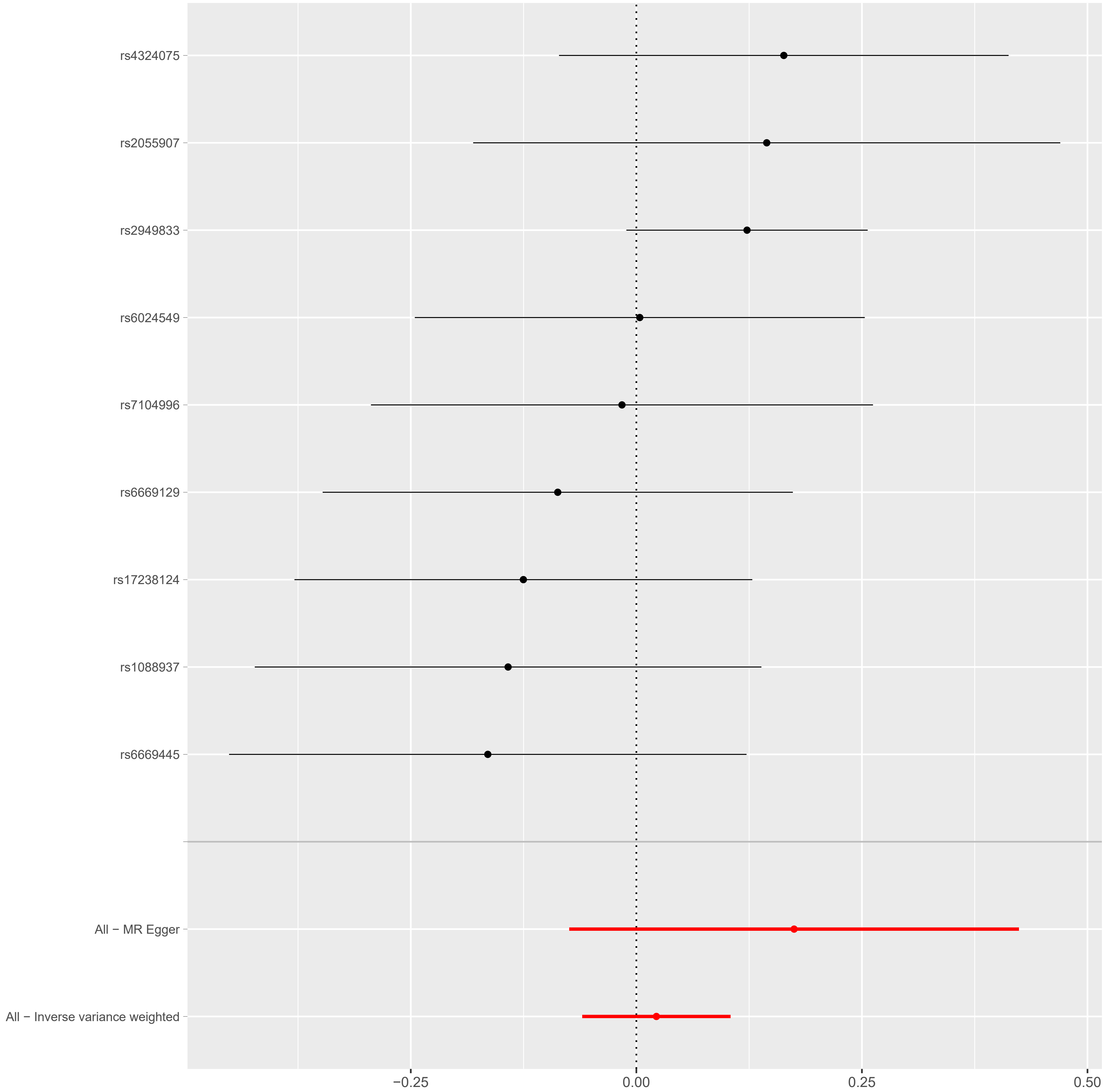

Forest plots for MR analyses of the causal effect of IGFBP-3 using each SNP singly on osteoporosis based on Finn trait

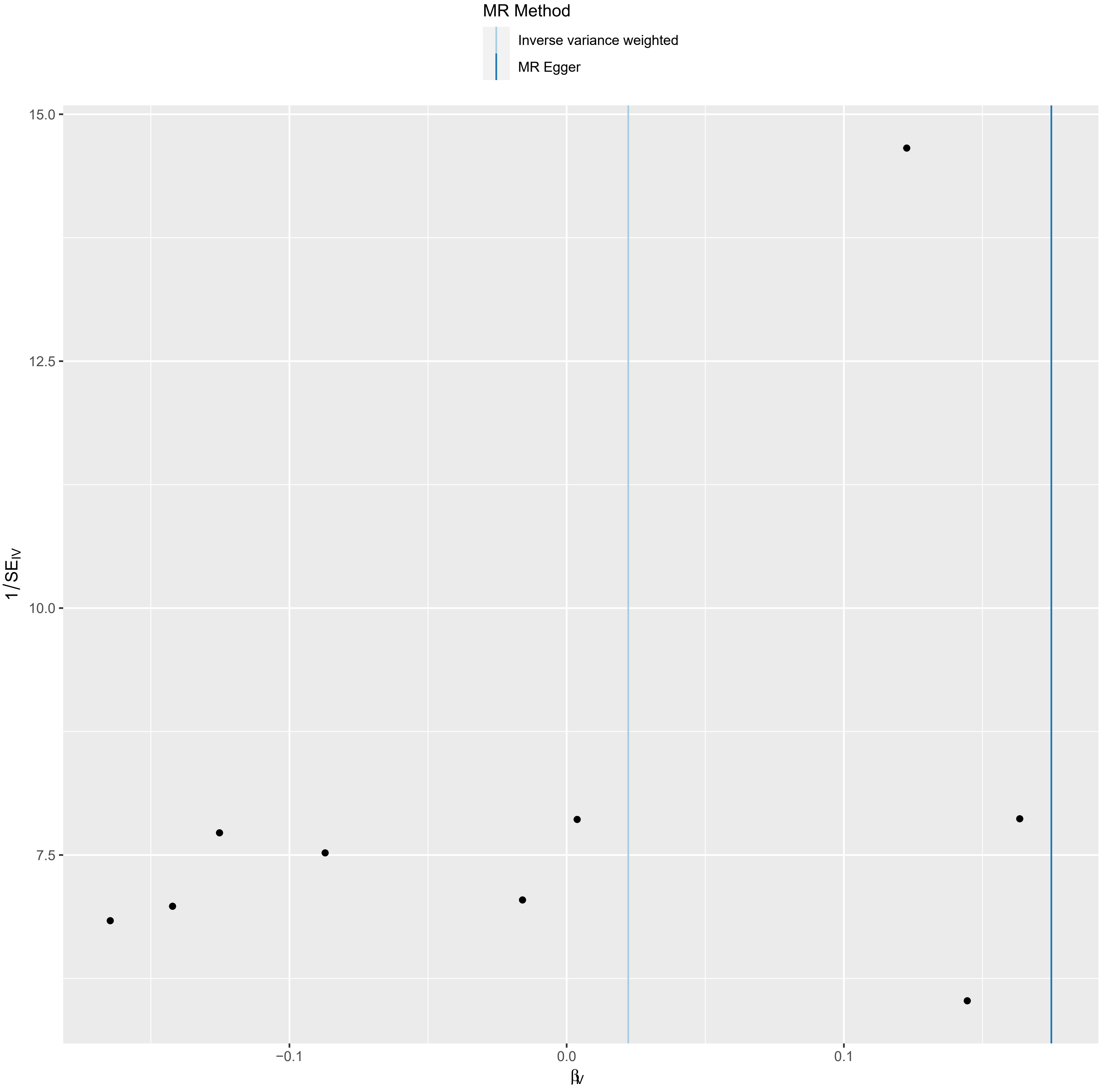

Funnal plots to assess heterogeneity for IGFBP-3 using all SNPs with the MR Egger and IVW methods

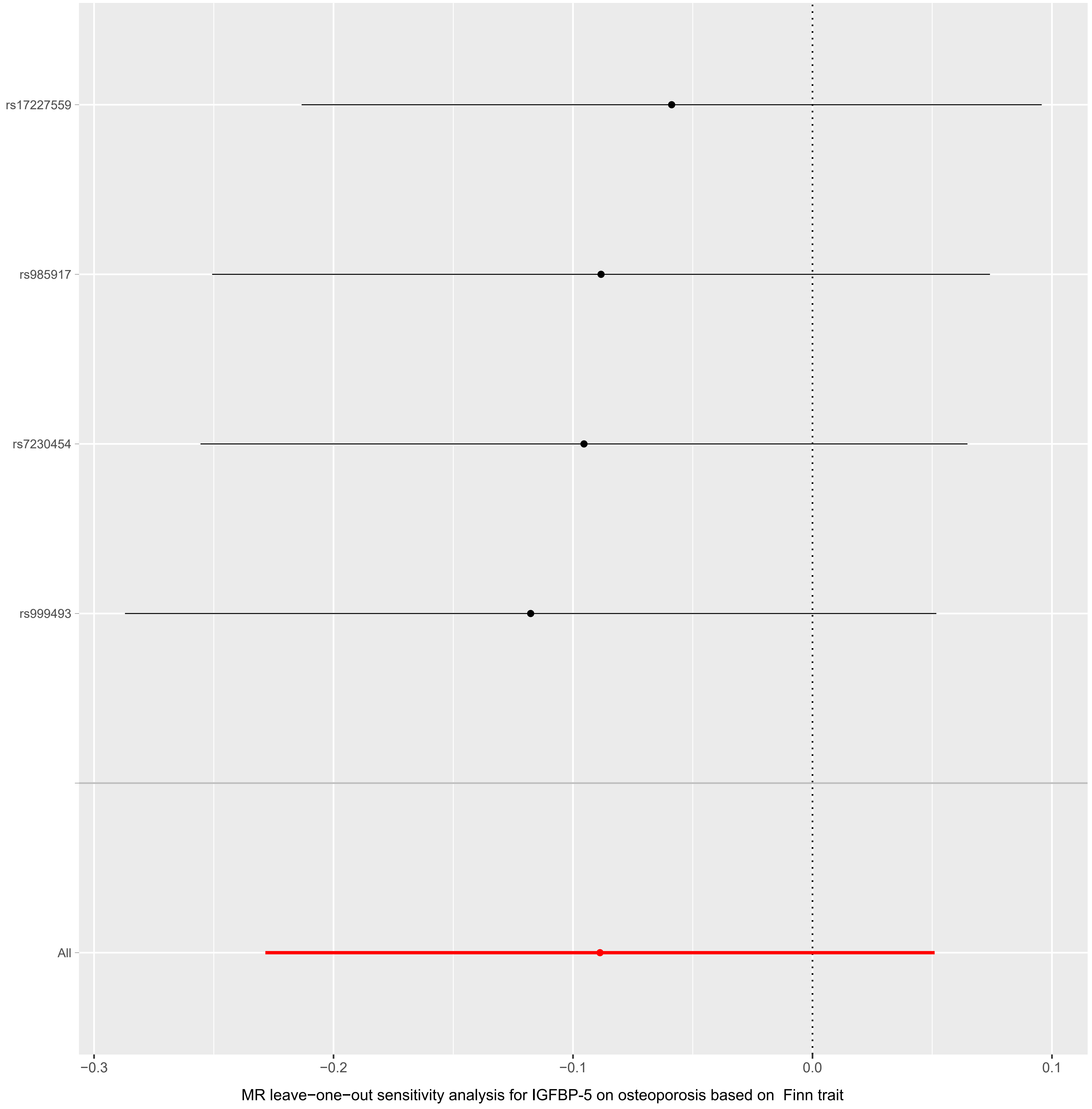
rs17227559
rs985917
rs7230454
rs999493
All
−0.3
−0.2
−0.1
0.0
0.1
MR leave−one−out sensitivity analysis for IGFBP-5 on osteoporosis based on Finn trait

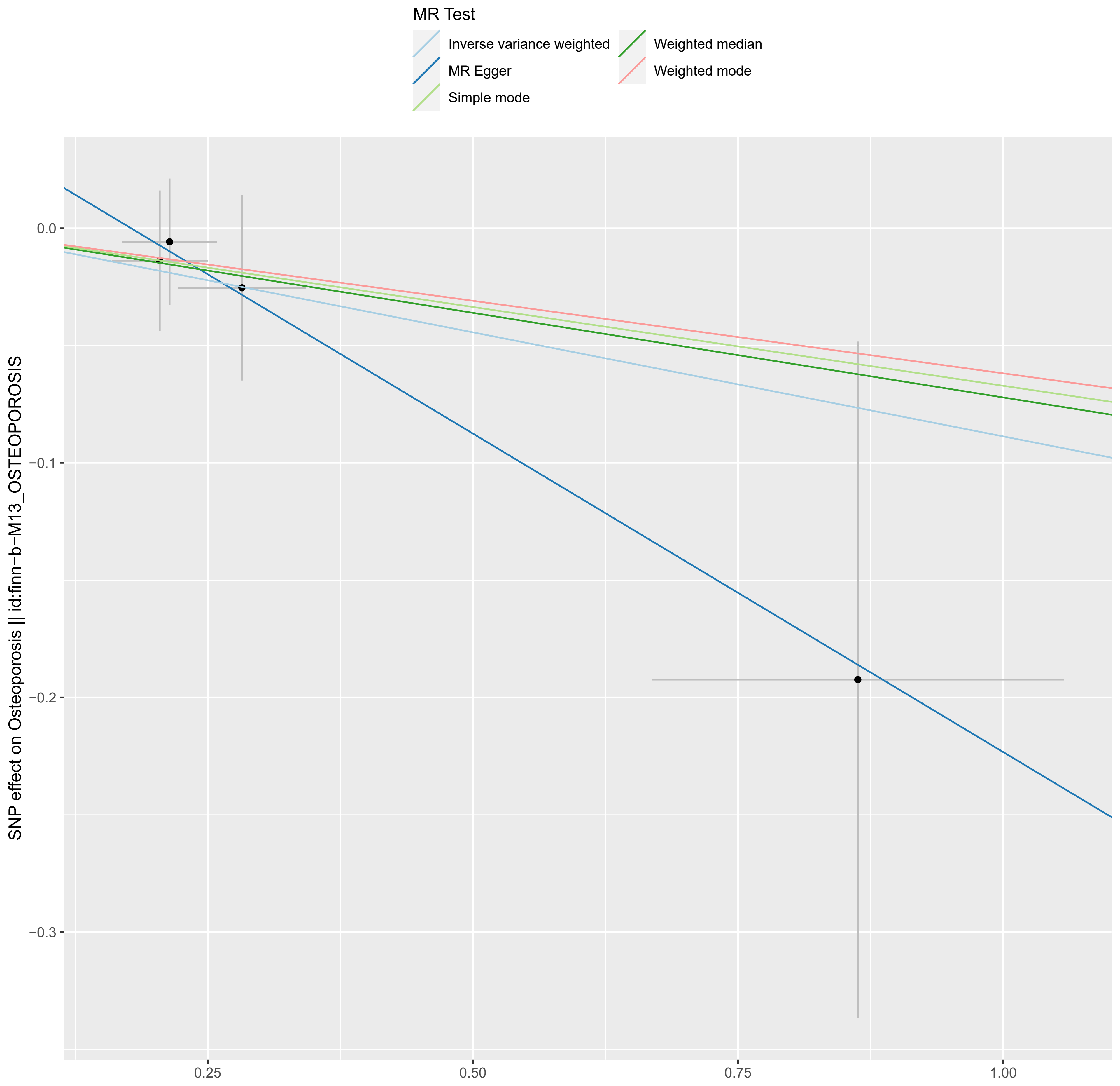

Scatter plots for MR analyses of the causal effect of IGFBP-5 on osteoporosis based on Finn trait

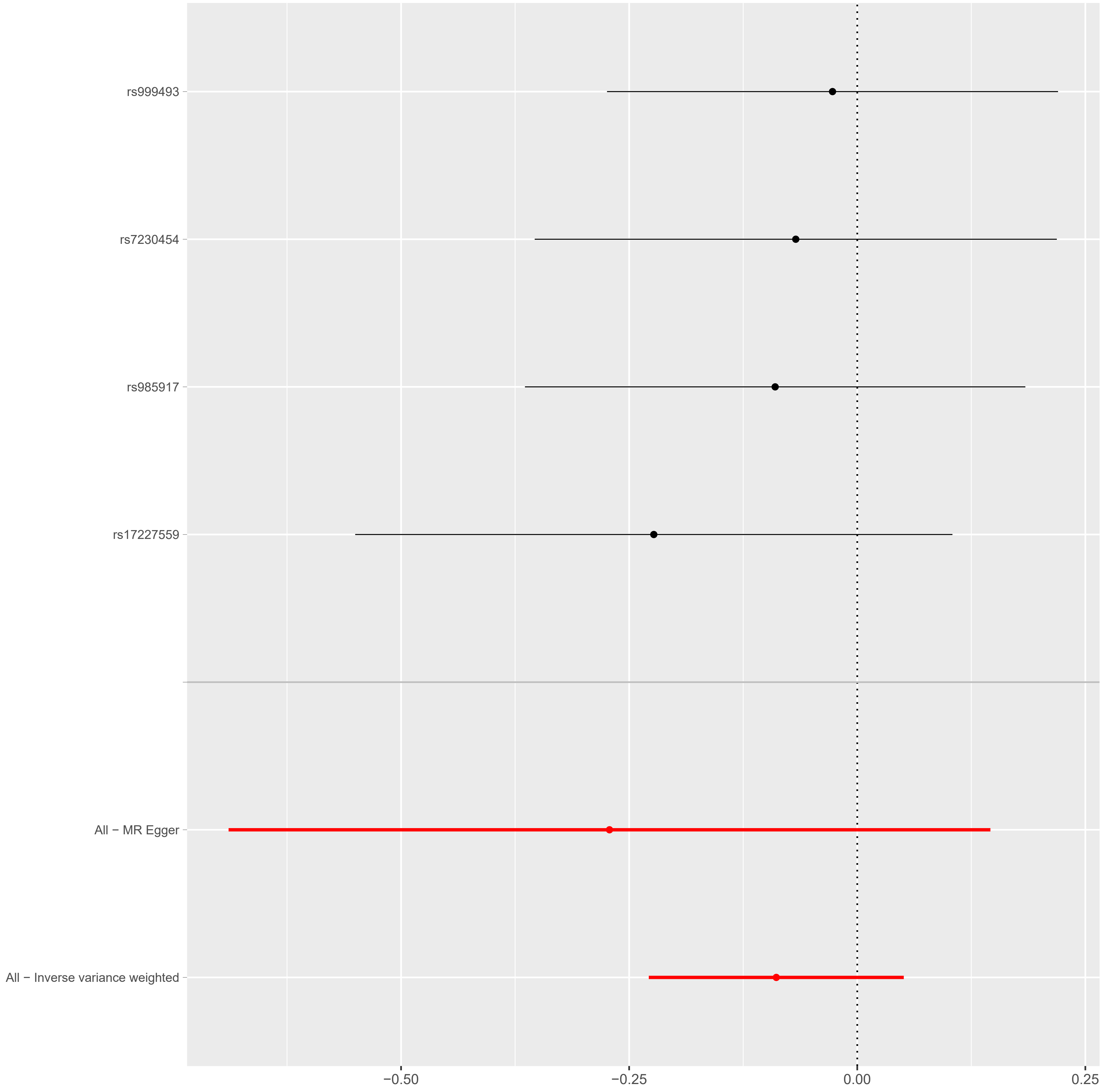

Forest plots for MR analyses of the causal effect of IGFBP-5 using each SNP singly on osteoporosis based on Finn trait

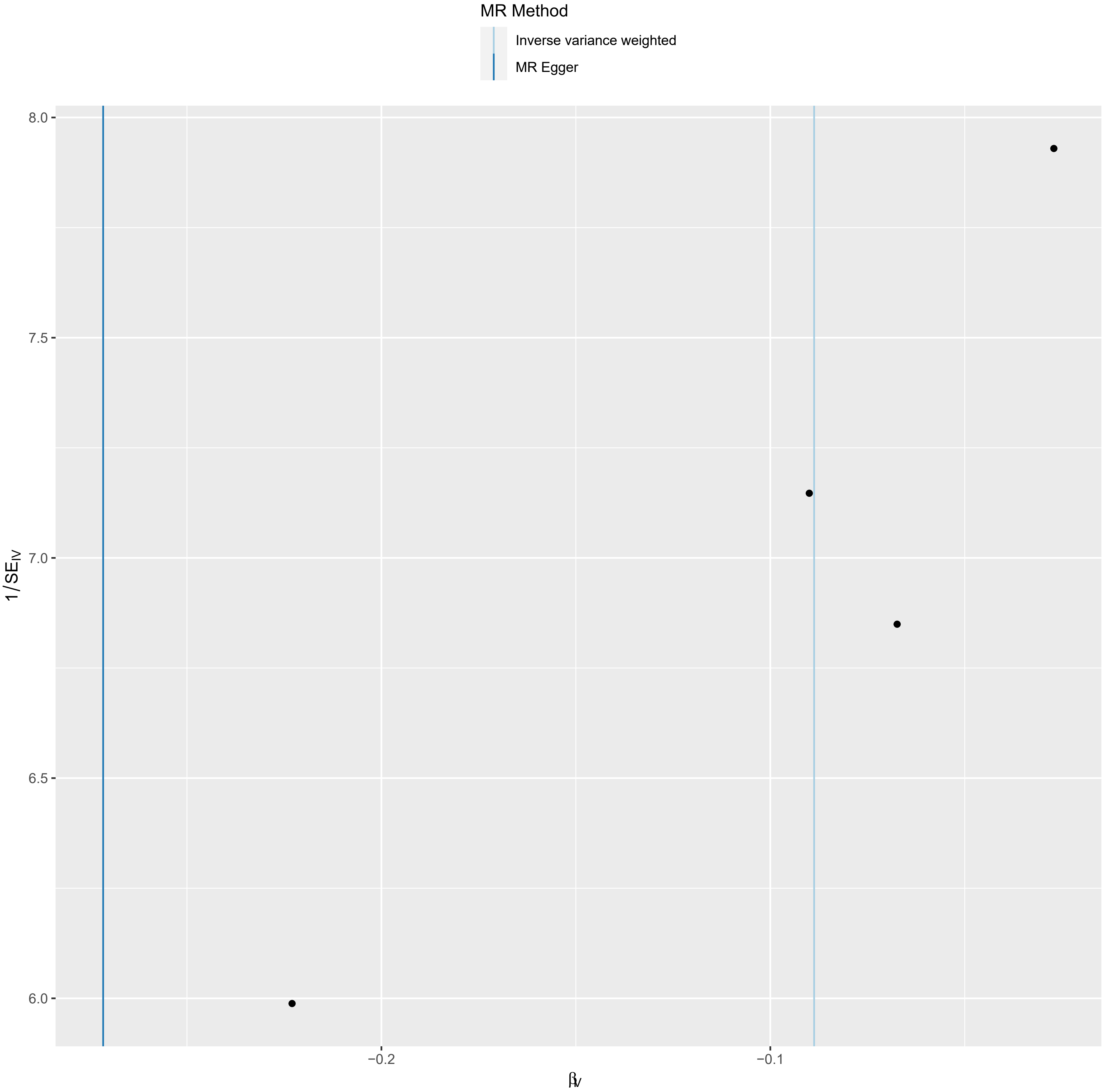

Funnal plots to assess heterogeneity for IGFBP-5 using all SNPs with the MR Egger and IVW methods

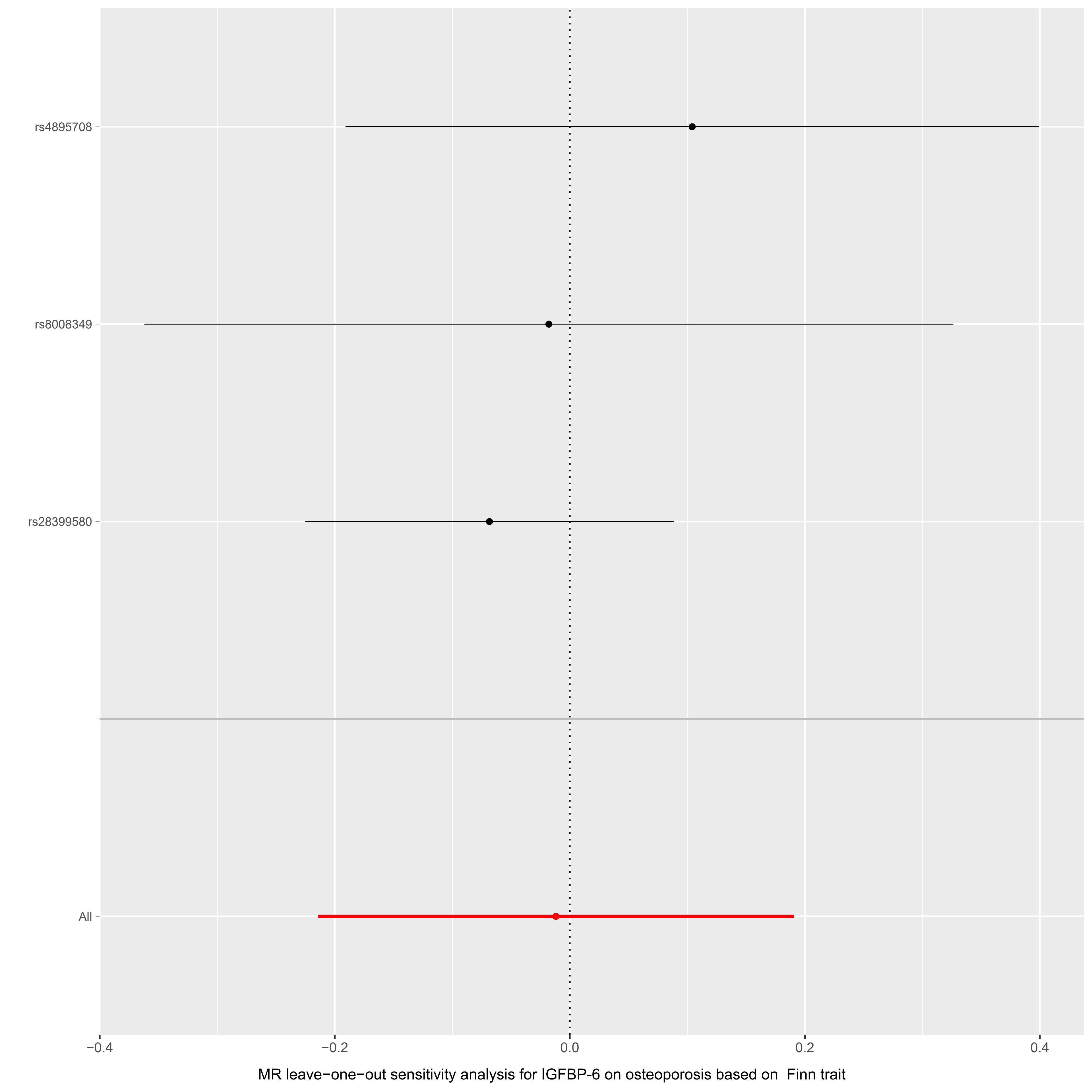
rs4895708
rs8008349
rs28399580
All
−0.4
−0.2
0.0
0.2
0.4
MR leave−one−out sensitivity analysis for IGFBP-6 on osteoporosis based on Finn trait

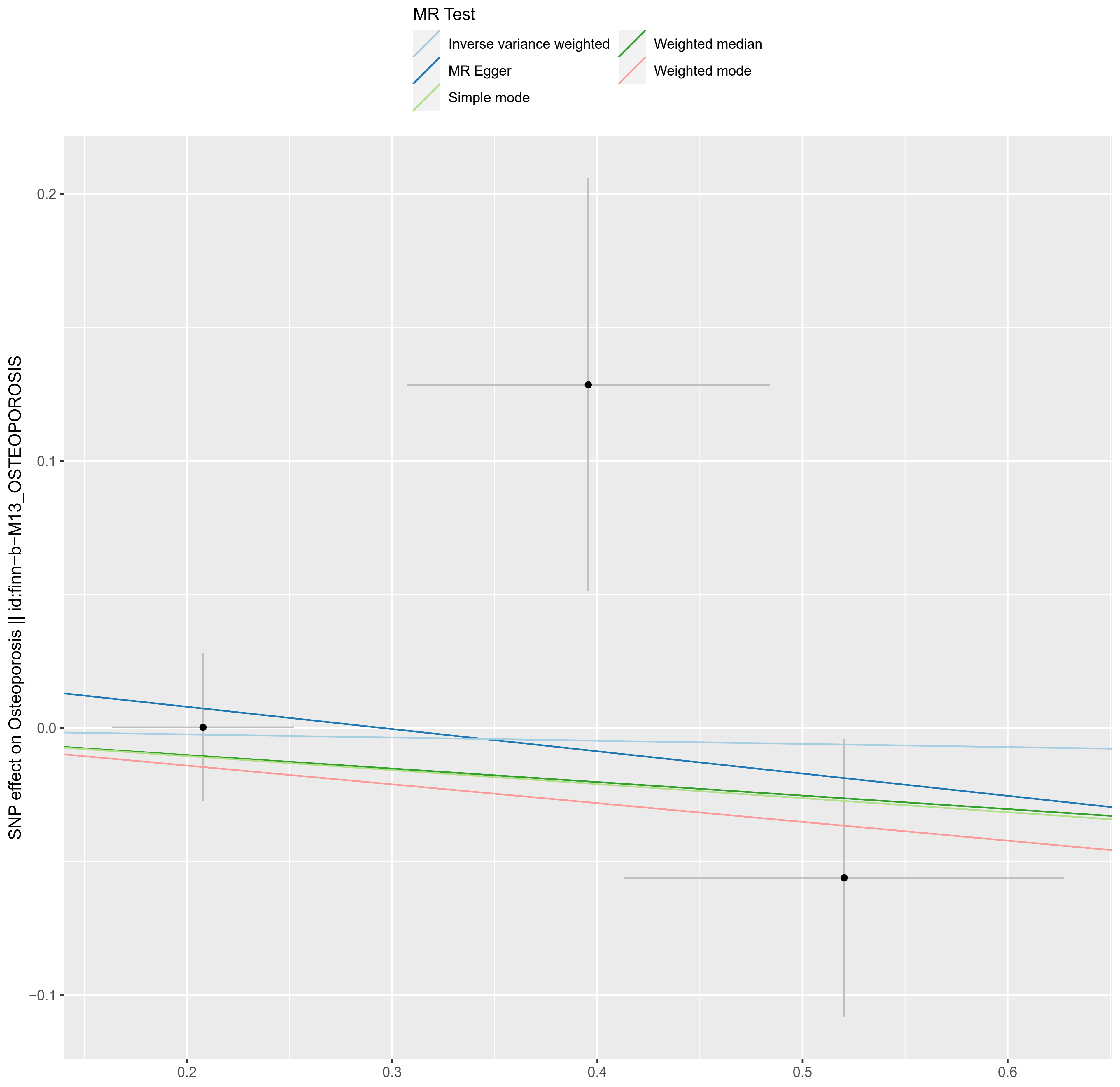

Scatter plots for MR analyses of the causal effect of IGFBP-6 on osteoporosis based on Finn trait

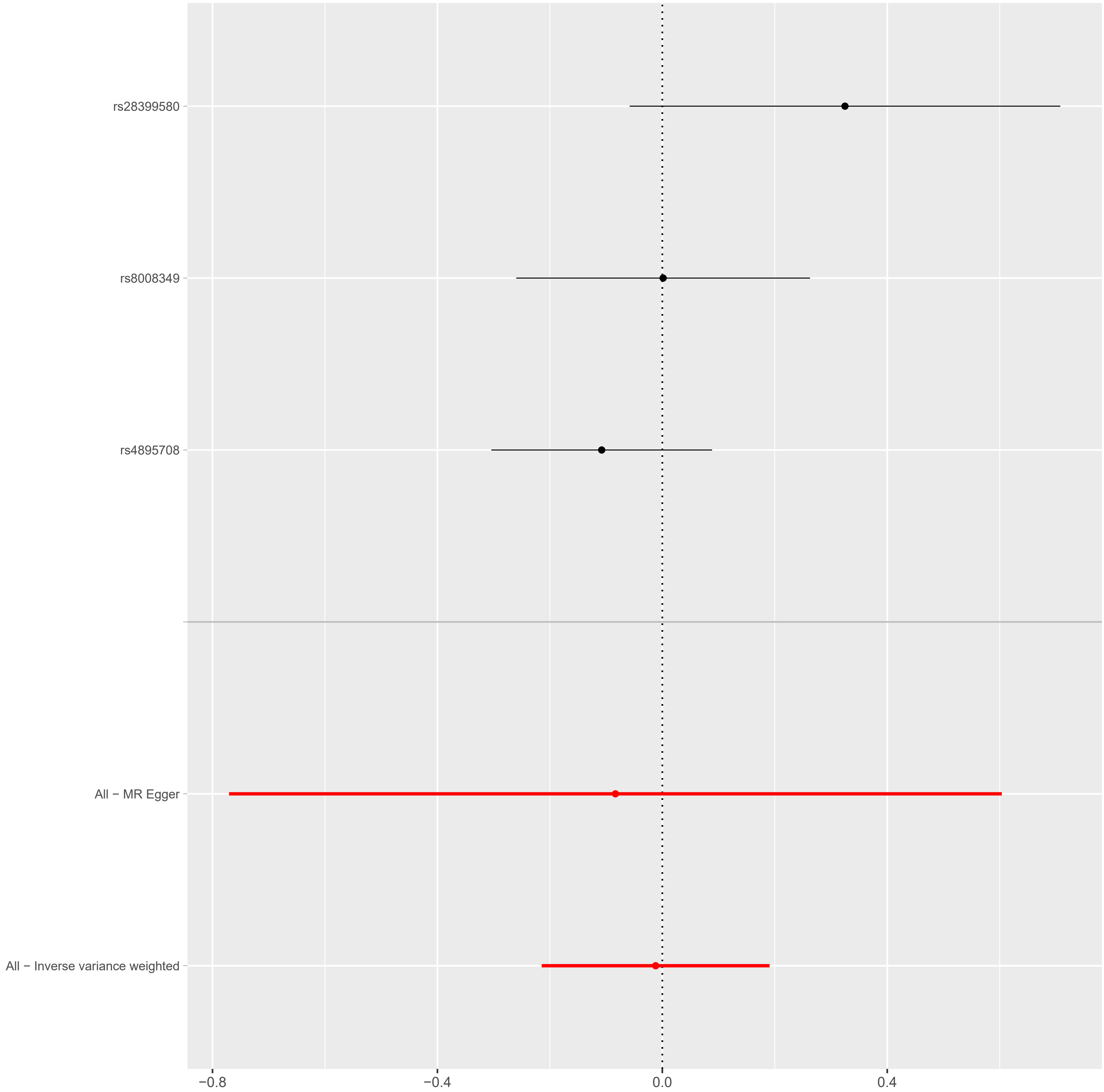

Forest plots for MR analyses of the causal effect of IGFBP-6 using each SNP singly on osteoporosis based on Finn trait

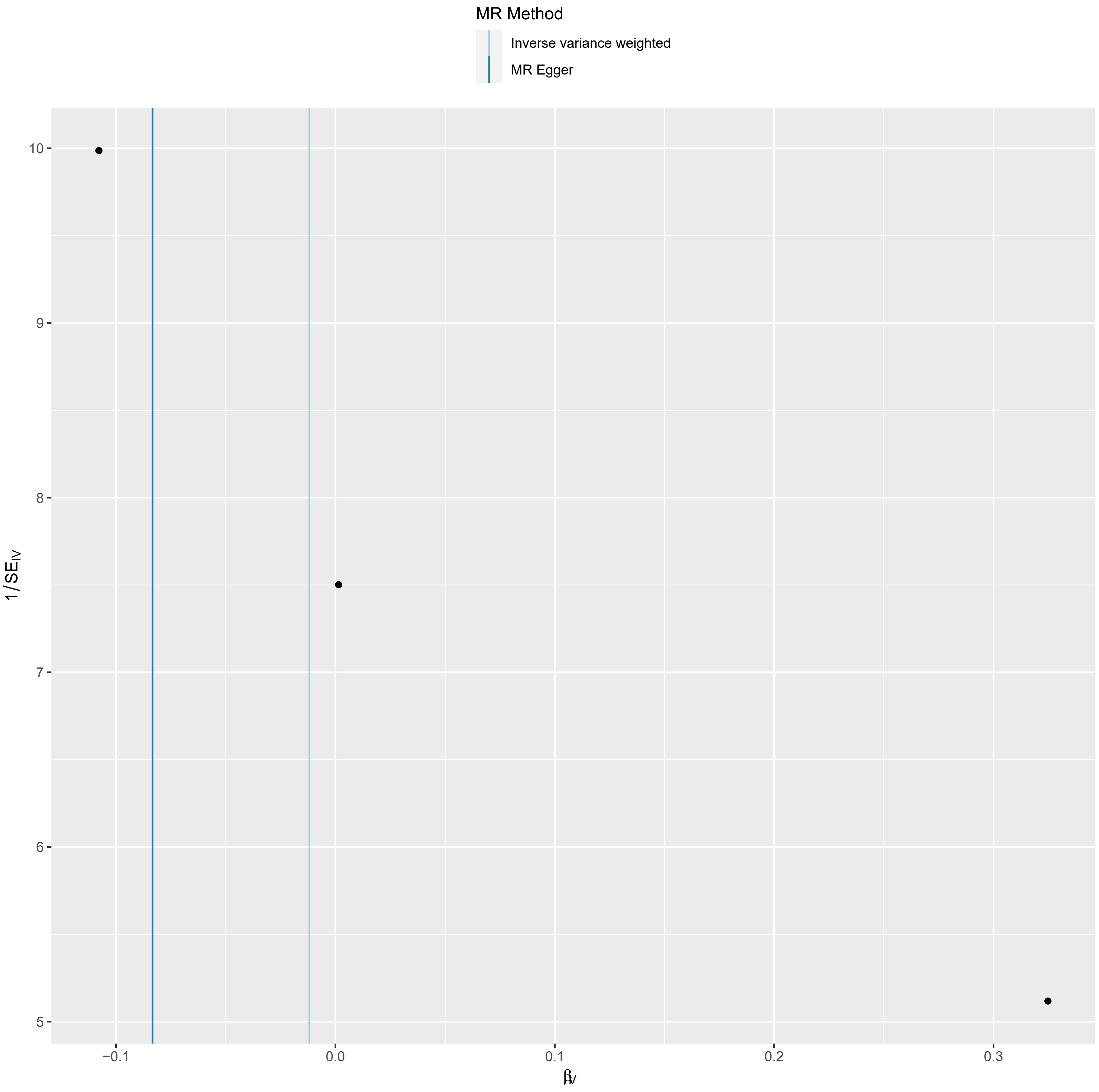

Funnal plots to assess heterogeneity for IGFBP-6 using all SNPs with the MR Egger and IVW methods

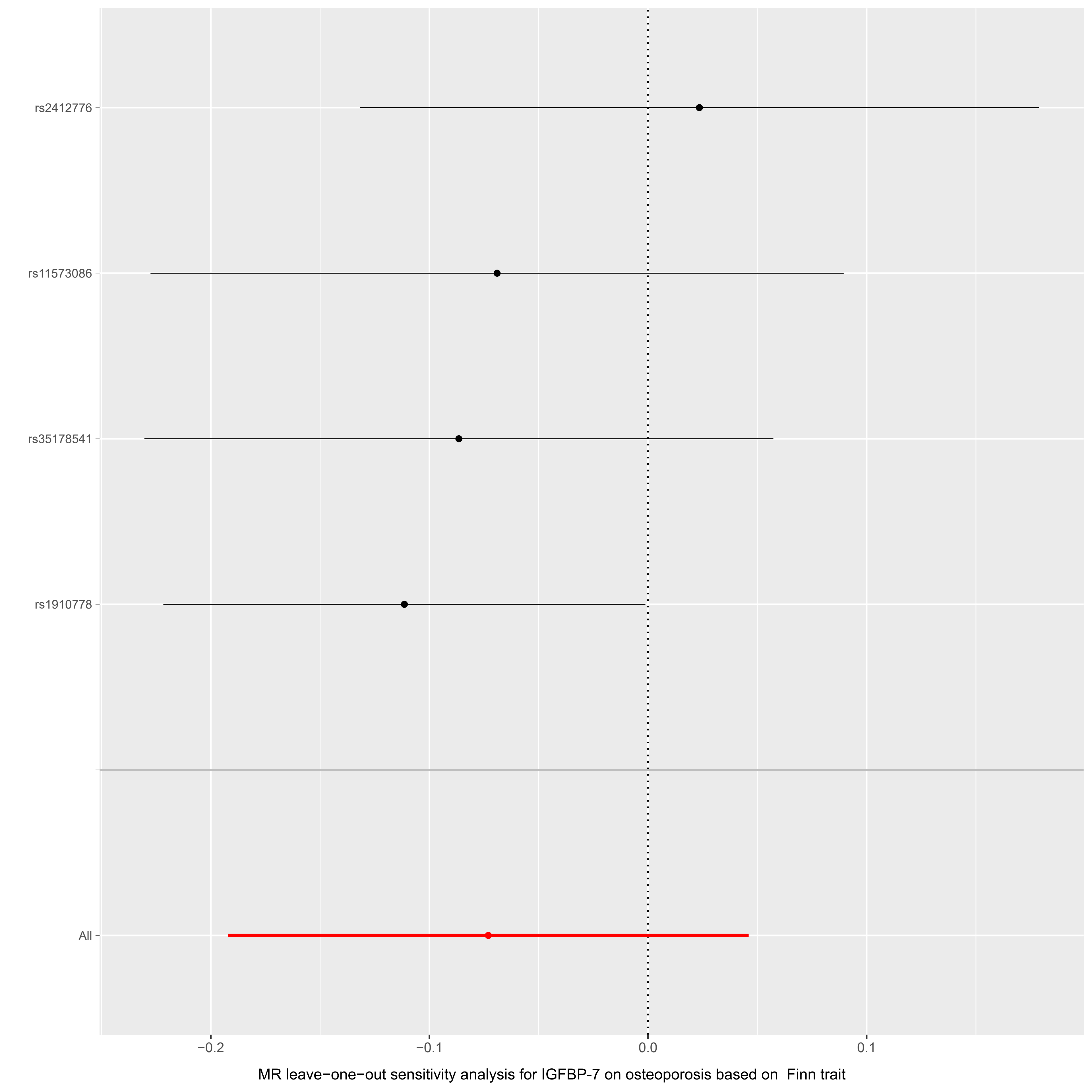
rs2412776
rs11573086
rs35178541
rs1910778
All
−0.2
−0.1
0.0
0.1
MR leave−one−out sensitivity analysis for IGFBP-7 on osteoporosis based on Finn trait

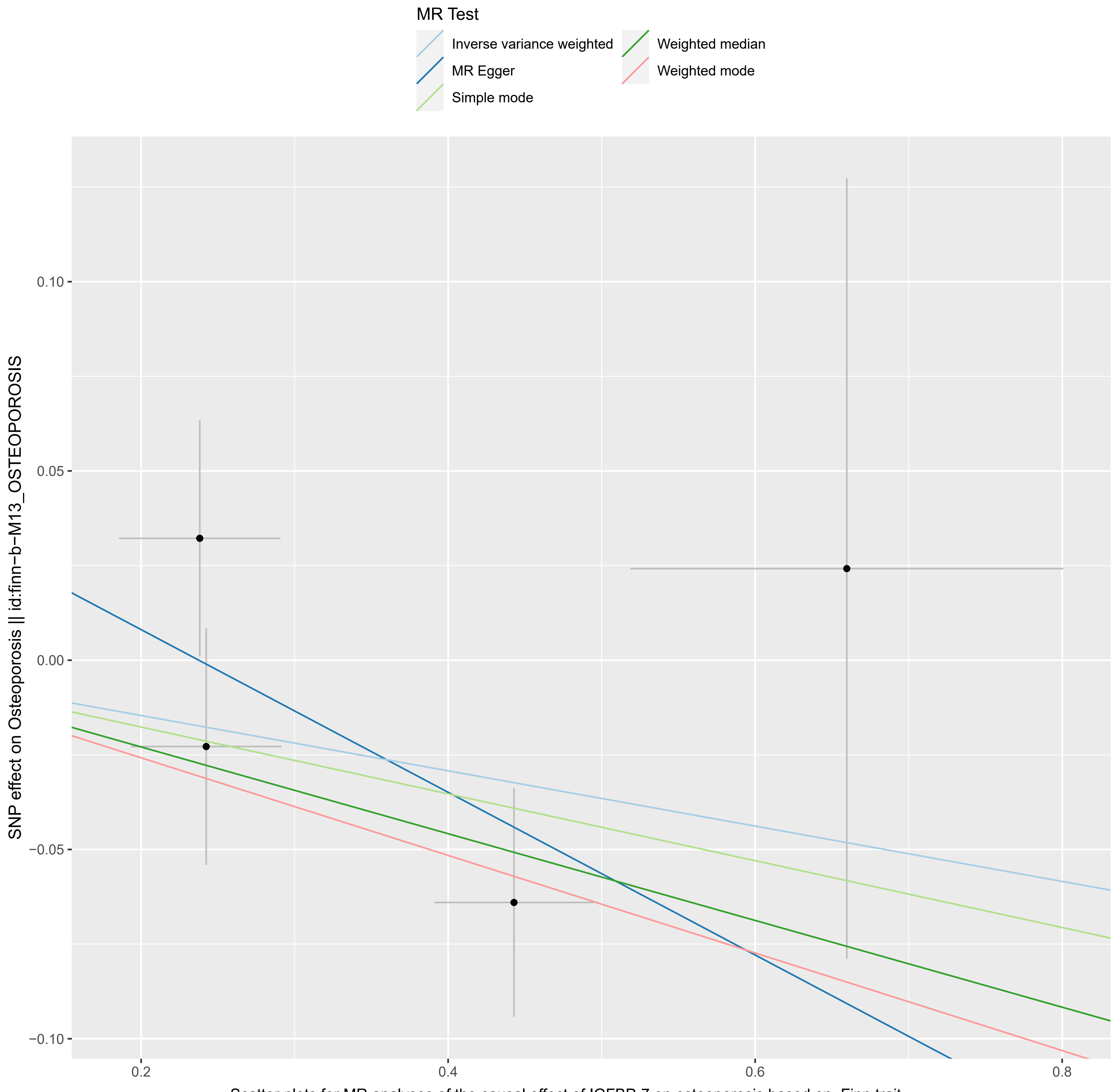

Scatter plots for MR analyses of the causal effect of IGFBP-7 on osteoporosis based on Finn trait

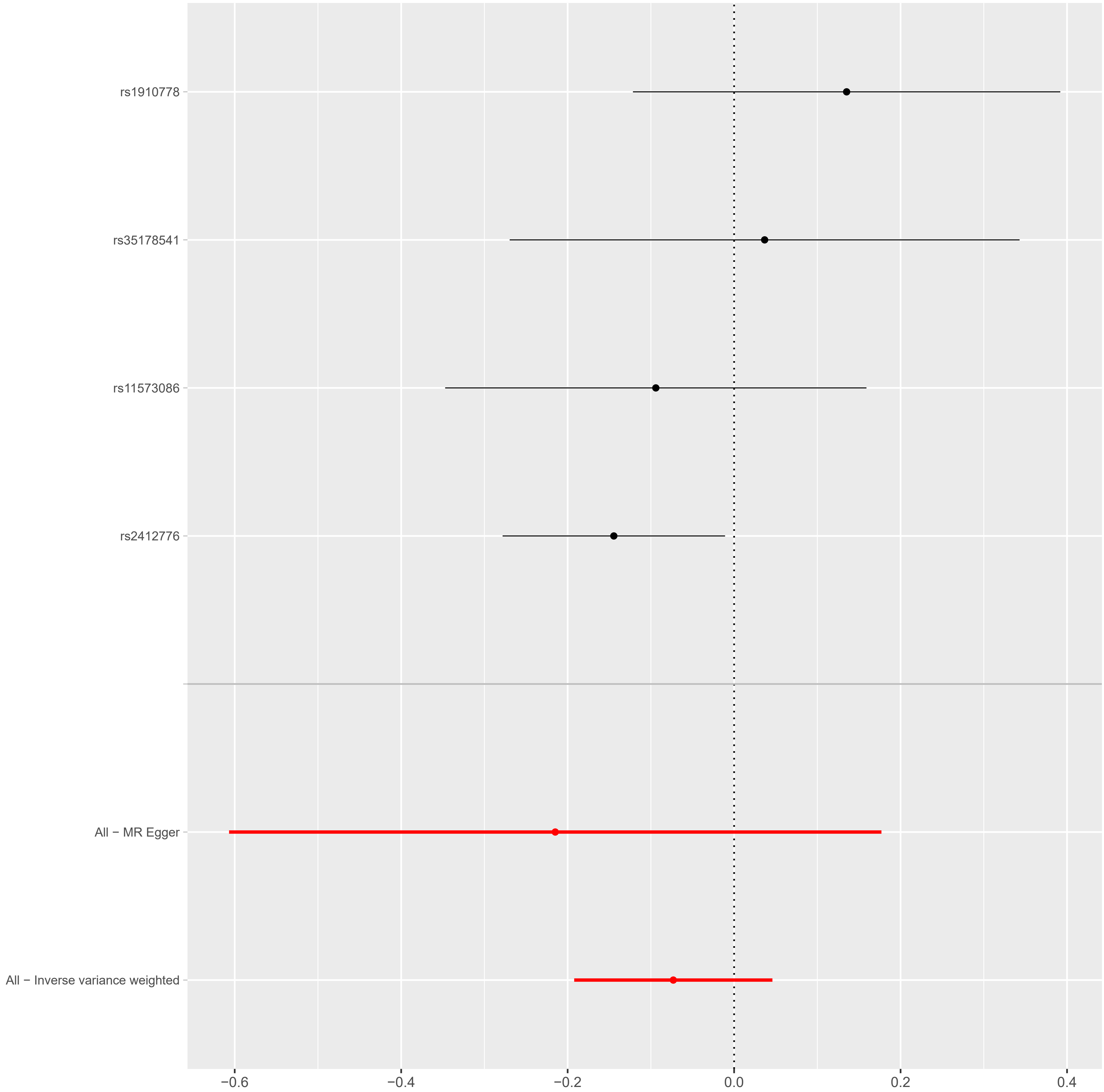

Forest plots for MR analyses of the causal effect of IGFBP-7 using each SNP singly on osteoporosis based on Finn trait

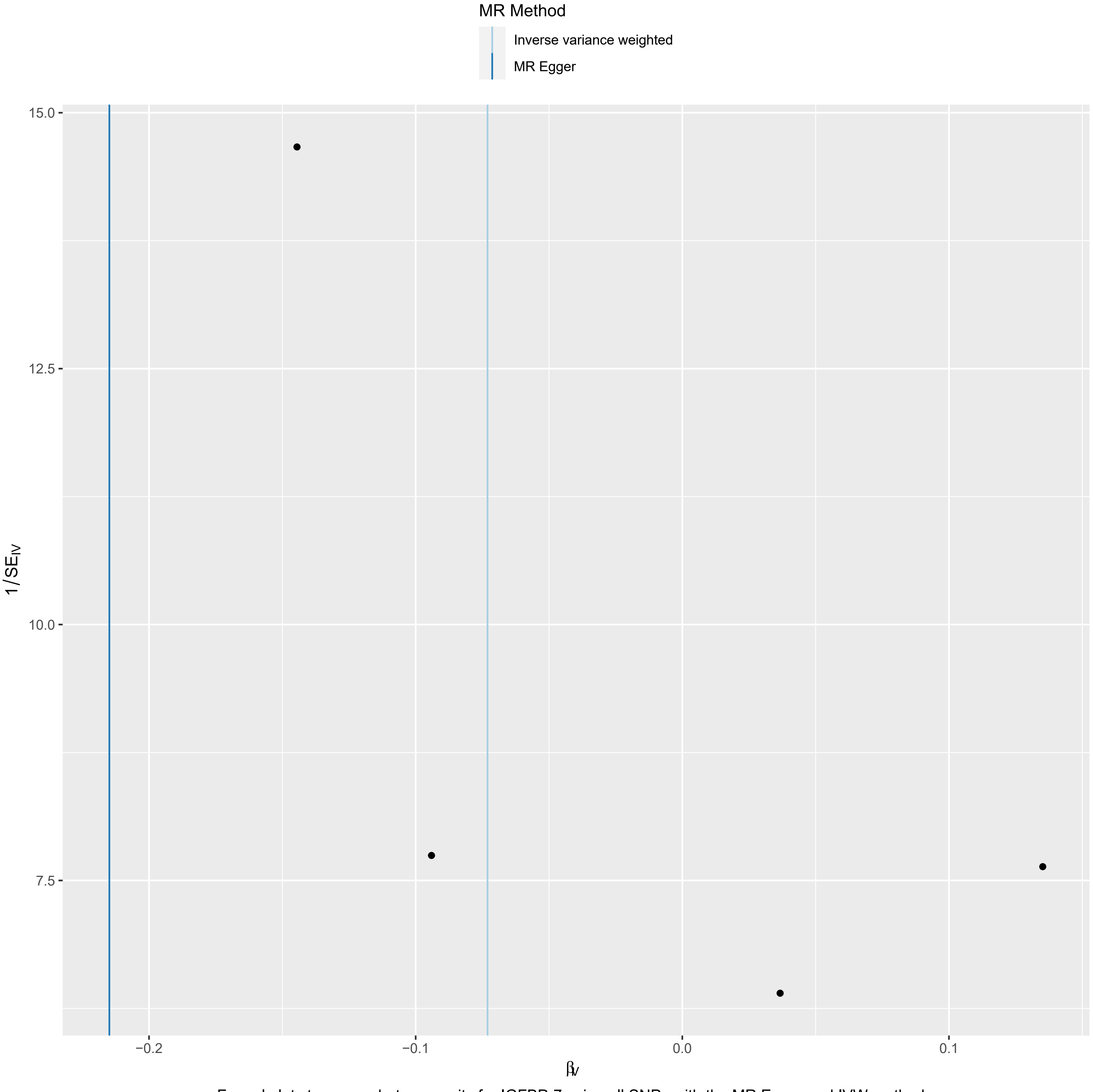

Funnal plots to assess heterogeneity for IGFBP-7 using all SNPs with the MR Egger and IVW methods

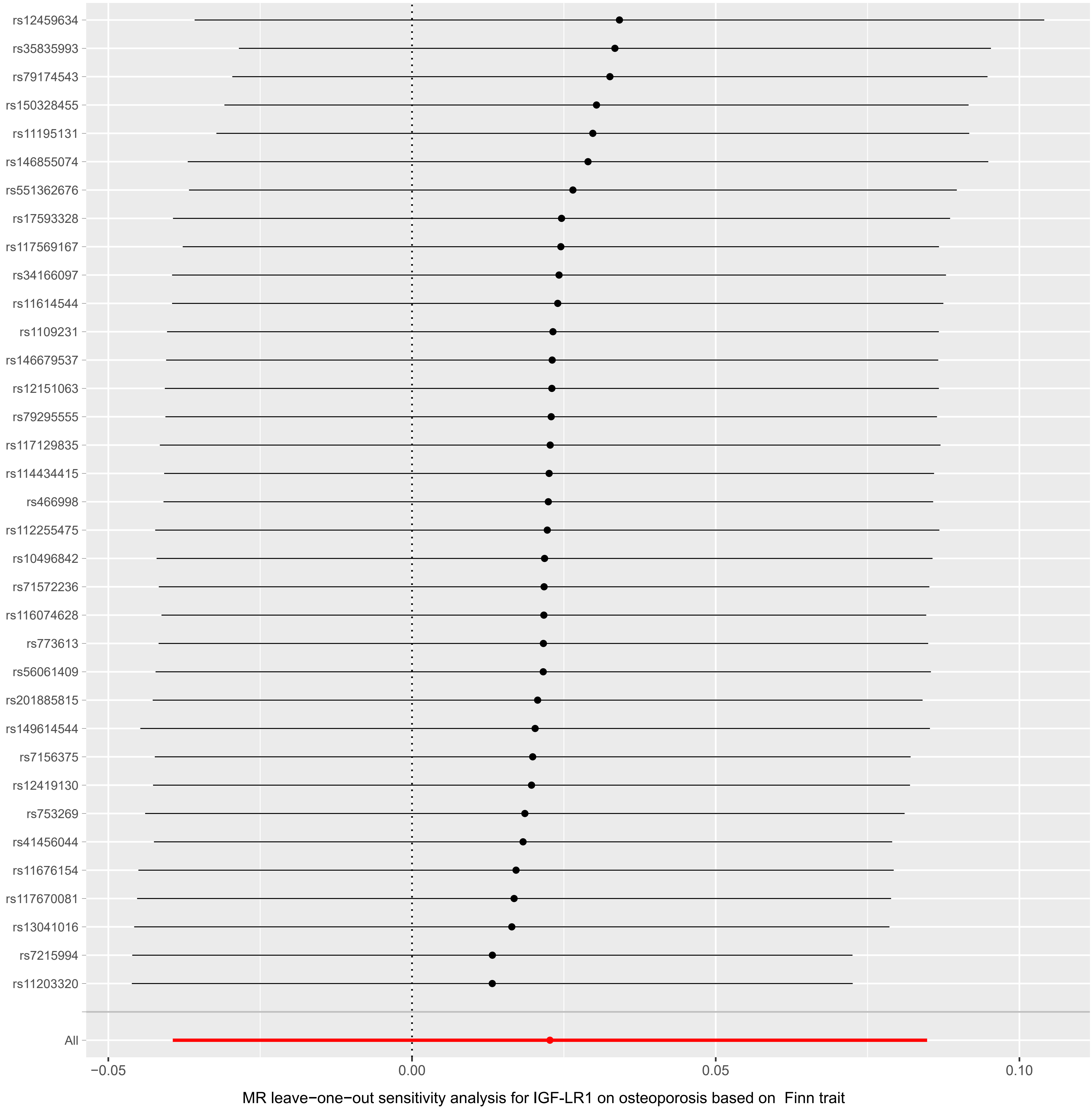

rs12459634
rs35835993
rs79174543
rs150328455
rs11195131
rs146855074
rs551362676
rs17593328
rs117569167
rs34166097
rs11614544
rs1109231
rs146679537
rs12151063
rs79295555
rs117129835
rs114434415
rs466998
rs112255475
rs10496842
rs71572236
rs116074628
rs773613
rs56061409
rs201885815
rs149614544
rs7156375
rs12419130
rs753269
rs41456044
rs11676154
rs117670081
rs13041016
rs7215994
rs11203320
All
−0.05
0.00
0.05
0.10
MR leave−one−out sensitivity analysis for IGF-LR1 on osteoporosis based on Finn trait

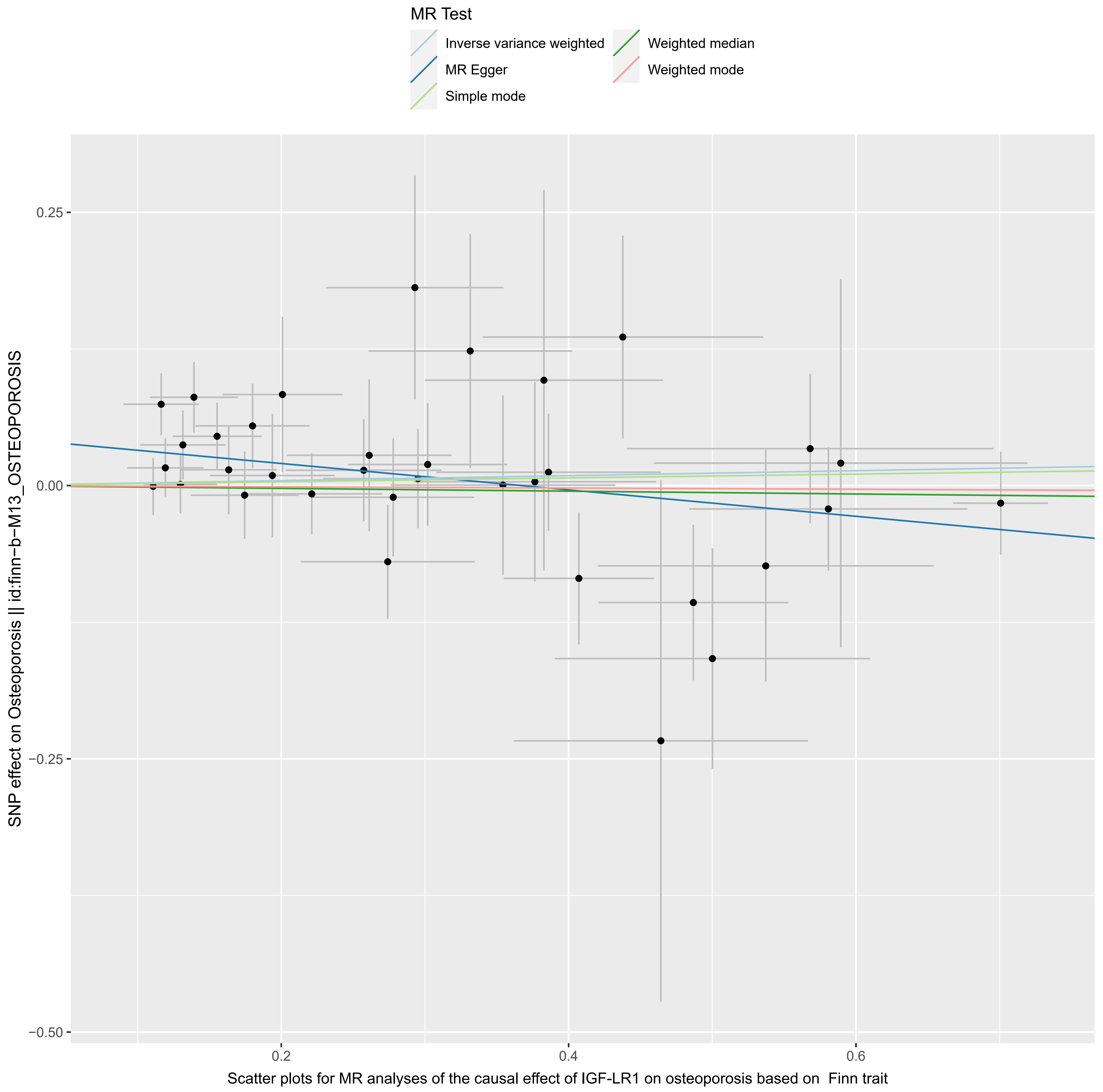
MR Test
Inverse variance weighted
MR Egger
Simple mode
Weighted median
Weighted mode
0.25
0.00
−0.25
−0.50
0.2
0.4
0.6
SNP effect on Osteoporosis || id:finn-b-M13_OSTEOPOROSIS
Scatter plots for MR analyses of the causal effect of IGF-LR1 on osteoporosis based on Finn trait

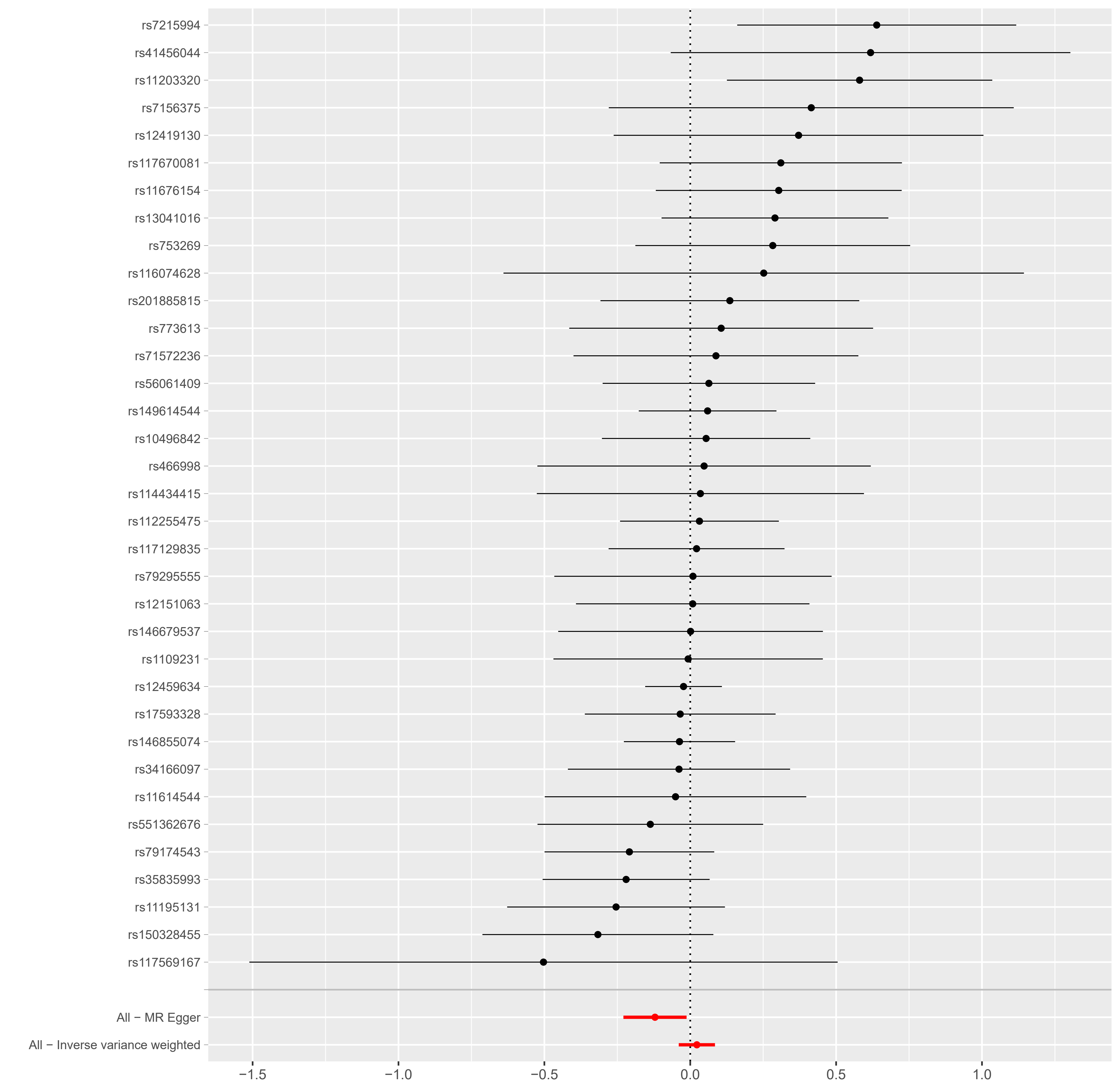

Forest plots for MR analyses of the causal effect of IGF-LR1 using each SNP singly on osteoporosis based on Finn trait

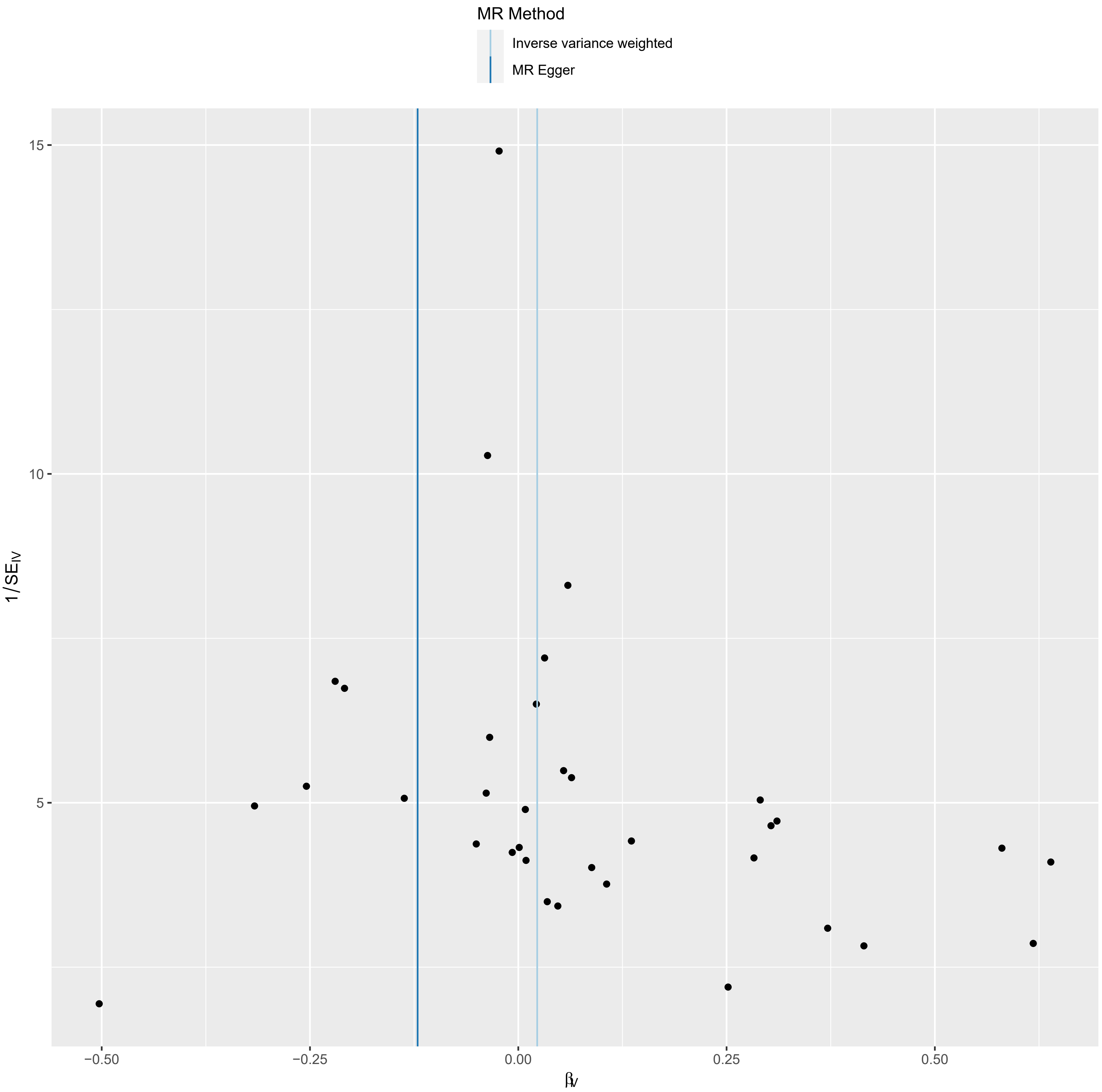

Funnal plots to assess heterogeneity for IGF1-LR1using all SNPs with the MR Egger and IVW methods

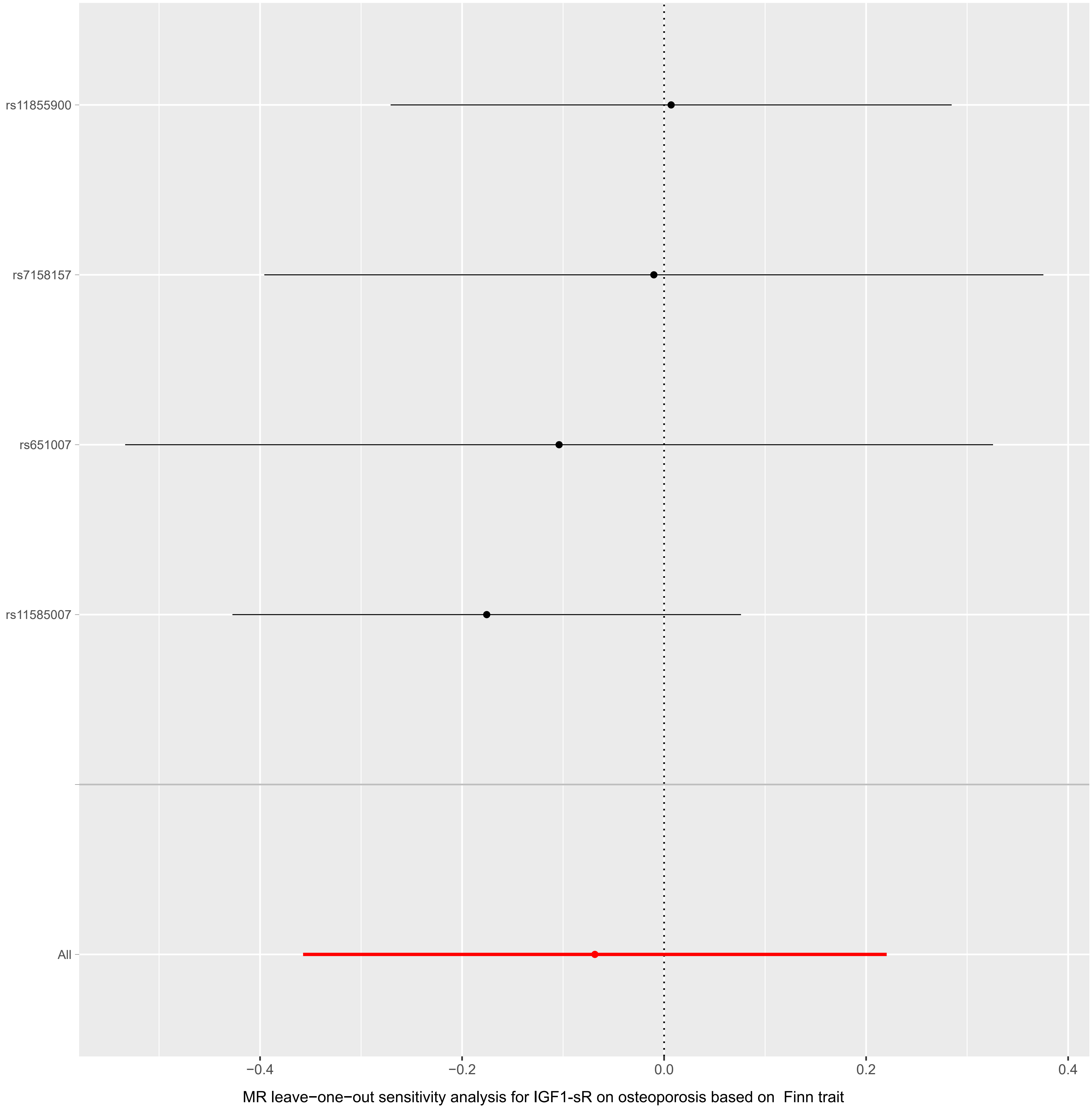
rs11855900
rs7158157
rs651007
rs11585007
All
−0.4
−0.2
0.0
0.2
0.4
MR leave−one−out sensitivity analysis for IGF1-sR on osteoporosis based on Finn trait

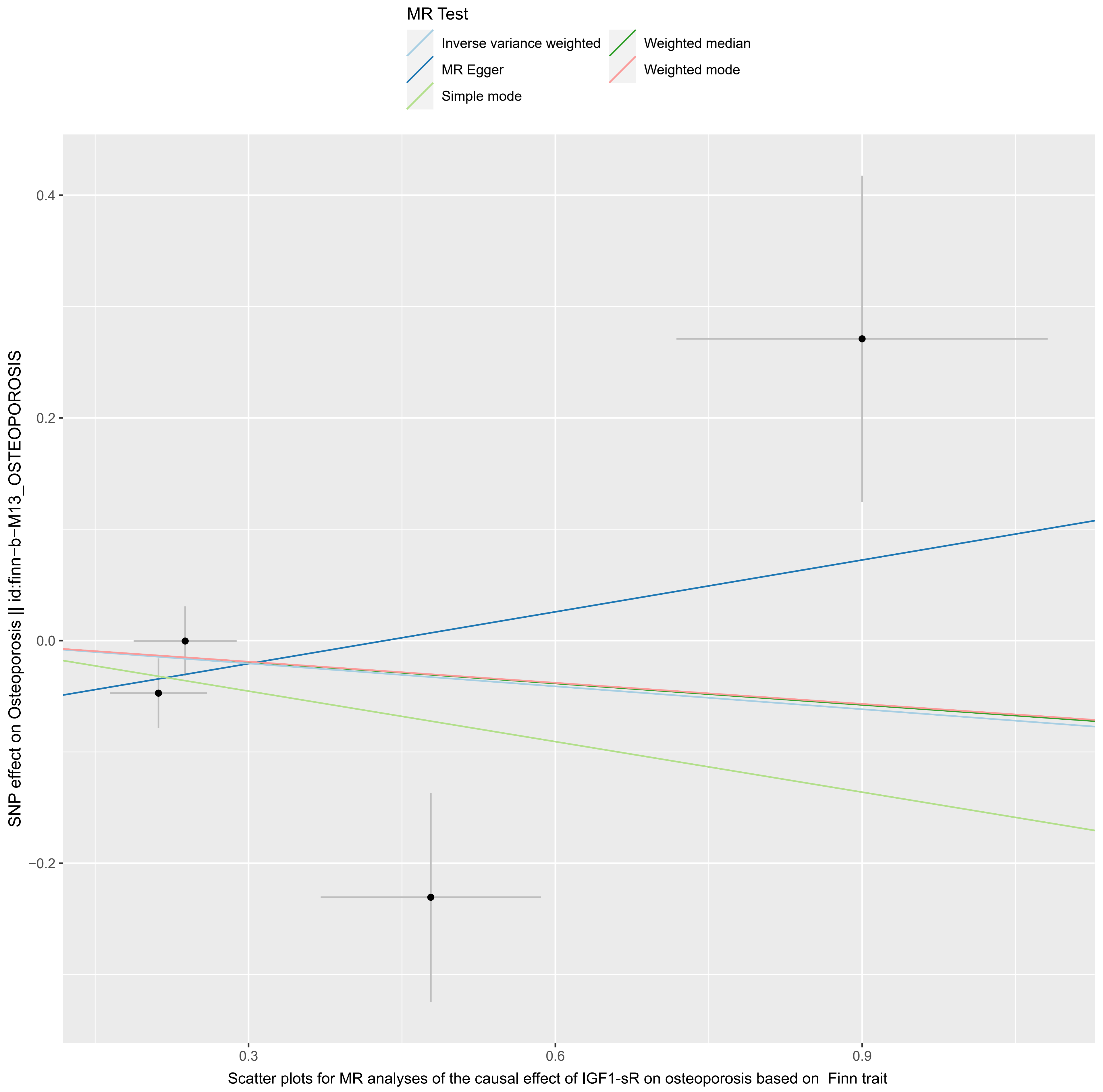
MR Test
Inverse variance weighted
MR Egger
Simple mode
Weighted median
Weighted mode
SNP effect on Osteoporosis || id:finn−b−M13_OSTEOPOROSIS
0.4
0.2
0.0
−0.2
0.3
0.6
0.9
Scatter plots for MR analyses of the causal effect of IGF1-sR on osteoporosis based on Finn trait

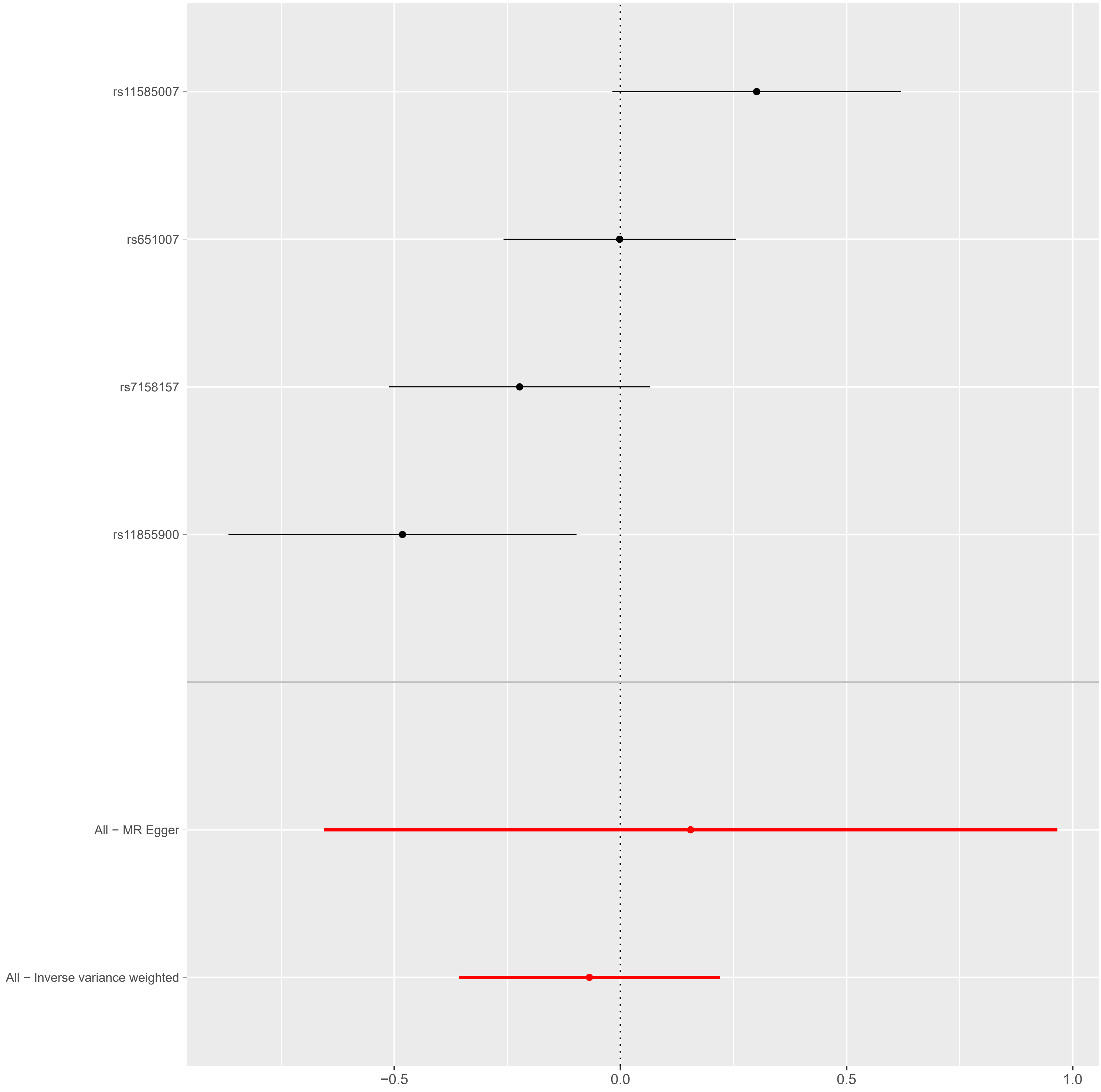

Forest plots for MR analyses of the causal effect of IGF1-sR using each SNP singly on osteoporosis based on Finn trait

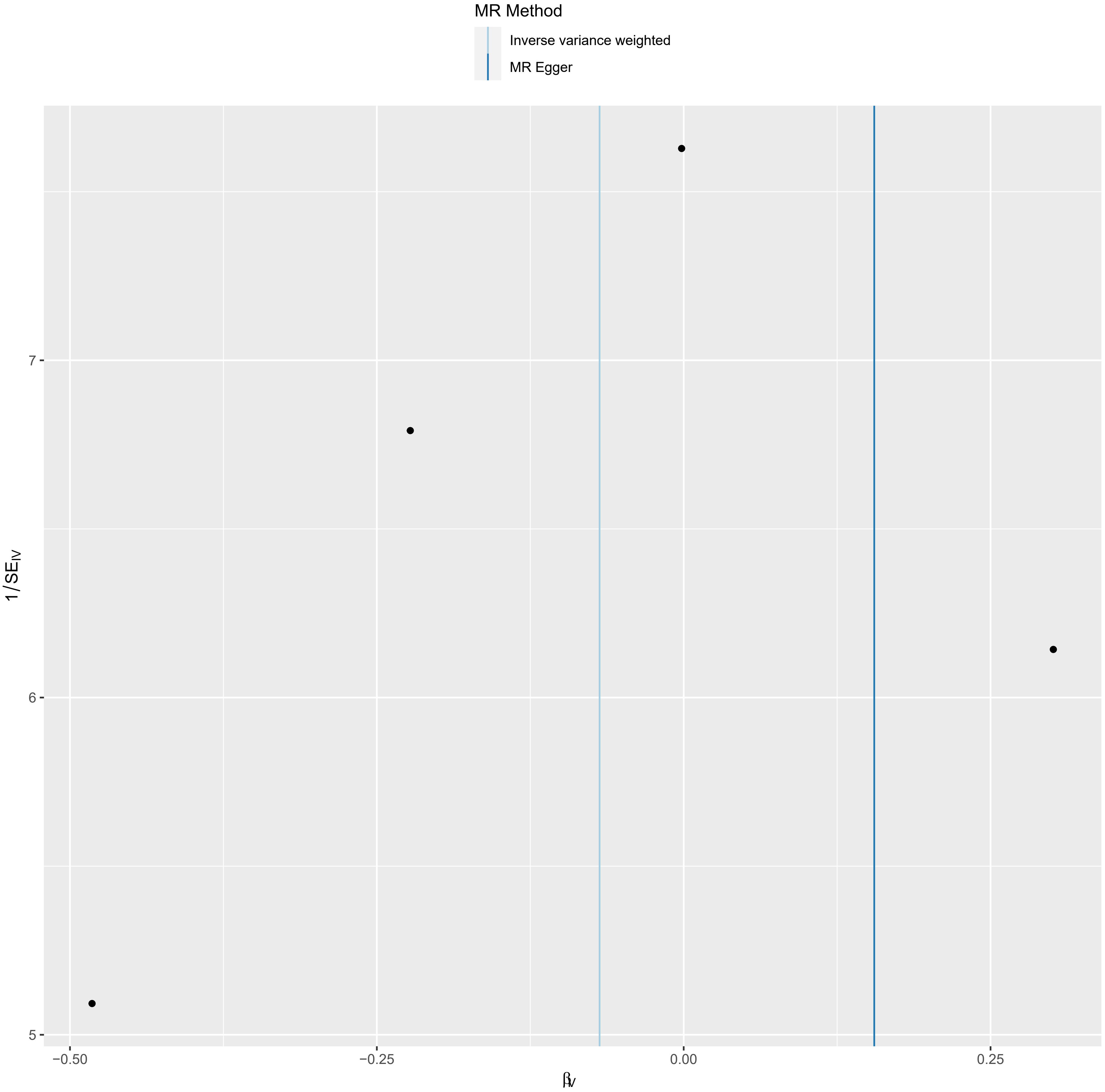

Funnal plots to assess heterogeneity for IGF1-sR using all SNPs with the MR Egger and IVW methods

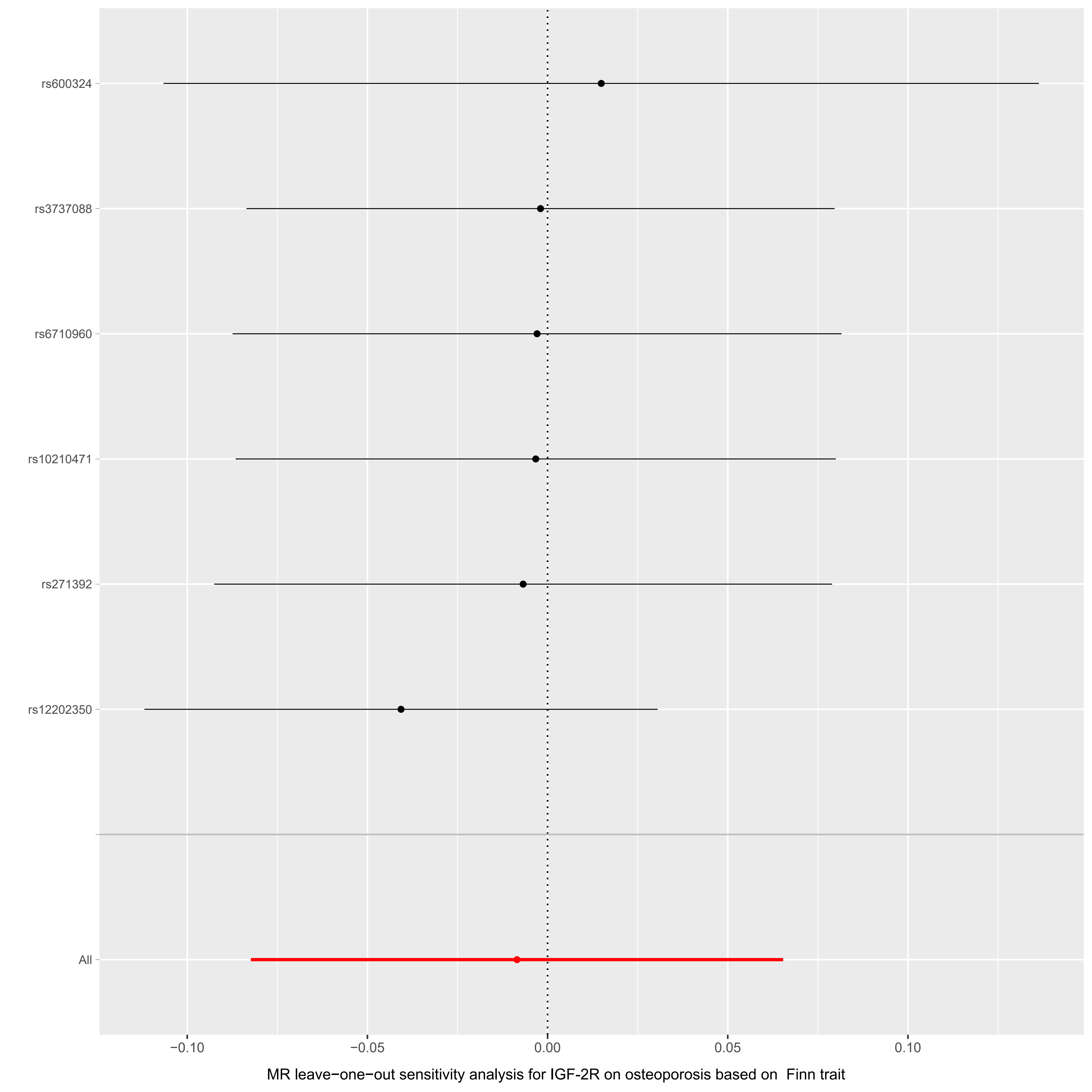
rs600324
rs3737088
rs6710960
rs10210471
rs271392
rs12202350
All
−0.10
−0.05
0.00
0.05
0.10
MR leave−one−out sensitivity analysis for IGF-2R on osteoporosis based on Finn trait

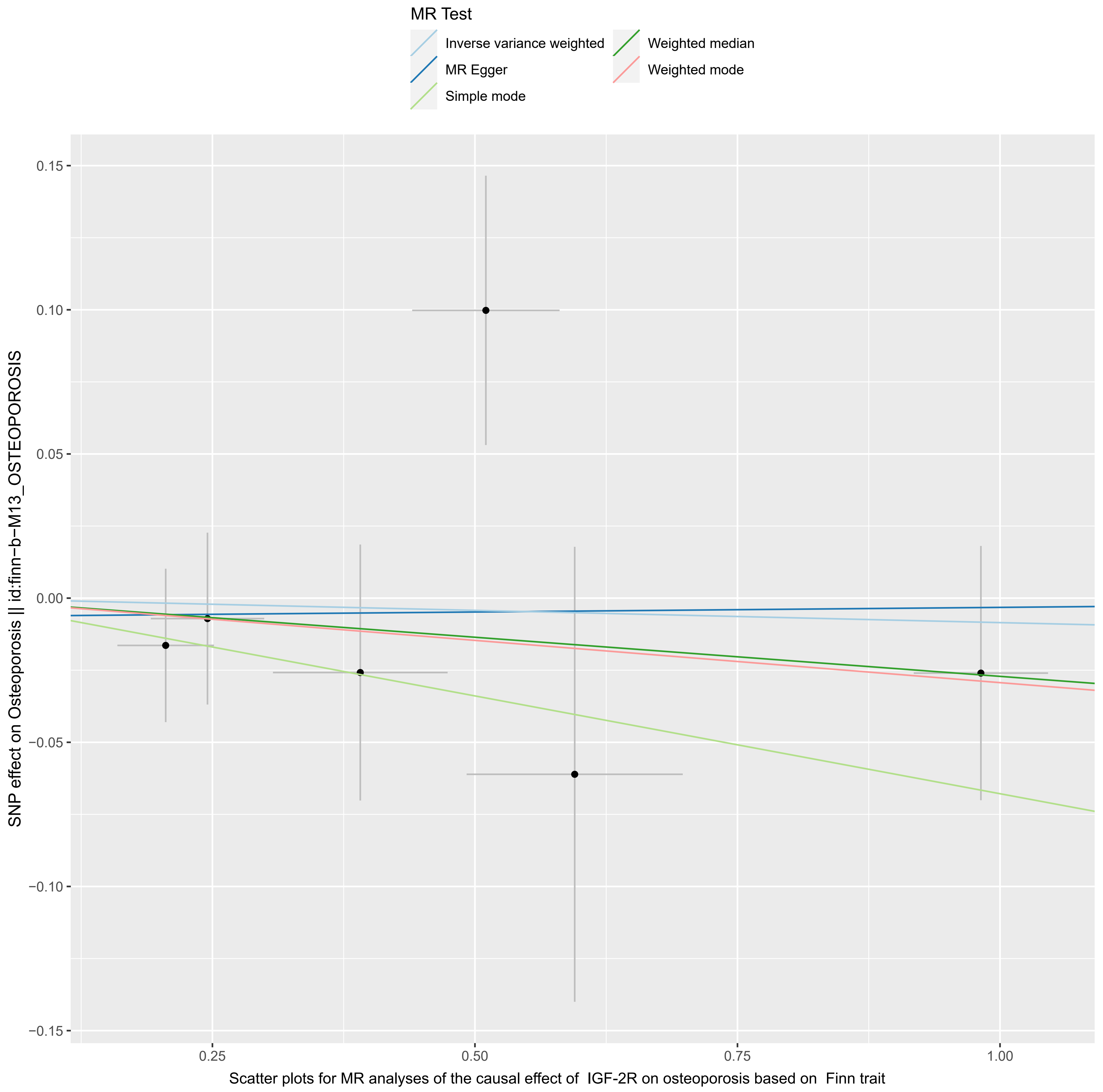
MR Test
Inverse variance weighted
MR Egger
Simple mode
Weighted median
Weighted mode
0.15
0.10
0.05
0.00
−0.05
−0.10
−0.15
SNP effect on Osteoporosis || id:finn-b-M13_OSTEOPOROSIS
0.25
0.50
0.75
1.00
Scatter plots for MR analyses of the causal effect of IGF-2R on osteoporosis based on Finn trait

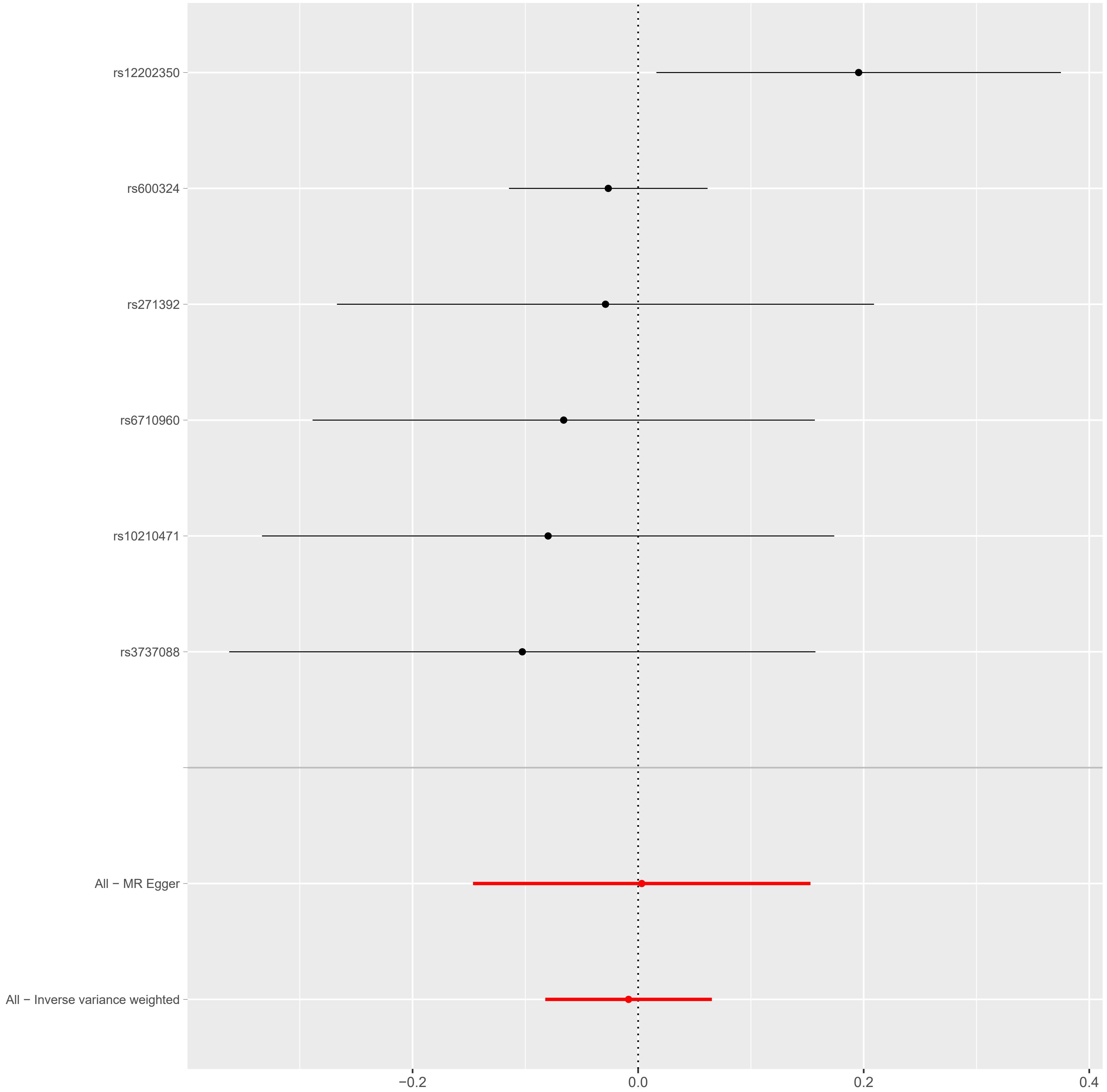

Forest plots for MR analyses of the causal effect of IGF-2R using each SNP singly on osteoporosis based on Finn trait

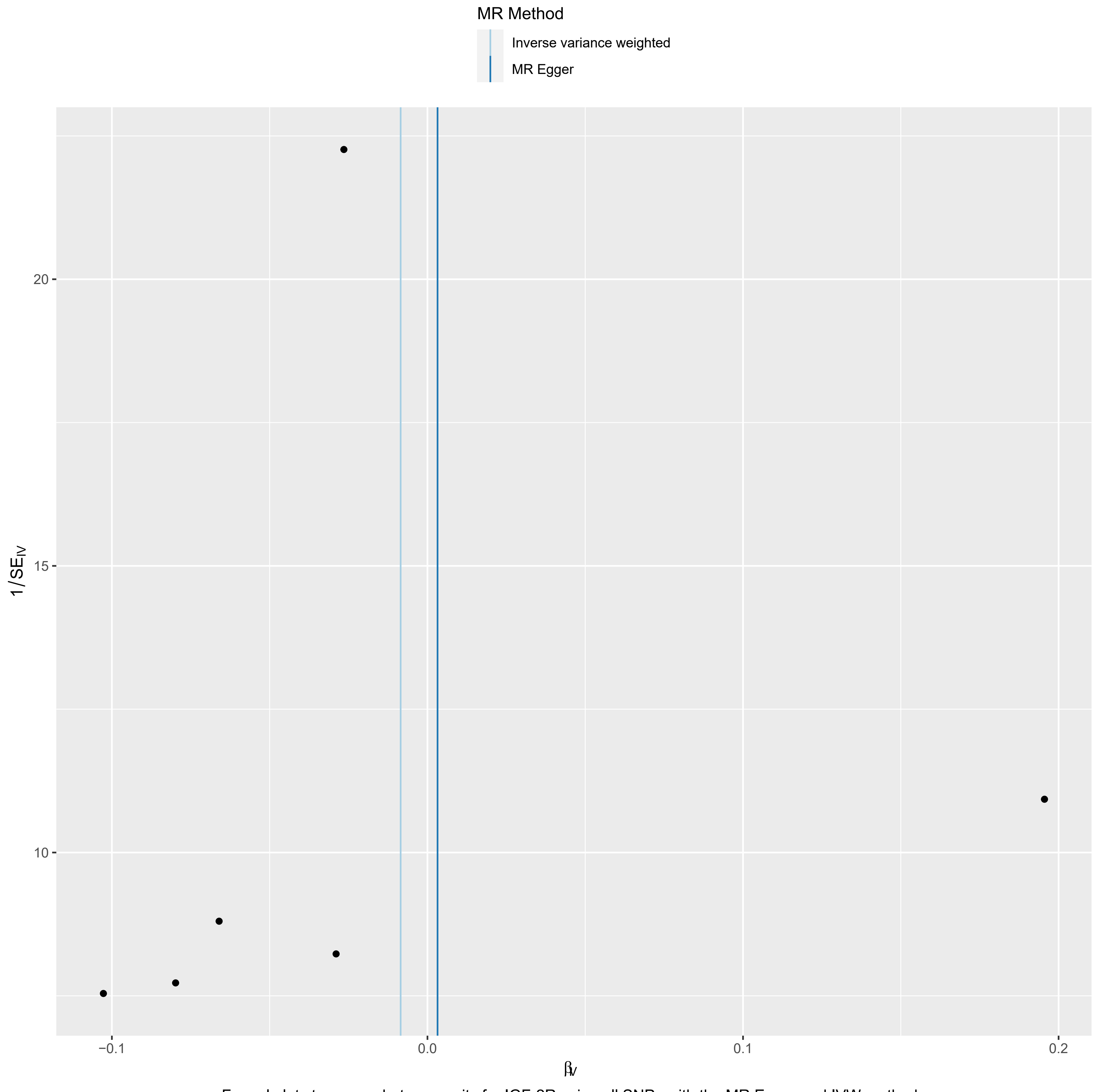

Funnal plots to assess heterogeneity for IGF-2R using all SNPs with the MR Egger and IVW methods

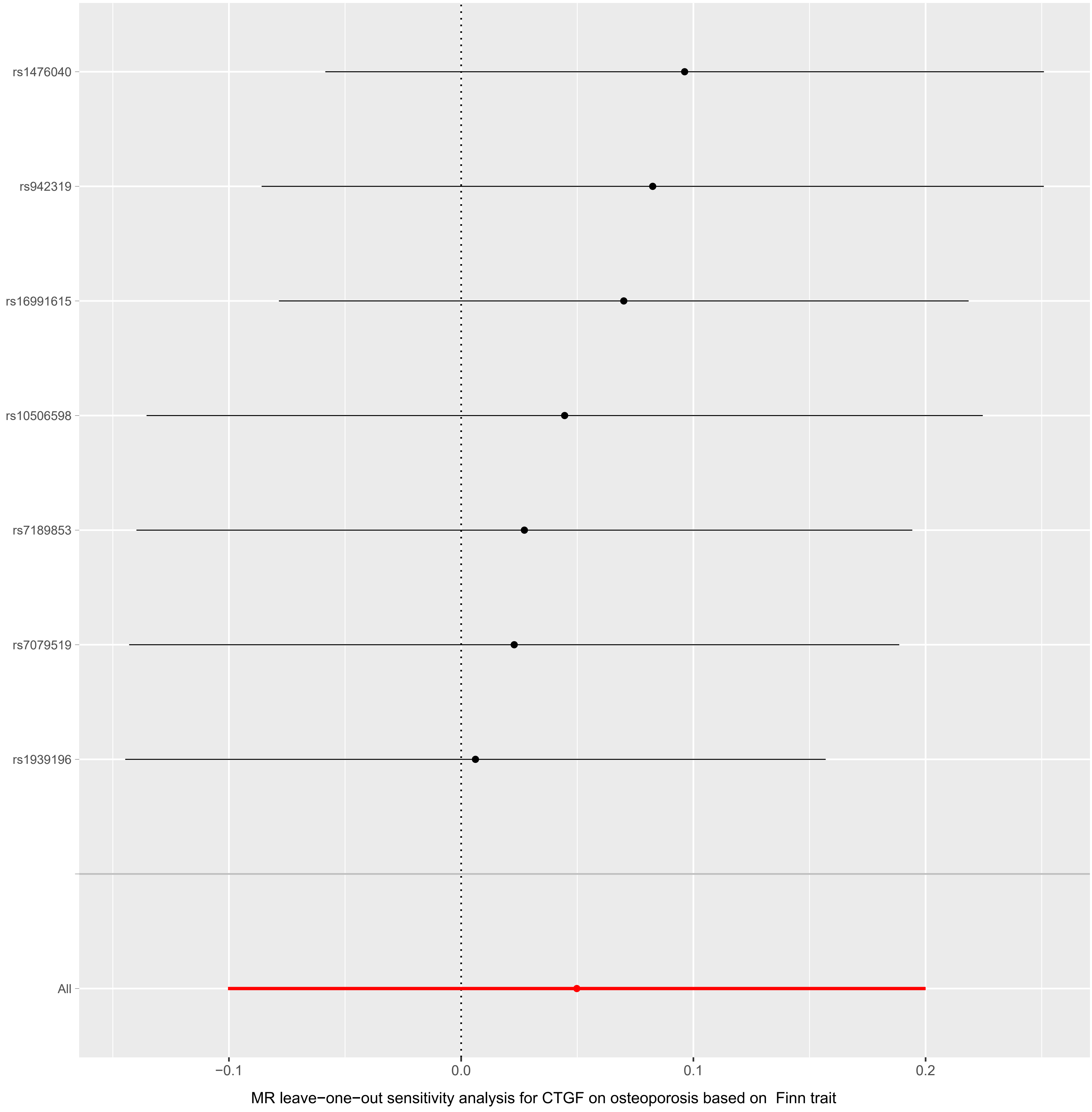
rs1476040
rs942319
rs16991615
rs10506598
rs7189853
rs7079519
rs1939196
All
−0.1
0.0
0.1
0.2
MR leave−one−out sensitivity analysis for CTGF on osteoporosis based on Finn trait

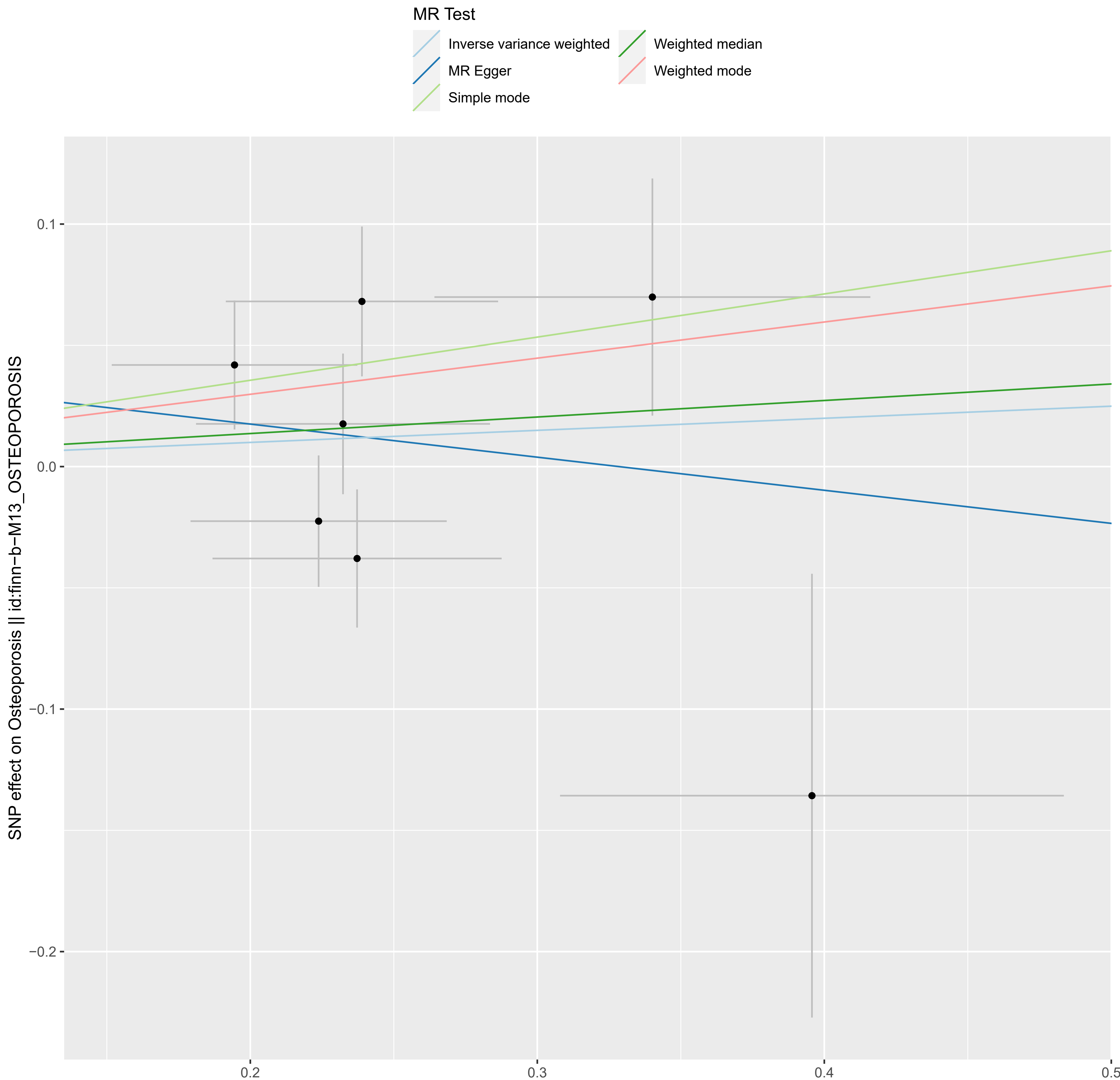

Scatter plots for MR analyses of the causal effect of CTGF on osteoporosis based on Finn trait

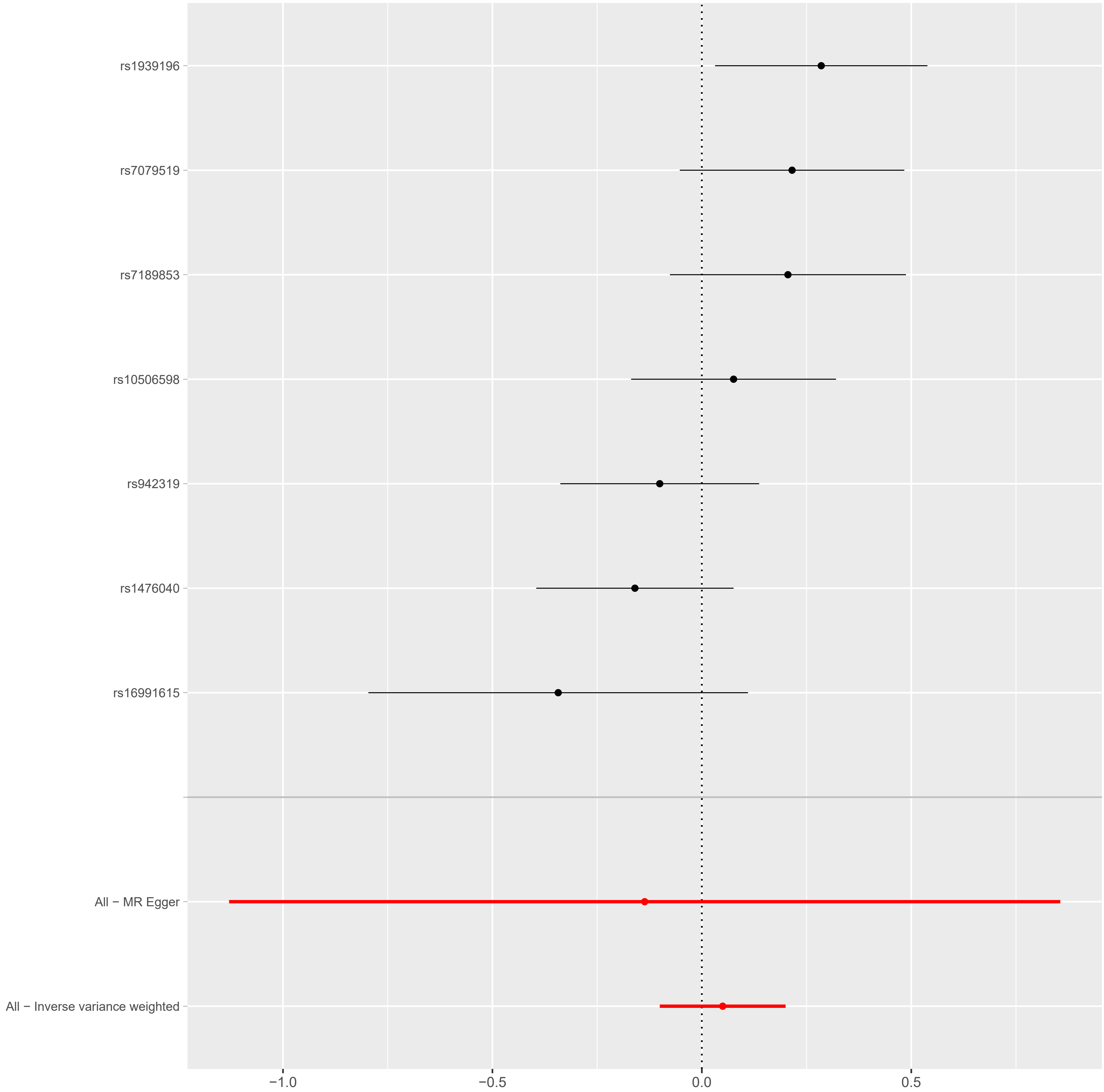

Forest plots for MR analyses of the causal effect of CTGF using each SNP singly on osteoporosis based on Finn trait

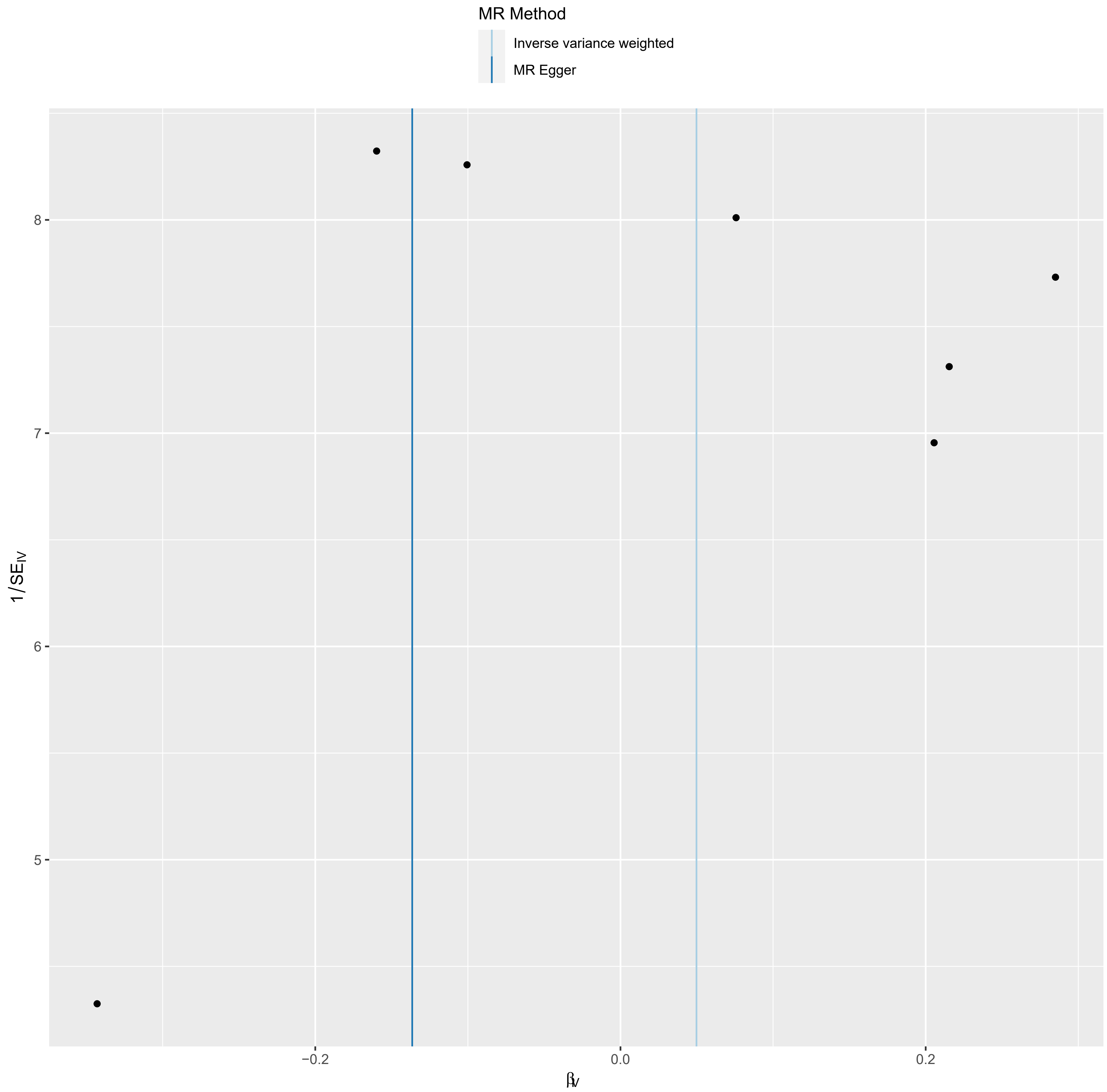

Funnal plots to assess heterogeneity for CTGF using all SNPs with the MR Egger and IVW methods

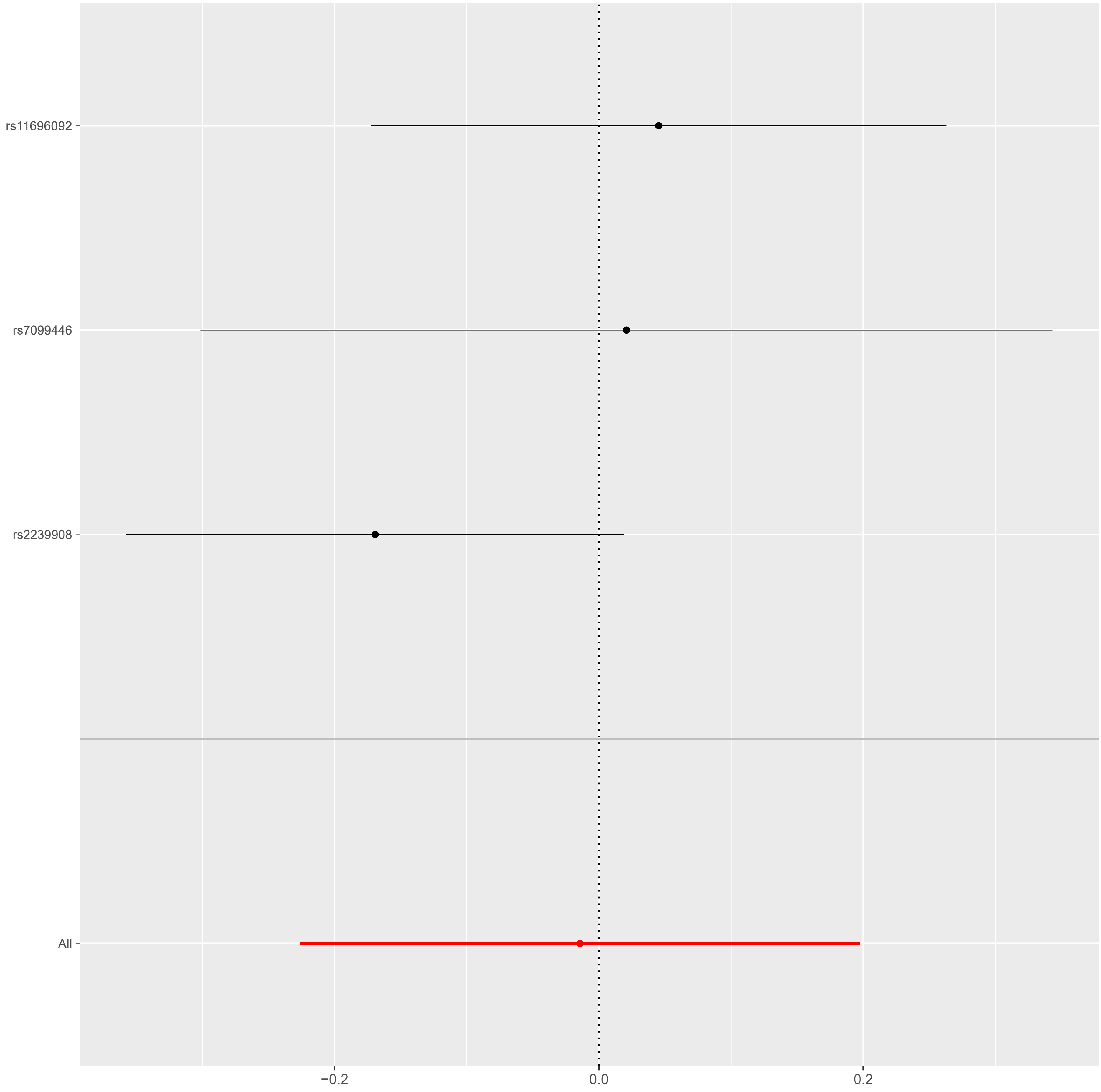

MR leave−one−out sensitivity analysis for WISP-1 on osteoporosis based on Finn trait

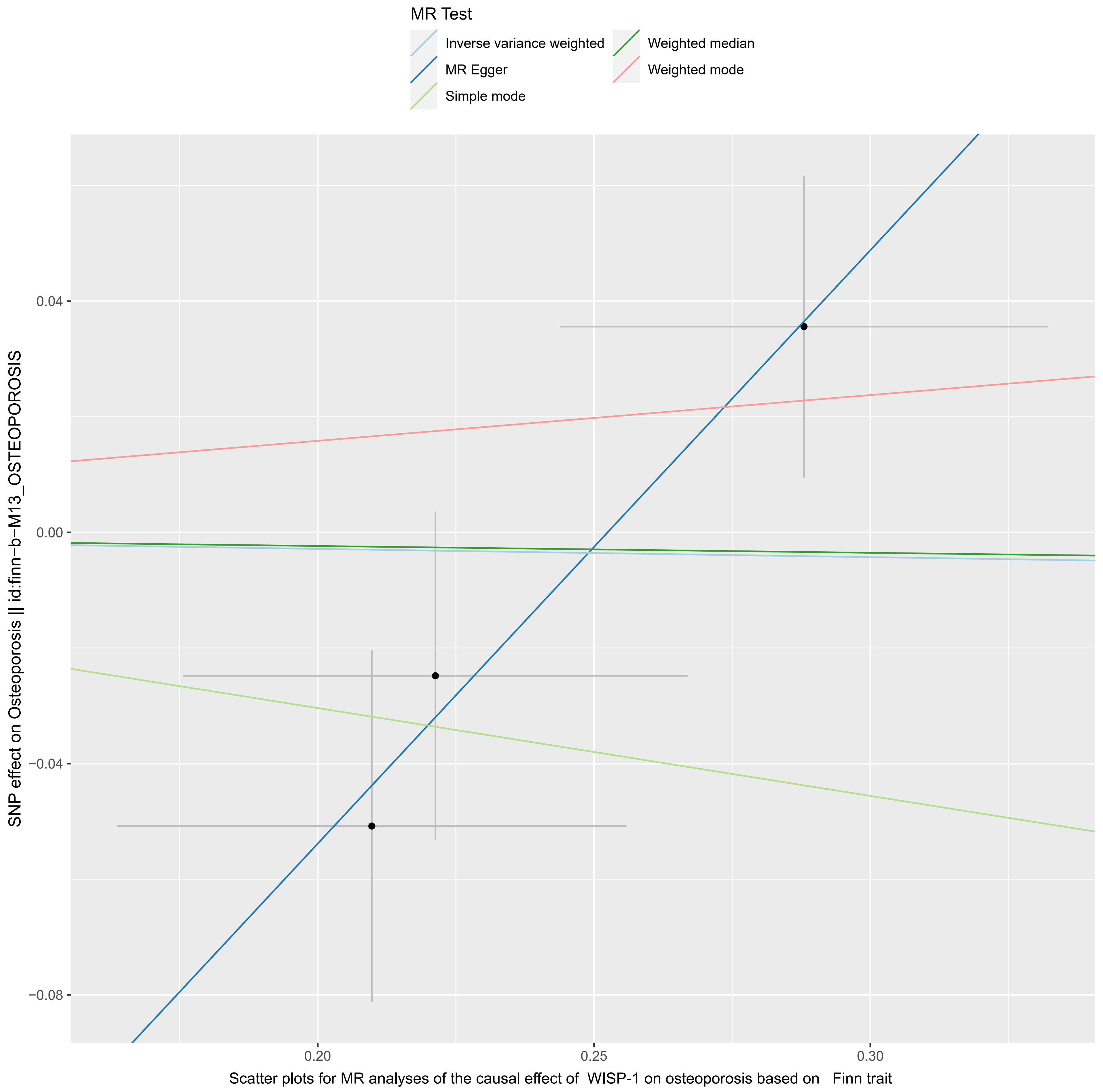
MR Test
Inverse variance weighted
MR Egger
Simple mode
Weighted median
Weighted mode
SNP effect on Osteoporosis || id:finn-b-M13_OSTEOPOROSIS
0.04
0.00
−0.04
−0.08
0.20
0.25
0.30
Scatter plots for MR analyses of the causal effect of WISP-1 on osteoporosis based on Finn trait

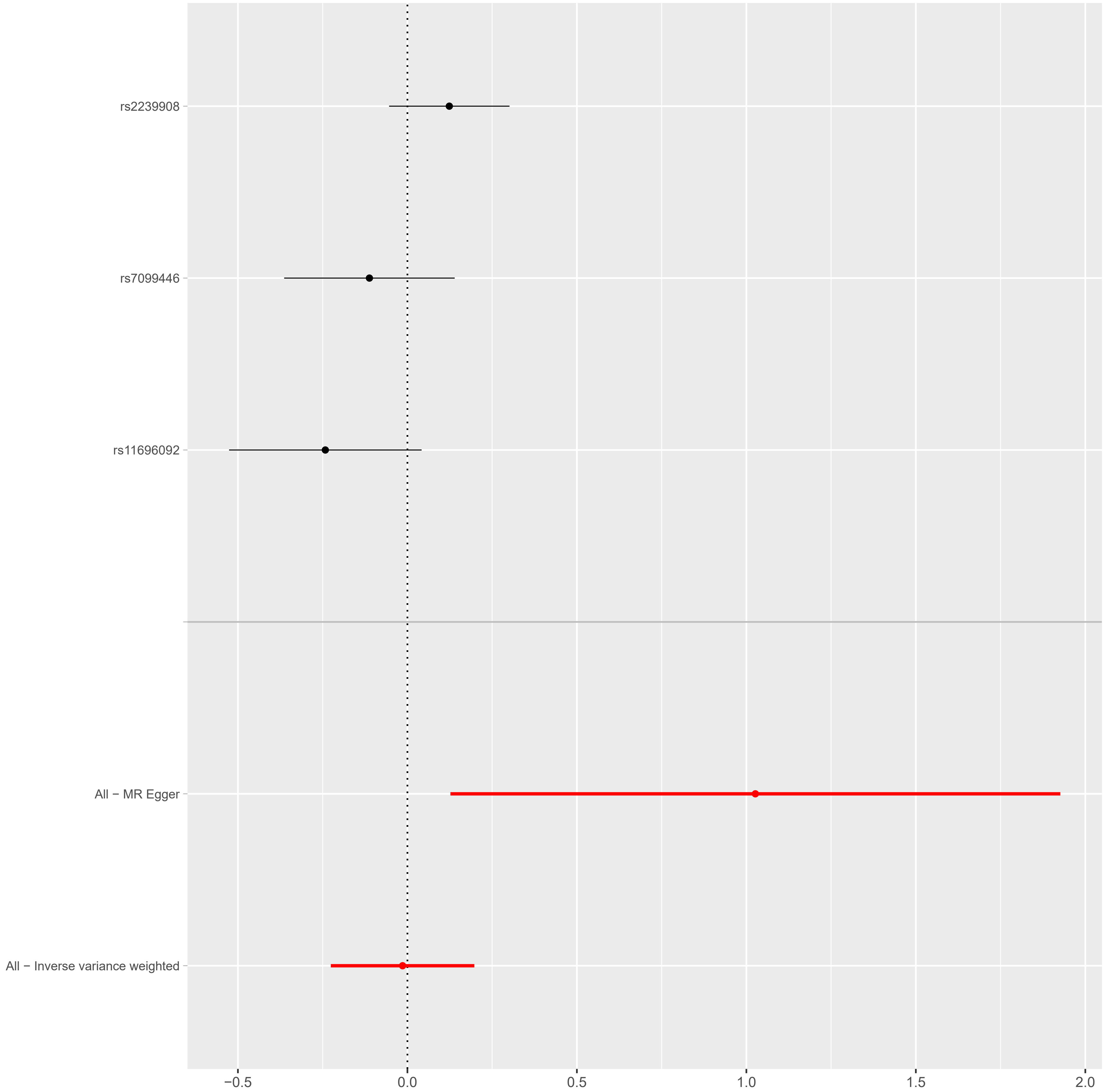

Forest plots for MR analyses of the causal effect of WISP-1 using each SNP singly on osteoporosis based on Finn trait

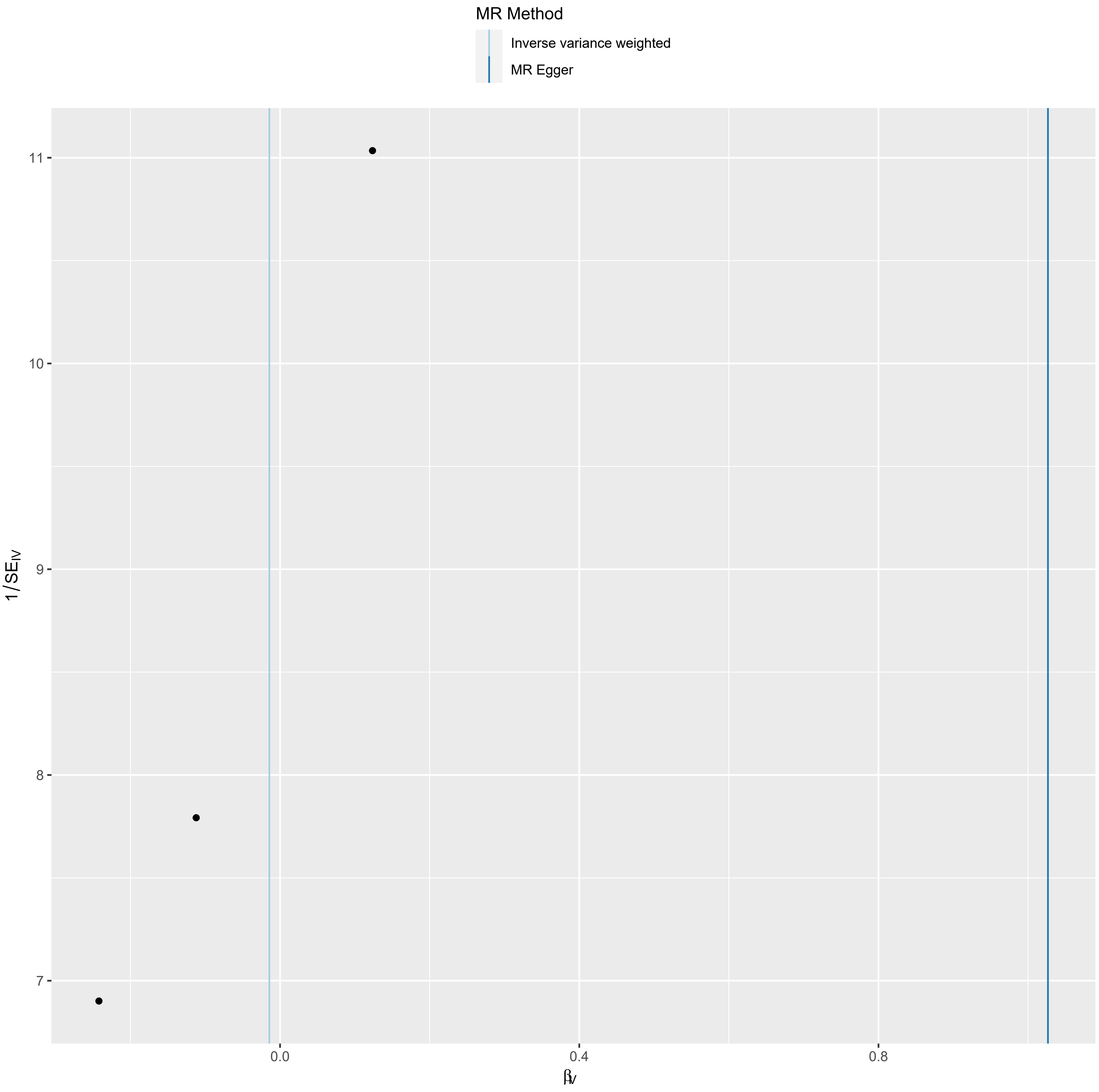

Funnal plots to assess heterogeneity for WISP-1 using all SNPs with the MR Egger and IVW methods

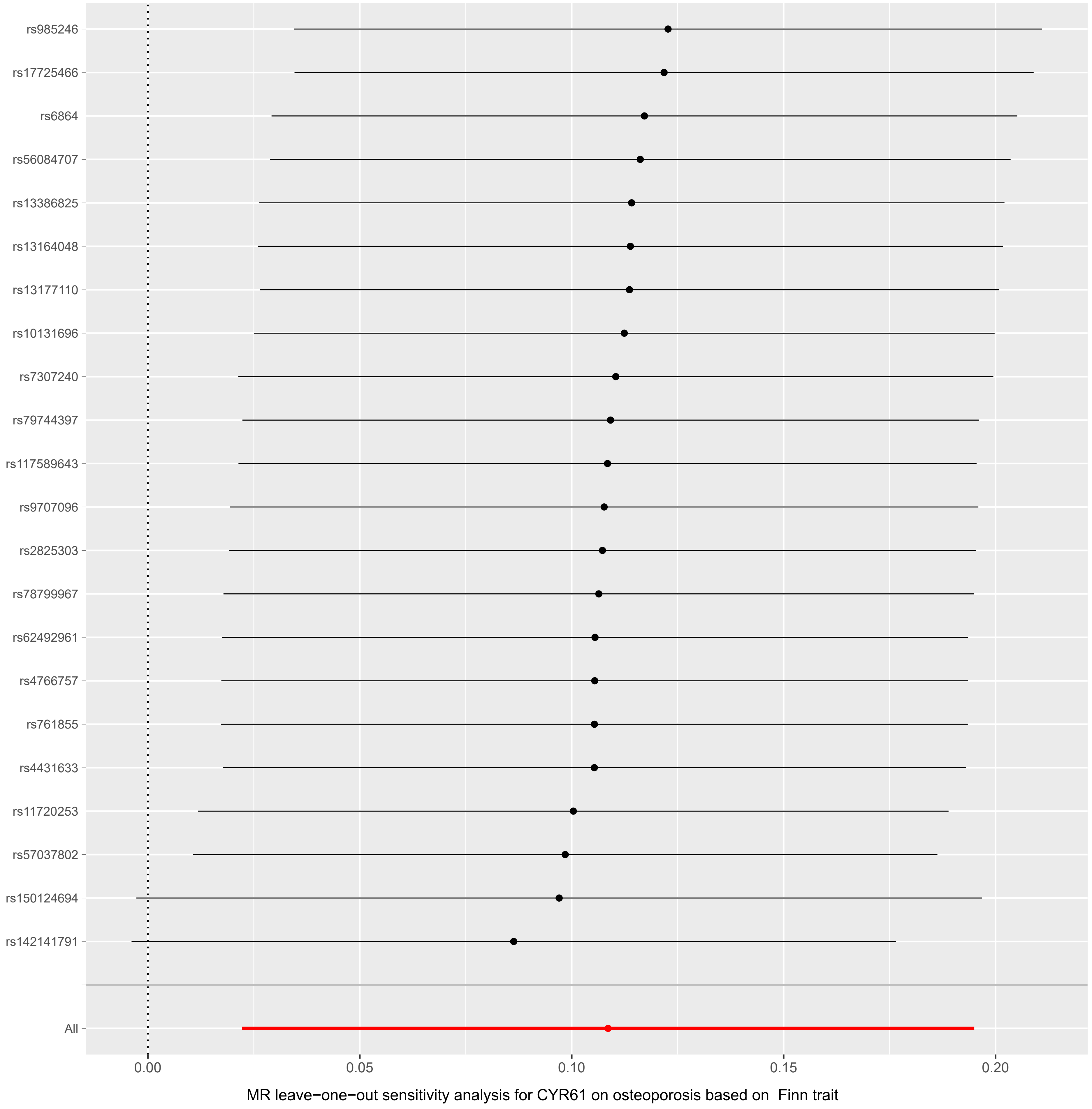
rs985246
rs17725466
rs6864
rs56084707
rs13386825
rs13164048
rs13177110
rs10131696
rs7307240
rs79744397
rs117589643
rs9707096
rs2825303
rs78799967
rs62492961
rs4766757
rs761855
rs4431633
rs11720253
rs57037802
rs150124694
rs142141791
All
0.00
0.05
0.10
0.15
0.20
MR leave−one−out sensitivity analysis for CYR61 on osteoporosis based on Finn trait

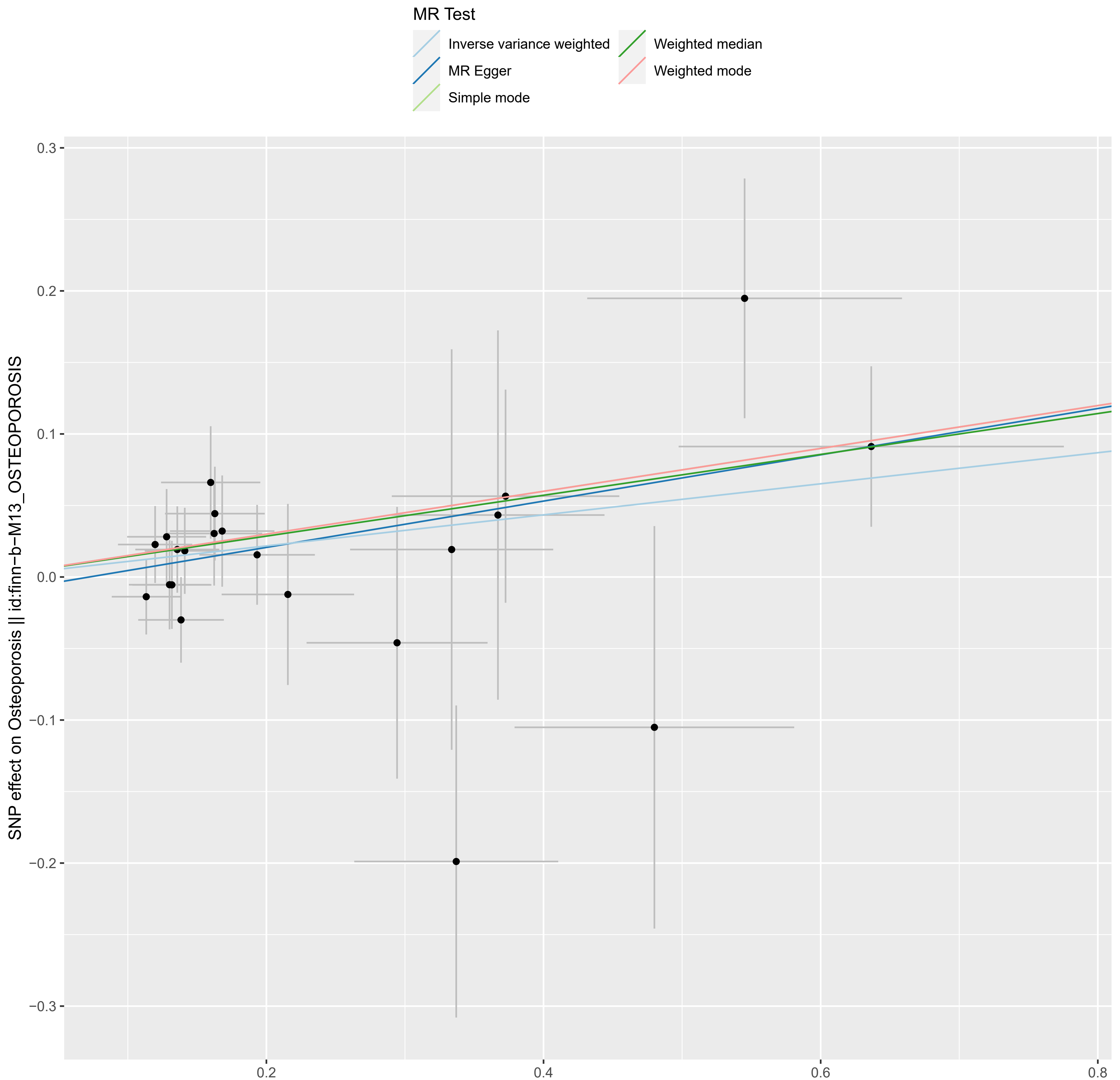

Scatter plots for MR analyses of the causal effect of CYR61 on osteoporosis based on Finn trait

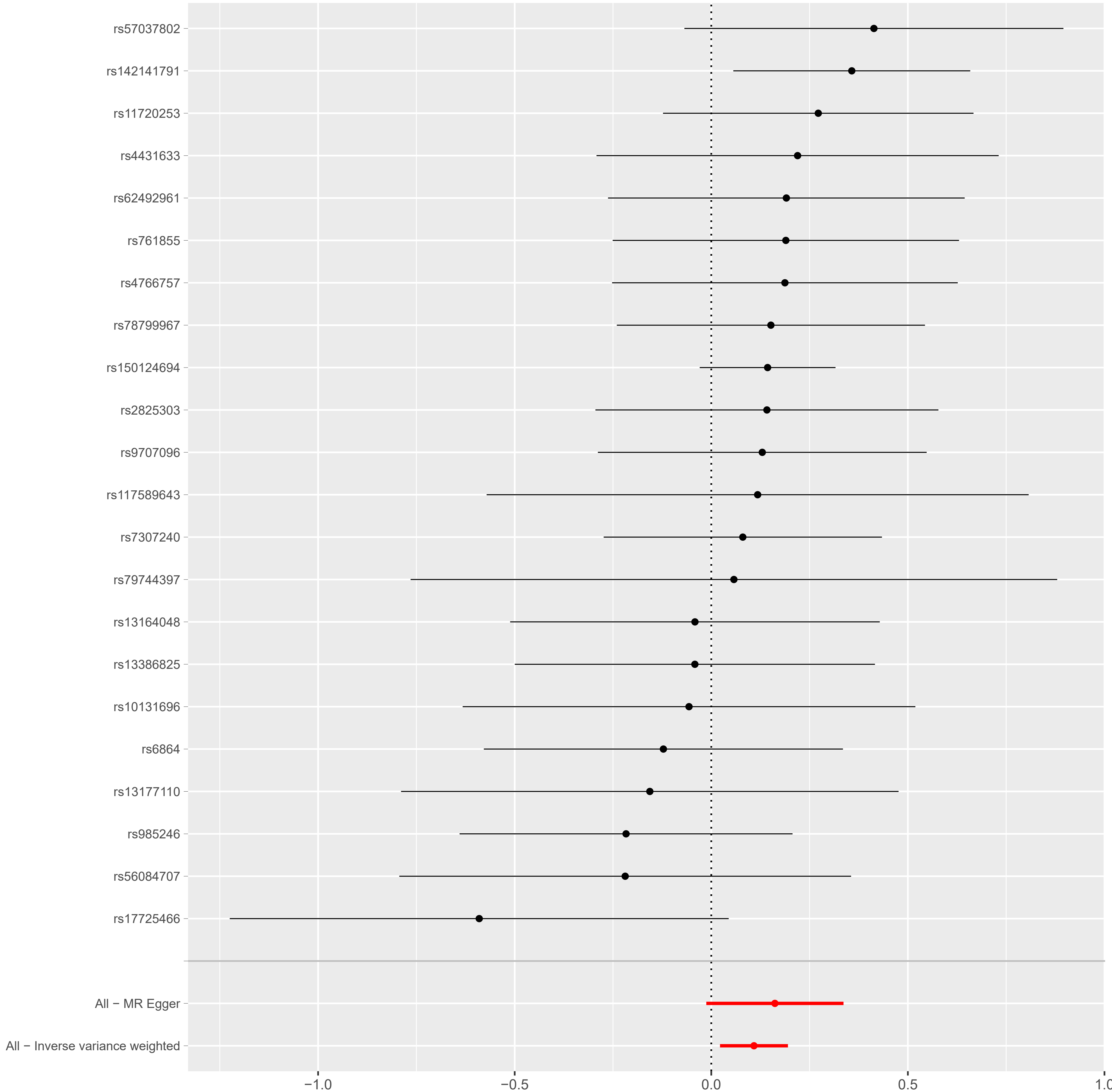

Forest plots for MR analyses of the causal effect of CYR61 using each SNP singly on osteoporosis based on Finn trait

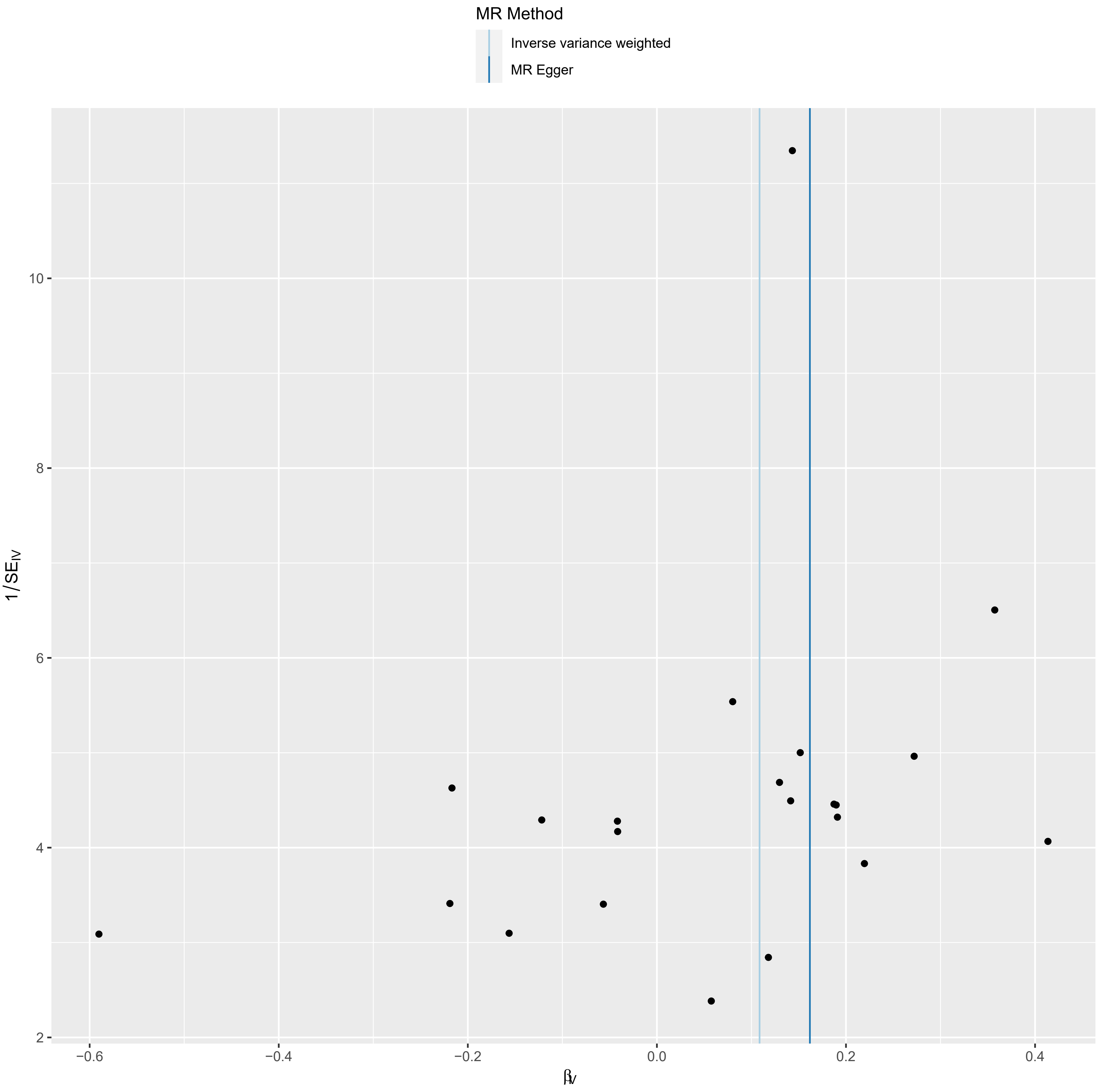

Funnal plots to assess heterogeneity for CYR61 using all SNPs with the MR Egger and IVW methods
